# Application of Proteomic Workflows for the Identification of Biomarkers for the Retrospective Verification of Sulfur Mustard Intoxication

**DOI:** 10.1021/acs.analchem.5c07620

**Published:** 2026-04-01

**Authors:** Gianin Thomann, Maximilian Brackmann, Christian G. Bochet, Christophe Curty

**Affiliations:** † 30868 Spiez Laboratory, Federal Office for Civil Protection, 3700 Spiez, Switzerland; ‡ Department of Chemistry, 27211 University of Fribourg, 1700 Fribourg, Switzerland

## Abstract

The family of sulfur mustards, as potent alkylating agents, has been used as chemical warfare agents for over a century. The retrospective verification of exposure and, hence, the confirmation of alleged use of such agents, remains a challenging task with high demand for resources in the identification, preparation, and analysis of potential specific biomarkers. We report a facile, high-throughput methodology appropriate for biomedical samples using proteomic workflows and the implementation of statistical models. Following the strategy, full proteome analysis of blood serum exposed to three different sulfur mustard representatives, namely, mustard gas, sesquimustard, and O-mustard, revealed 270 potential biomarkers with 213 novel alkylation sites. Targeted sample preparation by human serum albumin enrichment using Cibacron Blue-modified magnetic beads and analysis in prm-PASEF mode allowed the unequivocal retrospective verification of in vitro exposure to a level of 5 μM final concentration of agent in serum. Finally, the synthesis of four alkylated amino acid building blocks (Glu, Asp, Cys, and His) and their implementation in four different representative peptide sequences are demonstrated.

## Graphical abstract

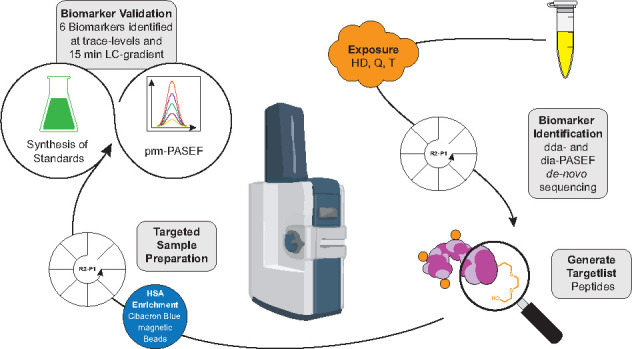

## Introduction

The production, stockpiling, and use of chemical warfare agents (CWAs) are prohibited by the Chemical Weapons Convention (CWC). It lists certain chemicals in three schedules in its annex and is enforced by the Organization for the Prohibition of Chemical Weapons (OPCW).^1^ In the past years, the use of different CWAs and, in particular, mustard gas (HD) as a representative of the family of sulfur mustards (SMs) regained international attention due to multiple uses in conflicts, predominantly in the Syrian Civil War,^2–4^ highlighting the persistent threat posed by CWAs. The family of SM features a total of nine agents, inter alia, HD (bis­(2-chloroethyl)­sulfide) as well as nonvolatile sesquimustard (1,2-bis­(2-chloroethylthio)­ethane, Q) and O-mustard (bis­(2-chloroethylthioethyl) ether, T) with the corresponding structures in Figure 1A. The latter two are generally present as impurities or intentionally added to HD to adjust the physical properties as well as the vesicancy of the agent.^5^

**Figure 1:**
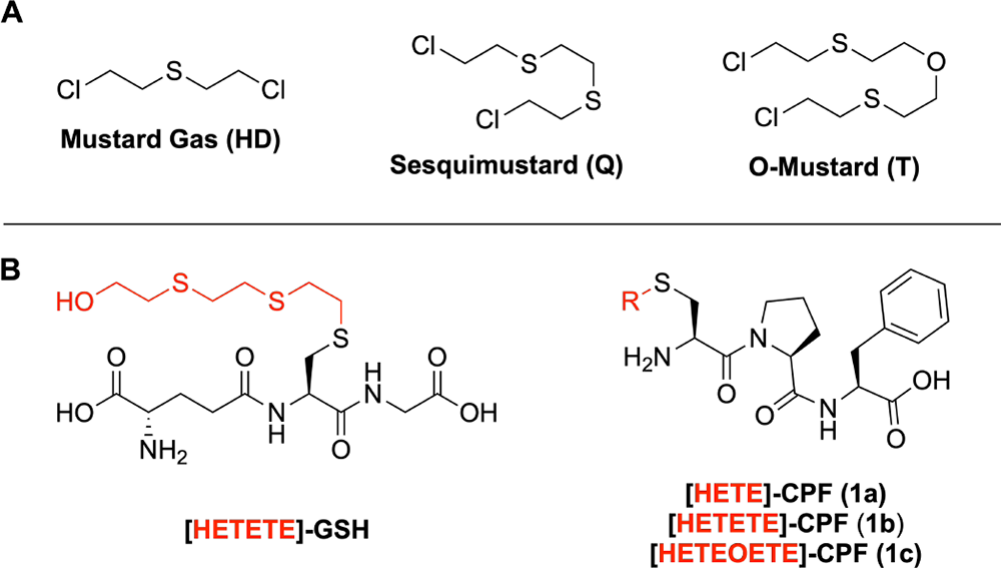
Family of sulfur mustards. (A) Three known representatives of the SM family. All are listed in the annex of the CWC. (B) Sesquimustard adducts to glutathione (GSH)—as an example for a metabolomic biomarker—and CPF as a peptide from HSA. In both cases, the corresponding adducts of HD (**1a**) and T (**1c**) are also already known.

SMs form an equilibrium with their thiiranium analogues via intramolecular nucleophilic substitution and, thus, are excellent alkylating agents.^6^ From a physiological perspective, this characteristic leads to respiratory distress upon inhalation,^7,8^ skin blisters^9–11^ in combination with apoptosis,^12^ and in the long-term, potentially cancer.^13,14^ On a molecular level, these effects are detected in the proteome, metabolome, and genome, generally following adductomic approaches.^15,16^

The retrospective verification of exposure in environmental and biomedical specimens is a key aspect of enforcing the CWC. For this, the detection of degradation products in the environment and biomedical samples, as well as adducts on metabolites or proteins, has been proven to be a suitable approach in terms of specificity and selectivity. To date, a large number of biomarkers for exposure to SMs have been described, most importantly intermediates of the beta-lyase metabolism,^17,18^ modified glutathione^19–21^ (GSH), and adducts on human serum albumin (HSA, Figure 1B). For the latter, the isolation of the cysteine-34 (Cys^34^) adduct marks today’s gold standard in the retrospective verification of exposure of biomedical specimens to HD.^22–24^ First described in 1999, Proteinase K (ProtK) digestion of purified blood plasma or serum produces a tripeptide with a cysteine-proline-phenylalanine (Cys-Pro-Phe, CPF) sequence, of which Cys can be modified ([HETE]-CPF, **1a**).

Recent works demonstrate the formation of the corresponding tripeptides after treatment of HSA or blood plasma with different SMs and mixtures thereof, in particular Q and T, resulting in the hydroxyethyl-thioethyl-thioethyl ([HETETE]-CPF, **1b** in Figure 1B)^25,26^ and hydroxyethyl-thioethyl-oxyethyl-thioethyl ([HETEOETE]-CPF, **1c**)^27^ adducts, respectively, after digestion with ProtK. Treatment of HSA with pronase leads to the formation of the corresponding CP dipeptide adducts.^28,29^ In addition to Cys, other amino acids have been shown to be modified by HD, namely, methionine (Met, M),^30,31^ histidine (His, H),^32,33^ glutamic acid (Glu, E),^34^ and N-terminal valine (Val, V).^35,36^ In a recent study, Chen and co-workers demonstrated bottom-up proteomics as a very promising approach for the identification of novel alkylation sites in HSA,^37^ although observing a lack of sensitivity in tryptic peptides and, hence, using other non-specific proteases for analysis of samples at low exposure levels.

In general, these approaches require large sample amounts and targeted analyses, resulting in reduced information on the matrix and, consequently, limiting the application of statistical approaches during analysis. Additionally, different sample preparation approaches and derivatization techniques are applied depending on the suspected CWAs, emphasizing the lack of more general methodologies. Finally, the identification of novel peptides is very challenging and demands different derivatization techniques, as presented in the literature mentioned previously. To our knowledge, the symbiosis of proteomic workflows, data mining, and validation via organic synthesis, enabling higher throughput and increasing identification confidence, is still underdeveloped in the adductomic field.

The present study addresses this issue by applying a broader, non-specific, high-throughput, and scalable proteomic sample preparation based on tryptic digest and subsequent analysis via nanoliquid chromatography-trapped ion mobility quadrupole time-of-flight mass spectrometry (nLC-timsTOF) for the identification of novel functionalization sites of SMs in human blood serum. For this purpose, data-dependent acquisition parallel accumulation serial fragmentation (dda-PASEF)^38^ methods to generate spectral libraries for subsequent measurements in data-independent acquisition (dia-PASEF) mode were used (Figure 2A). To enhance the sensitivity, a semiautomated enrichment strategy for HSA based on Cibacron Blue-modified magnetic beads (CibaMaBs) was applied, and the samples were analyzed with a parallel reaction monitoring (prm-PASEF) method (Figure 2B). Subsequently, we characterized a selection of biomarkers using synthetically prepared substrates (Figure 2C).

**Figure 2:**
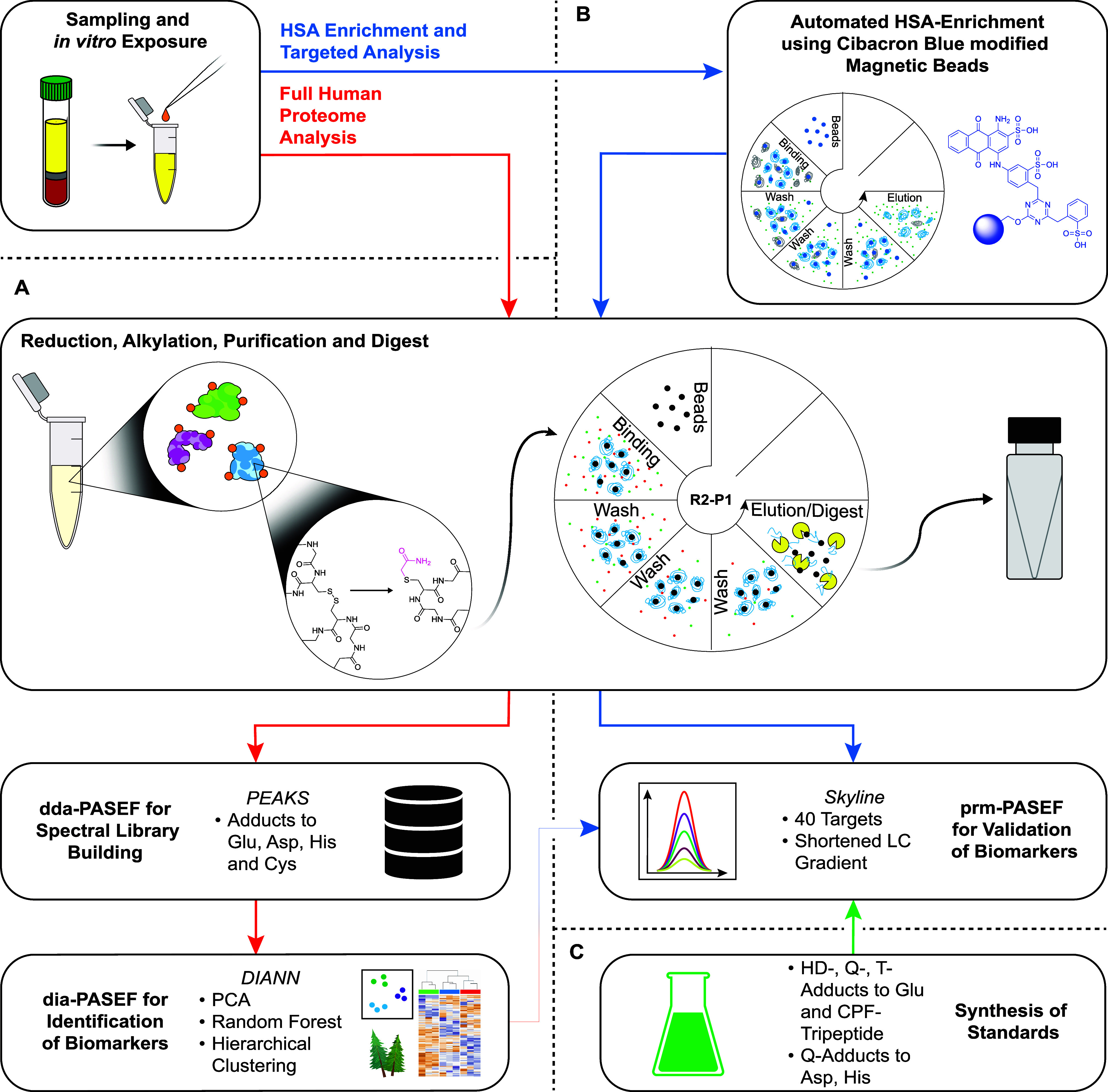
General approach for the identification of potential biomarkers for exposure to SMs using in vitro exposed blood serum and targeted analysis thereof. (A) Blood serum was exposed in vitro to different SMs and subjected to the R2-P1 protocol for full proteome analysis. The obtained samples were then analyzed via dda-PASEF to generate a spectral library and dia-PASEF to identify potential biomarkers and generate the target list (red pathway). (B) Targeted workflow started with HSA enrichment of blood serum exposed to Q using Cibacron Blue-modified magnetic beads prior to the R2-P1 protocol. The samples were analyzed in prm-PASEF mode using a target list based on dia-PASEF data and the previously generated spectral library (blue pathway). (C) General strategy for the synthesis of SM-adducts to Glu, Asp, His, and Cys is demonstrated, and the building blocks are integrated into a selection of representative peptides (green pathway). HD: mustard gas; Q, sesquimustard; T: O-mustard; Asp: aspartic acid; Cys: cysteine; dda: data-dependent acquisition; dia: data-independent acquisition; Glu: glutamic acid; His: histidine; HSA: human serum albumin; PASEF: parallel accumulation serial fragmentation; PCA: principal component analysis; prm: parallel reaction monitoring; and R2-P1: rapid-robotic proteomics protocol; all software names are in italic.

Eventually, we tested our approach using three representatives of the SM family. As introduced above, this family and their alkylation capability have been thoroughly investigated in the past. This makes the representatives of the aforementioned family suitable model substrates for testing and validating the proposed approach.

## Experimental Section

### Chemicals

SMs are toxic chemicals listed in the CWC. Likewise, chlorinated intermediates in the synthesis are considered as hemimustards. All of these chemicals must be handled by professional personnel only under appropriate safety conditions. All CWAs used in this study were provided in-house, along with diol starting materials for the synthesis. For all biomarkers and their intermediates, the synthesis details can be found in Section 1.3 of the Supporting Information. All other chemicals were commercially available.

### Exposure of Blood Serum to SMs

Blood serum was voluntarily provided by seven healthy individuals, four males and three females. The solutions of SMs (HD, Q, or T) in CH_3_CN (or plain CH_3_CN for non-exposed serum samples) were added to human blood serum (990 μL) in exposure levels (final concentration of SMs) of 5 mM (high), 50 μM (low, only Q), and 5 μM (trace, only Q), and the samples incubated at 37 °C for 24 h. After termination of incubation, the samples were stored at −23 °C until further use. For all experiments, quality control samples were prepared as equal-part mixtures of the corresponding non-exposed serum samples. The resulting samples were subjected to sample preparation along with exposed and non-exposed samples.

### Proteomic Sample Preparation and Enzymatic Digestion

Bottom-up proteomics samples were prepared following an automated protocol via single-pot, solid-phase enhanced sample preparation (SP3)^39^ implemented in the rapid-robotic proteomics (R2-P1) protocol^40^ using dithiothreitol (DTT) and iodoacetamide (IAA) for reduction and alkylation of disulfide bonds. The automated protocol was performed on a Thermo Fisher Scientific KingFisher APEX using carboxylated bead suspensions in H_2_O (Cytiva 1 μm avg particle size - 5% suspension, Cytiva 0.70 μm – 1.10 μm particle size - 5% suspension; 1/1, v/v; 2 mL). A detailed protocol is presented in Table S4 of the Supporting Information.

HSA enrichment was performed using Cibacron Blue-modified magnetic beads (CibaMaBs) prepared by the treatment of MagReSyn Hydroxyl beads (20 mg mL^–1^, 5 mL) with Cibacron Blue 3G-A solution (5 mg mL^–1^ in HPLC-grade H_2_O, 5 mL) and 2 M NaOH (5 mL) at 50 °C and 1000 rpm for 2 h. After several washes with HPLC-grade H_2_O, the beads were resuspended in HPLC-grade H_2_O to afford a 50 mg mL^–1^ solution. The resulting CibaMaBs were then implemented in a semiautomated protocol using a KingFisher APEX. Subsequently, the samples were reduced, alkylated, and purified following the R2-P1 protocol. Enzymatic digestion was performed using trypsin/Lys-C (5 μg mL^–1^, Promega) or ProtK (100 μg mL^–1^, Merck) in 50 mM NH_4_HCO_3_ buffer at 37 °C for 4 h or 50 °C for 90 min, respectively.

### nLC-tims-qTOF Analysis

Proteomic analysis via nLC-tims-qTOF was performed on a Bruker timsTOF Pro II coupled to a Bruker nanoElute equipped with an IonOpticks Aurora Elite (150 mm × 0.075 mm, 1.7 μm particle size) at 50 °C with the following eluents: (A) H_2_O + 0.1% FoA and (B) CH_3_CN + 0.1% FoA with 1 μL injection volume. An elution program starting with a linear gradient from 2% B to 35% B in 25 min and then from 35 to 95% in 0.5 min was performed. This composition was held for 5.5 min. In trace analysis, a shortened gradient was used, also referred to as a short gradient: A linear gradient from 2% B to 50% B in 12.5 min and then from 50 to 95% in 2.5 min was performed.

### Data Processing and Evaluation

Proteomics data evaluation of dda measurements was performed using PEAKS Studio (10.6 build 2020 1221, Bioinformatics Solutions Inc.) using an error tolerance of 15 ppm on precursor mass and 0.05 Da for the fragment ion mass. The digest mode was set to specific, with a maximum missed cleavages per peptide of 2. Human proteome (UP000005640) was used as the database with post-translational modifications (PTMs), as presented in Table S3 of the Supporting Information. Dia measurements were evaluated with DIANN (Version 1.9+) using the spectral library exported from PEAKS and converted to the needed format for DIANN via an in-house Python script. Prm-PASEF data were processed with Skyline (24.1.0.199, 64-bit). All data were evaluated using in-house R scripts.

## Results and Discussion

### Proteomic Analysis of Blood Serum Exposed to Q

For the identification of potential modification sites, blood serum was exposed in vitro to Q at two different exposure levels of the agent in serum: 5 mM (high) and 50 μM (low) final concentrations. While the former probably exceeds the concentrations that are relevant for in vivo studies, it was nonetheless particularly important for optimal spectral library building within the workflow that has been proposed. After incubation with the agent, the samples were prepared for nLC-timsTOF analysis by SP3 ^39^ implemented in the R2-P1 protocol^40^ using carboxylated magnetic beads followed by tryptic digest (see Section 1.2, Supporting Information). This technique allows a simple, robust, and high-throughput sample preparation with rather small sample amounts (less than 1 μL blood serum per sample) compared to current techniques in this field.

Dictated by the nature of the workup protocol, potential interference of the agent with the formed peptides upon enzymatic digestion can be neglected. This can be attributed to quenching of the putative remaining agent with DTT during reduction and alkylation of the proteins in conjunction with the multiple washing steps incorporated within the protocol using wet ethanol.

As shown in Figure 3A, principal component analysis (PCA) of samples measured in dia-PASEF mode allowed us to readily distinguish high-level exposed samples from non-exposed serum samples in the first dimension. At lower exposure levels, no significant variation in the samples could be identified compared to non-exposed serum samples. The corresponding loading plot of the PCA depicted in Figure 3B shows the separation between high-level exposure samples to other treatments primarily being dictated by HETETE-modified peptides (wine red); the alkylated peptides which are inversely proportional to PC1 are assumed to be potential biomarkers for an exposure to Q. Furthermore, we used an orthogonal approach to validate these markers via random forest algorithm to correlate loading in the PCA to the importance of individual features, i.e., peptides.

**Figure 3:**
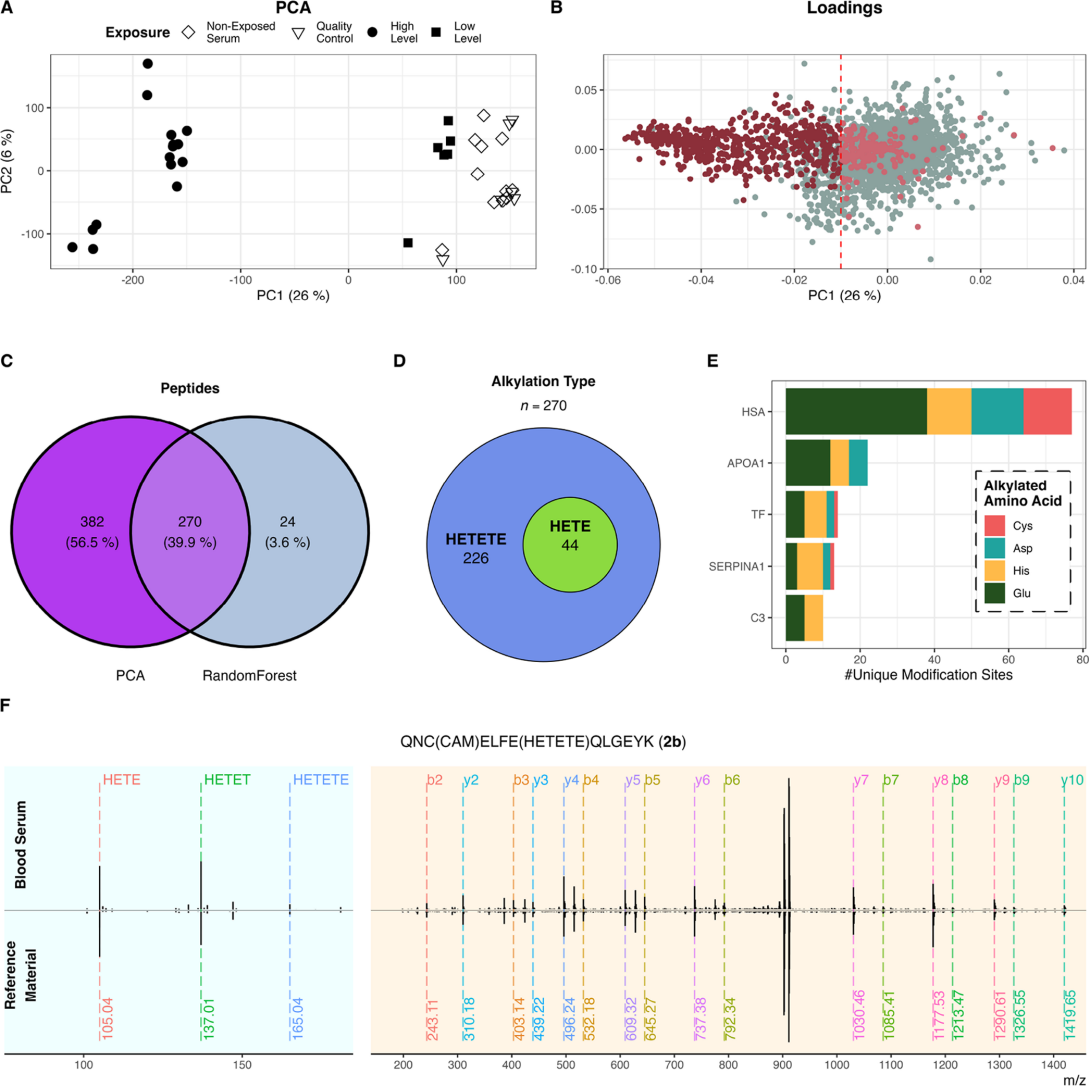
Identification of potential biomarkers in blood serum (biological replicates, tryptic digest) exposed to different levels of Q, i.e., 5.0 mM (high-level, *n* = 15) and 50 μM (low-level, *n* = 6). (A) PCA analysis allows good separation between treated and control samples but only at high-level exposure. The subclustering of some samples—especially visible for treated ones at high exposure level—originates from residual batch effects. Quality control: equal part mixture of non-exposed serum samples prior to digest. (B) Loading plot distinguishing peptides bearing at least one HETETE-alkylated site (colored) and native peptides, i.e., not alkylated by Q (gray). The dashed, red line refers to the cutoff value for the comparison with the random forest. (C) Venn diagram of most important features, i.e., peptides, in PCA (loadings (PC1) < 0.01 and HETETE-modified, i.e., wine red in loadings plot) and random forest (non-zero mean decrease Gini, 5-fold cross-validation). (D) Of the 270 potential biomarkers identified in both approaches, 44 peptides were also found as their corresponding HETE-alkylated peptides, which would only be expected when HD was used as the CWA. (E) Distribution of unique modification sites of peptides in the intersection area of the Venn diagram on proteins and specification of the alkylated amino acid. HSA: human serum albumin; APOA1: apolipoprotein A-I; TF: serotransferrin; SERPINA1: alpha-1-antitrypsin; and C3: complement C3. (F) MS2 of HETETE-modified **2b** found in blood serum (top) compared to synthetically prepared reference material (bottom, see Synthesis of Standards section) at a 24 min retention time and 1.12 V s cm^–2^ ion mobility. Yellow: measurement in standard dda-PASEF mode; blue: measurement in low-mass dda-PASEF.

As depicted by the Venn diagram in Figure 3C, a total of 270 modified peptides were found to be promising biomarkers for future investigations. A full list of the found peptides is given in Table S11 in the Supporting Information. From the present data, 232 different modification sites, of which 213 are novel to our knowledge, have been identified. This number of biomarkers is remarkable and superior to that of known approaches in terms of time and material effort. We also identified a few peptides modified at two positions. In contrast to using the full peptide space, filtering for the mentioned important peptides allowed the successful separation of the low-level exposed samples from control samples (see Figure S8, Supporting Information), as well as correct classification via random forest.

Depending on which SM was used, different adducts are expected to be formed. Interestingly, when treating blood serum with Q, HETE-alkylated peptides are also observed, which actually would be expected only when using HD as CWA. Specifically, from the 270 potential biomarkers evaluated by PCA and random forest, 44 also occur as the corresponding HETE-modified peptides in blood serum exposed to Q (Figure 3D). This effect is already known from decontamination experiments,^41^ where it has been computationally rationalized that hydrolysis of Q can occur via two pathways: (A) attack of the hydroxide on the carbon of the thiiranium ion leads to the formation of desired HETETE adduct or (B) attack on the α carbon provoking the elimination of thiiran and formation of the corresponding HETE-alkylation (see Scheme S2, Supporting Information).

Our results indicate that Q primarily alkylates glutamic acid in HSA, which is in accordance with the literature^37^ (Figure 3E). In detail, 74 different modification sites are identified in HSA, which predominantly occupy the surface of the protein (Figure S9, Supporting Information). Notably, no significant difference was found between samples from male or female donors. Also, we observed reduced intensity of some glutamic acid adducts upon longer digestion times and suspect that this arises from partial hydrolysis of these adducts by saponification of the γ-ester (see Figures S2 and S3, Supporting Information).

For unambiguous identification of the proposed biomarkers by the present approach, samples exposed to Q were also measured in a so-called low-mass dda-PASEF, where the sensitivity of the spectrometer is shifted to lower mass-to-charge ratios to allow the detection of the fragments from the alkyl side chain (see Scheme S1, Supporting Information), in specific 105.04 *m*/*z* (HETE, C_4_H_9_SO), 137.01 *m*/*z* (HETET, C_4_H_9_S_2_O), and 165.01 *m*/*z* (HETETE, C_6_H_13_S_2_O) with a mass accuracy of 20 ppm on MS1 and MS2 levels. This manual evaluation revealed 25 different peptides (see Table S10, Supporting Information), inter alia, peptide **2b** whose spectrum is depicted in Figure 3F (top). For this peptide, the amino acid sequence and the modification sites were unambiguously identified by the comparison to the synthetically prepared analogue (bottom; see Synthesis of Standards section).

### Enrichment of HSA and Subsequent Targeted Analysis

With the aim to increase sensitivity of the demonstrated approach, we implemented an enrichment strategy for HSA to decrease background and enable higher on-column loading of the targeted peptides without decreasing separation efficiency. For this, hydroxylated beads were modified with Cibacron Blue under basic conditions. This dye is known for its semiselectivity to HSA in affinity chromatography and, therefore, is potentially useful for the purification or depletion of this protein from a matrix.^42–45^ The resulting Cibacron Blue-modified magnetic beads (CibaMaBs) were then implemented in a semiautomated protocol prior to R2-P1. To the best of our knowledge, this is the first description of this tandem sample preparation method. To evaluate the enrichment efficacy, blood serum (1:100 dilution) was either treated following the optimized enrichment strategy or with CibaMaBs under non-selective isolation conditions, i.e., no salt additive during binding and washing steps, and compared to blood serum prepared by the standard R2-P1 protocol. Following this strategy leads to a reduction in protein counts of around 58% compared to simple full serum proteome sample preparation, indicating successful simplification of the matrix.

To investigate the enrichment quality of our approach, we further compared the individual protein quantities in the enriched samples to the residual proteins found in the binding fraction. This reveals a significant increase in HSA content in the former (Figure 4). Furthermore, several proteins are largely depleted using this strategy, including serotransferrin and immunoglobulin G, represented by its subchains. As depicted by the spot size, in general, multiple proteins with high abundance in the full blood serum proteome can be successfully depleted. These results underline the semiselectivity of the CibaMaBs toward HSA under the conditions used and confirm its suitability to enrich HSA to potentially lower the limit of detection.

**Figure 4:**
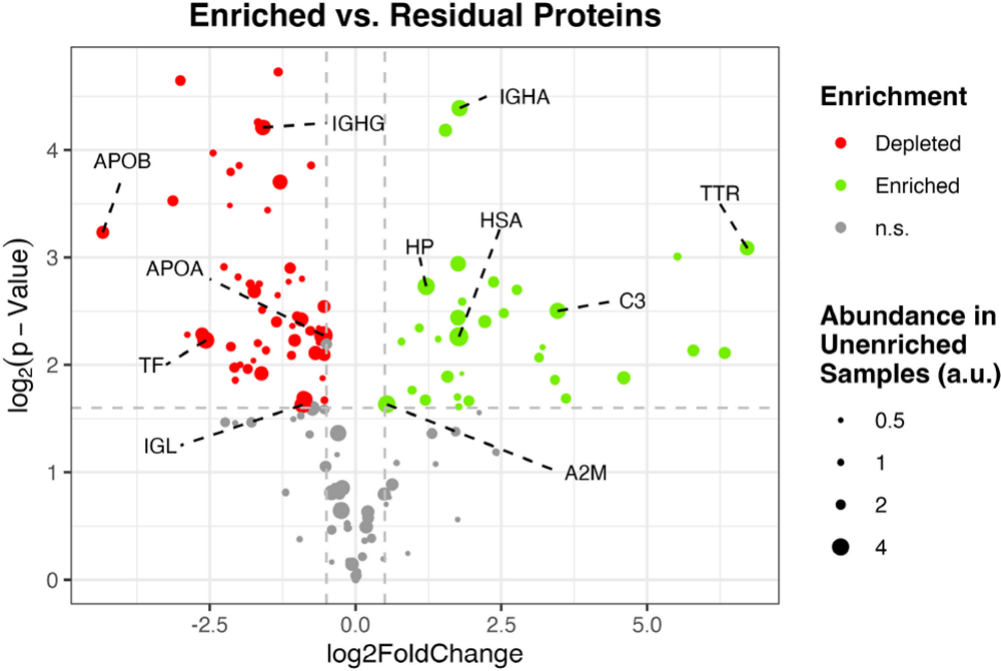
Evaluation of the enrichment and depletion efficacy of the developed semiautomated sample preparation strategy for HSA enrichment with CibaMaBs (technical replicates, *n* = 3, tryptic digest). The volcano plot compares the content of residual proteins during binding with CibaMaBs to those of the proteins observed upon elution. The size of the spots indicates the abundance in the full blood serum proteome. TF: serotransferrin; APOB: apolipoprotein B-100; IGL: immunoglobulin lambda-1 light chain; APOA: apolipoprotein A-I; TTR: transthyretin; C3: complement C3; A2M: alpha-2-macroglobulin; IGHA: immunoglobulin heavy constant alpha 1; IGHG: immunoglobulin heavy constant gamma 3; and HP: haptoglobin.

We used this approach for exposed blood serum to generate a target list for prm-PASEF measurements. As depicted in Figure 5, five peptides could be identified even at trace-level exposure (5.0 μM) with our instrumentation and using a 30 min gradient. We then challenged our approach by reducing the gradient time to 15 min for higher throughput, which led to a decrease in the number of identified peptides. Nonetheless, we observed three additional modified peptides at trace levels compared to the long gradient. Further, the verification of exposure at even lower levels, i.e., 0.5 μM, was still unsuccessful, confirming the lower limit of detection with the present instrumentation at around 5.0 μM of agent, i.e., approximately 10-fold the spiking concentration used in proficiency tests by the OPCW.

**Figure 5:**
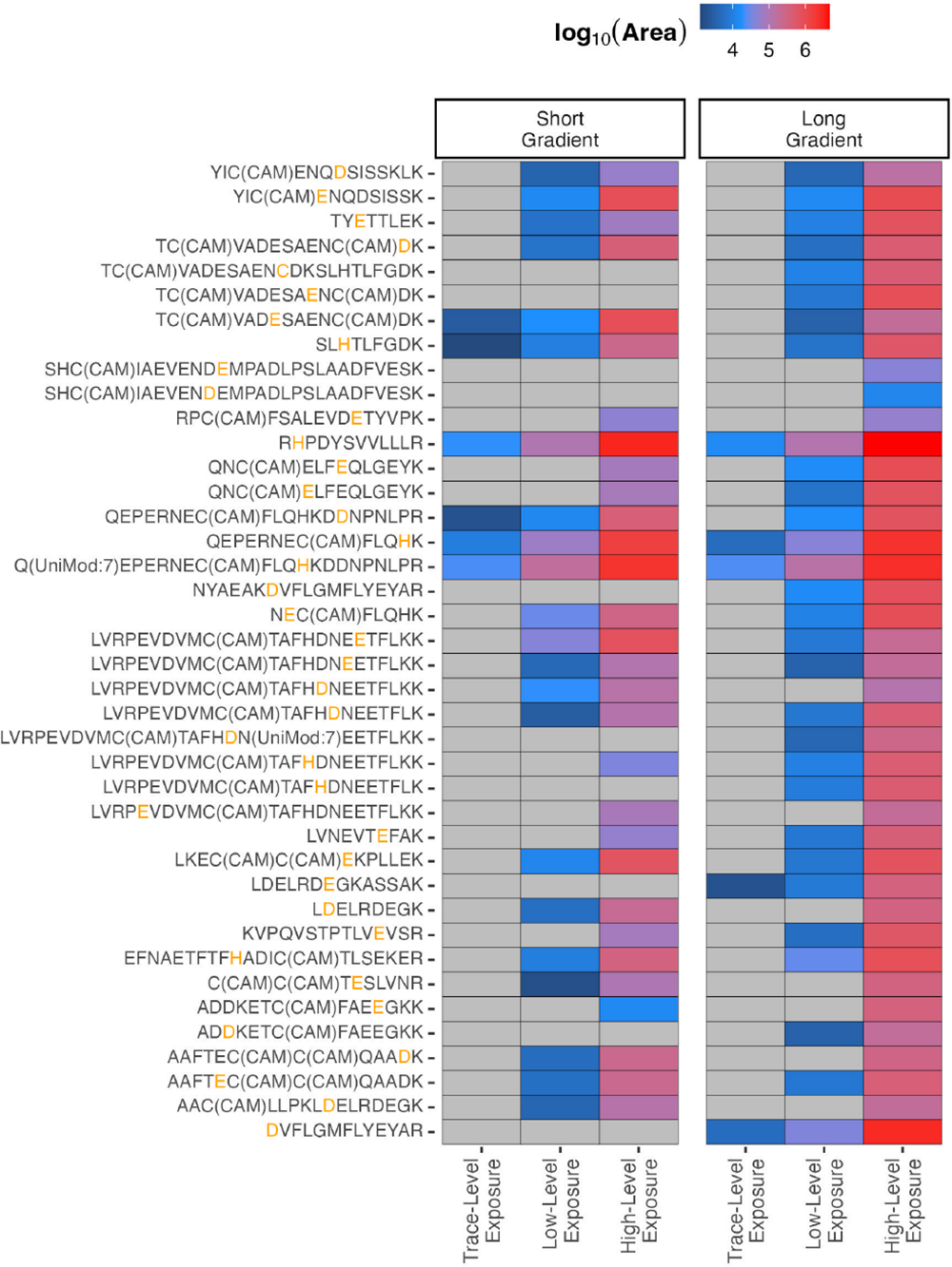
Targeted analysis of blood serum exposed to Q at three levels of exposure and after HSA enrichment using Cibacron Blue-modified beads and measurement in prm-PASEF mode (biological replicates, *n* = 3, tryptic digest). The strategy was evaluated using two different gradients (short: 15 min gradient; long: 30 min gradient, i.e., standard gradient used in untargeted analysis). Gray areas indicate intensity below the limit of detection of the peptide, and the modified amino acid is highlighted in orange.

### Comparison of HD-, Q-, and T-Adducts

To investigate the specificity of the marker peptides, we tested whether different representatives of the sulfur mustard family (HD, Q, and T) alkylate the same sites. For this, we performed a hierarchical clustering analysis of bottom-up proteomics data at high exposure levels and with anonymized modifications (Figure 6A). In summary, there are two important conclusions to be drawn from the clustering: (1) samples exposed to the same CWA cluster, as depicted by the dendrogram, and (2) all three SMs alkylate predominantly the same protein sites. Remarkably, the intensity distribution over the peptides varies between the utilized SM, thereby enabling the differentiation of clusters. This may allow the establishment of protocols for the identification of the applied SMs using an untargeted analysis.

**Figure 6:**
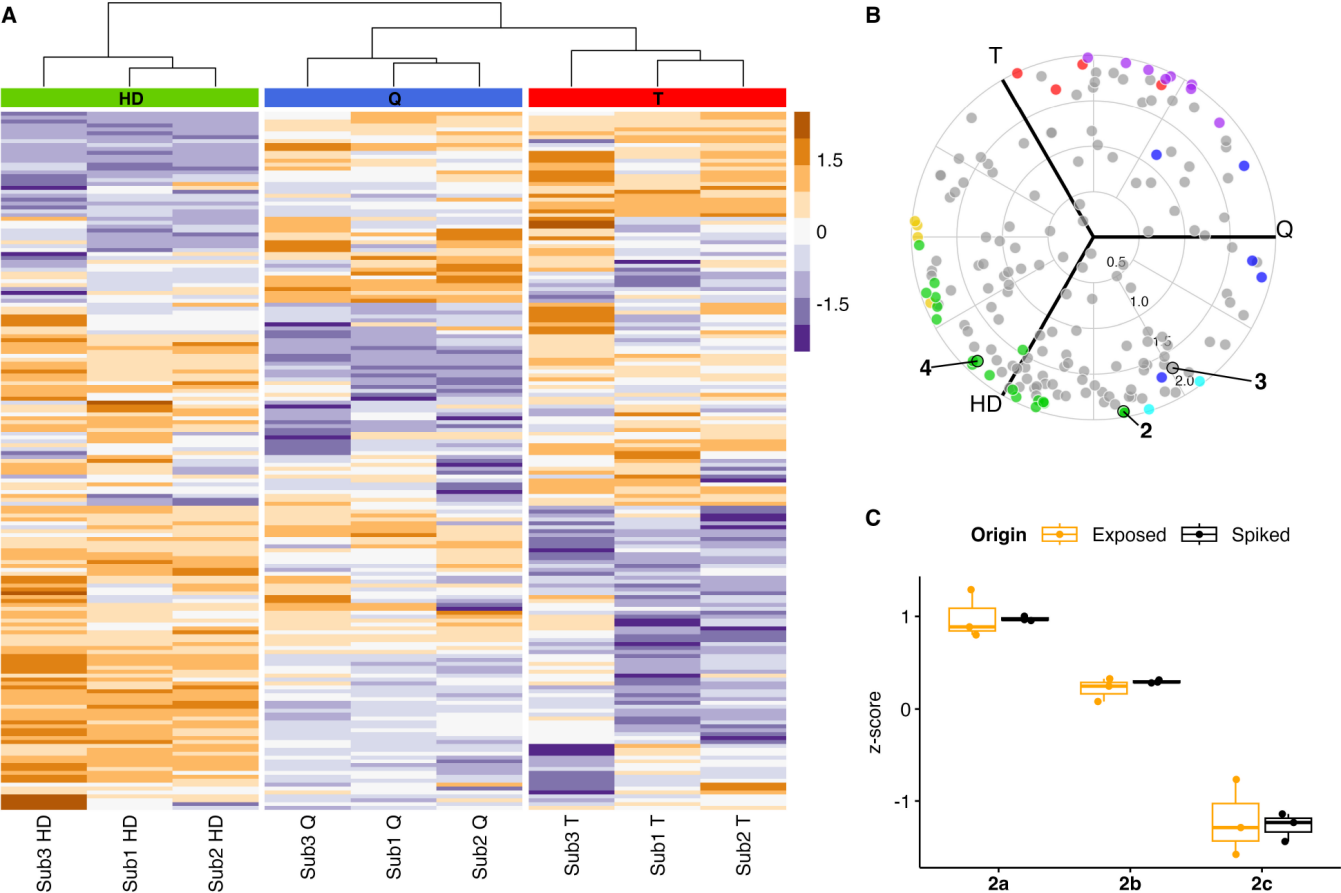
Comparison of adducts formed by HD, Q, and T (5.0 mM, biological replicates, *n* = 3, tryptic digest). (A) Hierarchical clustering of blood serum exposed to HD (green), Q (blue), and T (red) using Ward’s method (*n* = 3; log_2_-transformed; *z*-score normalized). Each row represents one alkylated peptide sequence, where the type of alkyl chain attached to the peptide was anonymized, and therefore, only the peptide sequence with generic modifications and the corresponding intensity was used for the clustering. (B) Two-dimensional projection of a three-correlation volcano plot using peptides with anonymized alkylations. Primary colors (red, blue, and green) represent peptides that are significantly more intense for one CWA compared to the other SM representatives. Similarly, secondary colors (yellow, purple, and cyan) indicate higher intensity for two SMs compared to exposure to the third. Three peptides are highlighted: QNC­(CAM)­ELFE*QLGEYK (**2**), SLHTLFGD*K (**3**), and RH*PDYSVVLLLR (**4**) as the sample substrates for the synthesis, where * denotes an HETE, HETETE, or HETEOETE modification. (C) Comparison of the relative intensities of peptide **2**, depending on the type of alkylation and origin, i.e., modified peptide measured in blood serum exposed to the different SMs or the corresponding synthetically prepared peptides spiked into blood serum in equimolar amounts (checked by HPLC-UV at 276 nm with L-tyrosine as internal standard) and measured intensity *z*-score normalized.

To test for significant deviation in intensity of each peptide between the applied CWA, a two-dimensional projection of a three-correlation volcano plot is given in Figure 6B. Several peptides appear with significantly higher intensity compared to their analogues upon treatment with another SM (colored points). This effect is caused by either a difference in ionization efficiency or deviating from alkylation preference. A preference could be rationalized by the difference in the vesicant powers of the agents. Nevertheless, Q has been shown to be 500 times more physiologically powerful than HD,^46^ which would result in a higher intensity of the corresponding peptides originating from Q compared to HD. Therefore, we spiked the synthesized peptides with corresponding modifications into untreated blood serum in equimolar concentrations.

As shown in Figure 6C, the intensity of peptide **2** differs significantly depending on the alkyl chain present in the sequence (black), which is in accordance with the results obtained from exposed blood serum (orange). Hence, the deviation can be rationalized by the difference in ionization efficiency of the respective peptide depending on the alkyl chain present. The variation in intensity highlights the need to consider the nature of the adduct for every analysis, especially in targeted analysis.

### Synthesis of Standards

For unambiguous identification of a peptide and verification of exposure, the synthesis of standards remains crucial in forensic analysis. For this, we demonstrate a synthesis approach of some of the identified peptides. First, a strategy for the preparation of alkylated glutamic acid, as presented for the example of the synthesis of **2**, was developed (Figure 7A). Commercially available diol **5** was methoxymethyl (MOM) protected, affording compounds **6** and undesired bisprotected **6′**. The former was further used in the esterification of the γ-carboxylic acid of **9** - obtained from Fmoc-Glu­(O^*t*^Bu)-OH (**7**) via esterification with dimethoxy-nitrobenzyl alcohol (Nvoc), yielding **8** and subsequent removal of the ^*t*^Bu group upon treatment with trifluoroacetic acid (TFA) - under optimized Yamaguchi conditions. Protection of the α-carboxylic acid with Nvoc was necessary due to the lack of selectivity in esterification and formation of undesired side-products, inter alia, bis-alkylated glutamic acid.

**Figure 7:**
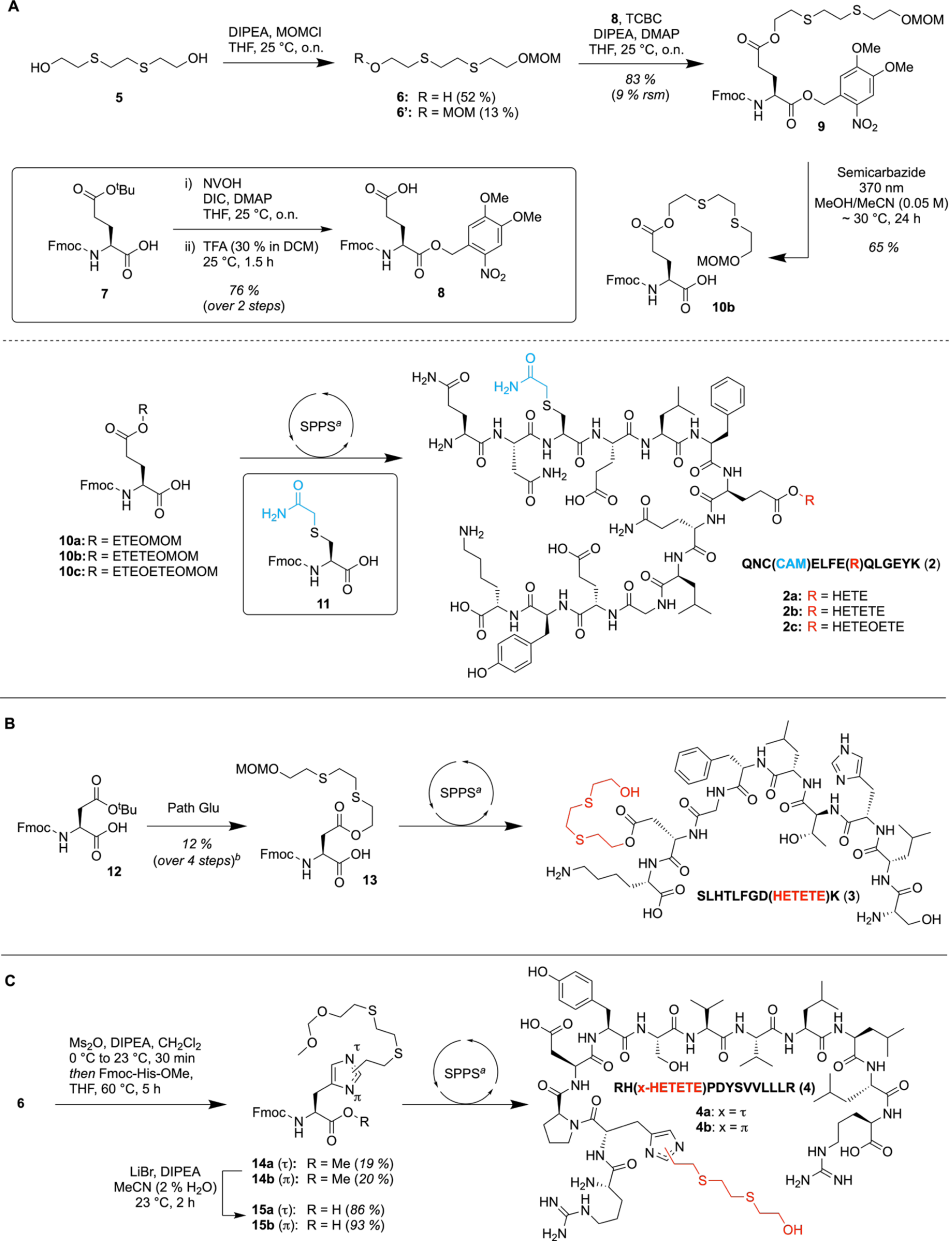
Synthesis of the building blocks and incorporation into different tryptic peptides previously identified. (A) Preparation of glutamic acid adduct starting from diol **5**. The synthesis was demonstrated for the HD, Q, and T analogues, and the resulting building blocks were incorporated into the corresponding peptide **2**. (B) Adaptation of the procedure to aspartic acid was performed for the Q derivative exclusively. (C) Synthesis of a histidine building block starting from compound **6**. The synthesis was only demonstrated for the Q derivative. ^a^Detailed reaction conditions are given in Section 1.3 of the Supporting Information. ^b^Non-optimized conditions.

In the following step, irradiation at 370 nm under an inert atmosphere—to prevent oxidation of the thioether^47^—afforded desired building block **10b** in 28% yield over three steps. The corresponding MOM-protected adducts of HD and T were prepared in comparable yields. The building blocks **10a–c** were then integrated into the previously mentioned peptide sequence **2** via solid-phase peptide synthesis (SPPS) while also using carbamidomethylated cysteine **11**. Cleavage from the 2-chlorotrityl (2CT) resin was performed under mildly acidic conditions to prevent the formation of N-terminal pyroglutamic acid.

Likewise, the synthesis of the aspartic acid building block was achieved following the same procedure as for glutamic acid, starting from diol **4** and protected aspartic acid **12** to afford building block **13** in 12% yield over three steps using non-optimized conditions (Figure 7B). The obtained building block was implemented into peptide sequence **3** as a representative peptide found in the statistical analysis with high confidence.

Further, a synthesis strategy for the SM-modified histidine building block was developed. Several approaches were followed, starting from histidine, *N*-Fmoc protected, as well as *N*-Fmoc- and *O*-Me-protected histidine. Alkylation of Fmoc-His-OMe using the alkyl chloride derivatives of **6** failed mainly due to the limited reactivity of the latter in organic solvents, highlighting potential pure S_N_1 behavior of this reagent. The same observations were made for unprotected histidine using known procedures for selective *N* ^τ^ alkylation of the imidazole.^48^ Using Fmoc-His-OH as the starting material and wet organic solvents leads to full conversion of the alkyl chloride according to HPLC analysis, but only alkylation of the carboxylic acid and hydrolysis of the alkyl chloride forming starting material **6** were observed. We also investigated other approaches, such as alkylation under the support of different Lewis acids, using Mitsunobu conditions, or converting the alcohol of **6** to different alkyl halides, all without success. Moreover, treatment of the mentioned alcohol with mesyl chloride exclusively gave the corresponding alkyl chloride.

To our delight, in situ preparation of the corresponding mesylate using mesyl anhydride, followed by exposure to Fmoc-His-OMe, led to successful formation of the monoalkylated derivatives **14a** and **14b**, which could be separated by column chromatography (Figure 7C). Reduced yield is provoked by quaternization of the imidazole, which could be diminished by performing the reaction under reflux conditions and using an excess of Fmoc-His-OMe. Having the two monoalkylated histidine derivatives in hand, the free carboxylic acid was obtained by treatment with excess LiBr^49^ in wet acetonitrile to afford the desired building blocks **15a** and **15b**. These were further implemented into the peptide sequence RHPDYSVVLLLR (**4**) to afford peptides **4a** and **4b**, respectively. Notably, a comparison of the two synthetically prepared adducts to the identified peptide via nLC-timsTOF analysis suggests that the biomarker present in exposed blood serum is primarily the π-adduct. This indicates either preferred modification of the *N^π^* position or deviating digestion efficiency depending on the modification site of the imidazole (see Figure S14 in the Supporting Information).

### Analytical Verification of the [HETETE]-CPF Tripeptide

To compare our approach to the current gold standard,^25^ i.e., the formation and detection of tripeptide-adducts **1**, we also performed a ProtK digest of blood serum exposed to Q after the R2-P1 protocol. Even at low-level exposure, the tripeptide was identified by measurement in prm-PASEF mode (Figure 8A). However, in the LC, the analyte is accompanied by a satellite peak with the same fragmentation pattern as the main peak, which was also observed for the corresponding synthetically prepared peptide adducts **1a** and **1c** (see Figure S11, Supporting Information). Moreover, the same behavior was also observed in the mobilogram upon direct infusion of the synthetically prepared peptide **1b**, but only after modulating the tims separation to higher resolution in the range of the peptide’s mobility (Figure 8B).

**Figure 8:**
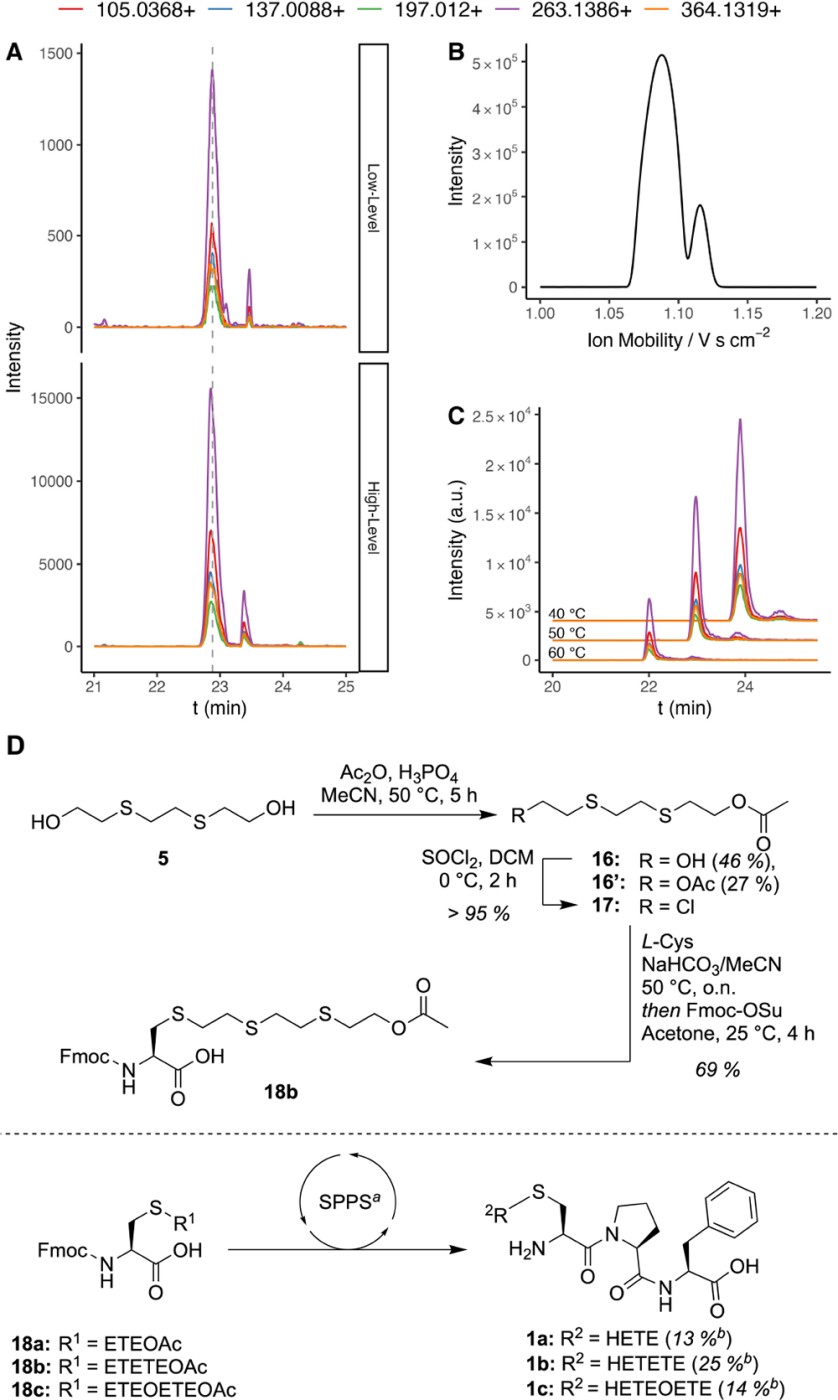
Measurement of the tripeptide [HETETE]-CPF (**1b**) in blood serum at two exposure levels: 5.0 mM (high) and 0.05 mM (low), and enzymatic digest using ProtK. (A) XICs of prm-transitions of the tripeptide (Savitzky–Golay smoothing). (B) Mobilogram of synthetically prepared **1b** reveals two peaks in the mobility dimension upon increasing the sensitivity. (C) XICs of prm-transitions of synthetically produced **1b** standard were evaluated at three different column temperatures. (D) Preparation of Fmoc-protected and *S*-modified cysteine building block at the example of the Q derivative (**18b**) and the implementation of the three adduct analogues into the tripeptide. ^a^Detailed reaction conditions are given in Section 1.3 of the Supporting Information. ^b^Overall yield starting from the corresponding diol.

The satellite peaks probably arise from isomers. Indeed, rotamers could be excluded by the peak area being unaffected by the temperature change of the column, as depicted in Figure 8C. Isomerization of synthetic peptides can stem either from reduced enantiomeric purity of the starting materials or racemization of amino acids, especially cysteine, during SPPS.^50^ However, this process is rare for peptides from natural sources. Moreover, proline can occur as a *cis-* or *trans*-isomer, which influences the three-dimensional structure of the resulting peptide. Indeed, the two isomers were confirmed to be present via NMR spectroscopy as a mixture with a preference for the *trans*-isomer. Although this potentially explains the peak separation, the assumption requires the interconversion rate between the two isomers to be low, i.e., outside the elution time regime of the LC, even at the applied temperature of the column. Hence, further experiments, outside the scope of this work, would be required to infer causality.

Figure 8D presents the synthesis of the cysteine building block with the example of the Q derivative starting from the corresponding diol. Different protecting groups were evaluated, while the acetyl protection (Ac) was found to work best (see Schemes S5 and S6, Supporting Information). Hence, diol **5** was acetyl-protected using acetic anhydride and catalyzed by phosphoric acid^51,52^ to afford **16**, which was subsequently transformed to the alkyl chloride **17** in quantitative yield. Coupling to *L*-Cys and subsequent Fmoc-protection of the amine afforded building block **18b**. The corresponding HD (**18a**) and T (**18c**) adducts were obtained accordingly. Finally, the building blocks were incorporated into the tripeptide via SPPS. While still attached to the solid support, the peptide was treated with 1 M KOH in MeOH and finally released from the resin under mild acidic conditions to afford peptides **1a–c**.

## Conclusions

As demonstrated in the previous paragraphs, our novel strategy allows for the identification of biomarkers for retrospective verification of exposure to sulfur mustards in blood serum using proteomic workflows and statistical analysis. This strategy significantly increases the throughput in biomarker identification as well as the number of samples, making our methodology an excellent high-throughput approach. Our technique not only confirmed previously known alkylation sites but also provided a better understanding of their location within a protein and the biomarker. This constitutes both validation of the workflow and a more accurate determination of a CWA exposure. The current limit of detection of around 5.0 μM final concentration of Q in blood serum is intimately linked to our own instrument sensitivity and could be lowered by appropriate instrumental adjustments. Moreover, implementation of an enrichment strategy based on CibaMaBs is interesting in lowering the matrix complexity in the dda-PASEF mode and when using shorter chromatography gradients. By essence, this strategy is able to identify new biomarkers of different electrophiles in a high-throughput manner. Hence, we propose our workflow not only for the identification of novel biomarkers to any chemical warfare agents via adductomics but also for use when high-throughput sample analysis is necessary.

## Supplementary Material



## Data Availability

Proteomics raw data are available from PRIDE (PXD068389); R codes are available from GitHub (github.com/Chemdrumz/Proteomics-SM); and NMR raw data are available from Zenodo (DOI: 10.5281/zenodo.16810855).

## Notes

Supplementary, experimental and full characterization data of synthetically prepared substrates; and appendix (PDF)
