# Supplementary material for: Application of Proteomic Workflows for the Identification of Biomarkers for the Retrospective Verification of Sulfur Mustard Intoxication
Source: Anal Chem. 2026 Apr 1;98(14):10550–61. doi: 10.1021/acs.analchem.5c07620 (PMC13084624; doi:10.1021/acs.analchem.5c07620)

Application of Proteomic Workflows for the Identification of Biomarkers for the Retrospective Verification of Sulfur Mustard Intoxication

Gianin Thomann,[a,b] Maximilian Brackmann,*[a] Christian G. Bochet[b] and Christophe Curty*[a]

[a]Spiez Laboratory, Federal Office for Civil Protection, 3700 Spiez, Switzerland
[b]Department of Chemistry, University of Fribourg, 1700 Fribourg, Switzerland

Corresponding E-Mail: maximilian.brackmann@babs.admin.ch
christophe.curty@babs.admin.ch

Contents

1 Materials and Methods **S4**
1.1 General Information S4
1.1.1 Chemicals S4
1.1.2 Instrumentation and Materials for the Synthesis of Standards S4
1.1.3 Instrumentation for the Analysis of Synthetic Products S4
1.1.4 Instrumentation for Proteomic Sample Preparation and Analysis S5
1.2 Exposure Experiments of Blood Serum to SM S6
1.2.1 Sample Preparation S6
1.2.2 Manual confirmation of Peptide Alkylation upon Exposure to Q S8
1.2.3 Experiment 1: Influence of Storage and Digestion Time on the Adduct Intensities S9
1.2.4 Experiment 2: Comparison of Individuals and Sexes S10
1.2.5 Experiment 3: Comparison of HD, Q and T S11
1.2.6 Meta-Analysis of Experiments 1 - 3 . . . . . S13
1.2.7 Experiment 4: Target-Analysis of Selected Peptides S17
1.2.8 Experiment 5: Experimental Verification of Tripeptide in Blood Serum Exposed to Q S18
1.3 Synthesis of Standards S21
1.3.1 Solid-Phase Peptide Synthesis (SPPS) S21
1.3.2 Synthesis of SM-Adducts to Glutamic Acid and Aspartic Acid S22
1.3.3 Synthesis of Q-Adduct of Histidine S37
1.3.4 Synthesis of Tripeptide S43

2 Appendix **S58**
2.1 Supplementary Tables S58
2.2 NMR Spectra S82
2.3 MS/MS-Spectra of Synthetically Prepared Peptides S140

List of Figures

S1 Comparison of spectral library building in DIANN against PEAKS. S9
S2 Influence of digestion time and storage time on the analysis. S10
S3 Background level of peptides and influence of sexes on the intensity of adducts. S11
S4 PCA of blood serum exposed to HD, Q or T. S12
S5 Influence of HSA-peptides on the clustering of the data. S13
S6 Influence of batch correction on the data using empirical Bayes. S14
S7 Presence of HETE-adducts characteristic for HD exposure in blood serum treated to Q. S15
S8 PCA using filtered data. S16
S9 Visualization of modification sites on HSA S16
S10 prm-PASEF of exposed blood serum digested with ProtK for the verification of the tripeptide. . . . S19
S11 prm-PASEF of synthesized Tripeptides. S19
S12 2D-NMR of synthesized Tripeptide. S20
S13 Set-up irradiation for Nvoc removal. S24
S14 Retention time comparison of pro- and tele-adducts to histidine S42

List of Schemes

S1 Fragmentation of HETETE adducts. S8
S2 Hydrolysis pathways of Q. S14
S3 Preparation of glutamic acid building block. S22
S4 Synthesis approach for the preparation of the tripeptide [HETETE]-CPF. S43
S5 Failed synthesis attempts for tripeptides. S52

List of Tables

S1 Ionisation Settings for timsTOF measurement. Captive Spray Source was primarily applied for proteomic analysis while the Apollo II source were used for direct infusion experiments. S5
S2 General Settings for timsTOF measurement. S5
S3 PTMs considered for the evaluation of proteomic data sets. S6
S4 Charging of the APEX well plates for R2-P1 protocol. S7
S5 Settings for timsTOF measurement in low-mass dda-PASEF mode. S8
S6 Batches to investigate the influence of storage and digestion time on the adduct Intensities. S9
S7 timsTOF-settings for the measurement of the tripeptide in prm-PASEF mode. For not-listed parameters the default settings as given in Table S7 were used. S18
S8 Optimization of the esterification of Fmoc and Nvoc protected glutamic acid. S23
S9 Optimization of Cys alkylation and Fmoc protection. S44
S10 Manually confirmed peptide adduct identified via de novo sequencing. S59
S11 Identified peptide adducts with importance in random forest as well as contribution to PCA and their HD and T analogs. S60
S12 Targetlist for prm-PASEF measurement using a short elution gradient S79
S13 Targetlist for prm-PASEF measurement using a long elution gradient S80

1 Materials and Methods

1.1 General Information

1.1.1 Chemicals

Commercially available chemical reagents were bought from Fisher Scientific, Merck, Sigma-Aldrich and Iris Biotech and were used without further purification. Starting Material 3,9-dithia-6-oxa-undecane-1,11-diol as well as chemical warfare agents (CWAs) – namely Mustard Gas (HD), Sesquimustard (Q) and O-Mustard (T) – were provided by the Spiez Laboratory. *Note, that the sulfur mustard derivatives as CWAs are highly toxic and should only be used by trained personal and under suitable safety measurements.* Anhydrous solvents were used exclusively for synthesis and dried using molecular sieves or obtained commercially. Solvents for column chromatography, e.g. CH_2Cl_2, EtOAc, n-Hex, c-Hex, Et_2O, CH_3CN and MeOH (all LiChroSolv) were used without drying and are obtained from Merck as well as H_2O (HPLC Grade) and CH_3CN (HPLC Grade) were purchased from J. T. Baker. All resins for solid-phase peptide synthesis (SPPS) were obtained from Iris Biotech.
Trypsin/Lys C Mass spectrometry grade was obtained from Promega. Proteinase K and Human Serum Albumin were obtained from Merck. Carboxylated beads were obtained from Cytiva and MagReSyn hydroxyl beads from ReSyn Biosciences.
Human blood serum was provided by healthy individuals with their consent. Note, that according to the *Federal Office of Public Health (FOPH) - Human research: approval of research projects* no authorisation is required for projects with anonymized and non-genetic data[1].

1.1.2 Instrumentation and Materials for the Synthesis of Standards

Reactions under inert atmosphere were conducted in heat-gun or oven-dried glassware attached to a Schlenk-line. Solutions were concentrated under reduced pressure by using a rotary evaporator. Reactions carried out at 0 °C were cooled using an ice bath. Reactions at −78 °C were cooled with dry ice in acetone.
Flash column chromatography was performed on an *Isolera One* from Biotage. Consumables were bought from Biotage including cartridges like: Sfar HC Duo 10 g / 25 g / 50 g / 100 g and Sfar C-18 Duo 12 g / 30 g / 60 g. For all dry loadings for normal phase chromatography KP-SIL silica 40 µm – 63 µm from Biotage was used. For all reversed phase chromatography dry loadings KP-C18-HS silica was used.
SPPS was carried out on a *Biotage®* Initiator+ Alstra™ Automated Microwave Peptide Synthesizer. Consumables for SPPS were all coming from Biotage.
Centrifugation of synthesized compounds was performed in an *Eppendorf Centrifuge 5810 R* at 4000 rpm at ambient temperature for 5 min (Centrifuge tubes obtained from VWR).
Lyophilisation was carried out overnight on an *Alpha 1-2 LD plus Lyophilisator* from Christ.

1.1.3 Instrumentation for the Analysis of Synthetic Products

NMR analysis was carried out at room temperature on a *Bruker Avance III HD 400 MHz Nano Bay* with an operation frequency of 400 MHz for ^{1}H-NMR, 101 MHz for ^{13}C-NMR. The solvents used were $CDCl_3$, CD_3OD, CD_3CN, D_2O, DMSO-d^6 or mixtures thereof. NMR spectra are reported downfield from tetramethylsilane (TMS) (δ = 0.00) added to the corresponding solvent system. Chemical shifts (labelled as δ) are reported in ppm. Coupling-constants (J) are reported in Hz. Multiplicities are declared as s (singlet), d (doublet), t (triplet), q (quadruplet), quint (quintet), sext (sextet), sept (septet) and m (multiplet) or combinations thereof.
Liquid-chromatography, electron spray ionization, high resolution time of flight (LC/ESI-TOF) was performed on an *Agilent Technologies 1290 Infinity II LC* coupled to a *Bruker Daltonics maXis II UHR QTof HRMS* equipped with a Sigma–Aldrich Discovery HS C18 (150 mm × 2.1 mm, particle size 5 µm). H_2O + 10 % MeOH with 5 mM NH_4Ac and MeOH with 5 mM NH_4Ac were used as eluents with a flow rate of 0.6 mL/min.
(Preparatory) High Performance Liquid Chromatography with diode array detection and electron spray ionization single quadrupole mass spectrometry ((Prep)HPLC-DAD/ESI-Q) in sequence, was performed on an *Agilent 1260 Infinity II LC/MS system* equipped with a ZORBAX SB-C18 (150 mm 4.6 mm, particle size 5 µm) for analysis and an Agilent 5 Prep-C18 (150 mm 21.2 mm, particle size 5 µm) for preparatory applications. H_2O with 0.1 % FoA

and MeCN with 0.1 % FoA were used as eluent. Peptide purity was evaluated at 215 nm wavelength.
Gas-chromatography mass spectrometry with electron impact ionization (GC-MS (ESI)) was performed on an *Agilent Technologies 7890A* coupled to a single quadrupole. A *Bruker Tensor II* equipped with a *Golden Gate diamond ATR system* was used to record infrared spectra. All melting points were measured on a *Büchi 535* or *MettlerToledo MP80*.

1.1.4 Instrumentation for Proteomic Sample Preparation and Analysis

Incubation of biological samples was performed on a *ThermoMixer® C* with heated lid and 0.5 mL, 1.5 mL, 2.0 mL or PCR 96 SmartBlocks from Eppendorf.
Automated R2-P1 protocol was performed on a *KingFisher Apex System* from ThermoFisher Scientific.
Fluorescence measurement for the determination of the tryptophan/protein content was performed on a *ClarioStar* from BMG Labtech with an 280 nm excitation filter and an 360 nm emission filter or on a *Infinite® F Nano+* microplate reader from Tecan with an 280 nm excitation filter and an 360 nm emission filter. The proteomic samples were submitted to the system in QuanRecovery with MaxPeak HPS vials.
Nano Liquid-chromatography, electron spray ionization, trapped ion mobility spectrometry, quadrupole time of flight mass spectrometry (nLC-timsTOF) was performed on a *Bruker nanoElute* coupled to a *Bruker timsTOF Pro II*. The column used was an IonOpticks Aurora Elite (150 mm × 0.075 mm, 1.7 µm particle size) at 50 °C with the following eluents: (A) H_2O + 0.1 % FoA and (B) CH_3CN + 0.1 % FoA with 1 µL injection volume, if not elsewise stated. An elution program starting with a linear gradient from 2 % B to 35 % B in 25 min and then from 35 % to 95 % in 0.5 min was performed. This composition was held for 5.5 min. The following capillary settings of the source were used:

Table S1 Ionisation Settings for timsTOF measurement. Captive Spray Source was primarily applied for proteomic analysis while the Apollo II source were used for direct infusion experiments.

	Captive Spray	Apollo II
Capillary [V]	1500	3500
Dry Gas [L min^{-1}]	3.0	3.0
Dry Temperature [°C]	180	180

Settings of the timsTOF are presented in the following table:

Table S2 General Settings for timsTOF measurement. These may deviate between experiments. For run specific settings, please consult the raw data files (PRIDE, Project-Key: PXD068389)

1/k0 start	0.70 V s cm^{-2}
1/k0 end	1.50 V s cm^{-2}
Cycle Ramp Time	100 ms
Scan Range MS1	100 m/z – 1700 m/z
Funnel 1 RF	475.0 Vpp
isCID Energy	0.0 eV
Funnel 2 RF	200.0 Vpp
Multipole RF	200.0 Vpp
Collision RF	1500.0 Vpp
Collision Energy	10.0 eV
Transfer Time	60 µs
Pre-pulse Storage Time	12 µs
Dt1 (Defl. Transfer → Capillary Exit)	−20.0 V
Dt2 (Defl. Discard → Defl. Transfer)	−160.0 V
Dt1 (Funnel 1 In → Defl. Transfer)	110.0 V
Dt1 (Accu. Trap → Funnel 1 In)	110.0 V
Dt1 (Accu. Exit → Accu. Transfer)	0.0 V
Dt1 (Ramp Start → Accu. Exit)	55.0 V
Collision Cell in	300.0 V

Calibration of the TIMS cell was performed on the following masses: 622.0290 m/z (hexakis(2,2-difluoroethoxy)-phosphazene, 186817-57-2, Apollo Scientific), 922.0098 m/z (hexakis(2,2,3,3-tetrafluoropropoxy)phosphazene,

58943-98-9, Apollo Scientific) and 1221.9906 m/z (Chip Cube High Mass Reference, G1982-85001, Agilent Technologies). Gas flow in the cell was adjusted so that the voltage of the 622.0290 m/z peak is set to $\sim$ 132 V. Calibration of the TOF is performed using sodium formate (NaFa) via CaptiveSpray or ESI-L Tuning Mix (G1969-85000) from Agilent Technologies via Apollo II Source.
Instrument performance was monitored using Pierce™ HeLa Protein digest standard (100 ng µL^{-1}) with 0.5 equiv indexed Retention Time (iRT) standard provided by Biognosys and the data evaluated with *QuiC™ Professional+* (Peregrine Falcon, 64-bit) from Biognosys.

1.1.4.1 Data Analysis

Data evaluation of proteomics data-dependent acquisition (dda) measurements was performed using PEAKS Studio® (10.6 build 2020 1221, Bioinformatics Solutions Inc)[2,3] using an error tolerance of 15 ppm on precursor mass and 0.05 Da for the fragment ion mass. The digest mode was set to specific with maximum missed cleavages per peptide of 2. Human proteome (UP000005640) with BSA as an additional entry was used as the database. Different post-translational modifications (PTMs) were considered in the analysis:

Table S3 PTMs considered for the evaluation of proteomic data sets. ^{a}Modification site. ^{b}Mass difference compared to native peptide.

Name	UniMod-No	Mod-Site^a	Δm/z^b	Classification
Carbamidomethylation	UniMod:4	Cys	+57.0215	Variable
Oxidation	UniMod:35	Met	+15.9949	fixed
Deamidation	UniMod:7	Asn/Gln	+0.9840	fixed
Acetylation (N-term)	UniMod:1	Anywhere	+42.0106	fixed
HETE	-	Cys/Glu/Asp/His	+104.0296	Variable
HETETE	-	Cys/Glu/Asp/His	+164.0330	Variable
HETEOETE	-	Cys/Glu/Asp/His	+208.0592	Variable

Data-independent acquisition (dia) measurements were evaluated with DIANN[4] (Version 1.9+) using the spectral library exported from PEAKS and converted to the needed format for DIANN via an in-house python script. MS-data from reference materials were extracted using Compass DataAnalysis© (Version 6.0 or newer, 64-bit) from Bruker Daltonics and processed using R[5] (4.3.1 GUI 1.79 Big Sur ARM build and newer). prm-PASEF data were evaluated with Skyline (24.1.0.199, 64-bit)[6]. Manual data treatment was carried out with R using the following packages: tidyverse[7], qvalue[8], randomForest[9], pheatmap[10], ggplot2[11], ggpubr[12], ggfortify[13], ggVennDiagram[14], scales[15], RColorBrewer[16] and volcano3d[17].
Protein visualization is performed using ChimeraX-1.11[18].

1.2 Exposure Experiments of Blood Serum to SM

1.2.1 Sample Preparation

General Procedure for the Incubation of Blood Serum with Sulfur Mustard (GPI)
Blood Serum was voluntarily provided by seven healthy individuals, four male, three female. For this, full blood was sampled by trained personal in a serum separation tube and centrifuged (23 °C, 2000 g, 20 min). The supernatant was transferred into a sterile falcon tube and stored at −23 °C until further use. The samples were treated anonymously and randomly. Only the sex of the individual the corresponding serum sample originates from was disclosed for the presented studies. The corresponding solutions of Sulfur Mustard (HD, Q or T) in CH_3CN (or plain CH_3CN for non-exposed serum samples) was added to human blood serum (990 µL) in exposure levels (final concentration of SM) of 5.0 mM (high-) and 50 µM (low), and the samples incubated at 37 °C for 24 h. After termination of incubation, the samples were stored at −23 °C until further use. For all experiments, quality control samples were prepared as equal part mixtures of the corresponding non-exposed serum samples in each experiment. The resulting samples were subjected to sample preparation along with exposed and non-exposed serum samples.

General Procedure for Proteomic Sample Preparation (GPPSP)
Bottom-up proteomics samples were prepared following an automated protocol adapted from Hughes et al.[19] and Leutert et al.[20]. In brief, blood serum (2.5 µL) was diluted with phosphate buffered saline (PBS, pH 7.4, 97.5 µL) and treated with dithiothreitol (DTT, 100 mM, 5 µL) at 60 °C for 30 min, iodacetamide (IAA, 200 mM,

10 µL) at room temperature and in the absence of light for 30 min and finally quenched with DTT (100 mM, 5 µL) at room temperature for additional 15 min. The resulting mixture was stored at −23 °C until further use. Carboxylated bead solution was prepared according to the named publication: In an Eppendorf tube, bead stock solution (Cytiva 1 µm avg. particle size - 5 % suspension, Cytiva 0.70 µm – 1.10 µm particle size - 5 % suspension; 1/1, v/v; 2 mL) was incubated on a magnetic rack and the supernatant removed. The beads were washed with HPLC grade H_2O (4 × 1 mL) and then resuspended in HPLC grade H_2O (1.9 mL) to give a 50 µg μL^{-1} suspension which was stored at 4 °C until further use. Rapid-robotic proteomics (R2-P1) protocol was performed in accordance to literature[20] using APEX 96 well plates. The corresponding wells on the plates are charged with solutions according to the following table:

Table S4 Charging of the APEX well plates for R2-P1 protocol. [a]Carboxylated beads in HPLC grade H_2O (50 mg mL^{-1}). [b]Trypsin/Lys-C (5 µg mL^{-1}) or ProtK (100 µg mL^{-1}) in 50 mM NH_4HCO_3 buffer.

Beads plate:	Bead solution[a] (5 µL), HPLC grade H_2O (45 µL)
Binding plate:	Corresponding protein sample (approx. 1 µg μL^{-1}, 25 µL) and EtOH (100 %, 25 µL)
Wash plates 1-3:	EtOH (80 %, 50 µL)
Elution plate:	Protease in buffer[b] (100 µL)

Protein content of the samples was checked by fluorescence measurements of the binding plates at 25 °C prior to the R2-P1 protocol but after centrifugation (500*g*, 1 min). As calibration standards, *L*-tryptophan (Sigma-Aldrich) in EtOH/HPLC grade H_2O (1/1, v/v; 50 µL) in the following concentrations was used: 2.0 µg mL, 4.0 µg mL, 5.0 µg mL, 7.0 µg mL, 8.0 µg mL, 10.0 µg mL. The time protocol of R2-P1 was executed as followed:

1. Pick up beads from beads plate
2. Elute beads into binding plate and incubate for 12 min
3. Transfer beads to Wash 1 and incubate for 2 min
4. Transfer beads to Wash 2 and incubate for 2 min
5. Transfer beads to Wash 3 and incubate for 2 min
6. Transfer beads into elution plate
7. Transfer elution plate for external digest.
8. Remove beads

For protease digestion, the elution plate was covered with an adhesive film and incubated in a ThermoMixer at the temperature and time indicated for each experiment. After removal of the beads, the samples were transferred into an Eppendorf tube, centrifuged (10000*g*, 5 min) and an aliquot of the supernatant transferred into a vial for measurement via nLC-timsTOF. The intensities of the identified peptides were summed over all charge states within a single run and then $\log_2$-transformed in the data analysis.

1.2.2 Manual confirmation of Peptide Alkylation upon Exposure to Q

Blood serum from one healthy individual was exposed to Q as described in **GPI** at an exposure level of 5.0 mM in biological triplicates. Proteomic sample preparation was performed according to **GPPSP**. Digestion was performed at 37 °C for 18 h using trypsin/Lys-C. The resulting samples were first analyzed in dda-PASEF mode and evaluated with PEAKS and then via low-mass dda-PASEF, meaning the MS parameters were adjusted as follows:

Table S5 Settings for timsTOF measurement in low-mass dda-PASEF mode. For not-listed parameters the default settings as given in Table S7 were used.

1/k0 start	0.60 V s cm^{-2}
1/k0 end	1.60 V s cm^{-2}
Cycle Ramp Time	100 ms
Scan Range MS1	100 m/z – 1700 m/z
Funnel 1 RF	450.0 Vpp
isCID Energy	0.0 eV
Funnel 2 RF	250.0 Vpp
Multipole RF	250.0 Vpp
Collision RF	500.0 Vpp
Collision Energy	10.0 eV
Transfer Time	50 µs
Pre-pulse Storage Time	8.0 µs
Dt1 (Defl. Transfer → Capillary Exit)	−20.0 V
Dt2 (Defl. Discard → Defl. Transfer)	−160.0 V
Dt1 (Funnel 1 In → Defl. Transfer)	110.0 V
Dt1 (Accu. Trap → Funnel 1 In)	110.0 V
Dt1 (Accu. Exit → Accu. Transfer)	0.0 V
Dt1 (Ramp Start → Accu. Exit)	55.0 V
Collision Cell in	300.0 V

This allows to also investigate the lower masses in MS2, in specific the masses 105.04 m/z, 137.01 m/z and 165.04 m/z in MS2 (see Scheme S1). These transitions (qualifiers) of the HETETE side-chain were used to confirm identifications made by de novo sequencing in PEAKS. For this, peptides found in PEAKS are identified in the low-mass dda-PASEF runs to confirm the presence of the named fragment ions. Following this approach, 26 peptides were identified successfully (Table S10). To reduce the number of peptides for manual confirmation, only peptides which full-fill the following caveats are considered at this stage:

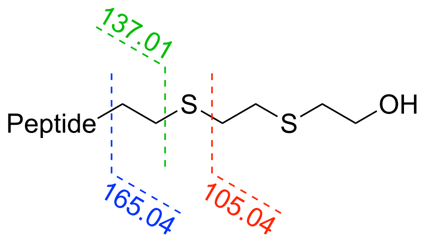

Scheme S1 Fragmentation of HETETE adducts.

1. $-10 \log P > 40$
2. Only present in treated samples
3. Good signal-to-noise of the fragments in MS2 (subjective)
4. Length: Should be feasible for synthesis, i.e. max 15 AA.

A full list of the identified peptides is given in Table S10. However, this approach would be very elaborative to do so for every peptide found in PEAKS. But the given selection demonstrates, that the findings by de novo sequencing appear to be very sound and therefore the statistic significance of the identification is reliable for future experiments. Moreover, the samples were also measured in dia-PASEF mode followed by spectral library building and data evaluation using DIANN. However, it has been observed that the algorithm for retention time and ion mobility prediction within DIANN is not applicable for the presented customized PTMs. For this, three different spectral library techniques were chased: (i) a focused DIANN search where every modification site A, E, H or C were searched one at a time in DIANN (i.e. only one variable modification at a time) and the results were combined, (ii) all modification sites were searched together in a rather open mode (four variable modifications possible), and (iii) DIANN evaluation was performed using spectral library exported from PEAKS. Subfigure Figure S1 A shows quite good matching between all three approaches, however there is a rather large

number of peptides which are found by DIANN but not in PEAKS which probably originates from the higher sensitivity in dia-PASEF (many of these peptides are quite low abundant). A similar picture is drawn when the whole peptide space is reduced to HETETE-alkylated peptides in Figure S1 B. There are some adducts which are not found with PEAKS. Nevertheless, since most of high abundant peptides found with PEAKS (especially the ones which are identified via low-mass dda-PASEF) are not found with DIANN let assume that most of these identified peptides using the latter program are potential mismatches and are omitted. As a consequence, it was decided to follow general workflows (the gold standard in proteomics) by generating the spectral library in dda-PASEF mode with PEAKS and the samples further measured in dia-PASEF mode to increase sensitivity and subsequent DIANN evaluation.

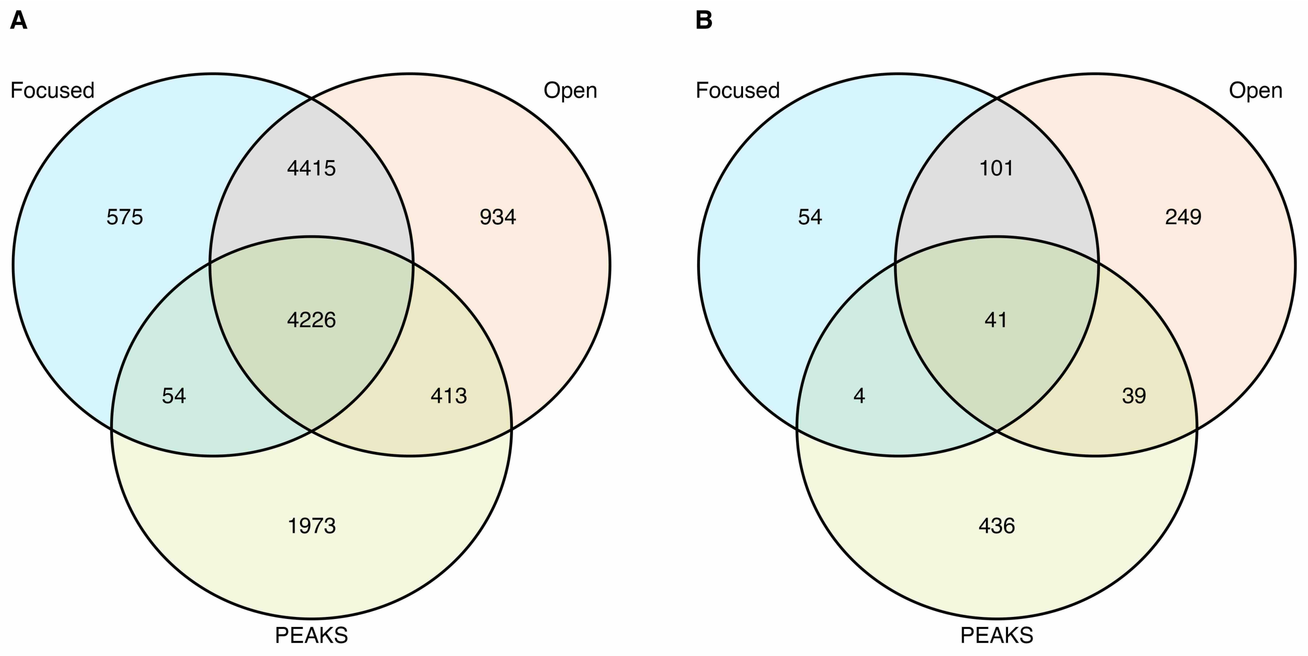

Figure S1 Comparison of spectral library building in DIANN against PEAKS when using alkylation of E, D, H and C as variable PTMs. **A** the whole peptide space, meaning generic and alkylated peptides, is considered. **B** Data filtered for HETETE-alkylated peptides. *Focused: One variable PTM at a time. Open: All variable PTMs are considered. PEAKS: Spectral Library generated from dda-PASEF runs in PEAKS was used.*

1.2.3 Experiment 1: Influence of Storage and Digestion Time on the Adduct Intensities

Table S6 Batches of the experiment: Blood serum from one individual sampled on two different dates was used. The serum was exposed in triplicates at a 5 mM exposure level, non-exposed serum single analysis for each sample batch.

Sample Batch	Time between Sampling and Exposure	Time between Exposure and Measurement
1	1 d	ca. 6 months
2	ca. 6 months	1 d
3	1 d	1 d

Blood serum from one healthy individual was exposed to Q as described in **GPI** at an exposure level of 5.0 mM. Proteomic sample preparation was performed according to **GPPSP**. Digestion time was either 4 h (short digest) or 18 h (long digest) at 37 °C using trypsin/Lys-C in both cases. The resulting samples were analyzed in dda-PASEF mode to build spectral library with PEAKS and then via dia-PASEF followed by data evaluation with DIANN. The results are presented below:

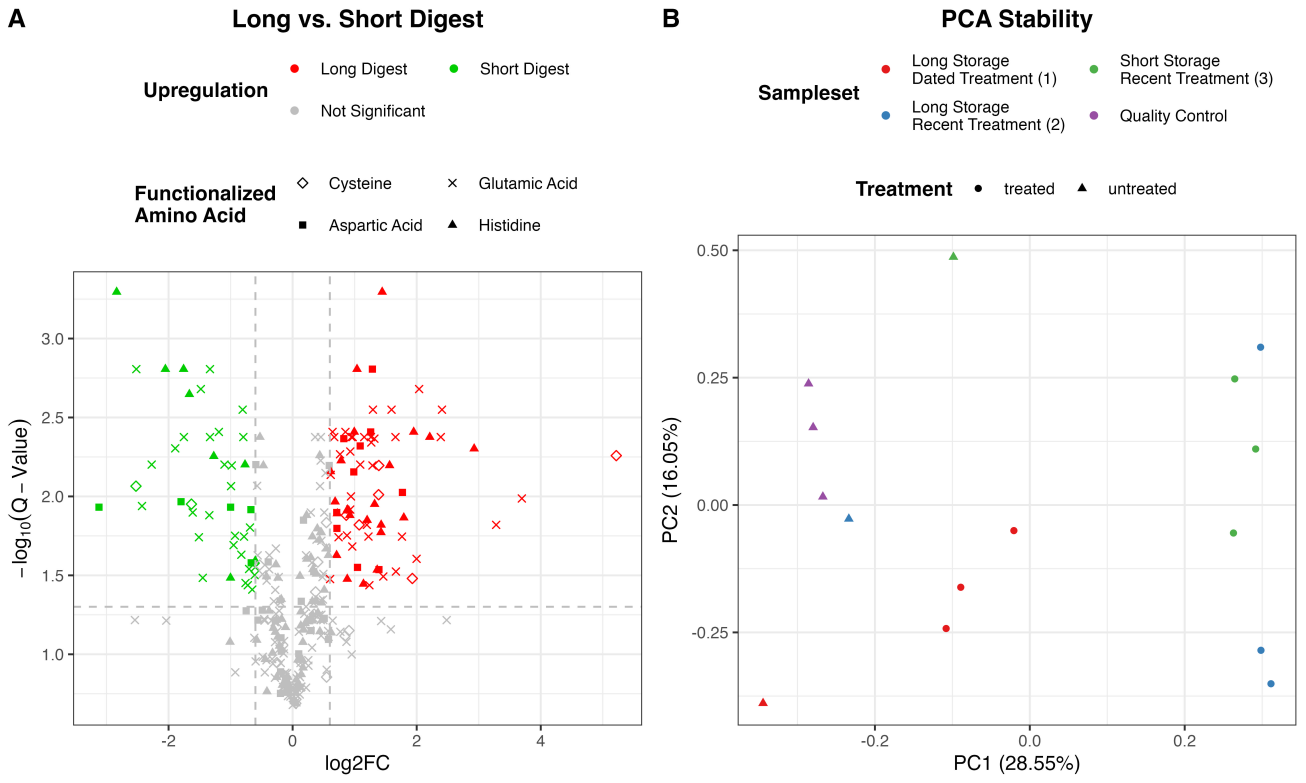

Figure S2 Evaluation of blood serum originating from one individual and three different batches. **A** Influence of digestion time on the intensity of the peptides using only the samples of batch 3 (Table S6). Especially glutamic acid adducts seem to be significantly more expressed in short digested samples. This probably originates from the potential of saponification (hydrolysis) of the ester bond between the amino acid and the alkyl chain. Also, it is important to note that some of the peptides are just increased due to higher probability of missed cleavages when reducing the digest time. **B** PCA of the three batches to reveal influence of the storage time of exposed blood serum at a temperature of $-23\,^{\circ}$C on the clustering of the samples. The evaluation hints partial degradation of the adducts leading to assimilation of exposed samples to non-exposed serum samples upon increased storage time using unsupervised models.

1.2.4 Experiment 2: Comparison of Individuals and Sexes

Blood serum from six healthy individuals was exposed to Q as described in **GPI**. Proteomic sample preparation and digestion was performed according to **GPPSP** at 37 °C for 4 h using trypsin/Lys-C. The resulting samples were analyzed in dia-PASEF mode using the spectral library generated under section 1.2.3 followed by data evaluation using DIANN. The results are presented below:

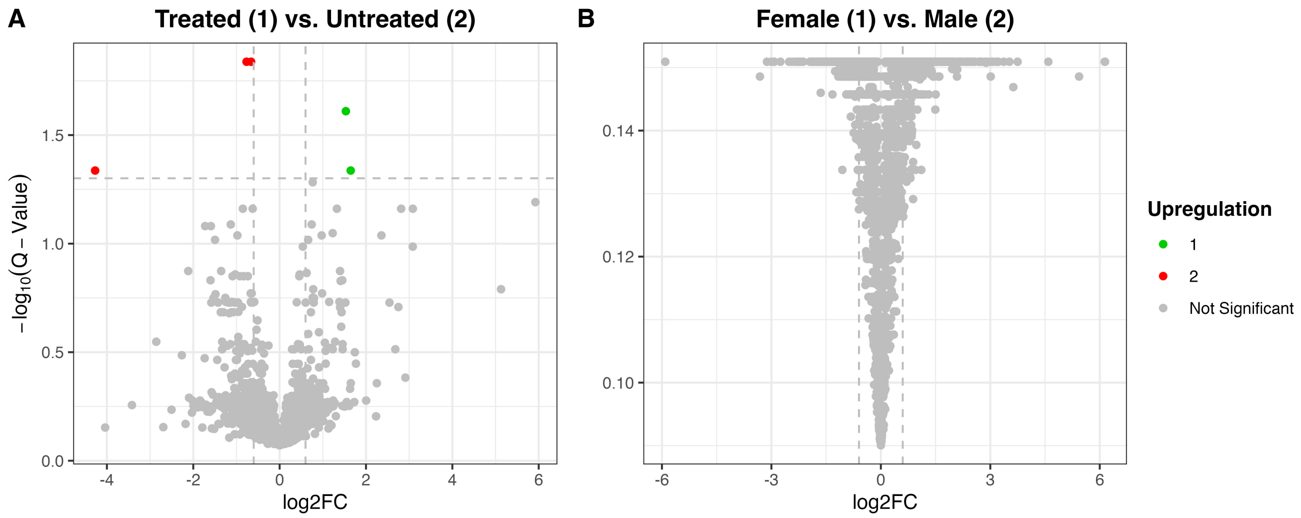

Figure S3 Volcano plots for the comparison of two classes using only non-exposed serum samples and samples exposed at a concentration of 5.0 mM of Q. The numbers of the legend are in accordance to the numbers in the corresponding plot title. **A** Comparison of the peptide background levels, meaning only considering non-alkylated peptides with a caveat for the peptides to be present in all six samples, i.e. individuals, of the corresponding treatment class. The evaluation show only a small number of peptides seem to be upregulated in one or the other class. Hence, the peptide intensities of non-alkylated peptides is not significantly affected by the treatment. **B** Comparing the results between sexes reveals no significant regulation of any of the peptides, neither alkylated nor non-alkylated. Hence, no influence of sex can be expected on the peptide intensity (concentration).

1.2.5 Experiment 3: Comparison of HD, Q and T

Blood serum from three healthy individuals was exposed to HD, Q and T, respectively, as described in **GPI** at an exposure level of 5.0 mM. Proteomic sample preparation and digestion was performed according to **GPPSP** at 37 °C for 4 h using trypsin/Lys-C. The resulting samples were analyzed in dda-PASEF mode to build spectral library with PEAKS and then via dia-PASEF followed by data evaluation with DIANN. For hierarchical clustering in Figure 6 of the manuscript, Figure S4 A and Figure S14, the alkyl modification, i.e. HETE, HETETE and HETEOETE were anonymised as * to allow direct comparison of the corresponding peptides considering only intensities. Additionally, for this purpose only correct alkylations were included in the evaluation, meaning specifically, peptides with HETE alkylation in samples not exposed to HD were considered as false discoveries here. The same was true for any other wrong matching between alkylation type and used agent. This prevented the occurrence of duplicates in the data. Identified peptides are listed in Table S11.

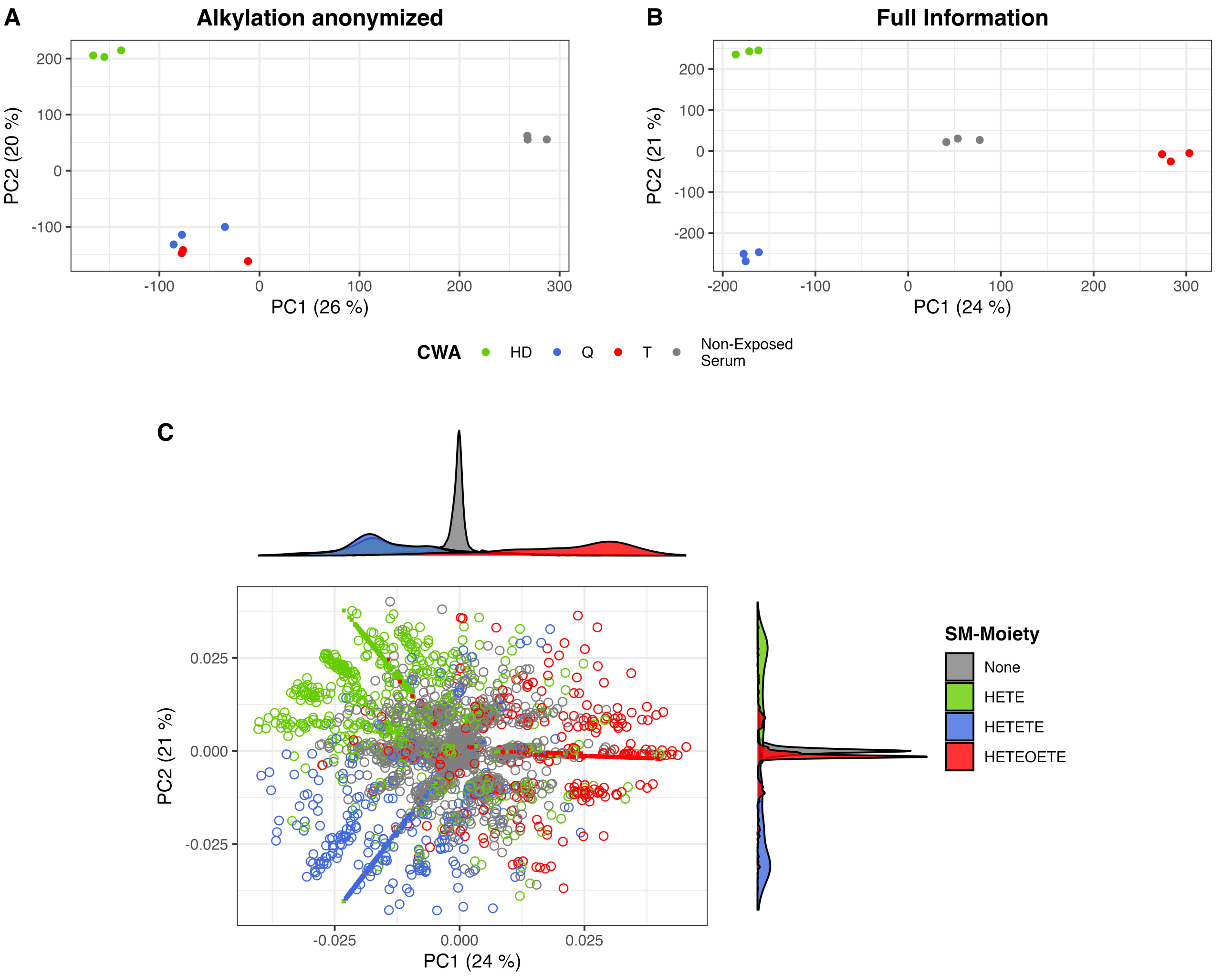

Figure S4 Principal component analysis (PCA) and corresponding loading plot of blood serum exposed to HD, Q or T. **A** PCA by treating the alkylation type (HETE, HETETE, and HETEOETE, respectively) anonymously, leading to clustering of samples exposed to Q and T together. **B** PCA using full data information, i.e. the information about type of alkylation was retained. This means also, that HETE-Adducts in samples exposed to Q are not treated as false discoveries in this case. Now the samples treated equally are clustered together. **C** Loadings of the latter PCA with the density plots in PC1 and PC2 dimension. The formation of the straight lines originate from peptide adducts which are unique for the corresponding CWA.

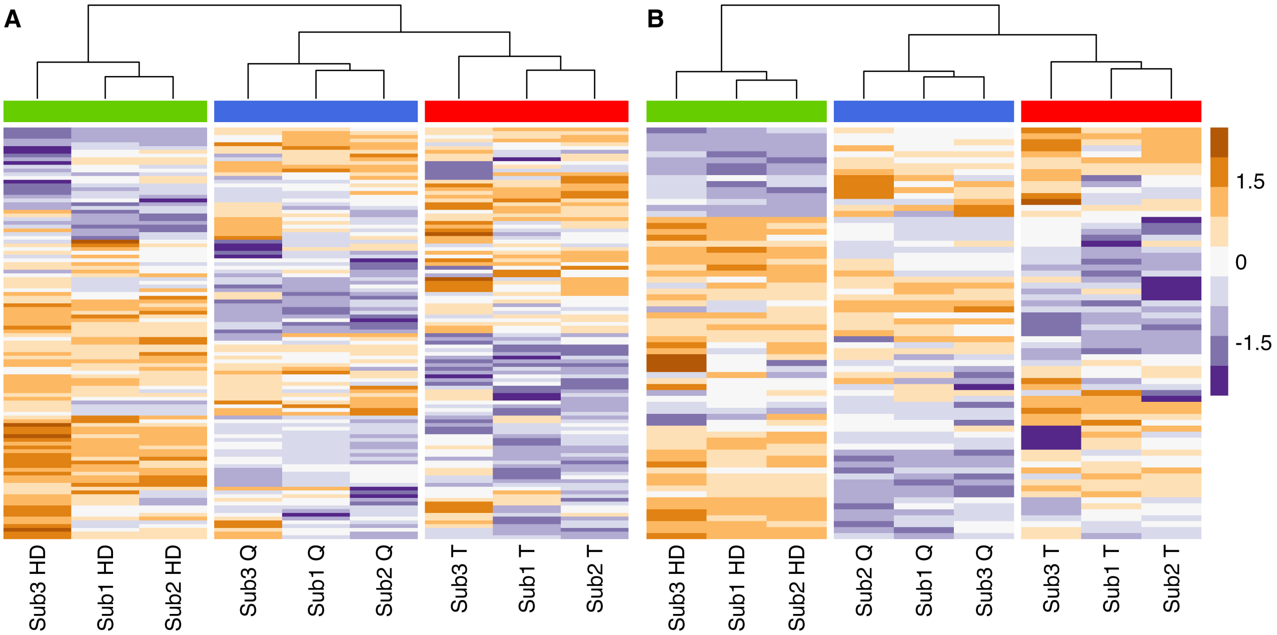

Figure S5 Heatmap with hierarchical clustering (dendrogram) comparing the exposure of blood serum based on the applied SM (green: HD; blue: Q; red: T) using $\log_2$-transformed and *z*-score-normalized data (clustering with Ward's method). **A** Considering only peptides not originating from HSA leads to clustering with similar pattern as when using the full data space. The same is true for **B**, where only peptides belonging to HSA were used for the clustering.

1.2.6 Meta-Analysis of Experiments 1 - 3

To increase statistical strength, the results of Experiments 1 to 3 were used for a broader data analysis, specifically PCA and Random Forest (see Figure 3 in manuscript). For this, all runs of these experiements were combined. However, samples which were long digested, long stored after exposure (in Experiment 1) or samples treated with HD or T (in Experiment 3) were omitted. The resulting data was log_2-transformed and batch corrected using ComBat[21] algorithm. Furthermore, PCA and random forest gave 270 peptides with Loadings(PC1) $<$ 0.01 and non-zero mean decrease Gini as well as using 5-fold cross validation (see Venn diagram in Figure 3 of manuscript). These peptides are listed in Table S11 in the Appendix.

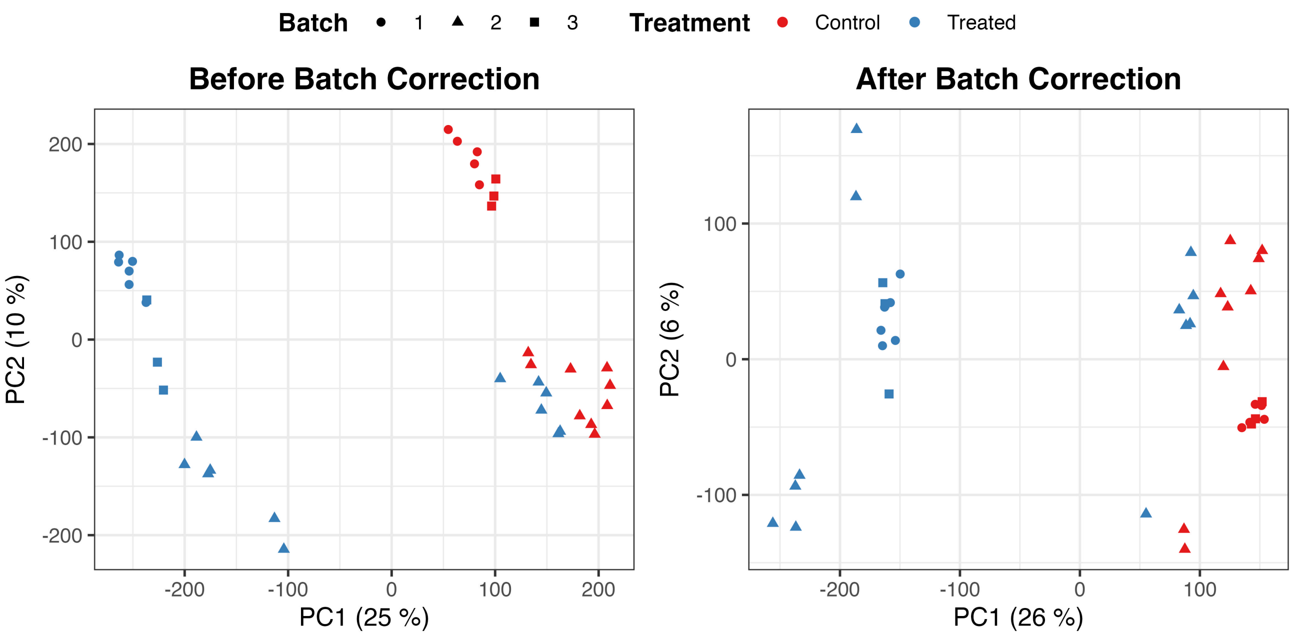

Figure S6 PCA of $\log_2$-transformed data before and after batch correction using ComBat algorithm (empirical Bayes). As depicted here, simple log-transformation already leads to separation of high level exposed samples to other data, however, with some batch effects present in PC2. After batch correction, the batch influence only leads to some residual, minor subclustering of the data. Treated samples clustered with non-exposed serum samples belong to exposure levels of 50 µM and 0.5 µM Q and, hence, are indistinguishable from non-exposed serum samples in this model.

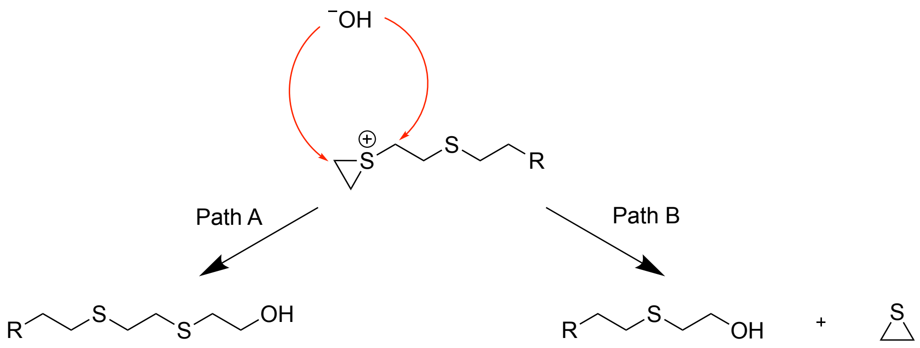

Scheme S2 Hydrolysis pathway of Q as demonstrated also by previous works to be present in decontamination pathways [22]. In this context, the R group may refer to Cl for an intact agent, OH in the case of a semi-hydrolized precursor, or even a biomolecule, such as an amino acid from a protein or metabolite. Considering a S_N2 pathway, the hydrolysis is initiated by the formation of the corresponding thiiranium derivative. Afterwards, there are two possible sites for the hydroxide to attack: (A) The nucleophile attacks one of the carbons of the thiiranium ring leading to formation of a sesquimustard derivative, or (B) the nucleophile attacks on the α-carbon of the propagating alkyl chain, leading to displacement of thiiran by the hydroxide to form an alcohol. The resulting product exhibits a structural similarity to HD rather than Q, thus, deviating from the expected outcome.

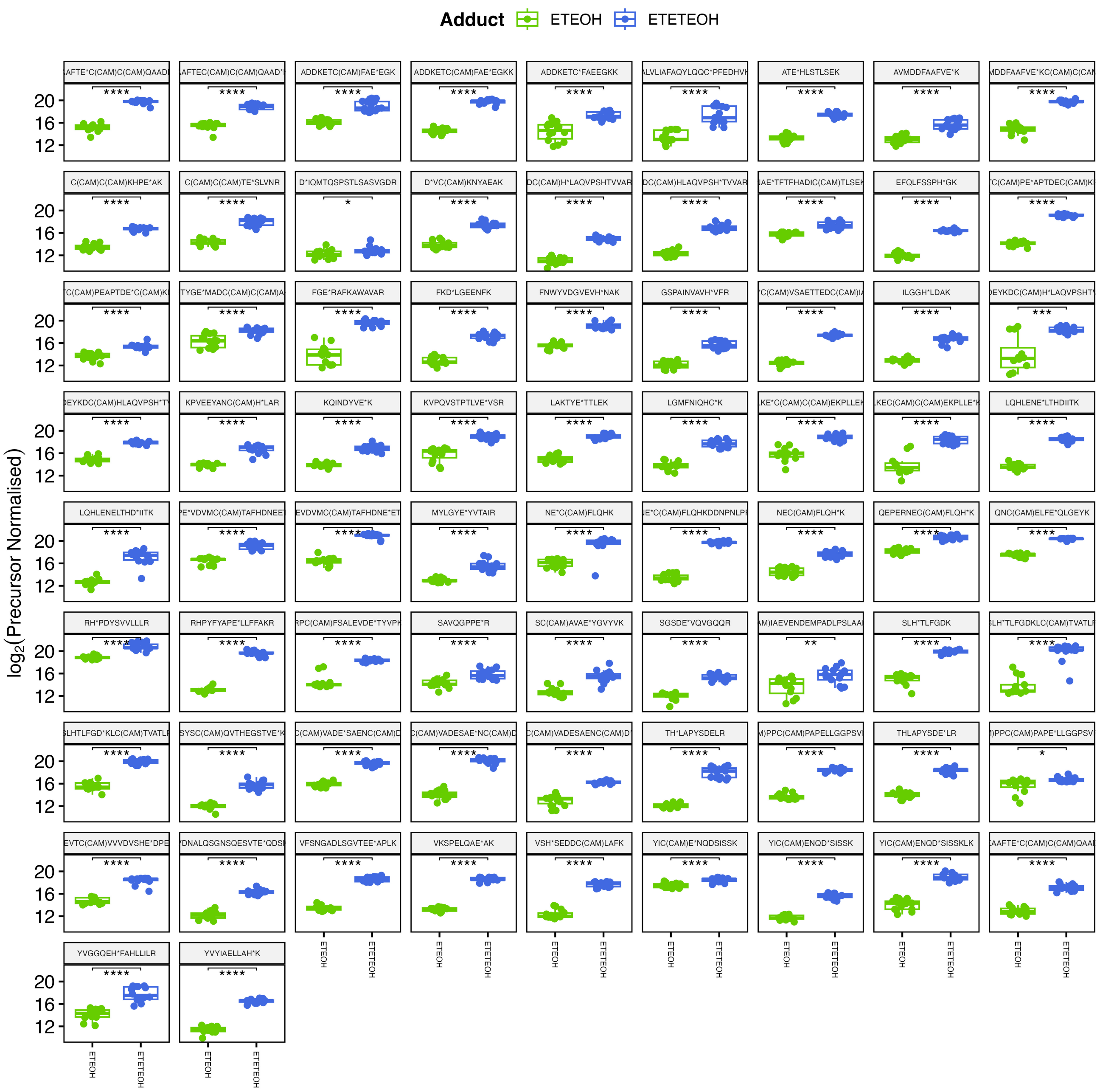

Figure S7 Multiple modification sites with HETE alkyl chain originating from HD exposure in samples only exposed to Q.

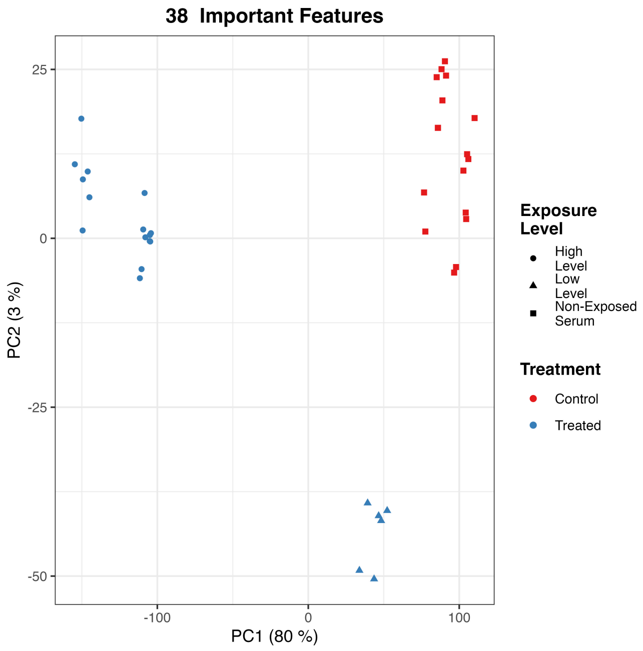

Figure S8 PCA when filtering the data for the 270 identified peptides by PCA and random forest. Furthermore, filtering allows for successful classification of the data in exposed and non-exposed serum via random forest or linear discriminant analysis (not shown), even for low level exposures.

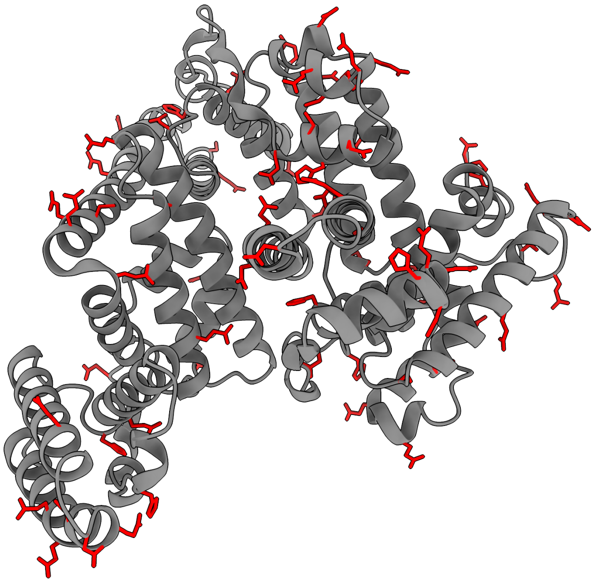

Figure S9 Visualization of 74 identified modification sites on HSA (side-chains in red). They are primarily located on the surface of the protein which is supported by the solvent-accessible surface area (SASA) calculated for these residues. Calculations with ChimeraX showed that the atoms of these sites account for only around 13 % of the atomic number of the entire protein (without hydrogen), while accounting for over 58 % of the SASA of the protein. The ChimeraX project is available from *https://github.com/Chemdrumz/Proteomics-SM*.

1.2.7 Experiment 4: Target-Analysis of Selected Peptides

1.2.7.1 Preparation of Cibacron Blue Beads (CibaMaB)

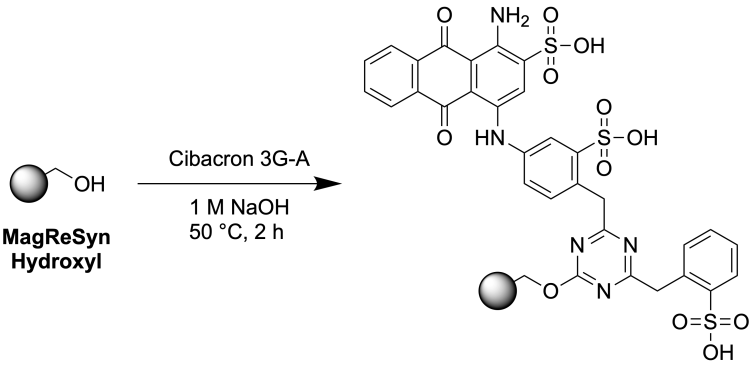

CibaMaB

MagReSyn Hydroxyl beads (20 mg mL^{-1}, 5 mL) were incubated on a magnetic rack and the supernatant removed. The beads were washed with HPLC grade H_2O (3 × 5 mL). Then, Cibacron Blue 3G-A solution (5 mg mL^{-1} in HPLC grade H_2O, 5 mL) was added followed by 2 M NaOH (5 mL). The resulting suspension was incubated at 50 °C and 1000 rpm for 2 h. The beads were again incubated on a magnetic rack, the supernatant removed and the remaining beads washed with HPLC grade H_2O until the supernatant remained colorless. The beads were then resuspended in HPLC grade H_2O to give a 50 mg mL^{-1} solution.

1.2.7.2 General Procedure for the Purification of HSA using Cibacron Blue Beads (GPHSA)

A broader study for the optimization of the HSA enrichment was performed including different pH conditions, NaCl concentrations and multiple washing steps. The optimized conditions are given in this section. KingFisher APEX 96-well plates were charged with

1. Beads plate: CibaMaBs (50 mg mL^{-1}, 10 µL) in HPLC grade H_2O (40 µL)
2. Binding plate: diluted blood serum (in 20 mM phosphate buffer with 0.15 M NaCl, 1:100; 100 µL)
3. Wash plate 1-3: 20 mM phosphate buffer with 0.2 M NaCl (100 µL)
4. Elution plate: 20 mM phosphate buffer with 0.4 M NaCl (50 µL)

with time program

1. Pick up beads from beads plate
2. Elute beads into binding plate and incubate for 12 min
3. Transfer beads to Wash 1 and incubate for 2 min
4. Transfer beads to Wash 2 and incubate for 2 min
5. Transfer beads to Wash 3 and incubate for 2 min
6. Transfer beads into elution plate
7. Transfer elution plate for external digest.
8. Remove beads

After enrichment, the samples in the elution plate were treated with DTT (100 mM, 2.5 µL) at 60 °C for 30 min, then with IAA (200 mM, 5 µL) at room temperature and in the absent of light for 30 min and finally alkylation was terminated with DTT (100 mM, 2.5 µL). The samples were diluted with EtOH (100 %, 60 µL) and the plate directly subjected as the binding plate to R2-P1 protocol as given in **GPPSP** using trypsin/Lys-C (10 µg mL^{-1}, 50 µL) at 37 °C for 4 h during external digest. Digestion was terminated by the addition of FoA (50 %, 0.5 µL).

1.2.7.3 Evaluation of Enrichment Quality

The following blood serum stock solutions were prepared

A. Blood serum in 20 mM phosphate buffer + 0.1 M NaCl (1:100)

B. Blood serum in 20 mM phosphate buffer (1:100)

Stock solution A was treated in triplicates and in accordance to **GPHSA**. After enrichment and prior to SP3/R2-P1, the samples on the binding plate were transferred onto the elution plate and diluted 100 %, 100 µL. Stock solution B was treated in triplicates using the same protocol given in **GPHSA**, however by using 20 mM phosphate buffer (100 µL) during the washing steps. The resulting samples were measured in dia-PASEF mode and evaluated with DIANN using a human proteome database (UP000005640) and default PTMs (UniMod:1, UniMod:4, UniMod:7). The results are presented in Figure 4 of the manuscript.

1.2.7.4 Optimization of prm-PASEF measurements

Measurement in standard dia-PASEF mode uses the following collisional energy (CE) equation:

$$\mathrm{CE} = 39.0 \cdot \mathrm{IM} - 3.4 \tag{1.1}$$

Following this linearity, for each target peptide the CE was calculated depending on its ion mobility and alternated with ΔCE = −5.0 eV, −2.5 eV, 2.5 eV, 5.0 eV and 7.5 eV. The CE with maximum area in the chromatogram of the corresponding peptide was determined via Skyline. The resulting targetlist is given in Table S13.

1.2.7.5 Target-Analysis of Selected peptides

Blood serum of three healthy individuals at exposure levels of 5.0 mM, 50 µM, 5 µM, 0.5 µM and plane CH_3CN (control) as given in **GPI** was prepared according to **GPHSA**. The samples were measured using the standard elution program first (see Instrumentation for Proteomic Sample Preparation and Analysis, referred to as long gradient). Also, the samples were measured with an adjusted elution program (referred to as short gradient): A linear gradient from 2 % B to 50 % B in 12.5 min and then from 50 % to 95 % in 2.5 min was performed. This composition was held for 5 min. The injection volume for enriched samples was 1.5 µL and 1.0 µL for unenriched samples. Mass spectrometry was performed in prm-PASEF mode using the targetlist given in Table S13 and the raw data was evaluated with Skyline (see Figure 5 in manuscript).

1.2.8 Experiment 5: Experimental Verification of Tripeptide in Blood Serum Exposed to Q

Blood serum from three healthy individuals was exposed Q as described in **GPI** at an exposure level of 5.0 mM. Proteomic sample preparation and digestion was performed according to **GPPSP** at 50 °C for 90 min using ProtK. The resulting samples were analyzed in prm-PASEF mode (Q: 530.2 ± 0.5 m/z; IM: 1.05 V s cm^{-2} – 1.15 V s cm^{-2}, RT: 20 ± 10 min; CE: 35 eV; MS1: 2 Hz) with the following instrumental parameters:

Table S7 timsTOF-settings for the measurement of the tripeptide in prm-PASEF mode. For not-listed parameters the default settings as given in Table S7 were used.

1/k0 start	0.45 V s cm^{-2}
1/k0 end	1.45 V s cm^{-2}
Cycle Ramp Time	100 ms
Scan Range MS1	100 m/z – 1700 m/z
Funnel 1 RF	450.0 Vpp
isCID Energy	0.0 eV
Funnel 2 RF	250.0 Vpp
Multipole RF	250.0 Vpp
Collision RF	500.0 Vpp
Collision Energy	10.0 eV
Transfer Time	50 µs
Pre-pulse Storage Time	8.0 µs

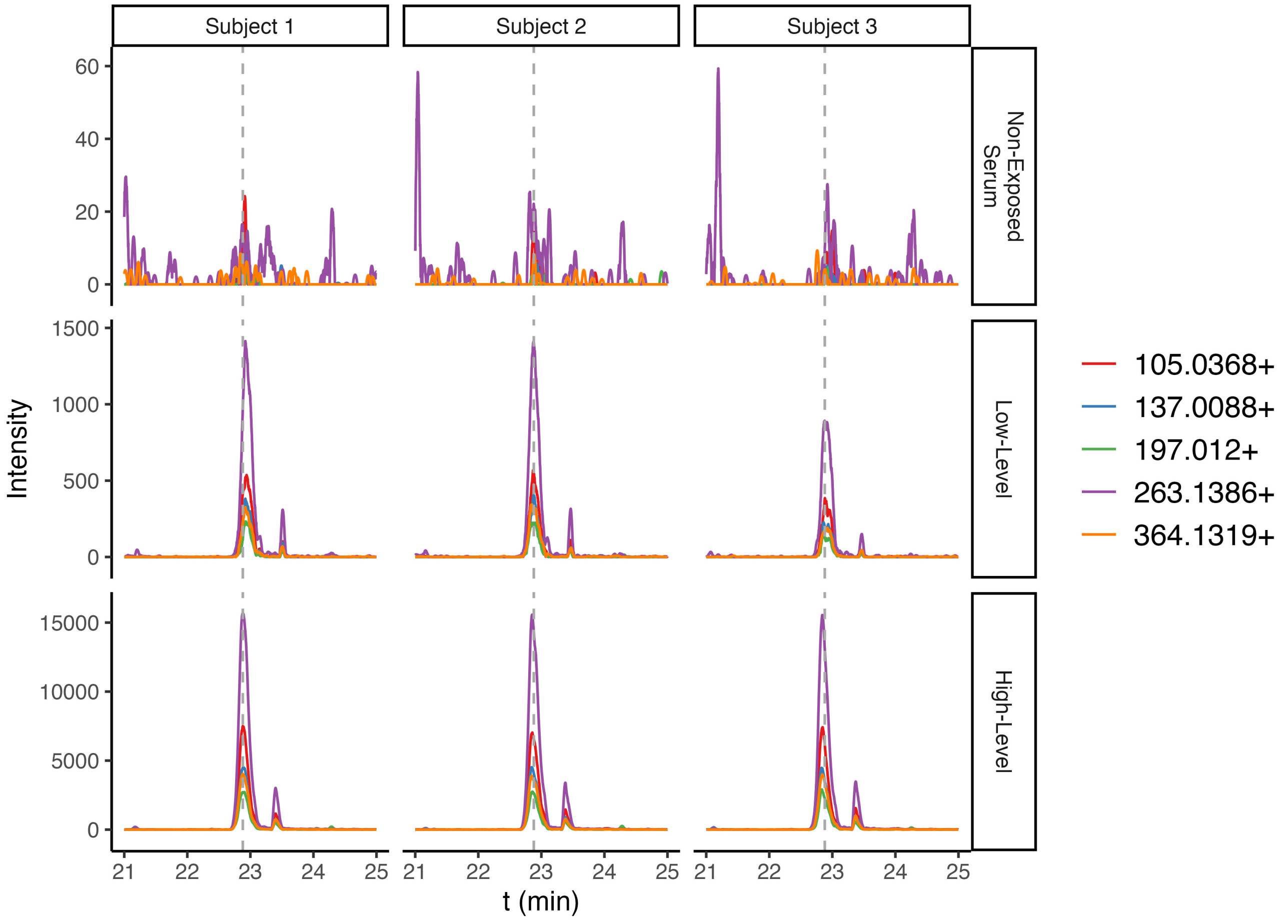

Figure S10 prm-PASEF of tripeptide in blood serum from three different individuals.

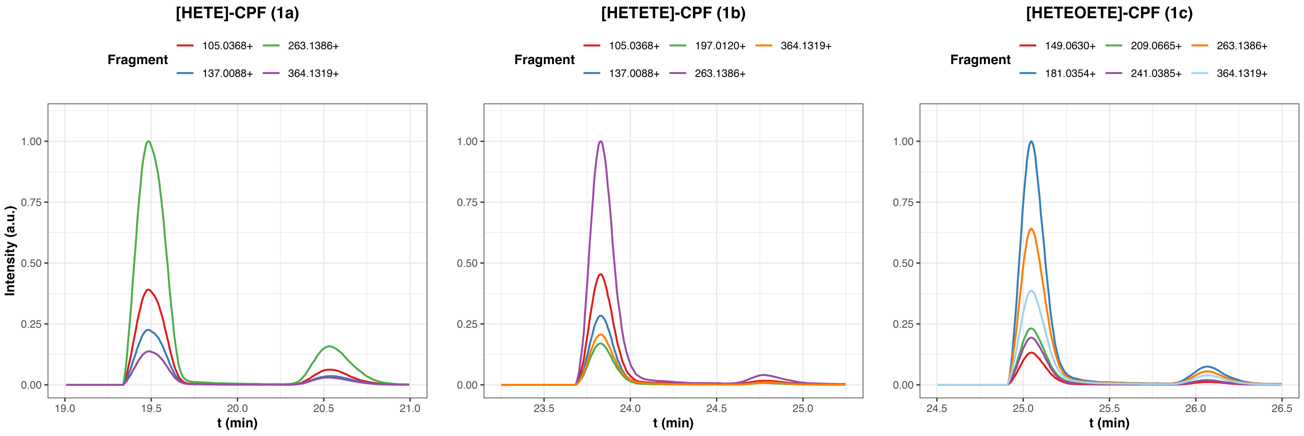

Figure S11 prm-PASEF of synthesized tripeptides revealing satellite peaks for all three adducts (Savitzky-Golay smoothened).

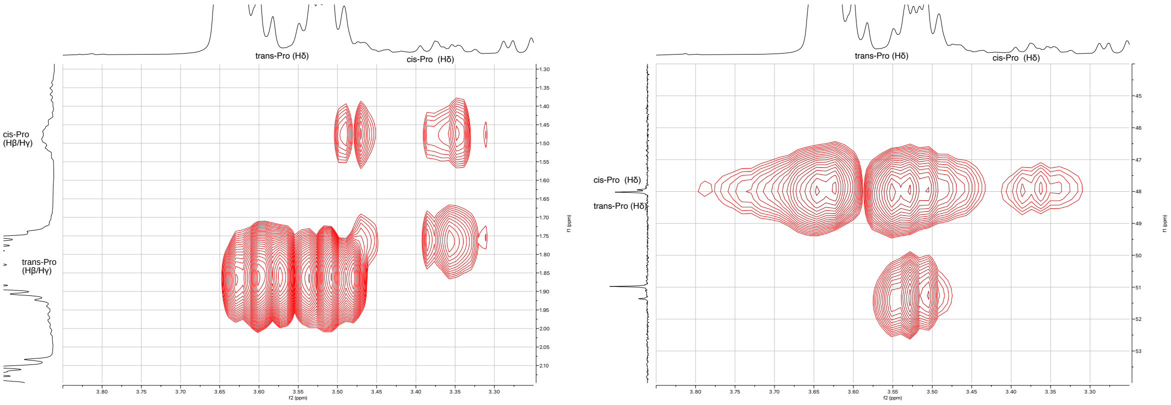

Figure S12 COSY (left) and HSQC (right) spectra (H_2O/D_2O, 9/1; water suppression) of tripeptide **1b** for the identification of the *cis-trans* prolyl isomers [23].

1.3 Synthesis of Standards

1.3.1 Solid-Phase Peptide Synthesis (SPPS)

General Procedure SPPS with Acid Labile Side-Chain Protection (GPSPPS-A)
The present protocol is used for batch sizes of 0.075 mmol – 0.1 mmol. It includes the following general steps:

1. Resin-Swelling in CH_2Cl_2 at room temperature (4.5 mL for 10 mL reactor vial, 9.0 mL for 30 mL reactor vial).
2. Addition of cycle amino acid in specified equivalents
3. Addition of coupling reagents in specified equivalents
4. Coupling for the specified time at specified temperature
5. Wash with DMF (4 × 4.5 mL for 10 mL reactor vial, 4 × 9.0 mL for 30 mL reactor vial).
6. Addition of piperazine (2 × 5.0 % in DMF), agitation at room temperature for 3 min and 15 min, respectively.
7. Wash with DMF (4 × 4.5 mL for 10 mL reactor vial, 4 × 9.0 mL for 30 mL reactor vial).
8. Repetition of steps 2 to 7 for each amino acid.
9. Wash alternately with CH_2Cl_2 and MeOH (4 × 4.5 mL for 10 mL reactor vial, 4 × 9.0 mL for 30 mL reactor vial)
10. Resin cleavage as specified

General Procedure SPPS with Base Labile Side-Chain Protection (GPSPPS-B)
The present protocol is used for batch sizes of 0.05 mmol – 0.4 mmol. It includes the following general steps:

1. Resin-Swelling in CH_2Cl_2 at room temperature (4.5 mL for 10 mL reactor vial, 9.0 mL for 30 mL reactor vial).
2. Addition of cycle amino acid in specified equivalents
3. Addition of coupling reagents in specified equivalents
4. Coupling for the specified time at specified temperature
5. Wash with DMF (4 × 4.5 mL for 10 mL reactor vial, 4 × 9.0 mL for 30 mL reactor vial).
6. Addition of piperazine (2 × 5.0 % in DMF), agitation at ambient conditions for 3 min and 15 min, respectively.
7. Wash with DMF (4 × 4.5 mL for 10 mL reactor vial, 4 × 9.0 mL for 30 mL reactor vial).
8. Repetition of steps 2 to 7 for each amino acid. For last cycle amino acid, skip steps 6-7.
9. Addition of 1 M KOH in MeOH (2 × 5 mL for 10 mL reactor vial, 2 × 10 mL for 30 mL reactor vial) and agitate at ambient conditions for 30 min.
10. Wash with MeOH (4 × 5 mL for 10 mL reactor vial, 4 × 10 mL for 30 mL reactor vial)
11. Wash alternately with CH_2Cl_2 and MeOH (4 × 4.5 mL for 10 mL reactor vial, 4 × 9.0 mL for 30 mL reactor vial)
12. Resin cleavage as specified

1.3.2 Synthesis of SM-Adducts to Glutamic Acid and Aspartic Acid

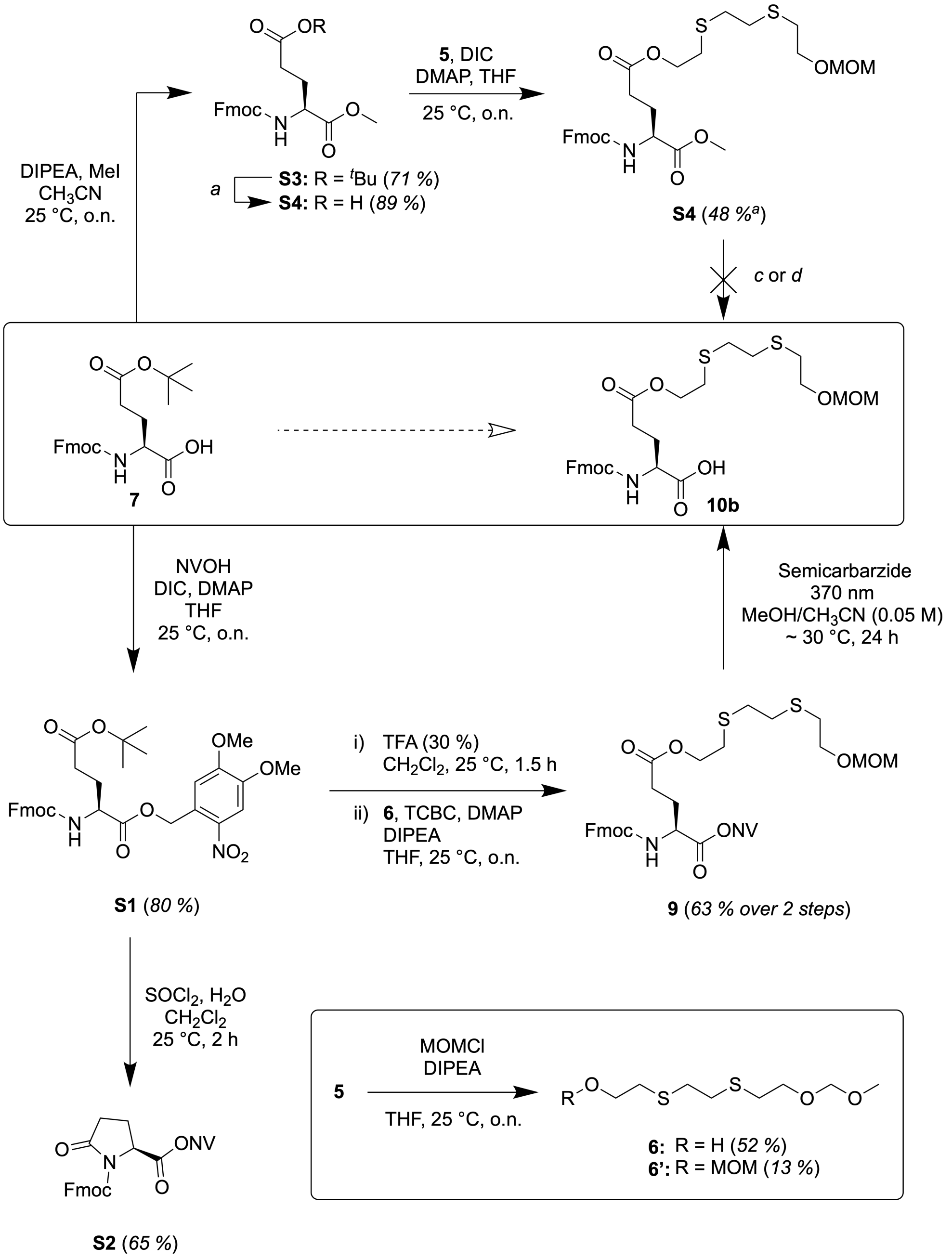

Scheme S3 Preparation of glutamic acid building block with thioether modification of the *gamma*-carboxylic acid at the example of the Q representatives. Approaching the synthesis using methyl protection failed due to unselective cleavage of the corresponding esters. Converting the *gamma*-carboxylic acid to the corresponding acyl chloride lead to direct cyclization and formation of the pyro-glutamic acid derivative **S2**. Using optimized conditions, the corresponding adducts for HD and T are prepared accordingly. *a*: TFA (30 % in CH_2Cl_2), 23 °C, 2 h; *b*: LiBr, DIPEA, CH_3CN, 25 °C, o.n.[24]; *c*: $AlCl_3$, dimethylaniline, CH_2Cl_2, reflux, 5 h[25].

1.3.2.1 Optimization of the Esterification

The reaction solutions were prepared in 0.1 mmol scales as 0.25 M solutions in oven-dried drum vials equipped with a stir bar and under inert atmosphere. Stock solutions were prepared and used whenever possible. The reactions were quenched with a TBAI solution (0.5 mL, 0.2 M in HPLC grade CH_3CN and an aliquot (50 µL in 950 µL HPLC grade CH_3CN) analyzed by HPLC-MS.

Table S8 Optimization of the esterification between glutamic acid **8** and alcohol **6**. [b]Only 0.2 equiv. [c]Same equivalents as **8**. [d]Formation of undesired side-product. *CDI*: Carbonyldiimidazole; *MPI*: *N*-Methylpyridinium iodide; *PPh_3*: Triphenylphosphine; *DIAD*: Diisopropylazodicarobxylate; *TCBC*: 2,4,6-Trichlorobenzoyl chloride; *DMC*: Dimethylcarbonate.

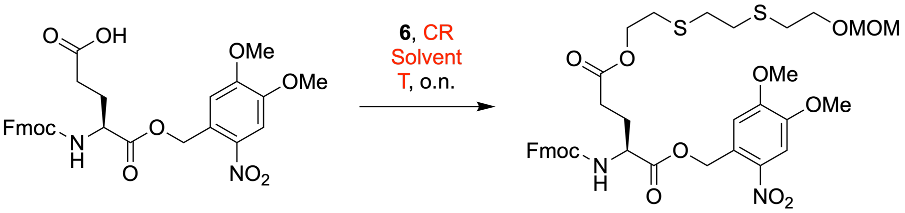

8 → **9**

Entry	Solvent	Coupling Reagent (CR)	CR [equiv]	8 [equiv]	6 [equiv]	T [°C]	Yield[a] [%]
1	THF	-	-	1.0	1.0	25	0
2	THF	CDI	1.0	1.0	1.0	25	34
3	THF	MPI, 2,6-Lutidine	1.0	1.0	1.0	25	6
4	THF	DIC, DMAP[b]	1.0	1.0	1.0	25	66
5	THF	TCBC, DIPEA[c], DMAP[b]	1.0	1.0	1.0	25	61
6	THF	PPh_3, DIAD	1.0	1.0	1.0	25	66
7	THF	PPh_3, DIAD	1.0	1.0	1.0	25	71
8	EtOAC	PPh_3, DIAD	1.0	1.0	1.0	25	69
9	CH_3CN	PPh_3, DIAD	1.0	1.0	1.0	25	60
10	CH_2Cl_2	PPh_3, DIAD	1.0	1.0	1.0	25	62
11	DMC	PPh_3, DIAD	1.0	1.0	1.0	25	29
12	DMF	PPh_3, DIAD	1.0	1.0	1.0	25	67
13	THF	PPh_3, DIAD	1.0	1.0	1.0	25	69
14	THF	PPh_3, DIAD	1.2	1.0	1.0	25	71
15	THF	PPh_3, DIAD	1.5	1.0	1.0	25	60[d]
16	THF	PPh_3, DIAD	2.0	1.0	1.0	25	44[d]
17	THF	PPh_3, DIAD	1.0	1.0	1.2	25	76
18	THF	PPh_3, DIAD	1.2	1.0	1.2	25	77
19	THF	PPh_3, DIAD	1.4	1.0	1.2	25	72[d]
20	THF	PPh_3, DIAD	1.0	1.2	1.0	25	79
21	THF	PPh_3, DIAD	1.2	1.2	1.0	25	77
22	THF	PPh_3, DIAD	1.4	1.2	1.0	25	78
23	THF	PPh_3, DIAD	1.0	1.2	1.0	25	75
24	THF	PPh_3, DIAD	1.0	1.2	1.0	40	68[d]
25	THF	PPh_3, DIAD	1.0	1.2	1.0	60	66[d]
26	THF	TCBC, DIPEA[c], DMAP[b]	1.0	1.0	1.0	25	74
27	THF	TCBC, DIPEA[c], DMAP[b]	1.2	1.2	1.0	25	77
28	THF	TCBC, DIPEA[c], DMAP[b]	1.2	1.0	1.2	25	83
29	THF	TCBC, DIPEA[c], DMAP[b]	1.5	1.0	1.0	25	58
30	THF	TCBC, DIPEA[c], DMAP[b]	1.5	1.2	1.0	25	69
31	THF	TCBC, DIPEA[c], DMAP[b]	1.5	1.0	1.2	25	62

1.3.2.2 Preparation and Characterization of Substrates

General Procedure for MOM-Protection of Alcohols (GPA)
A heat-gun dried round-bottomed flask equipped with a stir bar, reflux condenser and septum was charged with the corresponding diol (20.0 mmol, 1.0 equiv) and THF (40 mL). To the resulting solution, MOMCl (20.0 mmol, 1.0 equiv) was added in one portion followed by the dropwise addition of DIPEA (20.0 mmol, 1.0 equiv). The resulting reaction mixture was stirred at 25 °C overnight. The formed precipitate was removed via filtration of the reaction mixture through cotton and celite with the aid of THF and the filtrate concentrated under reduced pressure.

General Procedure of the Esterification of Side-Chain Carboxylic acids (GPB)
A heat-gun dried, two-necked round bottomed flask equipped with a stir bar and a septum was charged with the corresponding α-carboxylic acid protected amino acid (1.0 equiv), DMAP (0.2 equiv) and THF (6.0 mL). Then, DIPEA (1.0 equiv) was added at 0 °C and the solution stirred for 5 min. After the dropwise addition of TCBC (1.2 equiv) and subsequent stirring for 10 min, a solution of the corresponding alcohol (1.2 equiv) in THF (2 mL) was added dropwise.
The resulting reaction mixture was stirred at 0 °C for 1 h, allowed to warm up to room temperature and stirred overnight. The formed slurry was filtered through cotton and celite with the aid of THF and all volatiles removed under reduced pressure.

General Procedure for the Photocleavage of the NV-Protecting Group (GPC)
Preparation of 1.0 M *Semicarbazide in MeOH*: Semicarbazide hydrochloride (50 mmol) was added to a 1.0 M KOH in MeOH solution (50 mL) in one portion and the resulting slurry sonicated for 30 min. Then, the slurry was filtered through cotton into an oven-dried vial with molecular sieve (4 Å) and stored until further use.

Irradiation of NV-Protected Amino Acid: To an oven-dried Schlenk tube equipped with a stir bar and a cooling finger the corresponding amino acid building block (1.0 equiv) in MeCN (0.1 M) followed by 1.0 M Semicarbazide in MeOH solution (10.0 equiv) was added and the resulting solution degassed by evacuation and flushing with Ar three times. Then, the reaction mixture was irradiated at 370 nm (2 x Kessil PR160L, ≈ 3 cm distance) for 24 h. For improved temperature control, the set up was cooled by continuous external airflow. Irradiation was terminated and the reaction mixture filtered through cotton and celite by the aid of additional CH_3CN. After removal of MeOH under reduced pressure, the filtrate was filtered again through cotton and celite. The filtrate was concentrated under reduced pressure to receive crude product.

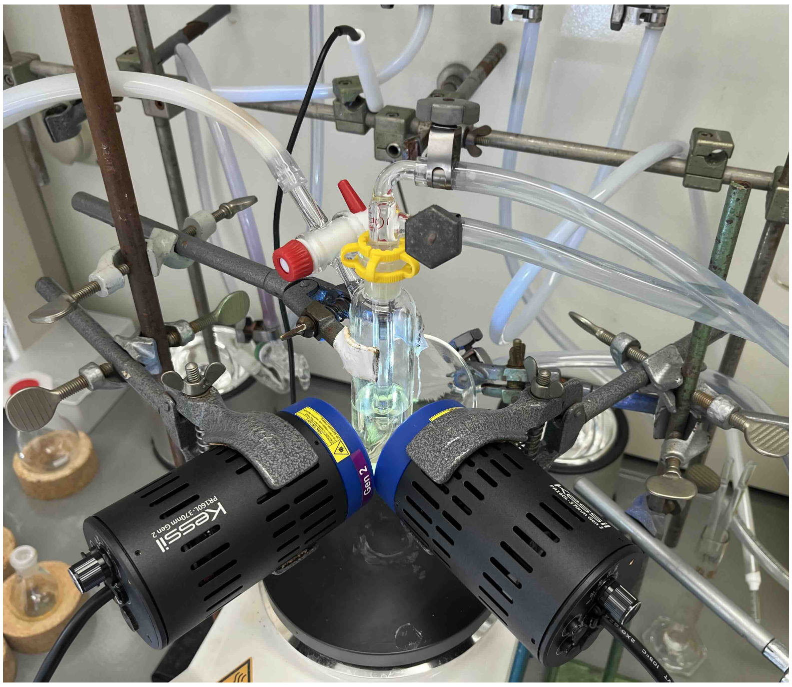

Figure S13 Set up for the irradiation of Nvoc protected amino acids. The set up consists of Schlenk flask with cooling finger, Ar supply via hose connector and two irradiation lamps. From the back, the system is additionally cooled using pressured air.

Glutamic ester (S1)

A heat-gun dried two-necked round-bottomed flask equipped with a stir bar and septum was charged with **7** (11.0 mmol, 1.1 equiv), NVOH (10.0 mmol, 1.0 equiv), DMAP (2.0 mmol, 0.2 equiv) and THF (10 mL) and the resulting reaction mixture cooled to 0 °C. To the resulting suspension, DIC (11.0 mmol, 1.1 equiv) was added dropwise and the resulting reaction mixture allowed to warm up to 25 °C and stirred for 21 h. The resulting orange slurry was filtered through cotton with the aid of THF and the filtrate concentrated under reduced pressure. Purification via flash column chromatography (Isolera Sfar HC Duo 50 g, 80 mL min^{-1}, n-Hex/Et_2O, 25 % Et_2O (10.0 CV), 25 % – 75 % Et_2O (10.0 CV), 75 % Et_2O (5.0 CV)) afforded the title compound as a greenish honey which was used without further drying.

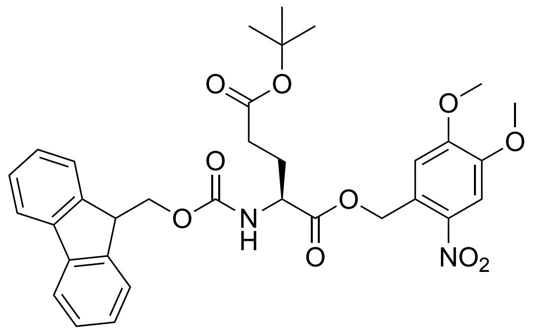

$C_{33}H_{36}N_2O_{10}$
MW: 620.66
S1

^{1}**H-NMR** (400 MHz, $CDCl_3$) δ 7.75 (d, *J* = 7.6 Hz, 2H), 7.71 (s, 1H), 7.57 (d, *J* = 7.5 Hz, 2H), 7.39 (q, *J* = 6.9 Hz, 3H), 7.34 – 7.25 (m, 2H), 7.03 (s, 1H), 5.68 – 5.53 (m, 3H), 4.47 (td, *J* = 8.4, 4.7 Hz, 1H), 4.42 – 4.30 (m, 2H), 4.19 (t, *J* = 7.1 Hz, 1H), 3.96 (s, 3H), 3.94 (s, 3H), 2.49 – 2.30 (m, 2H), 2.23 (dtd, *J* = 14.3, 7.2, 4.9 Hz, 1H), 2.10 – 1.97 (m, 1H), 1.45 (s, 9H). ^{13}**C-NMR** (101 MHz, $CDCl_3$) δ 172.1, 171.8, 156.2, 153.8, 148.3, 143.8, 143.6, 141.3, 139.6, 127.8, 127.1, 126.8, 125.1, 120.0, 110.1, 108.2, 81.1, 67.2, 64.2, 56.6, 56.4, 53.9, 47.1, 31.5, 28.1, 27.0. **LC-HRMS** (Discovery HS C18, ESI/qTOF) calc. for $C_{33}H_{37}N_2O_{10}^+$ $[M+H]^+$: 621.2443 m/z; found 621.2448 m/z. **FT-IR** (neat) [cm^{-1}]: 1748 (m), 1728 (m), 1692 (s), 1522 (vs), 1328 (m), 1273 (vs), 1220 (m), 1147 (s), 1065 (s), 985 (m), 873 (w), 796 (w), 737 (m). **mp**: 69 °C. **R**$_f$ (n-Hex/EtOAc, 3/1): 0.26.

Glutamic acid derivative (8)

In a round-bottomed flask equipped with a stir bar, protected glutamic acid **S1** was treated with TFA (30 % in CH_2Cl_2, 10 mL) at 25 °C for 1 h. After removal of all volatiles under reduced pressure, the residue was recrystallized from acetone and the collected solids dried under high vacuum to afford the title compound as a off-white solid (4.290 g, 7.60 mmol, 76 % over two steps).

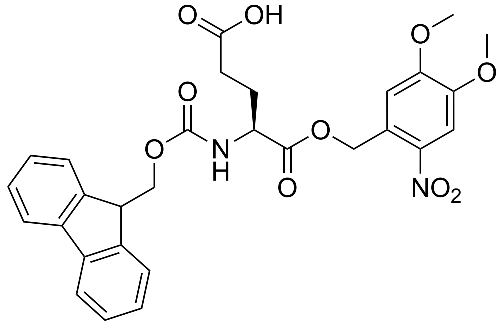

$C_{29}H_{28}N_2O_{10}$
MW: 564.55
8

^{1}**H-NMR** (400 MHz, DMSO-d^6) δ 12.20 (s, 1H), 7.90 (dd, *J* = 16.4, 7.6 Hz, 3H), 7.72 – 7.65 (m, 3H), 7.40 (tt, *J* = 7.5, 1.4 Hz, 2H), 7.30 (t, *J* = 7.4 Hz, 2H), 7.16 (s, 1H), 5.51 – 5.38 (m, 2H), 4.36 – 4.17 (m, 4H), 3.86 (d, *J* = 3.7 Hz, 7H), 2.36 (t, *J* = 7.4 Hz, 2H), 2.12 – 1.98 (m, 1H), 1.95 – 1.80 (m, 1H). ^{13}**C-NMR** (101 MHz, DMSO-d^6) δ 173.7, 171.8, 156.2, 153.4, 147.9, 143.8, 143.7, 140.7, 139.4, 127.6, 127.1, 127.0, 126.3, 125.2, 125.2, 120.1, 110.8, 108.2, 65.7, 63.2, 56.2, 56.1, 53.3, 46.6, 29.9, 25.8. **LC-HRMS** (Discovery HS C18, ESI/qTOF) calc. for $C_{29}H_{29}N_2O_{10}^+$ $[M+H]^+$: 565.1817 m/z; found 565.1818 m/z. **FT-IR** (neat) [cm^{-1}]: 3384 (m), 3306 (m), 2941 (w), 1755 (m), 1725 (s), 1683 (s), 1522 (s), 1444 (m), 1433 (m), 1331 (m), 1278 (vs), 1214 (vs), 1169 (s), 1149 (s), 1105 (s), 1065 (s), 985 (m), 869 (m), 797 (m), 736 (vs), 621 (m). **mp:** 152 °C.

Pyroglutamic acid (S2)

A round-bottomed flask covered with aluminum foil and equipped with a stir bar was charged with **S1** (1.00 mmol, 1.0 equiv) and $SOCl_2$ (10.0 mmol, 10.0 equiv). After the addition of H_2O (1.0 mmol, 1.0 equiv) the resulting reaction mixture stirred for 2 h. Then, the reaction was quenched with toluene (2 × 1 mL) and concentrated under reduced pressure and successful reaction checked via NMR. Purification via flash column chromatography (Isolera Sfar HC Duo 25 g, 80 mL min^{-1}, n-Hex/EtOAc, 20 % – 40 % EtOAc (17.0 CV), 40 % – 70 % EtOAc (8.0 CV), 70 % EtOAc (5.0 CV)) and lyophilization afforded the title compound as a greenish powder (358 mg, 0.65 mmol, 65 %).

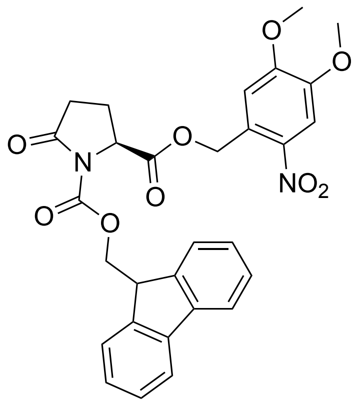

$C_{29}H_{26}N_2O_9$
MW: 546.53
S2

^{1}H-NMR (400 MHz, $CDCl_3$) δ 7.77 – 7.70 (m, 3H), 7.70 (s, 1H), 7.66 (dd, J = 7.5, 1.0 Hz, 1H), 7.43 – 7.34 (m, 2H), 7.31 (td, J = 7.5, 1.2 Hz, 1H), 7.27 (td, J = 7.5, 1.2 Hz, 1H), 6.91 (s, 1H), 5.63 – 5.43 (m, 2H), 4.75 (dd, J = 9.5, 2.5 Hz, 1H), 4.49 (ddd, J = 42.4, 10.6, 7.3 Hz, 2H), 4.24 (t, J = 7.2 Hz, 1H), 3.93 (s, 3H), 3.90 (s, 3H), 2.76 (ddd, J = 17.6, 10.6, 9.5 Hz, 1H), 2.61 (ddd, J = 17.6, 9.3, 3.1 Hz, 1H), 2.44 (ddt, J = 13.4, 10.6, 9.4 Hz, 1H), 2.19 (ddt, J = 13.4, 9.5, 2.9 Hz, 1H). **^{13}C-NMR** (101 MHz, $CDCl_3$) δ 172.4, 170.6, 153.8, 151.7, 148.4, 143.3, 143.1, 141.3, 141.2, 139.7, 127.9, 127.3, 127.2, 126.2, 125.3, 125.2, 120.0, 110.3, 108.2, 69.1, 64.7, 58.8, 56.7, 56.4, 46.5, 31.2, 21.9. **LC-HRMS** (Discovery HS C18, ESI/qTOF) calc. for $C_{29}H_{27}N_2O_9^+$ $[M + H]^+$: 547.1711 m/z; found 547.1715 m/z. **FT-IR** (neat) [cm^{-1}]: 1796 (w), 1752 (m), 1716 (m), 1580 (w), 1520 (m), 1450 (w), 1386 (w), 1327 (m), 1301 (m), 1275 (s), 1221 (m), 1175 (s), 1066 (m), 1032 (w), 982 (w), 869 (w), 796 (w), 759 (m), 741 (m), 600 (w). **mp**: 93 °C. **R$_f$** (n-Hex/EtOAc, 1/1): 0.37.

Glutamic ester (S3)

A heat-gun dried, two-necked round-bottomed flask was equipped with a stir bar, reflux condenser and septum was charged with **7** (10.0 mmol, 1.0 equiv) and CH_3CN (20 mL). To the resulting solution, DIPEA (10.0 mmol, 1.0 equiv) was added and the solution stirred for 5 min. Then, MeI (15.0 mmol, 1.5 equiv) was added dropwise and the reuslting reaction mixture stirred at 25 °C overnight. The solvent was removed under reduced pressure and EtOAc (50 mL) was added. The formed slurry was washed with 10 % aq. NH_4Cl (3 × 25 mL), sat. aq. $NaHCO_3$ (3 × 25 mL) and brine (2 × 25 mL). The organic layer was dried over Na_2SO_4, filtered and concentrated under reduced pressure. Purification via column chromatography (Isolera Sfar HC Duo 50 g, 80 mL min^{-1}, n-Hex/EtOAc, 15 % – 30 % EtOAc (20.0 CV)) afforded the title compound as a colorless liquid (3.14 g, 7.1 mmol, 71 %).

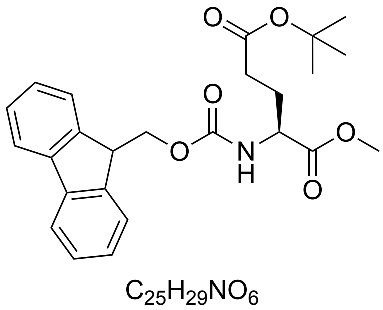

$C_{25}H_{29}NO_6$
MW: 439.51
S3

^{1}H-NMR (400 MHz, $CDCl_3$) δ 7.76 (dt, J = 7.6, 1.0 Hz, 2H), 7.60 (dd, J = 7.6, 4.3 Hz, 2H), 7.44 – 7.37 (m, 2H), 7.31 (tt, J = 7.5, 1.0 Hz, 2H), 5.48 (d, J = 8.2 Hz, 1H), 4.39 (qd, J = 10.5, 7.4 Hz, 3H), 4.22 (t, J = 7.0 Hz, 1H), 3.76 (s, 3H), 2.42 – 2.23 (m, 2H), 2.23 – 2.10 (m, 1H), 2.04 – 1.90 (m, 1H), 1.45 (s, 9H). **^{13}C-NMR** (101 MHz, $CDCl_3$) δ 172.5, 172.0, 156.0, 143.9, 141.3, 127.7, 127.1, 125.1, 120.0, 80.9, 67.1, 53.5, 47.1, 31.4, 28.1, 27.5. **R$_f$** (n-Hex/EtOAc, 3/1): 0.41.

Glutamic acid derivate (S4)

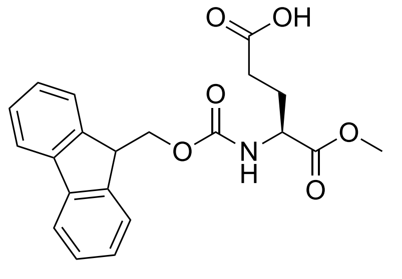

$C_{21}H_{21}NO_6$
MW: 383.40
S4

In a round-bottomed flask equipped with a stir bar, glutamic acid derivative **S3** was treated with TFA (30 % in CH_2Cl_2, 20 mL) at 23 °C for 2 h. The volatiles were removed under reduced pressure and the residue co-evaporated with CH_2Cl_2 several times. Recrystallization from CH_2Cl_2/n-Hex, filtration and drying under ambient conditions afforded the title compound as a white solid (2.45 g, 6.4 mmol, 64 % over two steps).
^{1}**H-NMR** (400 MHz, $CDCl_3$) δ 7.75 (dt, *J* = 7.6, 1.0 Hz, 2H), 7.62 – 7.50 (m, 2H), 7.39 (ddt, *J* = 8.4, 7.6, 1.6 Hz, 2H), 7.31 (tt, *J* = 7.4, 1.5 Hz, 2H), 5.45 (d, *J* = 8.2 Hz, 1H), 4.52 – 4.35 (m, 3H), 4.21 (t, *J* = 6.8 Hz, 1H), 3.75 (s, 3H), 2.53 – 2.34 (m, 2H), 2.09 (ddt, *J* = 103.9, 14.7, 7.3 Hz, 2H). ^{13}**C-NMR** (101 MHz, $CDCl_3$) δ 177.7, 172.3, 156.0, 143.7, 141.3, 127.8, 127.1, 125.1, 120.0, 67.1, 53.1, 47.2, 29.8, 27.4. Spectral data matches literature[26].

Alcohol (6)

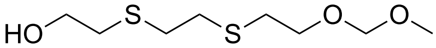

$C_8H_{18}O_3S_2$
MW: 226.35
6

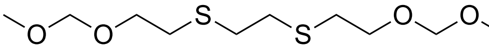

$C_{10}H_{22}O_4S_2$
MW: 270.40
6'

According to **GPA** using 3,6-dithia-1,8-octanediol (**5**) (20.0 mmol). Purification of the crude product via column chromatography (Isolera Sfar HC Duo 50 g, 100 mL min^{-1}, n-Hex/EtOAc, 10 % – 50 % EtOAc (24.0 CV)) afforded the title compound as a colorless liquid (2.38 g, 10.5 mmol, 52 %) and undesired bisprotected side-product **6'** as a colorless oil (737 mg, 2.7 mmol, 13 %).
^{1}**H-NMR** (400 MHz, $CDCl_3$) δ 4.65 (s, 2H), 3.75 (q, *J* = 6.0 Hz, 3H), 3.72 (t, *J* = 6.5 Hz, 2H), 3.38 (s, 2H), 2.86 – 2.72 (m, 8H), 2.37 (t, *J* = 6.1 Hz, 1H). ^{13}**C-NMR** (101 MHz, $CDCl_3$) δ 96.5, 67.4, 60.7, 55.4, 35.3, 32.6, 32.0, 31.9. **LC-HRMS** (Discovery HS C18, ESI/qTOF) calc. for $C_8H_{19}O_3S_2^+$ [M + H]$^+$: 227.0770 m/z; found 227.0772 m/z. **FT-IR** (neat) [cm^{-1}]: 3437 (br), 2924 (m), 2883 (m), 2824 (w), 1464 (w), 1440 (w), 1424 (w), 1381 (w), 1290 (w), 1202 (m), 1147 (m), 1105 (m), 1064 (s), 1029 (vs), 918 (m). **R**$_f$ (n-Hex/EtOAc, 2/1): 0.13.
Bisprotected Side-Product: ^{1}**H-NMR** (400 MHz, $CDCl_3$) δ 4.65 (s, 4H), 3.72 (t, *J* = 6.6 Hz, 4H), 3.38 (s, 6H), 2.82 – 2.74 (m, 8H). ^{13}**C-NMR** (101 MHz, $CDCl_3$) δ 96.5, 67.3, 55.3, 32.6, 32.0. **LC-HRMS** (Discovery HS C18, ESI/qTOF) calc. for $C_{10}H_{23}O_4S_2^+$ [M + H]$^+$: 271.1032 m/z; found 271.1033 m/z. **FT-IR** (neat) [cm^{-1}]: 2926 (m), 2886 (m), 2823 (w), 1466 (w), 1440 (w), 1406 (w), 1380 (w), 1290 (w), 1202 (m), 1145 (s), 1104 (s), 1065 (s), 1025 (vs), 916 (s), 653 (m), 626 (m). **R**$_f$ (n-Hex/EtOAc, 2/1): 0.43.

Glutamic ester (S5)

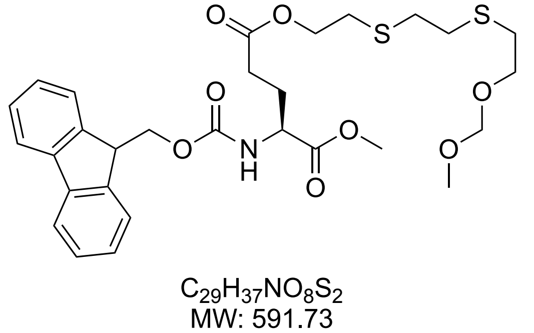

$C_{29}H_{37}NO_8S_2$
MW: 591.73
S5

A heat-gun dried, two-necked round-bottomed flask equipped with a stir bar, reflux condenser and septum was charged with **S4** (4.0 mmol, 1.0 equiv), **6** (4.0 mmol, 1.0 equiv) and DMAP (0.8 mmol, 0.2 equiv) and the set up evacuated and flushed with Ar twice. Then, THF (8 mL) was added and the reaction mixture stirred until homogeneous. To this solution DIC (4.0 mmol, 1.0 equiv) was added and the resulting reaction mixture stirred at 25 °C overnight and then at 60 °C for 2.5 h. The formed precipitate was removed by filtration of the resutling slurry through cotton and celite and the collected filtrate was concentrated under reduced pressure. Purification via column chromatography (Isolera Sfar HC C18 Duo 60 g, 50 mL min^{-1}, H_2O/CH_3CN, 40 % – 70 % CH_3CN (42.0 CV)) afforded the title compound as a transparent liquid (1.158 g, 1.9 mmol, 48 %).
^{1}**H-NMR** (400 MHz, $CDCl_3$) δ 7.77 (dd, *J* = 7.6, 1.2 Hz, 2H), 7.60 (dd, *J* = 7.5, 3.6 Hz, 2H), 7.40 (tt, *J* = 7.8, 1.4 Hz, 2H), 7.32 (tt, *J* = 7.5, 1.3 Hz, 2H), 5.47 (d, *J* = 8.3 Hz, 1H), 4.63 (s, 2H), 4.47 – 4.34 (m, 3H), 4.23 (t, *J* = 6.9 Hz, 2H), 3.77 (s, 3H), 3.70 (t, *J* = 6.5 Hz, 2H), 3.36 (s, 3H), 2.82 – 2.70 (m, 8H), 2.53 – 2.33 (m, 2H), 2.13 (ddt, *J* = 94.1, 14.4, 7.1 Hz, 2H). ^{13}**C-NMR** (101 MHz, $CDCl_3$) δ 172.4, 172.3, 155.9, 143.9, 141.3, 127.7, 127.1, 125.1, 120.0, 96.5, 67.3, 67.1, 63.6, 55.3, 53.3, 52.6, 47.2, 32.5, 32.4, 32.0, 30.5, 30.1, 27.6. **LC-HRMS** (Discovery HS C18, ESI/qTOF) calc. for $C_{29}H_{38}NO_8S_2^+$ [M + H]$^+$: 592.2033 m/z; found 592.2038 m/z. **FT-IR** (neat)

[cm^{-1}]: 3334 (br), 2950 (w), 1722 (vs), 1523 (m), 1449 (m), 1208 (s), 1168 (m), 1147 (s), 1105 (m), 1065 (s), 1032 (s), 916 (w), 760 (m), 741 (m), 634 (m).

Glutamic ester (9)

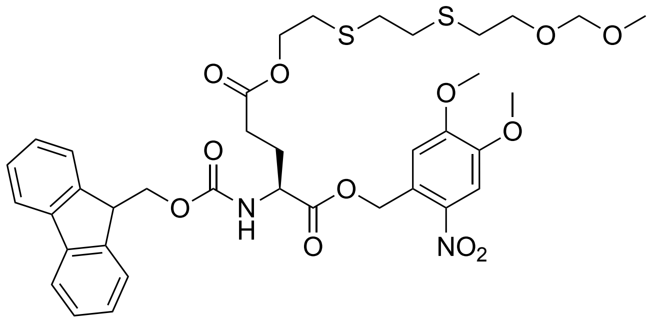

$C_{37}H_{44}N_2O_{12}S_2$
MW: 772.88
9

According to **GPB** using **6** (2.4 mmol). Purification of the crude product via column chromatography (Isolera Sfar HC Duo 25 g, 80 mL min^{-1}, CH_2Cl_2/CH_2Cl_2 + 1.0 % MeOH (A/B), 10 % B (1.0 CV), 10 % – 100 % B (25.0 CV), 100 % B (1.0 CV)) afforded the title compound as a yellow sticky solid (1.29 g, 1.67 mmol, 83 %; 9 % rsm **8**). **^{1}H-NMR** (400 MHz, $CDCl_3$) δ 7.76 (d, J = 7.6 Hz, 2H), 7.72 (s, 1H), 7.57 (d, J = 7.5 Hz, 2H), 7.39 (q, J = 7.0 Hz, 2H), 7.29 (q, J = 7.0 Hz, 2H), 7.01 (s, 1H), 5.59 (d, J = 3.7 Hz, 2H), 5.54 (d, J = 8.0 Hz, 1H), 4.62 (s, 2H), 4.50 (td, J = 8.5, 4.9 Hz, 1H), 4.39 (dd, J = 7.2, 2.2 Hz, 2H), 4.24 (t, J = 6.8 Hz, 2H), 4.20 (t, J = 7.2 Hz, 1H), 3.96 (s, 3H), 3.95 (s, 4H), 3.69 (t, J = 6.5 Hz, 2H), 3.36 (s, 3H), 2.81 – 2.70 (m, 8H), 2.58 – 2.40 (m, 2H), 2.37 – 2.24 (m, 1H), 2.14 – 2.00 (m, 1H). **^{13}C-NMR** (101 MHz, $CDCl_3$) δ 172.4, 171.6, 156.1, 153.8, 148.4, 143.7, 141.3, 139.8, 127.8, 127.1, 126.4, 125.0, 120.0, 110.3, 108.2, 96.5, 67.3, 67.2, 64.4, 63.7, 56.6, 56.4, 55.4, 53.6, 47.1, 32.5, 32.4, 32.0, 30.5, 30.2, 27.1. **LC-HRMS** (Discovery HS C18, ESI/qTOF) calc. for $C_{37}H_{48}N_3O_{12}S_2^+$ $[M + NH_4]^+$: 790.2674 m/z; found 790.2700 m/z. **FT-IR** (neat) [cm^{-1}]: 3314 (w), 2934 (w), 1728 (m), 1689 (m), 1582 (w), 1523 (s), 1450 (m), 1327 (m), 1274 (vs), 1221 (m), 1168 (s), 1147 (m), 1104 (m), 1065 (vs), 1031 (m), 986 (m), 873 (w), 796 (m), 759 (m), 739 (s). **R$_f$** (1 % MeOH in CH_2Cl_2): 0.20.

Glutamic ester (10b)

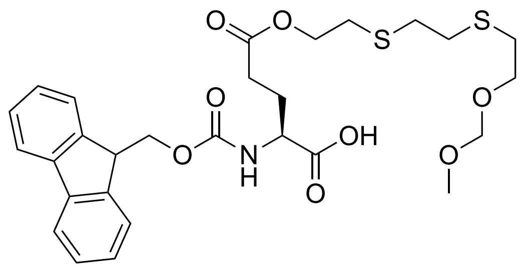

$C_{28}H_{35}NO_8S_2$
MW: 577.71
10b

According to **GPC** using **9** (1.66 mmol). Purification via column chromatography (Isolera Sfar HC Duo 25 g, 80 mL min^{-1}, CH_2Cl_2/CH_2Cl_2 + 10 % MeOH (A/B), 10 % B (5.0 CV), 10 % – 20 % B (5.0 CV), 20 % B (10.0 CV) afforded the title compound as a brownish honey (630 mg, 1.1 mmol, 65 %). **^{1}H-NMR** (400 MHz, $CDCl_3$) δ 7.77 (d, J = 7.6 Hz, 3H), 7.59 (d, J = 7.0 Hz, 3H), 7.40 (tt, J = 7.3, 1.3 Hz, 2H), 7.32 (tt, J = 7.4, 1.1 Hz, 2H), 5.58 (d, J = 7.8 Hz, 1H), 4.66 (d, J = 1.7 Hz, 2H), 4.47 – 4.38 (m, 3H), 4.25 (t, J = 6.6 Hz, 2H), 4.22 (t, J = 6.8 Hz, 1H), 3.72 (t, J = 6.6 Hz, 2H), 3.39 (s, 3H), 2.81 – 2.74 (m, 8H), 2.58 – 2.38 (m, 1H), 2.19 (dtd, J = 75.9, 14.2, 6.9 Hz, 1H). **^{13}C-NMR** (101 MHz, $CDCl_3$) δ 174.0, 172.6, 156.1, 143.8, 141.3, 127.8, 127.1, 125.1, 120.0, 96.3, 67.4, 67.2, 64.0, 55.4, 53.0, 47.2, 32.5, 32.4, 31.9, 30.6, 30.1, 27.3. **LC-HRMS** (Discovery HS C18, ESI/qTOF) calc. for $C_{28}H_{36}NO_8S_2^+$ $[M + H]^+$: 578.1877 m/z; found 578.1884 m/z. **FT-IR** (neat) [cm^{-1}]: 3312 (w), 2940 (m), 2826 (w), 1716 (vs), 1524 (m), 1449 (m), 1416 (w), 1324 (w), 1205 (s), 1168 (s), 1147 (s), 1102 (m), 1064 (s), 1031 (vs), 919 (w), 760 (m), 740 (s), 646 (m), 618 (m). **R$_f$** (5 % MeOH CH_2Cl_2): 0.1.

Alcohol (S6)

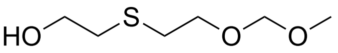

$C_6H_{14}O_3S$
MW: 166.24
S6

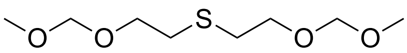

$C_8H_{18}O_4S$
MW: 210.29
S6'

According to **GPA** using thiodiglycol (**TDG**) (20.0 mmol). Purification via flash column chromatography (Isolera Sfar HC Duo 50 g, 120 mL min^{-1}, n-Hex/EtOAc, 10 % – 60 % EtOAc (25.0 CV)) afforded the title compound as a colorless liquid (1.77 g, 10.6 mmol, 53 %) and undesired bisprotected side-product **S6'** as a colorless oil (557 mg, 2.6 mmol, 13 %).
^{1}**H-NMR** (400 MHz, $CDCl_3$) δ 4.66 (s, 2H), 3.76 (q, *J* = 5.9 Hz, 2H), 3.72 (t, *J* = 6.4 Hz, 2H), 3.39 (s, 3H), 2.79 (t, *J* = 5.9 Hz, 2H), 2.78 (t, *J* = 6.4 Hz Hz, 2H), 2.56 (t, *J* = 6.1 Hz, 1H). ^{13}**C-NMR** (101 MHz, $CDCl_3$) δ 96.5, 67.3, 60.8, 55.4, 35.9, 31.7. **LC-HRMS** (Discovery HS C18, ESI/qTOF) calc. for $C_6H_{15}O_3S^+$ $[M + H]^+$: 167.0736 m/z; found 167.0737 m/z. **FT-IR** (neat) [cm^{-1}]: 3407 (br), 2924 (m), 2886 (m), 2826 (w), 1646 (w), 1466 (m), 1441 (w), 1407 (w), 1381 (w), 1287 (w), 1214 (w), 1147 (m), 1105 (m), 1065 (m), 1029 (s), 949 (m), 918 (m). **R**$_f$ (n-Hex/EtOAc, 2/1): 0.13.
Bisprotected Side-Product: ^{1}**H-NMR** (400 MHz, $CDCl_3$) δ 4.65 (s, 4H), 3.72 (t, *J* = 6.6 Hz, 4H), 3.38 (s, 6H), 2.80 (t, *J* = 6.6 Hz, 4H) . ^{13}**C-NMR** (101 MHz, $CDCl_3$) δ 96.5, 67.3, 55.3, 32.2. **LC-HRMS** (Discovery HS C18, ESI/qTOF) calc. for $C_8H_{19}O_4S^+$ $[M + H]^+$: 211.0999 m/z; found 211.0996 m/z. **FT-IR** (neat) [cm^{-1}]: 2989 (w), 2927 (m), 2886 (m), 2824 (w), 1466 (w), 1441 (w), 1406 (w), 1381 (w), 1291 (w), 1214 (m), 1147 (s), 1105 (s), 1066 (s), 1028 (vs), 949 (m), 916 (s), 663 (m), 628 (m). **R**$_f$ (n-Hex/EtOAc, 2/1): 0.46.

Glutamic ester (S7)

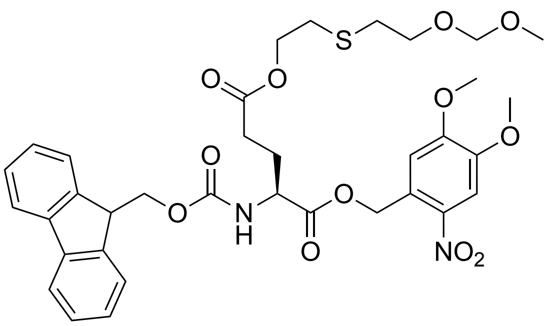

$C_{35}H_{40}N_2O_{12}S$
MW: 712.77
S7

According to **GPB** using **S6** (2.4 mmol). Purification of the crude product via column chromatography (Isolera Sfar HC Duo 25 g, 80 mL min^{-1}, CH_2Cl_2/CH_2Cl_2 + 1.0 % MeOH (A/B), 20 % B (5.0 CV), 20 % – 100 % B (25.0 CV), 100 % B (16.2 CV)) afforded the title compound as a yellow sticky solid (0.93 g, 1.3 mmol, 65 %).
^{1}**H-NMR** (400 MHz, $CDCl_3$) δ 7.76 (d, *J* = 7.6 Hz, 2H), 7.72 (s, 1H), 7.57 (d, *J* = 7.5 Hz, 2H), 7.39 (q, *J* = 7.0 Hz, 3H), 7.30 (q, *J* = 6.8 Hz, 1H), 7.01 (s, 1H), 5.60 (d, *J* = 4.8 Hz, 2H), 5.56 (d, *J* = 8.0 Hz, 1H), 4.63 (s, 2H), 4.50 (td, *J* = 8.5, 4.9 Hz, 1H), 4.39 (dd, *J* = 7.2, 2.0 Hz, 2H), 4.25 (t, *J* = 6.8 Hz, 2H), 4.20 (t, *J* = 7.1 Hz, 1H), 3.96 (s, 3H), 3.95 (s, 4H), 3.69 (t, *J* = 6.6 Hz, 2H), 3.62 (td, *J* = 6.6, 2.0 Hz, 4H), 3.36 (s, 3H), 2.80 (t, *J* = 6.8 Hz, 2H), 2.77 (t, *J* = 6.7 Hz, 2H), 2.74 (t, *J* = 6.7 Hz, 2H), 2.73 (t, *J* = 6.5 Hz, 2H), 2.57 – 2.40 (m, 2H), 2.36 – 2.23 (m, 1H), 2.13 – 2.00 (m, 1H). ^{13}**C-NMR** (101 MHz, $CDCl_3$) δ 172.5, 171.6, 156.1, 153.8, 148.4, 143.7, 141.3, 139.8, 128.1, 127.8, 127.1, 126.5, 125.0, 120.0, 110.3, 108.2, 96.5, 67.3, 64.4, 63.8, 56.6, 56.4, 55.3, 53.6, 47.1, 32.2, 30.9, 30.2, 27.1. **LC-HRMS** (Discovery HS C18, ESI/qTOF) calc. for $C_{35}H_{44}N_3O_{12}S^+$ $[M + NH_4]^+$: 730.2640 m/z; found 730.2655 m/z. **FT-IR** (neat) [cm^{-1}]: 3312 (w), 2943 (w), 2889 (w), 1731 (m), 1716 (m), 1693 (m), 1580 (w), 1522 (s), 1450 (m), 1384 (w), 1327 (m), 1273 (vs), 1220 (s), 1168 (s), 1147 (s), 1104 (m), 1066 (vs), 1031 (s), 986 (m), 915 (m), 875 (m), 796 (m), 757 (m), 739 (s), 696 (w). **R**$_f$ (1 % MeOH in CH_2Cl_2): 0.23.

Glutamic ester (10a)

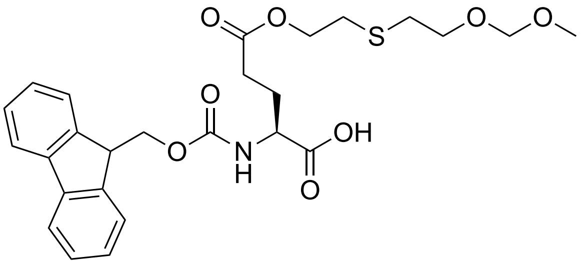

$C_{26}H_{31}NO_8S$
MW: 517.59
10a

According to **GPC** using **S7** (1.0 mmol). Purification via column chromatography (Isolera Sfar HC Duo 25 g, 80 mL min^{-1}, CH_2Cl_2/CH_2Cl_2 + 10 % MeOH (A/B), 10 % B (5.0 CV), 10 % – 20 % B (5.0 CV), 20 % B (8.3 CV)) afforded the title compound as a brownish honey (332 mg, 0.64 mmol, 64 %).
^{1}**H-NMR** (400 MHz, $CDCl_3$) δ 7.76 (d, *J* = 7.5 Hz, 2H), 7.59 (dd, *J* = 7.8, 3.0 Hz, 2H), 7.39 (t, *J* = 7.5 Hz, 2H), 7.31 (tt, *J* = 7.5, 1.1 Hz, 2H), 5.61 (d, *J* = 7.8 Hz, 1H), 4.64 (s, 2H), 4.42 (td, *J* = 11.0, 6.8 Hz, 3H), 4.23 (dt, *J* = 10.9, 6.6 Hz, 3H), 3.71 (t, *J* = 6.5 Hz, 2H), 3.37 (s, 3H), 2.79 (t, *J* = 6.7 Hz, 2H), 2.76 (t, *J* = 6.6 Hz, 2H), 2.58 – 2.37 (m, 1H), 2.18 (dtd, *J* = 72.7, 13.4, 7.3 Hz, 1H). ^{13}**C-NMR** (101 MHz, $CDCl_3$) δ 174.6, 172.8, 156.1, 143.8, 141.3, 127.8, 127.1, 125.1, 120.0, 96.3, 67.2, 67.2, 63.8, 55.4, 53.2, 47.1, 32.0, 30.8, 30.1, 27.2. **LC-HRMS** (Discovery HS C18, ESI/qTOF) calc. for $C_{26}H_{35}N_2O_8S^+$ $[M + NH]^+$: 535.2109 m/z; found 535.2113 m/z. **FT-IR** (neat) [cm^{-1}]: 3329 (br), 2942 (w), 1716 (s), 1522 (m), 1449 (m), 1410 (w), 1325 (w), 1211 (m), 1147 (s), 1105 (m), 1056 (s), 1031 (vs), 760 (m), 740 (vs).

Alcohol (S8)

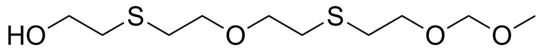

$C_{10}H_{22}O_4S_2$
MW: 270.40
S8

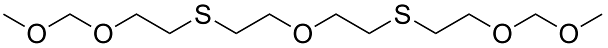

$C_{12}H_{26}O_5S_2$
MW: 314.46
S8'

According to **GPA** using 3,9-dithia-6-oxa-undecane-1,11-diol (20.0 mmol) which was provided in-house. Purification of the crude product via column chromatography (Isolera Sfar HC Duo 50 g, 100 mL min^{-1}, n-Hex/EtOAc, 20 % – 60 % EtOAc (25.0 CV)) afforded the title compound as a colorless liquid (2.75 g, 10.2 mmol, 51 %) and undesired bisprotected side-product **S8'** as a colorless oil (0.88 g, 2.8 mmol, 14 %).
^{1}**H-NMR** (400 MHz, $CDCl_3$) δ 4.65 (s, 2H), 3.79 – 3.68 (m, 4H), 3.66 (td, *J* = 6.5, 1.6 Hz, 4H), 3.38 (s, 3H), 2.83 – 2.71 (m, 8H), 2.53 (s, 1H). ^{13}**C-NMR** (101 MHz, $CDCl_3$) δ 96.5, 70.9, 70.9, 67.3, 60.9, 55.3, 35.9, 32.3, 31.9, 31.5. **LC-HRMS** (Discovery HS C18, ESI/qTOF) calc. for $C_{10}H_{23}O_4S_2^+$ $[M + H]^+$: 271.1032 m/z; found 271.1034 m/z. **FT-IR** (neat) [cm^{-1}]: 3418 (br), 2921 (m), 2874 (m), 1732 (w), 1523 (w), 1464 (w), 1406 (w), 1381 (w), 1358 (w), 1281 (w), 1147 (m), 1102 (s), 1065 (m), 1031 (s), 948 (m), 918 (m). **R**$_f$ (n-Hex/EtOAc, 2/1): 0.08.
Bisprotected Side-Product: ^{1}**H-NMR** (400 MHz, $CDCl_3$) δ 4.64 (s, 4H), 3.71 (t, *J* = 6.6 Hz, 4H), 3.64 (t, *J* = 6.7 Hz, 4H), 3.38 (s, 6H), 2.78 (dt, *J* = 11.0, 6.7 Hz, 8H). ^{13}**C-NMR** (101 MHz, $CDCl_3$) δ 96.5, 70.8, 67.3, 55.3, 32.4, 31.9. **LC-HRMS** (Discovery HS C18, ESI/qTOF) calc. for $C_{12}H_{27}O_5S_2^+$ $[M + H]^+$: 315.1294 m/z; found 315.1294 m/z. **FT-IR** (neat) [cm^{-1}]: 2926 (m), 2883 (m), 2824 (w), 1464 (w), 1441 (w), 1406 (w), 1380 (w), 1358 (w), 1291 (w), 1212 (w), 1147 (m), 1104 (s), 1068 (m), 1029 (s), 949 (w), 916 (m), 631 (m). **R**$_f$ (n-Hex/EtOAc, 2/1): 0.29.

Glutamic ester (S9)

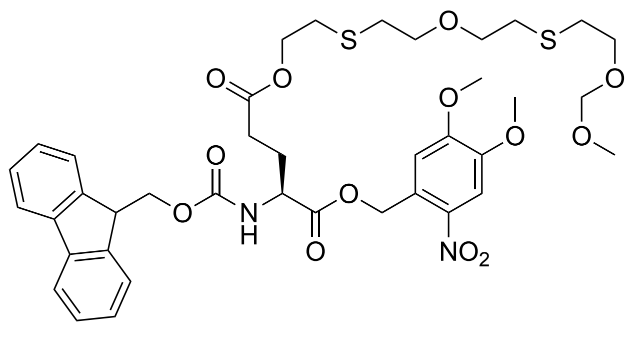

$C_{39}H_{48}N_2O_{13}S_2$
MW: 816.93
S9

According to **GPB** using **S8** (2.4 mmol). Purification of the crude product via column chromatography (Isolera Sfar HC Duo 25 g, 80 mL min^{-1}, CH_2Cl_2/CH_2Cl_2 + 1.0 % MeOH (A/B), 20 % B (5.0 CV), 20 % – 100 % B (25.0 CV), 100 % B (20.0 CV)) afforded the title compound as a yellow sticky solid (1.23 g, 1.5 mmol, 75 %).
^{1}**H-NMR** (400 MHz, $CDCl_3$) δ 7.76 (d, J = 7.6 Hz, 2H), 7.72 (s, 1H), 7.57 (d, J = 7.5 Hz, 2H), 7.39 (q, J = 7.3 Hz, 2H), 7.30 (q, J = 6.7 Hz, 2H), 7.01 (s, 1H), 5.59 (d, J = 4.8 Hz, 2H), 5.56 (d, J = 8.0 Hz, 1H), 4.63 (s, 2H), 4.50 (td, J = 8.2, 4.9 Hz, 1H), 4.39 (dd, J = 7.1, 2.0 Hz, 2H), 4.25 (t, J = 6.8 Hz, 2H), 4.20 (t, J = 7.1 Hz, 1H), 3.96 (s, 3H), 3.95 (s, 3H), 3.69 (t, J = 6.6 Hz, 2H), 3.62 (td, J = 6.6, 2.0 Hz, 4H), 3.36 (s, 3H), 2.80 (t, J = 6.9 Hz, 2H), 2.77 (t, J = 6.6 Hz, 2H), 2.74 (t, J = 6.7 Hz, 2H), 2.73 (t, J = 6.5 Hz, 2H), 2.56 – 2.39 (m, 2H), 2.18 (ddt, J = 92.1, 14.9, 7.6 Hz, 2H). ^{13}**C-NMR** (101 MHz, $CDCl_3$) δ 172.5, 171.6, 156.1, 153.8, 148.4, 143.7, 141.3, 139.7, 128.1, 127.8, 127.1, 126.5, 125.0, 120.0, 110.3, 108.2, 96.5, 70.8, 70.8, 67.3, 67.2, 64.4, 63.8, 56.6, 56.4, 55.3, 53.7, 47.1, 32.3, 31.9, 31.8, 31.0, 30.2, 27.1. **LC-HRMS** (Discovery HS C18, ESI/qTOF) calc. for $C_{39}H_{52}N_3O_{13}S_2^+$ $[M + NH_4]^+$: 834.2936 m/z; found 834.2968 m/z. **FT-IR** (neat) [cm^{-1}]: 3339 (br), 2949 (w), 2861 (w), 1722 (s), 1580 (m), 1522 (vs), 1450 (m), 1386 (w), 1327 (m), 1275 (vs), 1220 (s), 1169 (s), 1148 (m), 1104 (m), 1065 (vs), 1032 (s), 985 (m), 872 (w), 796 (w), 760 (m), 741 (m), 637 (m), 621 (m). **R**$_f$ (1 % MeOH in CH_2Cl_2): 0.12.

Glutamic ester (10c)

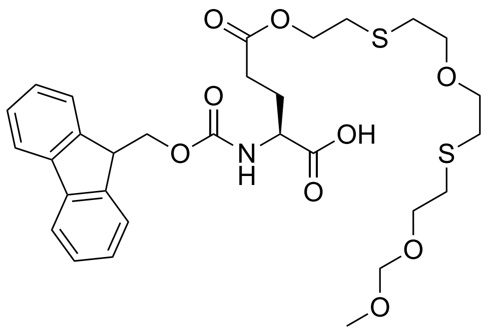

$C_{30}H_{39}NO_9S_2$
MW: 621.76
10c

According to **GPC** using **S9** (1.4 mmol). Purification via column chromatography (Isolera Sfar HC Duo 25 g, 80 mL min^{-1}, CH_2Cl_2/CH_2Cl_2 + 10 % MeOH (A/B), 10 % B (5.0 CV), 10 % – 20 % B (5.0 CV), 20 % B (15.0 CV) to afford the title compound as a brownish honey (543 mg, 0.87 mmol, 60 %).
^{1}**H-NMR** (400 MHz, $CDCl_3$) δ 7.76 (d, J = 7.6 Hz, 1H), 7.60 (d, J = 7.6 Hz, 2H), 7.40 (tt, J = 7.5, 1.3 Hz, 3H), 7.32 (tt, J = 7.5, 1.0 Hz, 2H), 5.60 (d, J = 7.9 Hz, 1H), 4.66 (s, 2H), 4.42 (d, J = 6.9 Hz, 3H), 4.32 – 4.18 (m, 3H), 3.71 (t, J = 6.7 Hz, 2H), 3.63 (td, J = 6.5, 2.3 Hz, 4H), 3.38 (s, 3H), 2.80 (t, J = 6.6 Hz, 2H), 2.78 (t, J = 6.7 Hz, 2H), 2.74 (t, J = 6.8 Hz, 3H), 2.60 – 2.36 (m, 2H), 2.18 (dtd, J = 77.5, 13.6, 6.6 Hz, 2H). ^{13}**C-NMR** (101 MHz, $CDCl_3$) δ 174.0, 172.7, 156.1, 143.8, 141.3, 127.8, 127.1, 125.1, 120.0, 96.3, 70.9, 70.9, 67.3, 67.1, 64.0, 55.3, 53.1, 47.2, 32.2, 31.8, 31.8, 31.0, 30.2, 27.3. **LC-HRMS** (Discovery HS C18, ESI/qTOF) calc. for $C_{30}H_{43}N_2O_9S_2^+$ $[M + NH_4]^+$: 639.2405 m/z; found 639.2416 m/z. **FT-IR** (neat) [cm^{-1}]: 3321 (br), 2944 (w), 1721 (s), 1520 (m), 1450 (m), 1403 (w), 1205 (m), 1168 (m), 1147 (s), 1104 (vs), 1065 (s), 1031 (vs), 760 (m), 740 (vs). **R**$_f$ (5 % MeOH in CH_2Cl_2): 0.08.

Cysteine Derivative (11)

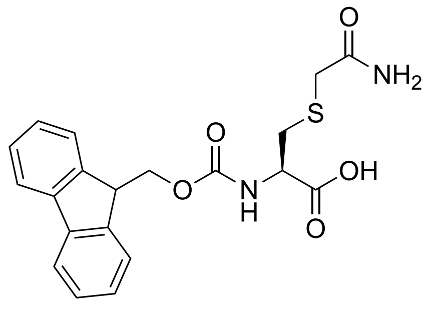

$C_{20}H_{20}N_2O_5S$
MW: 400.45
11

A round-bottomed flask equipped with a stir bar was charged with *L*-Cys (15.0 mmol, 1.5 equiv) and sat. $NaHCO_3$ (20 mL). To the solution 2-chloroacetamide (15.0 mmol, 1.5 equiv) was added in one portion and the resulting reaction mixture stirred at 25 °C for 1.5 h, monitored by TLC. Then, a solution of Fmoc-OSu (10.0 mmol, 1.0 equiv) in acetone (40 mL) was added and stirring continued for 2 h, monitored by TLC. The pH was adjusted to 9 with 10 % NaOH if necessary. The acetone was removed under reduced pressure and the formed slurry filtered through a sintered glass funnel. The collected solids were washed with ice-cold acetone and subsequently dried under high vacuum to afford the title compound as a white solid (3.67 g, 9.18 mmol, 91 %).
^{1}H-NMR (400 MHz, DMSO-d^6) δ 12.85 (s, 1H), 7.89 (d, *J* = 7.5 Hz, 2H), 7.83 (d, *J* = 8.3 Hz, 1H), 7.73 (d, *J* = 7.5 Hz, 2H), 7.46 (s, 1H), 7.37 (dtd, *J* = 35.6, 7.4, 1.1 Hz, 4H), 7.06 (s, 1H), 4.32 – 4.15 (m, 4H), 3.19 – 3.07 (m, 2H), 3.03 (dd, *J* = 13.6, 4.6 Hz, 1H), 2.83 (dd, *J* = 13.6, 9.7 Hz, 1H). **^{13}C-NMR** (101 MHz, DMSO-d^6) δ 172.3, 170.9, 156.0, 143.8, 140.7, 127.7, 127.1, 125.3, 120.1, 65.8, 53.6, 46.6, 34.6, 33.3, 30.7. **LC-HRMS** (Discovery HS C18, ESI/qTOF) calc. for $C_{20}H_{21}N_2O_5S^+$ $[M + H]^+$: 401.1166 m/z; found 401.1164 m/z. **FT-IR** (neat) [cm^{-1}]: 3401 (m), 3314 (m), 1917 (w), 1732 (s), 1680 (m), 1632 (m), 1529 (vs), 1450 (m), 1407 (m), 1293 (s), 1225 (vs), 1149 (w), 1081 (m), 1035 (s), 938 (w), 919 (w), 893 (w), 759 (m), 743 (s), 687 (m), 641 (s). **mp**: 198 °C. NMR data match commercially available product.

Peptide (2a)

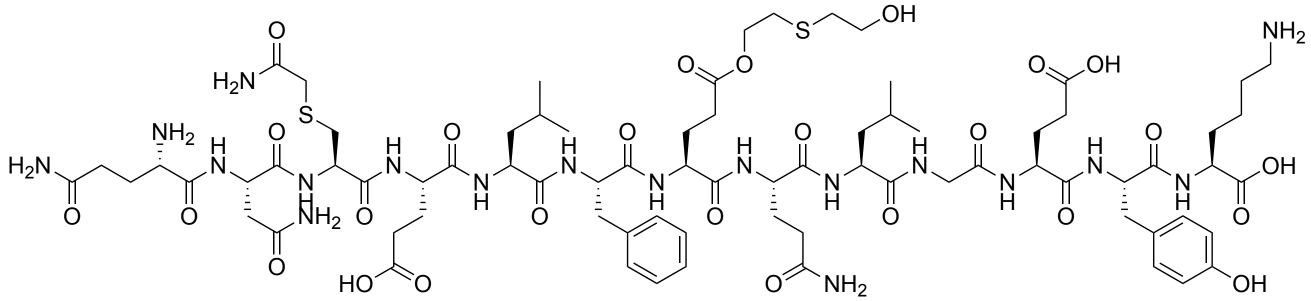

$C_{76}H_{116}N_{18}O_{26}S_2$
MW: 1761.98
2a

Resin:	H-*L*-Lys(Boc)-2CT
Scale:	0.075 mmol
Vial:	10 mL
Equivalents:	4.0 equiv
Amino Acids:	Fmoc-Gln(Trt)-OH, Fmoc-Asn(Trt)-OH, Fmoc-Glu(^{t}Bu)-OH, Fmoc-Leu-OH, Fmoc-Gly-OH and Fmoc-Tyr(^{t}Bu)-OH (all 0.5 M in DMF); Fmoc-Phe-OH and **10a** (both 0.5 M in NMP); **11** (0.5 M in DMSO)
Coupling Agents:	DIC and Oxyma (both 0.2 M in DMF), 75 °C for 5 min
Fmoc Deprotection:	5 % Piperazine in DMF (2 × 10 equiv)
Resin Cleavage:	TFA/TIPS/CH_2Cl_2 (20/5/75, v/v/v)

Peptide-synthesis according to **GPSPPS-A** using parameters as indicated above. The resin was transferred into a sintered glass funnel and treated with cleavage cocktail (3 × 10 mL) and liquid phase allowed to elute into a round-bottomed flask equipped with a stir bar. The collected filtrate was stirred at 25 °C for 2 h. The reaction mixture was concentrated to around 2 mL total volume and precipitated in ice-cold Et_2O. After sonication and centrifugation (4000 rpm, 5 min, ambient temperature), the liquid phase was decanted and the residue resuspended in ice-cold Et_2O and again centrifuged. The washing procedure was performed three times in total. Then, the residue was picked up in HPLC grade H_2O and lyophilized to afford the title compound as a white fluffy solid (96 mg, Peptide Purity: 55 %). Around 10 mg of crude product was further purified by HPLC (H_2O + 0.1 %

FoA (A)/CH_3CN + 0.1 % FoA (B), 5 % – 50 % B in 10 min, 50 % – 95 % B in 4 min, 95 % B for 2 min)
^{1}**H-NMR** (400 MHz, D_2O) δ . ^{13}**C-NMR** (101 MHz, D_2O) δ . **LC-HRMS** (Aurora Elite C18, CaptiveSpray/timsTOF) Parent Ion: calc. for $C_{76}H_{118}N_{18}O_{26}S_2^{2+}$ $[M+2H]^{2+}$: 881.3948 m/z; found 881.3992 m/z with fragment ions (dda-PASEF, 39.6 eV, in m/z) 243.1089 (b2), 310.1788 (y2), 403.1384 (b3), 439.2205 (y3), 496.2416 (y4), 532.1797 (b4), 609.3263 (y5), 645.2671 (b5), 737.383 (y6), 792.3386 (b6), 970.4562 (y7), 1025.4113 (b7), 1117.5234 (y8), 1153.4653 (b8), 1230.607 (y9), 1266.5545 (b9), 1359.6581 (y10), 1519.6776 (y11).

Peptide (2b)

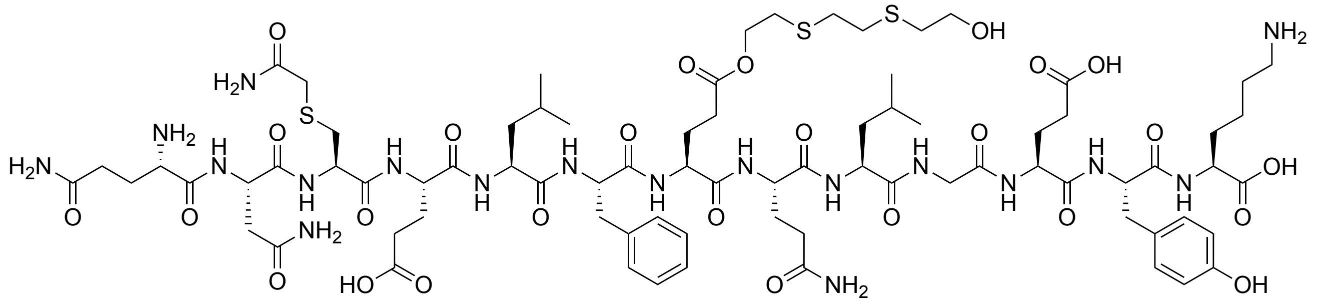

$C_{78}H_{120}N_{18}O_{26}S_3$
MW: 1822.10

2b

Resin:	H-*L*-Lys(Boc)-2CT
Scale:	0.075 mmol
Vial:	10 mL
Equivalents:	4.0 equiv
Amino Acids:	Fmoc-Gln(Trt)-OH, Fmoc-Asn(Trt)-OH, Fmoc-Glu(^{t}Bu)-OH, Fmoc-Leu-OH, Fmoc-Gly-OH and Fmoc-Tyr(^{t}Bu)-OH (all 0.5 M in DMF); Fmoc-Phe-OH and **10b** (both 0.5 M in NMP); **11** (0.5 M in DMSO)
Coupling Agents:	DIC and Oxyma (both 0.2 M in DMF), 75 °C for 5 min
Fmoc Deprotection:	5 % Piperazine in DMF (2 × 10 equiv)
Resin Cleavage:	TFA/TIPS/CH_2Cl_2 (20/5/75, v/v/v)

Peptide-synthesis according to **GPSPPS-A** using parameters as indicated above. The resin was transferred into a sintered glass funnel and treated with cleavage cocktail (3 × 10 mL) and liquid phase allowed to elute into a round-bottomed flask equipped with a stir bar. The collected filtrate was stirred at 25 °C for 2 h. The reaction mixture was concentrated to around 2 mL total volume and precipitated in ice-cold Et_2O. After sonication and centrifugation (4000 rpm, 5 min, ambient temperature), the liquid phase was decanted and the residue resuspended in ice-cold Et_2O and again centrifuged. The washing procedure was performed three times in total. Then, the residue was picked up in HPLC grade H_2O and lyophilized to afford the title compound as a white fluffy solid (108 mg, Peptide Purity: 46 %). Around 10 mg of crude product was further purified by HPLC (H_2O + 0.1 % FoA (A)/CH_3CN + 0.1 % FoA (B), 5 % – 50 % B in 10 min, 50 % – 95 % B in 4 min, 95 % B for 2 min).
LC-HRMS (Aurora Elite C18, CaptiveSpray/timsTOF) calc. for $C_{78}H_{122}N_{18}O_{26}S_3^{2+}$ $[M + 2H]^{2+}$: 911.3964 m/z; found 911.4005 m/z with fragment ions (dda-PASEF, 40.4 eV, in m/z) 243.1113 (b2), 310.1788 (y2), 403.1384 (b3), 439.2172 (y3), 496.238 (y4), 532.1797 (b4), 609.3224 (y5), 645.263 (b5), 737.383 (y6), 792.3341 (b6), 1030.4574 (y7), 1085.4019 (b7), 1177.5258 (y8), 1213.4822 (b8), 1290.6082 (y9), 1419.6573 (y10), 1579.6856 (y11).

Peptide (2c)

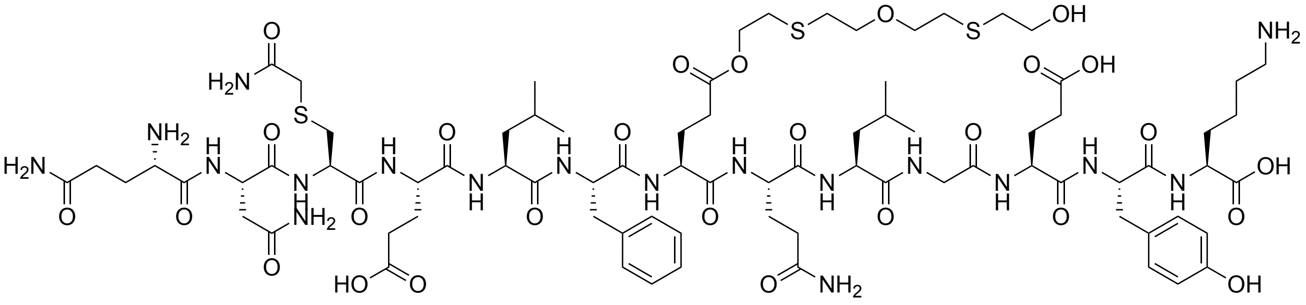

$C_{80}H_{124}N_{18}O_{27}S_3$
MW: 1866.15
2c

Resin:	H-*L*-Lys(Boc)-2CT
Scale:	0.075 mmol
Vial:	10 mL
Equivalents:	4.0 equiv
Amino Acids:	Fmoc-Gln(Trt)-OH, Fmoc-Asn(Trt)-OH, Fmoc-Glu(^{t}Bu)-OH, Fmoc-Leu-OH, Fmoc-Gly-OH and Fmoc-Tyr(^{t}Bu)-OH (all 0.5 M in DMF); Fmoc-Phe-OH and **10c** (both 0.5 M in NMP); **11** (0.5 M in DMSO)
Coupling Agents:	DIC and Oxyma (both 0.2 M in DMF), 75 °C for 5 min
Fmoc Deprotection:	5 % Piperazine in DMF (2 × 10 equiv)
Resin Cleavage:	TFA/TIPS/CH_2Cl_2 (20/5/75, v/v/v)

Peptide-synthesis according to **GPSPPS-A** using parameters as indicated above. The resin was transferred into a sintered glass funnel and treated with cleavage cocktail (3 × 10 mL) and liquid phase allowed to elute into a round-bottomed flask equipped with a stir bar. The collected filtrate was stirred at 25 °C for 2 h. The reaction mixture was concentrated to around 2 mL total volume and precipitated in ice-cold Et_2O. After sonication and centrifugation (4000 rpm, 5 min, ambient temperature), the liquid phase was decanted and the residue resuspended in ice-cold Et_2O and again centrifuged. The washing procedure was performed three times in total. Then, the residue was picked up in HPLC grade H_2O and lyophilized to afford the title compound as a white fluffy solid (111 mg, Peptide Purity: 54 %). Around 10 mg of crude product was further purified by HPLC (H_2O + 0.1 % FoA (A)/CH_3CN + 0.1 % FoA (B), 5 % – 50 % B in 10 min, 50 % – 95 % B in 4 min, 95 % B for 2 min).
LC-HRMS (Aurora Elite C18, CaptiveSpray/timsTOF) calc. for $C_{80}H_{126}N_{18}O_{27}S_3^{2+}$ $[M + 2H]^{2+}$: 933.4095 m/z; found 933.4140 m/z with fragment ions (dda-PASEF, 40.7 eV, in m/z) 243.1138 (b2), 310.1788 (y2), 403.1384 (b3), 439.2172 (y3), 496.238 (y4), 532.1797 (b4), 609.3224 (y5), 645.2631 (b5), 737.383 (y6), 792.3341 (b6), 1074.4863 (y7), 1221.5601 (y8), 1334.6441 (y9), 1623.7074 (y11).

Aspartic ester (S10)

A heat-gun dried two-necked round-bottomed flask equipped with a stir bar and septum was charged with Fmoc-Asp(^{t}Bu)-OH (**12**) (11.0 mmol, 1.1 equiv), NVOH (10.0 mmol, 1.0 equiv), DMAP (2.0 mmol, 0.2 equiv) and THF (10 mL) and the resulting reaction mixture cooled to 0 °C. To the resulting suspension, DIC (11.0 mmol, 1.1 equiv) was added dropwise and the resulting reaction mixture allowed to warm up to 25 °C and stirred for 20 h. The resulting orange slurry was filtered through cotton with the aid of THF and the filtrate concentrated under reduced pressure. Purification via flash column chromatography (Isolera Sfar HC Duo 50 g, 120 mL min^{-1}, n-Hex/Et_2O, 20 % Et_2O (5.0 CV), 20 % – 70 % Et_2O (15.0 CV), 70 % Et_2O (11.2 CV)), concentration under reduced pressure and subsequent drying under high vacuum afforded the title compound as a yellowish spongy solid

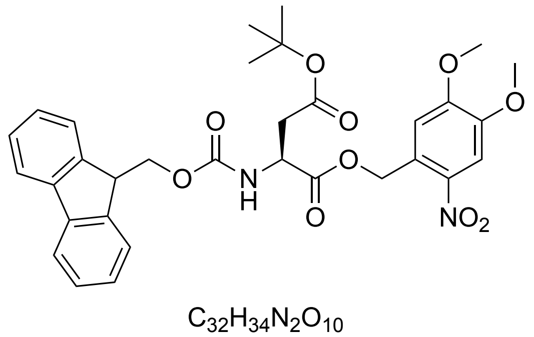

$C_{32}H_{34}N_2O_{10}$
MW: 606.63
S10

(4.02 g, 6.6 mmol, 66 %).
^{1}**H-NMR** (400 MHz, $CDCl_3$) δ 7.74 (dd, *J* = 7.6, 3.9 Hz, 2H), 7.71 (s, 1H), 7.57 (dd, *J* = 7.7, 2.8 Hz, 2H), 7.38 (td, *J* = 7.6, 2.9 Hz, 2H), 7.27 (td, *J* = 7.5, 1.3 Hz, 2H), 7.02 (s, 1H), 5.83 (d, *J* = 9.0 Hz, 1H), 5.62 (s, 2H), 4.72 (dt, *J* = 9.1, 4.7 Hz, 1H), 4.47 – 4.36 (m, 1H), 4.49 – 4.33 (m, 1H), 4.21 (t, *J* = 7.0 Hz, 1H), 3.95 (s, 3H), 3.94 (s, 3H), 2.90 (ddd, *J* = 84.4, 17.0, 4.7 Hz, 2H), 1.43 (s, 9H). ^{13}**C-NMR** (101 MHz, $CDCl_3$) δ 170.6, 170.2, 156.1, 153.8, 148.2, 143.7, 141.3, 139.6, 127.8, 127.8, 127.1, 126.8, 125.0, 120.0, 110.1, 108.1, 82.2, 67.2, 64.3, 56.6, 56.4, 50.7, 47.1, 37.5, 28.0. **LC-HRMS** (Discovery HS C18, ESI/qTOF) calc. for $C_{32}H_{35}N_2O_{10}^+$ [M+H]$^+$: 607.2286 m/z; found 607.2298 m/z. **FT-IR** (neat) [cm^{-1}]: 2939 (w), 1718 (s), 1582 (w), 1520 (s), 1450 (m), 1367 (m), 1327 (m), 1275 (vs), 1220 (s), 1148 (vs), 1065 (vs), 1033 (m), 985 (m), 870 (m), 846 (w), 796 (m), 759 (m), 740 (s). $\mathbf{R}_f$ (n-Hex/Et_2O, 1/1): 0.21.

Aspartic acid derivative (S11)

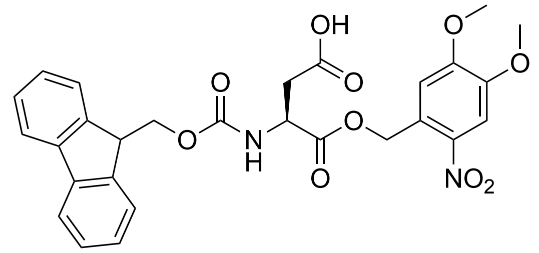

$C_{28}H_{26}N_2O_{10}$
MW: 550.52
S11

In a round-bottomed flask equipped with a stir bar, **S10** (6.6 mmol) was treated with TFA (30 % in CH_2Cl_2, 20 mL) at 25 °C for 1 h. After removal of the volatiles under reduced pressure and co-evaporation with CH_2Cl_2 and acetone subsequently, the product was recrystallized from acetone, filtered and washed with ice-cold acetone. The collected solid was dried under continuous airflow and then ambient conditions overnight to afford the title compound as a pale yellow solid (3.45 g, 6.2 mmol, 62 % over two steps).
^{1}**H-NMR** (400 MHz, $CDCl_3$+2 % DMSO-*d*^{6}) δ 7.78 – 7.71 (m, 2H), 7.69 (s, 1H), 7.62 – 7.55 (m, 2H), 7.42 – 7.33 (m, 2H), 7.32 – 7.24 (m, 2H), 7.02 (s, 1H), 6.10 (d, *J* = 9.0 Hz, 1H), 5.69 – 5.53 (m, 2H), 4.75 (dt, *J* = 9.1, 4.6 Hz, 1H), 4.46 – 4.35 (m, 1H), 4.47 – 4.32 (m, 1H), 4.20 (t, *J* = 7.0 Hz, 1H), 3.93 (s, 6H), 2.99 (ddd, *J* = 96.0, 17.4, 4.7 Hz, 2H). ^{13}**C-NMR** (101 MHz, $CDCl_3$+2 % DMSO-*d*^{6}) δ 173.0, 170.7, 156.2, 153.8, 148.1, 143.7, 141.2, 139.4, 127.7, 127.1, 125.1, 120.0, 109.9, 108.1, 67.2, 64.3, 56.7, 56.4, 50.6, 47.1, 36.2. **LC-HRMS** (Discovery HS C18, ESI/qTOF) calc. for $C_{28}H_{27}N_2O_{10}^+$ [M + H]$^+$: 551.1660 m/z; found 551.1662 m/z. **FT-IR** (neat) [cm^{-1}]: 3308 (m), 3063 (w), 2944 (w), 1732 (m), 1703 (s), 1682 (m), 1583 (w), 1542 (m), 1523 (s), 1450 (w), 1387 (w), 1325 (m), 1274 (vs), 1217 (s), 1175 (m), 1065 (m), 1035 (m), 1009 (m), 875 (m), 796 (m), 759 (m), 737 (s), 637 (m). **mp:** 172 °C.

Aspartic ester S12

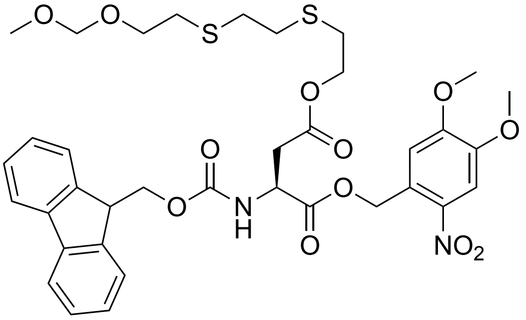

$C_{36}H_{42}N_2O_{12}S_2$
MW: 758.85
S12

According to **GPB** using **6** (2.4 mmol) and **S11** and stirring of the reaction mixture for 24 h. Purification of the crude product via column chromatography (Isolera Sfar HC Duo 25 g, 80 mL min^{-1}, CH_2Cl_2/CH_2Cl_2 + 1.0 % MeOH (A/B), 10 % B (2.0 CV), 10 % – 40 % B (7.0 CV), 40 % B (10.0 CV), 40 % – 100 % B (5.0 CV), 100 % B (12.2 CV)) afforded slightly impure fractions. Purification of these using the same gradient as above and recombination with the previous fractions afforded the title compound as a yellow sticky oil (616 mg, 0.8 mmol, 40 %).
^{1}**H-NMR** (400 MHz, $CDCl_3$) δ 7.75 (dd, *J* = 7.6, 2.6 Hz, 2H), 7.71 (s, 1H), 7.58 (dd, *J* = 7.4, 2.6 Hz, 1H), 7.39 (tt, *J* = 7.7, 1.4 Hz, 2H), 7.28 (td, *J* = 7.5, 1.2 Hz, 2H), 6.99 (s, 1H), 5.92 (d, *J* = 8.9 Hz, 1H), 5.68 – 5.54 (m, 2H), 4.77 (dt, *J* = 9.1, 4.7 Hz, 1H), 4.62 (s, 2H), 4.47 – 4.37 (m, 1H), 4.50 – 4.34 (m, 1H), 4.32 – 4.23 (m, 2H), 4.20 (t, *J* = 7.0 Hz, 1H), 3.95 (s, 3H), 3.94 (s, 3H), 3.70 (t, *J* = 6.5 Hz, 2H), 3.36 (s, 3H), 3.01 (ddd, *J* = 82.3, 17.2, 4.7 Hz, 2H), 2.80 – 2.71 (m, 8H). ^{13}**C-NMR** (101 MHz, $CDCl_3$) δ 170.6, 170.3, 156.1, 153.7, 148.4, 143.6, 141.3, 128.1, 127.8, 127.1, 126.4, 125.0, 120.0, 110.5, 108.2, 96.5, 67.3, 67.3, 64.6, 63.9, 56.6, 56.4, 55.4, 50.5, 47.1, 36.4, 32.5, 32.3, 32.0, 30.5. **LC-HRMS** (Discovery HS C18, ESI/qTOF) calc. for $C_{36}H_{46}N_3O_{12}S_2^+$ [M + NH_4]$^+$: 776.2517 m/z; found 776.2552 m/z. **FT-IR** (neat) [cm^{-1}]: 3358 (w), 2944 (w), 1728 (s), 1577 (m), 1522 (s), 1437 (m), 1381 (m), 1325 (m), 1275 (s), 1218 (vs), 1178 (m), 1147 (s), 1105 (m), 1064 (vs), 1031 (s), 981 (m), 919 (m), 883 (m), 796 (m), 756 (m), 737 (s). $\mathbf{R}_f$ (DCM+1 % MeOH): 0.17.

Aspartic ester 13

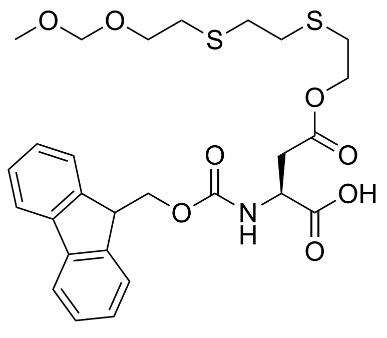

$C_{27}H_{33}NO_8S_2$
MW: 563.68
13

According to **GPC** using **S12** (1.05 mmol). Purification via column chromatography (Isolera Sfar HC Duo 50 g, 100 mL min^{-1}, CH_2Cl_2/CH_2Cl_2 + 10 % MeOH (A/B), 10 % B (10.0 CV), 10 % – 15 % B (10.0 CV), 15 % B (20.0 CV)) afforded the title compound as a brownish honey (269 mg, 0.48 mmol, 45 %).
^{1}**H-NMR** (400 MHz, $CDCl_3$) δ 7.76 (d, *J* = 7.5 Hz, 2H), 7.60 (dd, *J* = 7.6, 2.3 Hz, 2H), 7.40 (t, *J* = 7.5 Hz, 2H), 7.32 (td, *J* = 7.5, 1.2 Hz, 2H), 5.95 (d, *J* = 8.3 Hz, 1H), 4.75 – 4.63 (m, 3H), 4.48 – 4.34 (m, 3H), 4.27 – 4.15 (m, 2H), 3.73 (t, *J* = 6.7 Hz, 2H), 3.41 (s, 3H), 3.02 (ddd, *J* = 84.2, 17.2, 4.5 Hz, 2H), 2.86 – 2.68 (m, 8H). ^{13}**C-NMR** (101 MHz, $CDCl_3$) δ 172.9, 170.8, 156.1, 143.8, 143.7, 141.3, 127.8, 127.1, 125.1, 125.1, 120.0, 96.3, 67.3, 67.3, 63.4, 55.4, 50.1, 47.1, 36.6, 32.0, 31.9, 31.5, 30.4. **LC-HRMS** (Discovery HS C18, ESI/qTOF) calc. for $C_{27}H_{37}N_2O_8S_2^+$ [M + NH_4]$^+$: 581.1986 m/z; found 581.1990 m/z. **FT-IR** (neat) [cm^{-1}]: 2943 (w), 1723 (vs), 1514 (m), 1450 (m), 1390 (w), 1327 (w), 1191 (s), 1147 (s), 1105 (m), 1062 (s), 1031 (vs), 760 (m), 740 (s).

Peptide (3)

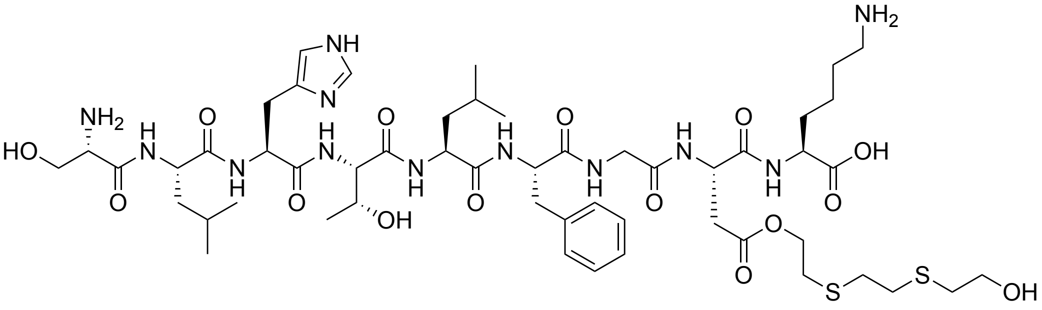

$C_{52}H_{84}N_{12}O_{15}S_2$
MW: 1181.43
3

Resin:	H-*L*-Arg(Pbf)-2CT
Scale:	0.075 mmol
Vial:	10 mL
Equivalents:	4.0 equiv
Amino Acids:	Fmoc-Gly-OH, Fmoc-Leu-OH, Fmoc-Thr(Trt)-OH, Fmoc-His(Trt)-OH, Fmoc-Ser(Trt)-OH (all 0.5 M in DMF); Fmoc-Phe-OH and **13** (both 0.5 M in NMP)
Coupling Agents:	DIC and Oxyma (both 0.2 M in DMF), 75 °C for 5 min
Fmoc Deprotection:	5 % Piperazine in DMF (2 × 10 equiv)
Resin Cleavage:	TFA/TIPS/CH_2Cl_2 (20/5/75, v/v/v)

Peptide-synthesis according to **GPSPPS-A** using parameters as indicated above. The resin was transferred into a sintered glass funnel and treated with cleavage cocktail (3 × 10 mL) and liquid phase allowed to elute into a round-bottomed flask equipped with a stir bar. The collected filtrate was stirred at 25 °C for 2 h. The reaction mixture was concentrated to around 2 mL total volume and precipitated in ice-cold Et_2O. After sonication and centrifugation (4000 rpm, 5 min, ambient temperature), the liquid phase was decanted and the residue resuspended in ice-cold Et_2O and again centrifuged. The washing procedure was performed three times in total. Then, the residue was picked up in HPLC grade H_2O and lyophilized to afford the title compound as a white fluffy solid (69 mg, Peptide Purity: 41 %).
LC-HRMS (Aurora Elite C18, CaptiveSpray/timsTOF) calc. for $C_{52}H_{86}N_{12}O_{15}S_2^{2+}$ [M + 2 H]$^{2+}$: 591.2883 m/z; found 591.2868 m/z with fragment ions (dda-PASEF, 32.4 eV, in m/z) 338.1805 (b3), 439.2305 (b4), 483.1921 (y3), 552.3144 (b5), 630.2621 (y4), 699.3813 (b6), 743.3455 (y5), 756.4049 (b7), 844.3938 (y6), 981.4545 (y7).

1.3.3 Synthesis of Q-Adduct of Histidine

1.3.3.1 Preparation and Characterization of Substrates

General Procedure Methyl Ester Hydrolysis (GPE)

Adapted from literature procedure[24]. A round-bottomed flask equipped with a stir bar was charged with the corresponding methyl ester and CH_3CN (10 mL per g starting material) and HPLC grade H_2O (2 vol%). DIPEA (3.0 equiv) and LiBr (10.0 equiv) was added, the suspension sonicated for a few seconds and then stirred at ambient conditions for 2 h. The reaction was quenched with 10 % NH_4Cl (10 mL) and EtOAc (25 mL) and the phases separated. The pH of the aqueous layer was adjusted to 3-4 with 1 M HCl and extracted with EtOAc (2 × 25 mL). The combined organic layers were washed with brine (25 mL), dried over Na_2SO_4 and concentrated under reduced pressure.

Fmoc-His-OMe (S13)

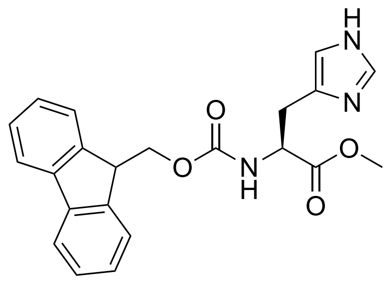

$C_{22}H_{21}N_3O_4$
MW: 391.43
S13

Adapted from literature procedure[27]. In a heat-gun dried, two-necked round-bottomed flask equipped with a stir bar and a dropping funnel, Fmoc-His(Trt)-OH (10 mmol, 1.0 equiv) was suspended in MeOH (50 mL) and AcCl (35.0 mmol, 3.5 equiv) was added dropwise at 0 °C. The resulting solution was allowed to warm up to room temperature and stirred overnight. The reaction mixture was concentrated under reduced pressure and the residue redissolved in DCM, dried over Na_2SO_4, filtered and concentrated under reduced pressure. Purification via column chromatography (Isolera Sfar HC Duo 50 g, 100 mL min^{-1}, CH_2Cl_2/CH_2Cl_2 + 10 % MeOH (A/B), 10 % B (10.0 CV), 10 % – 15 % B (10.0 CV), 15 % B (20.0 CV)) afforded a yellowish amorphous solid (5.1 g). The solid was suspended in DCM (60 mL) and TIPS (1.2 mL) followed by TFA (5.5 mL) was added at 0 °C upon stirring. The resulting solution was stirred at 23 °C overnight. After removal of all volatiles and co-evaporation with CH_2Cl_2, the residue was picked up with EtOAc (50 mL) and washed with sat. $NaHCO_3$ (3 × 25 mL) and brine (25 mL), dried over Na_2SO_4 and concentrated under reduced pressure by around half the volume at 50 °C. The resulting blurry solution was allowed to cool to room temperature upon vigorous stirring leading to formation of a white precipitate. The slurry was stored in the freezer for 2 h, filtered and the collected solids washed with n-Hex and dried at reduced pressure and 70 °C ovenright to afford the title compound as a white solid (2.0 g, 5.3 mmol, 53 %; positive Pauly Test).
^{1}**H-NMR** (400 MHz, CD_3CN) δ 9.12 (s, 1H), 7.75 (d, *J* = 7.6 Hz, 2H), 7.64 – 7.53 (m, 3H), 7.39 (t, *J* = 7.5 Hz, 2H), 7.29 (td, *J* = 7.5, 1.2 Hz, 2H), 6.79 (s, 1H), 6.24 (d, *J* = 8.0 Hz, 1H), 4.62 (q, *J* = 6.1 Hz, 1H), 4.37 (d, *J* = 7.2 Hz, 2H), 4.22 (t, *J* = 7.2 Hz, 1H), 3.70 (s, 3H), 3.14 (d, *J* = 5.3 Hz, 2H). ^{13}**C-NMR** (101 MHz, CD_3CN) δ 172.1, 156.1, 143.9, 143.8, 141.3, 141.3, 135.1, 134.2, 127.7, 127.1, 125.2, 120.0, 115.9, 67.1, 54.0, 52.5, 47.2, 29.5. Spectral data matches literature.

Histidine Derivatives (14a) and (14b)

A heat-gun dried, two-necked, round-bottomed flask equipped with a stir bar and a septum, was charged with **6** (2.0 mmol, 1.0 equiv), CH_2Cl_2 and DIPEA (6.6 mmol, 2.2 equiv) and the solution cooled to 0 °C. At this temperature, Ms_2O (2.4 mmol, 1.2 equiv was added in on one portion, the suspension stirred until homogenous, allowed to warm up to room temperature and stirred for 15 min.
Then, the reaction mixture was transferred to a dropping funnel attached to a heat-gun dried, two-necked, round-bottomed flask equipped with a stir bar and reflux condenser. The mixture was then added dropwise to a solution of **S13** (3.0 mmol, 1.5 equiv) in THF (8 mL) at 50 °C over 20 min. the resulting reaction mixture was then stirred at the given temperature for 3 h.
The reaction was quenched with EtOAc (25 mL) and sat. $NaHCO_3$ (10 mL) and the phases separated. The organic layer was washed with $NaHCO_3$ (2 × 10 mL) and brine (2 × 15 mL), dried over Na_2SO_4, filtered and concentrated under reduced pressure. The crude product was purified by column chromatography (Isolera Sfar HC Duo 25 g,80 mL min^{-1}, CH_2Cl_2/CH_2Cl_2 + 10.0 % MeOH (A/B), 20 % B (8.0 CV), 20 % – 40 % B (10.0 CV), 40 % – 100 % B (10.0 CV), 100 % B (23.3 CV)) to afford **14a** (228 mg, 0.38 mmol, 19 %) and **14a** (245 mg, 0.41 mmol, 20 %), both as colorless honeys (rsm: 53 %).

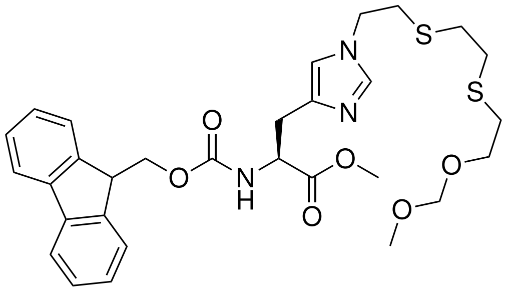

$C_{30}H_{37}N_3O_6S_2$
MW: 599.76
14a

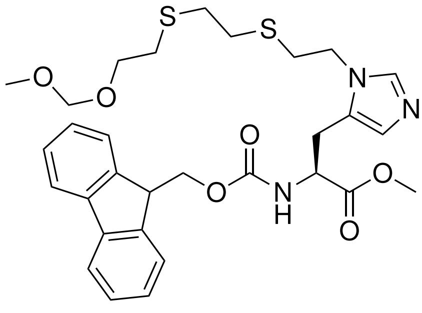

$C_{30}H_{37}N_3O_6S_2$
MW: 599.76
14b

14a: ^{1}**H-NMR** (400 MHz, $CDCl_3$) δ 7.76 (dt, J = 7.6, 0.9 Hz, 2H), 7.62 (t, J = 7.9 Hz, 2H), 7.46 (s, 1H), 7.40 (td, J = 7.6, 1.1 Hz, 2H), 7.31 (td, J = 7.4, 1.2 Hz, 2H), 6.72 (s, 1H), 6.36 (d, J = 8.1 Hz, 1H), 4.66 – 4.60 (m, 3H), 4.36 (dd, J = 7.3, 4.1 Hz, 2H), 4.25 (t, J = 7.3 Hz, 1H), 4.06 (t, J = 6.8 Hz, 2H), 3.73 (s, 3H), 3.69 (t, J = 6.4 Hz, 2H), 3.36 (s, 3H), 3.19 – 3.03 (m, 2H), 2.85 (t, J = 6.8 Hz, 2H), 2.76 – 2.70 (m, 4H), 2.65 – 2.59 (m, 2H). ^{13}**C-NMR** (101 MHz, $CDCl_3$) δ 172.2, 156.1, 144.1, 144.0, 141.3, 137.9, 137.0, 127.7, 127.1, 125.3, 125.3, 119.9, 116.5, 96.5, 67.3, 67.1, 55.4, 54.0, 52.4, 47.2, 33.4, 32.6, 32.5, 32.1 **LC-HRMS** (Discovery HS C18, ESI/qTOF) calc. for $C_{30}H_{38}N_3O_6S_2^+$ [M + H]$^+$: 600.2197 m/z; found 600.2209 m/z. **FT-IR** (neat) [cm^{-1}]: 2949 (m), 2924 (m), 2824 (w), 1722 (m), 1596 (w), 1500 (m), 1447 (m), 1204 (m), 1147 (s), 1105 (s), 1066 (s), 1028 (vs), 916 (m), 729 (vs), 620 (m).
14b: ^{1}**H-NMR** (400 MHz, $CDCl_3$) δ 7.77 (dt, J = 7.6, 1.0 Hz, 2H), 7.57 (dt, J = 7.5, 1.1 Hz, 2H), 7.51 (s, 1H), 7.41 (tt, J = 7.5, 1.0 Hz, 2H), 7.32 (td, J = 7.4, 1.1 Hz, 2H), 6.80 (s, 1H), 5.51 (d, J = 7.8 Hz, 1H), 4.65 – 4.54 (m, 3H), 4.42 (d, J = 7.0 Hz, 2H), 4.21 (t, J = 6.8 Hz, 1H), 4.10 – 3.99 (m, 2H), 3.76 (s, 3H), 3.68 (t, J = 6.5 Hz, 2H), 3.35 (s, 3H), 3.21 – 3.05 (m, 2H), 2.83 (t, J = 6.9 Hz, 2H), 2.75 – 2.68 (m, 4H), 2.64 – 2.58 (m, 2H). ^{13}**C-NMR** (101 MHz, $CDCl_3$) δ 171.4, 155.7, 143.7, 141.4, 137.8, 128.4, 127.8, 127.1, 125.7, 125.0, 120.1, 96.5, 67.3, 67.2, 55.4, 53.6, 52.8, 47.1, 44.9, 33.2, 32.5, 32.5, 32.0, 27.0. **LC-HRMS** (Discovery HS C18, ESI/qTOF) calc. for $C_{30}H_{38}N_3O_6S_2^+$ [M + H]$^+$: 600.2197 m/z; found 600.2210 m/z. **FT-IR** (neat) [cm^{-1}]: 2947 (m), 2924 (m), 1715 (s), 1532 (w), 1496 (m), 1449 (m), 1347 (w), 1264 (m), 1210 (s), 1147 (s), 1106 (s), 1065 (s), 1031 (vs), 916 (m), 760 (m), 740 (s).

Histidine Derivative (15a)

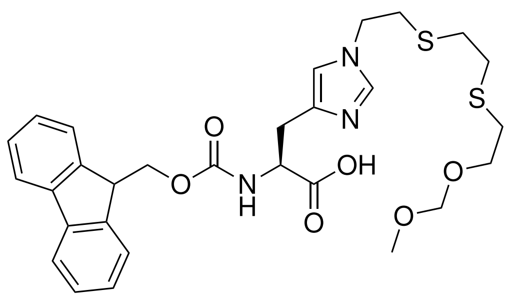

$C_{29}H_{35}N_3O_6S_2$
MW: 585.73
15a

According to **GPE** using **14a** (0.41 mmol) affording the title compound as a beige sticky solid (208 mg, 0.36 mmol, 86 %). The product was used for SPPS without further purification.
^{1}**H-NMR** (400 MHz, DMSO-d^6) δ 8.25 (s, 1H), 7.89 (d, J = 7.5 Hz, 2H), 7.73 – 7.63 (m, 3H), 7.42 (td, J = 7.5, 1.1 Hz, 2H), 7.32 (t, J = 7.4 Hz, 2H), 7.21 (s, 1H), 4.55 (s, 2H), 4.31 – 4.13 (m, 6H), 3.58 (t, J = 6.5 Hz, 2H), 3.24 (s, 3H), 3.02 (dd, J = 15.0, 4.8 Hz, 1H), 2.94 – 2.84 (m, 3H), 2.75 – 2.65 (m, 7H). ^{13}**C-NMR** (101 MHz, DMSO-d^6) δ 172.8, 155.9, 143.8, 143.7, 140.7, 136.1, 127.7, 127.1, 125.2, 120.1, 118.0, 95.6, 67.0, 65.7, 61.0, 54.7, 53.5, 46.9, 46.6, 33.9, 31.6, 31.4, 31.0, 30.9, 28.2. **LC-HRMS** (Discovery HS C18, ESI/qTOF) calc. for $C_{29}H_{36}N_3O_6S_2^+$ [M + H]$^+$: 586.2040 m/z; found 586.2040 m/z. **FT-IR** (neat) [cm^{-1}]: 3305 (br), 3136 (w), 2927 (m), 2887 (m), 1708 (s), 1619 (w), 1536 (m), 1449 (m), 1403 (m), 1321 (m), 1247 (m), 1210 (m), 1145 (s), 1104 (s), 1031 (vs), 916 (m), 760 (s), 740 (vs), 620 (s).

Histidine Derivative (15b)

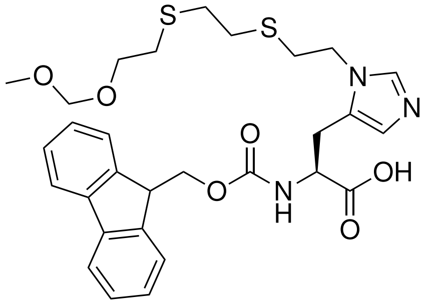

$C_{29}H_{35}N_3O_6S_2$
MW: 585.73
15b

According to **GPE** using **14b** (0.54 mmol) affording the title compound as a off-white waxy solid (296 mg, 0.51 mmol, 93 %). The product was used for SPPS without further purification.
^{1}**H-NMR** (400 MHz, DMSO-d^6) δ 7.88 (d, J = 7.5 Hz, 2H), 7.66 (d, J = 7.4 Hz, 2H), 7.53 (s, 1H), 7.41 (t, J = 7.5 Hz, 2H), 7.32 (t, J = 7.4 Hz, 2H), 7.00 (s, 1H), 6.62 (s, 1H), 4.55 (s, 2H), 4.31 – 4.16 (m, 3H), 4.16 – 3.97 (m, 2H), 3.99 – 3.90 (m, 1H), 3.58 (t, J = 6.5 Hz, 2H), 3.24 (s, 3H), 3.06 (dd, J = 15.3, 4.6 Hz, 1H), 2.90 (dd, J = 15.4, 7.6 Hz, 1H), 2.82 (t, J = 7.2 Hz, 2H), 2.73 – 2.61 (m, 6H). ^{13}**C-NMR** (101 MHz, DMSO-d^6) δ 172.4, 155.5, 143.9, 140.7, 137.0, 128.9, 128.0, 127.6, 127.3, 127.1, 127.1, 126.7, 125.3, 125.2, 121.4, 120.1, 109.8, 95.6, 67.0, 65.5, 54.7, 54.6, 46.7, 43.9, 31.8, 31.7, 31.3, 30.9, 26.0. **LC-HRMS** (Discovery HS C18, ESI/qTOF) calc. for $C_{29}H_{36}N_3O_6S_2^+$ [M + H]$^+$: 586.2040 m/z; found 586.2044 m/z. **FT-IR** (neat) [cm^{-1}]: 3319 (br), 3043 (w), 2923 (m), 1712 (s), 1597 (m), 1557 (m), 1496 (w), 1449 (m), 1439 (m), 1401 (m), 1262 (s), 1202 (m), 1144 (m), 1105 (m), 1071 (vs), 1032 (vs), 919 (m), 839 (m), 760 (s), 739 (vs).

1.3.3.2 Peptide-Synthesis

Peptide (4a)

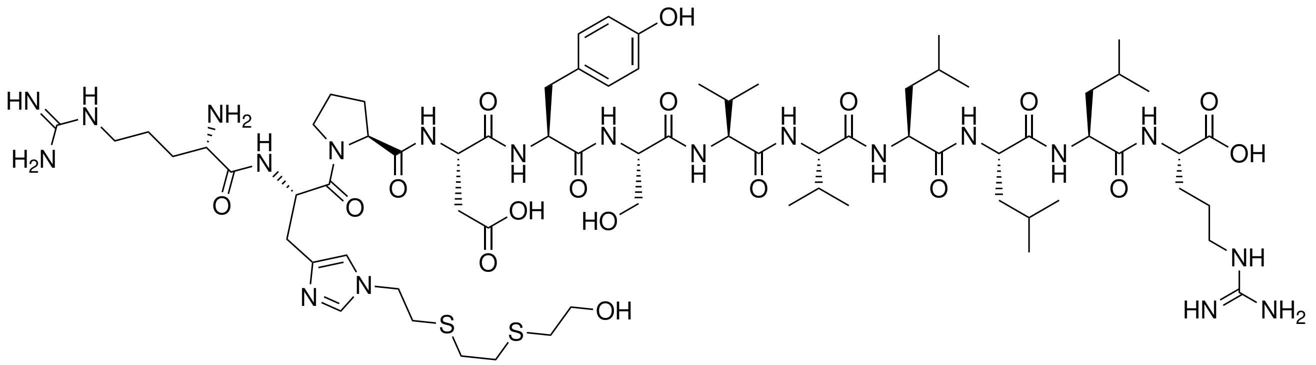

$C_{73}H_{122}N_{20}O_{18}S_2$
MW: 1632.02
4a

Resin:	H-*L*-Arg(Pbf)-2CT
Scale:	0.075 mmol
Vial:	10 mL
Equivalents:	4.0 equiv
Amino Acids:	Fmoc-Arg(Pbf)-OH, Fmoc-Pro-OH, Fmoc-Asp(^{t}Bu)-OH, Fmoc-Tyr(^{t}Bu)-OH, Fmoc-Ser(Trt)-OH, Fmoc-Val-OH, Fmoc-Leu-OH (all 0.5 M in DMF); **15a** (0.5 M in NMP)
Coupling Agents:	DIC and Oxyma (both 0.2 M in DMF), 75 °C for 5 min
Fmoc Deprotection:	5 % Piperazine in DMF (2 × 10 equiv)
Resin Cleavage:	TFA/TIPS/CH_2Cl_2 (20/5/75, v/v/v)

Peptide-synthesis according to **GPSPPS-A** using parameters as indicated above. The resin was transferred into a sintered glass funnel and treated with cleavage cocktail (3 × 10 mL) and liquid phase allowed to elute into a round-bottomed flask equipped with a stir bar. The collected filtrate was stirred at 25 °C for 2 h. The reaction mixture was concentrated to around 2 mL total volume and precipitated in ice-cold Et_2O. After sonication and centrifugation (4000 rpm, 5 min, ambient temperature), the liquid phase was decanted and the residue resuspended in ice-cold Et_2O and again centrifuged. The washing procedure was performed three times in total. Then, the residue was picked up in HPLC grade H_2O and lyophilized to afford the title compound as a white fluffy solid (113 mg, Peptide Purity: 59 %).

LC-HRMS (Aurora Elite C18, CaptiveSpray/timsTOF) calc. for $C_{73}H_{125}N_{20}O_{18}S_2^{3+}$ $[M + 3H]^{3+}$: 544.6302 m/z; found 544.6297 m/z with fragment ions (dda-PASEF, 31.7 eV, in m/z) 401.287 (y3), 458.1986 (b2), 670.2789 (b4), 799.5374 (y7), 920.3767 (b6), 962.6006 (y8), 1019.4432 (b7), 1077.6358 (y9), 1118.5134 (b8), 1174.6813 (y10), 335.6426 (b4), 401.287 (y3), 417.1768 (b5), 458.1986 (b2), 460.6915 (b6), 510.226 (b7), 514.3713 (y4), 559.7592 (b8), 613.4405 (y5), 670.2789 (b4), 712.5058 (y6), 738.3262 (y11), 799.5374 (y7), 833.3409 (b5), 920.3767 (b6), 962.6006 (y8), 1019.4432 (b7), 1077.6358 (y9), 1174.6813 (y10).

Peptide (4b)

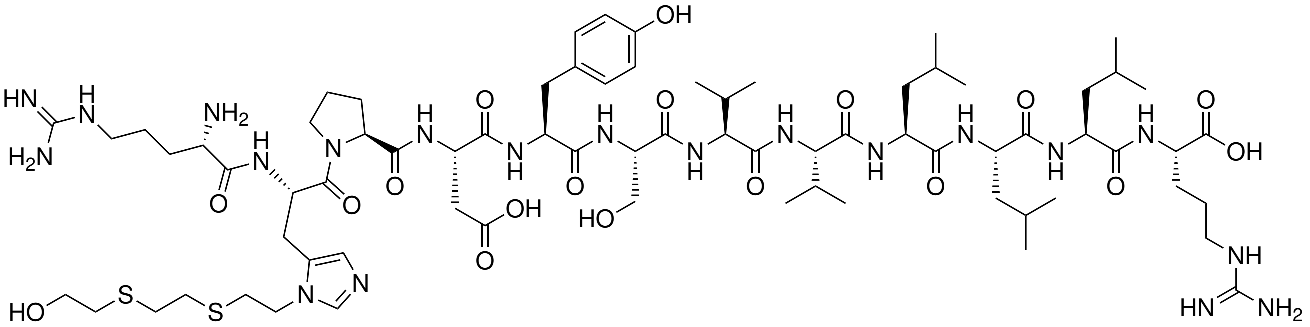

$C_{73}H_{122}N_{20}O_{18}S_2$
MW: 1632.02
4b

Resin:	H-*L*-Arg(Pbf)-2CT
Scale:	0.075 mmol
Vial:	10 mL
Equivalents:	4.0 equiv
Amino Acids:	Fmoc-Arg(Pbf)-OH, Fmoc-Pro-OH, Fmoc-Asp(^{t}Bu)-OH, Fmoc-Tyr(^{t}Bu)-OH, Fmoc-Ser(Trt)-OH, Fmoc-Val-OH, Fmoc-Leu-OH (all 0.5 M in DMF); **15b** (0.5 M in NMP)
Coupling Agents:	DIC and Oxyma (both 0.2 M in DMF), 75 °C for 5 min
Fmoc Deprotection:	5 % Piperazine in DMF (2 × 10 equiv)
Resin Cleavage:	TFA/TIPS/CH_2Cl_2 (20/5/75, v/v/v)

Peptide-synthesis according to **GPSPPS-A** using parameters as indicated above. The resin was transferred into a sintered glass funnel and treated with cleavage cocktail (3 × 10 mL) and liquid phase allowed to elute into a round-bottomed flask equipped with a stir bar. The collected filtrate was stirred at 25 °C for 2 h. The reaction mixture was concentrated to around 2 mL total volume and precipitated in ice-cold Et_2O. After sonication and centrifugation (4000 rpm, 5 min, ambient temperature), the liquid phase was decanted and the residue resuspended in ice-cold Et_2O and again centrifuged. The washing procedure was performed three times in total. Then, the residue was picked up in HPLC grade H_2O and CH_3CN and lyophilized to afford the title compound as a white fluffy solid (102 mg, Peptide Purity: 54 %).
LC-HRMS (Aurora Elite C18, CaptiveSpray/timsTOF) calc. for $C_{73}H_{125}N_{20}O_{18}S_2^{3+}$ $[M + 3H]^{3+}$: 544.6302 m/z; found 544.6287 m/z with fragment ions (dda-PASEF, 30.7 eV, in m/z) 401.287 (y3), 670.279 (b4), 799.5374 (y7), 920.3767 (b6), 962.6056 (y8), 1019.4483 (b7), 1174.6759 (y10), 335.6426 (b4), 401.287 (y3), 417.1736 (b5), 460.6915 (b6), 510.2261 (b7), 514.3713 (y4), 559.7592 (b8), 613.4405 (y5), 670.279 (b4), 712.51 (y6), 738.3305 (y11), 799.5374 (y7), 833.3409 (b5), 920.3767 (b6), 962.6056 (y8), 1019.4483 (b7), 1174.6759 (y10).

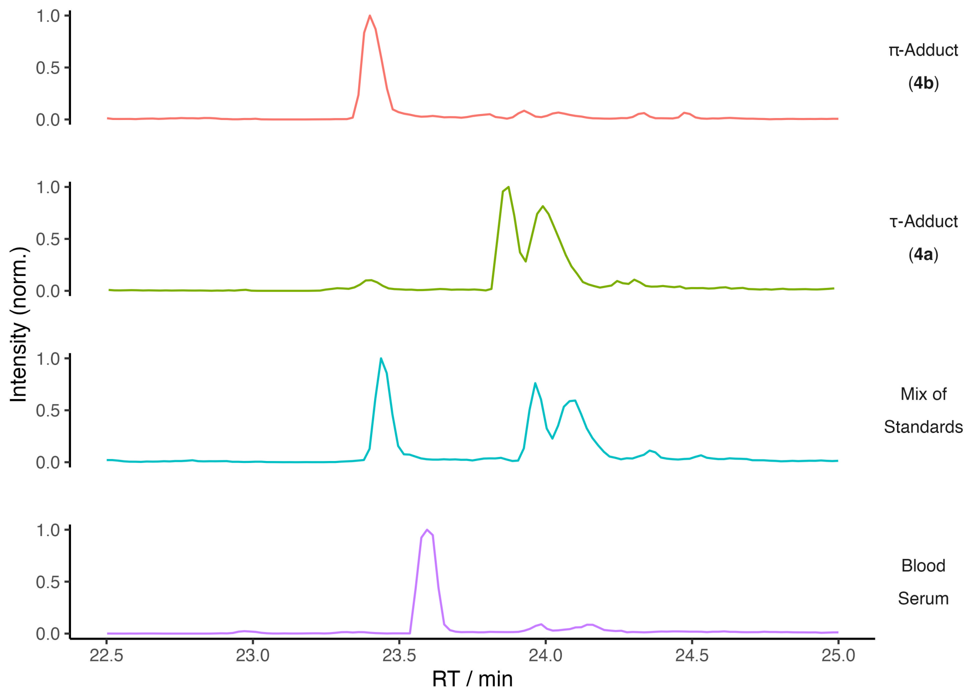

Figure S14 Retention time comparison of adducts **4a**and **4b** (top two panels) to biomarker found in blood serum (bottom panel). The findings suggest that the peptide sequence found in blood serum is almost exclusively dictated by the π-adduct.

1.3.3.3 Failed Approaches

Fmoc-His-OH (S14)

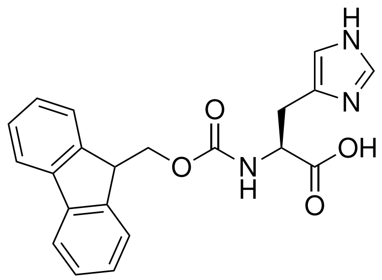

S14

In a round-bottomed flask, Fmoc-His(Trt)-OH (10.0 mmol, 1.0 equiv) was treated with a solution of TFA/TIPS/CH_2Cl_2 (30/10/60, 40 mL) at room temperature for 2 h. Then, the reaction mixture was concentrated under reduced pressure and deion. H_2O (25 mL) followed by EtOAc (50 mL), agitated and the phases separated. The organic layer was washed with sat. $NaHCO_3$ (20 mL), the pH of the combined aqueous layer adjusted to 4 and extracted with EtOAc (2 × 50 mL). The combined oragnic layer were washed with 10 % NH_4Cl/brine (2/1, v/v, 2 × 75 mL) with the aid of small amount of MeOH, dried over Na_2SO_4, filtered and concentrated under reduced pressure. The residue was re-crystallized from MeOH, filtered and the collected solids washed with toluene followed by n-Hexan. Drying under vacuum at 70 °C afforded the TFA salt of the title product as a white solid (2.64 g, 5.3 mmol, 53 %; positive Pauly Test).
^{1}**H-NMR** (400 MHz, DMSO-d^6) δ 8.95 (d, J = 1.4 Hz, 1H), 7.88 (d, J = 7.6 Hz, 2H), 7.81 (d, J = 8.4 Hz, 1H), 7.65 (d, J = 7.5 Hz, 2H), 7.46 – 7.37 (m, 2H), 7.37 – 7.25 (m, 3H), 4.34 (ddd, J = 10.0, 8.4, 4.9 Hz, 1H), 4.29 – 4.24 (m, 2H), 4.22 – 4.17 (m, 1H), 3.17 (dd, J = 15.2, 4.9 Hz, 1H), 3.02 (dd, J = 15.2, 10.1 Hz, 1H). ^{13}**C-NMR** (101 MHz, DMSO-d^6) δ 172.3, 158.5 (q, J = 31.4 Hz), 156.0, 143.8, 143.7, 140.6, 140.7, 134.0, 129.8, 127.7, 127.1, 125.2, 125.2, 120.2, 117.2 (q, J = 299.3 Hz), 117.0, 65.7, 53.0, 48.6, 46.6, 26.2. Spectral data matches literature[28].

Alkyl Chloride (S15)

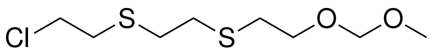

$C_8H_{17}ClO_2S_2$
MW: 244.79
S15

In a heat-gun dried, two-necked, round-bottomed flask equipped with a stir bar and septum, to a solution of **6** (5.0 mmol, 1.0 equiv) in CH_2Cl_2 (10 mL) and DIPEA (6.0 mmol, 1.2 equiv) was added and the resulting solution cooled to 0 °C. Then, MsCl (6.0 mmol, 1.2 equiv) was added and the reaction mixture was stirred at 0 °C for 1 h, then allowed to warm up to room temperature and stirring continued overnight. The reaction mixture was quenched with Et_2O (50 mL) and the solution washed with 0.1 M HCl (2 × 25 mL), brine (25 mL), dried over Na_2SO_4 and filtered. Concentration under reduced pressure of the filtrate afforded the title compound as a colorless liquid (1.20 g, 4.91 mmol, > 95 %).
^{1}**H-NMR** (400 MHz, $CDCl_3$) δ 4.65 (s, 2H), 3.72 (t, J = 6.5 Hz, 2H), 3.68 – 3.62 (m, 2H), 3.38 (s, 3H), 2.95 – 2.85

(m, 2H), 2.80 (s, 4H), 2.78 (t, J = 6.5 Hz, 2H). ^{13}**C-NMR** (101 MHz, $CDCl_3$) δ 96.5, 67.5, 55.4, 43.1, 34.3, 32.7, 32.5, 32.1. **LC-HRMS** (Discovery HS C18, ESI/qTOF) calc. for $C_8H_{18}ClO_2S_2^+$ [M + H]$^+$: 245.0431 m/z; found 245.0431 m/z. **FT-IR** (neat) [cm^{-1}]: 2927 (m), 2884 (m), 2823 (w), 1440 (m), 1424 (m), 1381 (w), 1293 (m), 1274 (m), 1201 (m), 1147 (s), 1105 (s), 1066 (s), 1031 (vs), 918 (m), 691 (m), 687 (m), 647 (m), 626 (m).

1.3.4 Synthesis of Tripeptide

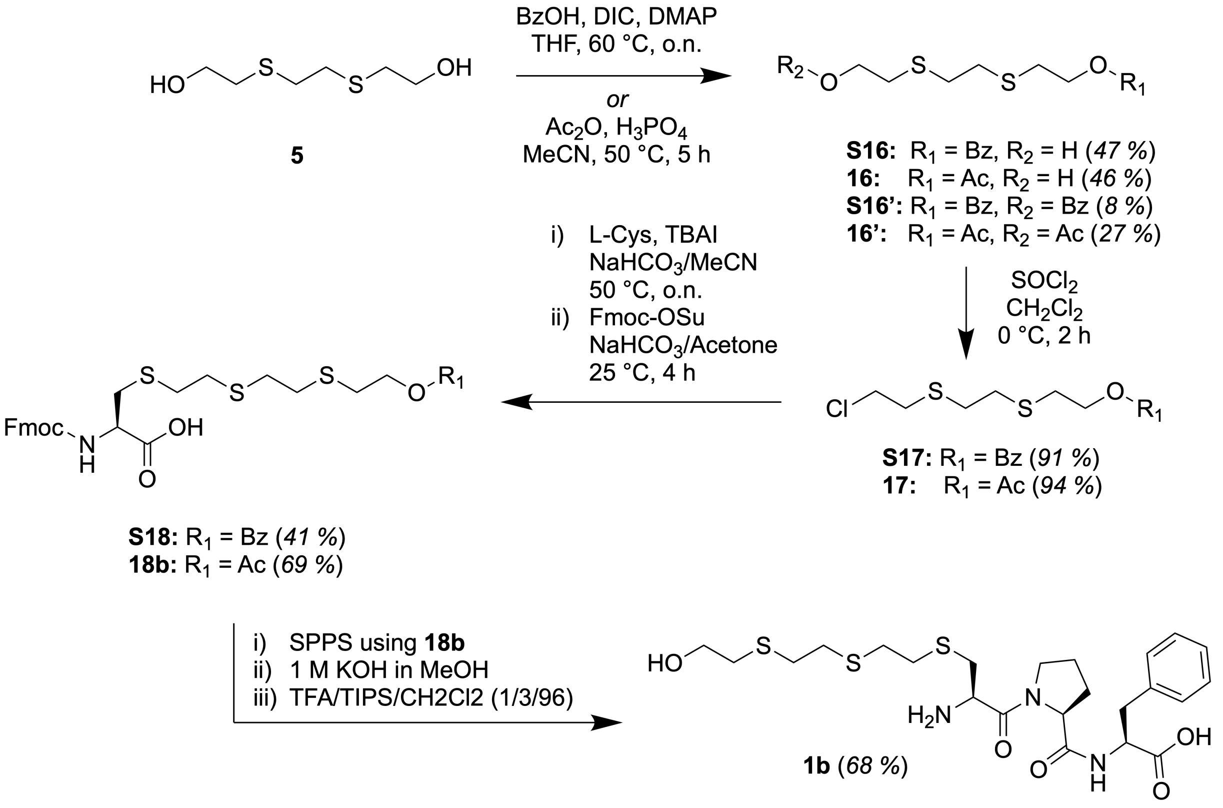

Scheme S4 Synthesis approach for the preparation of the tripeptide [HETETE]-CPF. The derivatives of HD and T are prepared analogously starting from the corresponding diols.

1.3.4.1 Optimization of Cysteine Etherification and Fmoc-Protection

Each entry is around 2.0 mmol ansatz. A round-bottomed flask was charged with the corresponding amount of *L*-Cys (1.0 equiv if not stated elsewise) and dissolved in sat. $NaHCO_3$. Then, a solution of **S16** or **16** in CH_3CN was added to receive a 0.1 M solution. The resulting reaction mixture was stirred at the indicated temperature for 24 h. Then, the resulting slurry was allowed to cool to room temperature and a solution of Fmoc-OSu in acetone (1.5 equiv, 0.3 M) was added and the pH adjusted to 8 - 9 if necessary. The resulting reaction mixture was stirred at room temperature overnight if not stated elsewise. The reaction mixture was then quenched with 2 M HCl to pH 1 and EtOAc (75 mL) and the layers were separated. The aqueous layer was extracted with EtOAc (2 × 75 mL) and the combined organic layers dried over Na_2SO_4, concentrated under reduced pressure and the crude product purified via column chromatography.

Table S9 Optimization of the coupling between Cys and monoprotected hydroxyethylthioethylalcohol. [a]Starting material used.

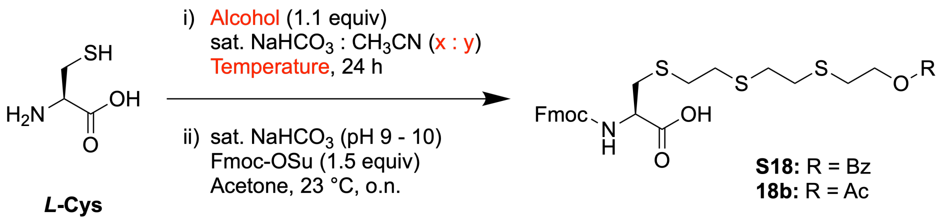

Entry	SM[a]	x	y	Temperature [°C]	Yield [%]	Remarks
1	**S16**	2	1	25	< 5	
2	**S16**	2	1	60	20	
3	**S16**	1	1	25	41	
4	**S16**	1	2	25	38	
5	**S16**	1	2	25	42	TBAI Additive
6	**S16**	1	2	25	41	Addition of Cys in sat. $NaHCO_3$ to **S17** in CH_3CN
7	**S16**	1	2	50	59	Impure, even after column
8	**S16**	1	2	50	49	TBAI Additive
9	**S16**	1	2	50		THF instead of CH_3CN, TBAI Additive
10	**S16**	1	3	25	35	
11	**S16**	1	1	25	-	CH_2Cl_2 instead of CH_3CN, TBAI Additive
12	**16**	1	2	50	50	optically impure product
13	**16**	1	2	50	49	TBAI Additive
14	**16**	1	2	50	68	*L*-Cys (1.5 equiv), **17** (1.0 equiv), TBAI Additive
15	**16**	1	2	50	< 5	Fmoc-OSu protection, then thioether synthesis using *L*-Cys (1.5 equiv), **17** (1.0 equiv, TBAI Additive, first Fmoc-protection, then thioether-formation

1.3.4.2 Preparation and Characterization of Substrates

General Procedure for Acetyl Protection of Alcohols (GPF)

A heat-gun dried two-necked round-bottomed flask equipped with a stir bar, dropping funnel and septum was charged with H_3PO_4 (1.8 mmol, 0.4 equiv) and CH_3CN (30 mL). Then, corresponding diol (50.0 mmol, 1.0 equiv) was added in one portion followed by dropwise addtion Ac_2O (55.0 mmol, 1.1 equiv) via dropping funnel and the funnel washed with CH_3CN (1 mL). The resulting reaction mixture was heated to 50 °C and stirred for 5 h. Then, the reaction was quenched with sat. $NaHCO_3$ (50 mL) and MeOH (30 mL) and stirred for 15 min. The mixture was transferred into a separatory funnel with the aid of deion. H_2O (30 mL) and Et_2O (50 mL). The phases were separated and the aqueous layer extracted with Et_2O (2×100 mL). The combined organic layers were washed with brine (75 mL), dried over Na_2SO_4 and concentrated under reduced pressure.

General Procedure for the Chlorination of Alcohols (GPH)

A heat-gun dried two-necked round-bottomed flask equipped with a stir bar and septum was charged with the corresponding alcohol (1.0 equiv) and evacuated and flushed with Ar three time. Then, CH_2Cl_2 (0.25 M – 0.3 M) was added and the resulting solution cooled to 0 °C in an ice bath. $SOCl_2$ (2.0 equiv) was added dropwise over 10 min at this temperature and the reaction mixture stirred at 0 °C for 2 h. After allowing to warm up to RT the solvent was removed under reduced pressure and the crude product purified by column chromatography to afford the title compound.

General Procedure for the Coupling of Thioether Chlorides to Cysteine and Subsequent *N*-Fmoc-Protection (GPI)

A round-bottomed flask equipped with a stir bar was charged with *L*-Cys (1.5 equiv) and sat. aq. $NaHCO_3$ (3 mL per mmol thioether chloride) and the suspension stirred until homogenous. Then, a solution of the corresponding thioether chloride (1.0 equiv) and TBAI (0.2 equiv) in CH_3CN (6 mL per mmol thioether chloride) was added and the reaction mixture stirred at 50 °C overnight. Then, the formed slurry was allowed to cool down to room temperature and a solution of Fmoc-OSu (1.5 equiv) in acetone (4 mL per mmol thioether chloride) was added dropwise, the pH adjusted between 9 – 10 with additional sat. aq. $NaHCO_3$ if necessary and the solution stirred at room temperature for 4 h.

The reaction mixture was quenched with 2 M HCl to a pH of 1 and EtOAc (approx. 15 mL per mmol thioether chloride). The layers were separated and the aqueous layer extracted with EtOAc twice. The combined organic layers were washed with brine, dried over Na_2SO_4, filtered and concentrated under reduced pressure.

Alcohol (S16)

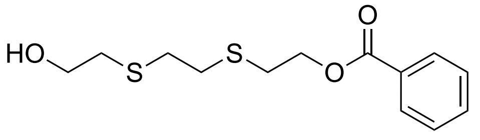

$C_{13}H_{18}O_3S_2$
MW: 286.40
S16

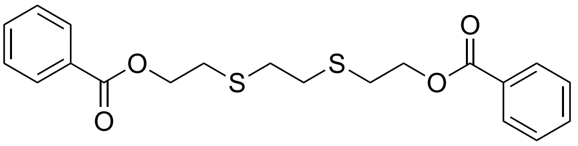

$C_{20}H_{22}O_4S_2$
MW: 390.51
S16'

According to **GPG** using diol **5** (20.0 mmol). Purification via flash column chromatography (Isolera Sfar HC Duo 50 g, 100 mL min^{-1}, n-Hex/EtOAc, 5 % – 10 % EtOAc (10.0 CV), 10 % EtOAc (10.0 CV), 10 % – 20 % EtOAc (5.0 CV), 20 % – 40 % EtOAc (5.0 CV), 40 % EtOAc (8.0 CV)) afforded the title compound as a colorless oil (2.73 g, 9.54 mmol, 47 %) and undesired bisprotected side-product **S16'** as a crystalline solid (634 mg, 1.63 mmol, 8 %)

^{1}**H-NMR** (400 MHz, $CDCl_3$) δ 8.08 – 8.00 (m, 2H), 7.62 – 7.53 (m, 1H), 7.50 – 7.40 (m, 2H), 4.48 (t, *J* = 6.9 Hz, 2H), 3.75 (q, *J* = 6.0 Hz, 2H), 2.95 – 2.72 (m, 9H), 2.40 (t, *J* = 6.0 Hz, 1H). ^{13}**C-NMR** (101 MHz, $CDCl_3$) δ 166.4, 133.2, 129.8, 129.6, 128.4, 64.0, 60.8, 35.3, 32.4, 31.9, 30.6. **LC-HRMS** (Discovery HS C18, ESI/qTOF) calc. for $C_{13}H_{19}O_3S_2^+$ [M + H]$^+$: 287.0770 m/z; found 287.0768 m/z. **FT-IR** (neat) [cm^{-1}]: 3420 (br), 2917 (w), 2877 (w), 1713 (s), 1602 (w), 1585 (w), 1451 (m), 1378 (m), 1268 (vs), 1202 (m), 1177 (m), 1109 (s), 1069 (m), 1025 (m), 955 (w), 710 (s). **R**$_f$ (n-Hex/EtOAc, 2/1): 0.35.

Bisprotected Side-Product: ^{1}**H-NMR** (400 MHz, $CDCl_3$) δ 8.08 – 8.00 (m, 4H), 7.60 – 7.51 (m, 2H), 7.48 – 7.39 (m, 4H), 4.48 (t, *J* = 6.9 Hz, 4H), 2.92 (t, *J* = 6.9 Hz, 4H), 2.87 (s, 4H). ^{13}**C-NMR** (101 MHz, $CDCl_3$) δ 166.3, 133.1, 129.9, 129.7, 128.4, 64.0, 32.4, 30.7. **LC-HRMS** (Discovery HS C18, ESI/qTOF) calc. for $C_{20}H_{23}O_4S_2^+$ [M + H]$^+$: 391.1032 m/z; found 391.1031 m/z. **FT-IR** (neat) [cm^{-1}]: 2967 (w), 1709 (vs), 1600 (m), 1582 (m), 1490 (w), 1464 (m), 1437 (m), 1437 (m), 1378 (m), 1311 (m), 1280 (vs), 1247 (s), 1205 (m), 1179 (m), 1116 (vs), 1068 (m), 1025 (m), 965 (m), 860 (w), 706 (vs). **mp:** 94.2 °C. **R**$_f$ (n-Hex/EtOAc, 6/1): 0.26.

Alkyl Chloride (S17)

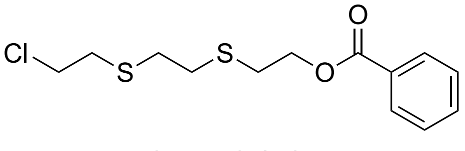

$C_{13}H_{17}ClO_2S_2$
MW: 304.85
S17

According to **GPH** using alcohol **S16** (4.5 mmol). Purification via flash column chromatography (Isolera SNAP Ultra 10 g, 36 mL min^{-1}, n-Hex/EtOAc, 0 % – 5 % EtOAc (8.0 CV), 5 % – 15 % EtOAc (3.0 CV), 15 % – 40 % EtOAc (3.0 CV)) afforded the title compound as a colorless liquid (1.253 g, 4.11 mmol, 91 %).

^{1}**H-NMR** (400 MHz, $CDCl_3$) δ 8.09 – 8.00 (m, 2H), 7.62 – 7.53 (m, 1H), 7.50 – 7.41 (m, 2H), 4.49 (t, *J* = 6.9 Hz, 2H), 3.68 – 3.59 (m, 2H), 2.96 – 2.77 (m, 8H). ^{13}**C-NMR** (101 MHz, $CDCl_3$) δ 166.34, 133.16, 129.87, 129.65, 128.44, 63.96, 43.09, 34.37, 32.50, 32.48, 30.73. **LC-HRMS** (Discovery HS C18, ESI/qTOF) calc. for $C_{13}H_{18}ClO_2S_2^+$ [M + H]$^+$: 305.0431 m/z; found 305.0431 m/z. **FT-IR** (neat) [cm^{-1}]: 2987 (w), 2969 (w), 2954 (w), 2929 (w), 1718 (s), 1600 (m), 1583 (m), 1464 (m), 1440 (m), 1423 (m), 1376 (m), 1313 (m), 1295 (m), 1270 (vs), 1242 (s), 1200 (m), 1174 (m), 1111 (s), 1098 (s), 1068 (s), 1023 (m), 956 (m), 857 (m), 809 (m), 709 (vs), 697 (s), 681 (s). **R**$_f$ (n-Hex/EtOAc, 15/1): 0.25.

Cysteine Derivative (S18)

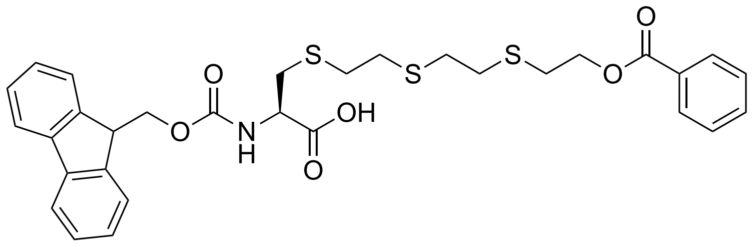

$C_{31}H_{33}NO_6S_3$
MW: 611.79
S18

According to **GPC** but using *L*-Cys (1.7 mmol, 1.0 equiv), thioether chloride **S17** (1.1 equiv) and sat. aq. $NaHCO_3$ (7.5 mL), CH_3CN (15 mL) and acetone (7 mL) as solvents. Purification via flash column chromatography (Isolera Sfar C18 Duo 30 g, 25 mL min^{-1}, H_2O + 0.1 % FoA/CH_3CN + 0.1 % FoA (A/B), 40 % – 50 % B (20.0 CV), 50 % – 60 % B (9.5 CV), 60 % – 70 % B (3.5 CV), 70 % B (2.3 CV)) afforded the title compound as a white solid (437 mg, 0.72 mmol, 41 %).
^{1}**H-NMR** (400 MHz, CD_3CN) δ 8.04 – 7.97 (m, 2H), 7.82 (d, *J* = 7.6 Hz, 2H), 7.70 – 7.56 (m, 3H), 7.52 – 7.46 (m, 2H), 7.41 (td, *J* = 7.5, 1.1 Hz, 2H), 7.33 (tt, *J* = 7.5, 1.3 Hz, 2H), 6.13 (d, *J* = 8.2 Hz, 1H), 4.47 – 4.29 (m, 4H), 4.24 (t, *J* = 6.9 Hz, 1H), 3.04 (dd, *J* = 13.9, 4.8 Hz, 1H), 2.95 – 2.67 (m, 12H). ^{13}**C-NMR** (101 MHz, CD_3CN) δ 172.3, 167.0, 156.9, 145.0, 145.0, 142.1, 134.1, 131.1, 130.3, 129.6, 128.7, 128.1, 128.1, 126.2, 121.0, 67.5, 65.0, 54.8, 47.9, 34.3, 33.3, 33.0, 32.6, 32.5, 31.1. **LC-HRMS** (Discovery HS C18, ESI/qTOF) calc. for $C_{31}H_{34}NO_6S_3^+$ [M + H]$^+$: 612.1543 m/z; found 612.1552 m/z. **FT-IR** (neat) [cm^{-1}]: 3312 (m), 3063 (w), 2924 (w), 1713 (s), 1689 (vs), 1534 (m), 1450 (m), 1421 (m), 1314 (m), 1268 (vs), 1194 (m), 1105 (s), 1046 (m), 986 (w), 938 (w), 757 (m), 737 (s), 709 (vs), 684 (m), 674 (m), 620 (m). **mp**: 55 °C. **R***f* (H_2O +0.1 % AcOH/CH_3CN +0.1 % AcOH, 3/7): 0.30.

Alcohol (16)

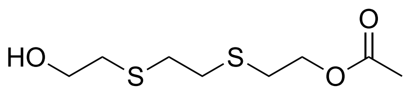

$C_8H_{16}O_3S_2$
MW: 224.33
16

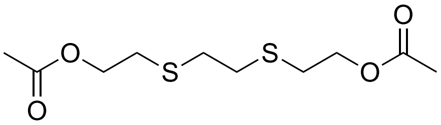

$C_{10}H_{18}O_4S_2$
MW: 266.37
16'

According to **GPF** using diol **5** (50.0 mmol). Purification via column chromatography (Isolera Sfar HC Duo 100 g, 100 mL min^{-1}, n-Hex/EtOAc, 20 % – 30 % EtOAc (10.0 CV), 30 % – 60 % EtOAc (5.0 CV), 60 % EtOAc (4.0 CV)) afforded the title compound as a colorless liquid (5.219 g, 23.2 mmol, 46 %) and undesired bisprotected side-product **16'** as a colorless liquid (3.644 g, 13.6 mmol, 27 %) .
^{1}**H-NMR** (400 MHz, $CDCl_3$) δ 4.23 (t, *J* = 6.9 Hz, 2H), 3.76 (q, *J* = 5.9 Hz, 2H), 2.85 – 2.72 (m, 8H), 2.26 (t, *J* = 6.1 Hz, 1H), 2.08 (s, 3H). ^{13}**C-NMR** (101 MHz, $CDCl_3$) δ 171.1, 63.5, 60.9, 35.5, 32.5, 32.0, 30.6, 21.1. **LC-HRMS** (Discovery HS C18, ESI/qTOF) calc. for $C_8H_{17}O_3S_2^+$ [M + H]$^+$: 225.0614 m/z; found 225.0615 m/z. **FT-IR** (neat) [cm^{-1}]: 3445 (br), 2923 (w), 2879 (w), 1735 (s), 1426 (m), 1383 (m), 1361 (m), 1227 (vs), 1029 (s), 970 (w).
Bisprotected Side-Product: ^{1}**H-NMR** (400 MHz, $CDCl_3$) δ 4.23 (t, *J* = 6.8 Hz, 4H), 2.83 – 2.75 (m, 8H), 2.08 (s, 6H). ^{13}**C-NMR** (101 MHz, $CDCl_3$) δ 170.9, 63.6, 32.4, 30.7, 21.0. **LC-HRMS** (Discovery HS C18, ESI/qTOF) calc. for $C_{10}H_{19}O_4S_2^+$ [M + H]$^+$: 267.0719 m/z; found 267.0719 m/z. **FT-IR** (neat) [cm^{-1}]: 2954 (w), 2921 (w), 1735 (vs), 1429 (w), 1381 (m), 1363 (m), 1224 (vs), 1026 (s), 970 (w). **R***f* (n-Hex/EtOAc, 5/1): 0.33.

Alkyl Chloride (17)

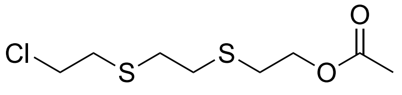

$C_8H_{15}ClO_2S_2$
MW: 242.78
17

According to **GPH** using alcohol **16** (21.9 mmol). Purification via flash column chromatography (Isolera Sfar HC Duo 50 g, 120 mL min^{-1}, n-Hex/EtOAc,5 % – 30 % EtOAc (15.0 CV)) afforded the title compound as a colorless liquid (5.133 g, 21.1 mmol, 94 %).
^{1}**H-NMR** (400 MHz, $CDCl_3$) δ 4.23 (t, *J* = 6.8 Hz, 2H), 3.69 – 3.61 (m, 2H), 2.95 – 2.86 (m, 2H), 2.82 – 2.76 (m, 6H), 2.08 (s, 3H). ^{13}**C-NMR** (101 MHz, $CDCl_3$) δ 170.8, 63.4, 43.1, 34.4, 32.4, 32.4, 30.6, 20.9. **LC-HRMS** (Discovery HS C18, ESI/qTOF) calc. for $C_8H_{16}ClO_2S_2^+$ [M + H]$^+$: 243.0275 m/z; found 243.0273 m/z. **FT-IR** (neat) [cm^{-1}]: 2960 (w), 2923 (w), 1735 (vs), 1426 (m), 1381 (m), 1363 (m), 1227 (s), 1028 (m), 970 (w), 687 (m), 643 (m). **R***f* (n-Hex/EtOAc, 7/1): 0.22.

Cysteine Derivative (18b)

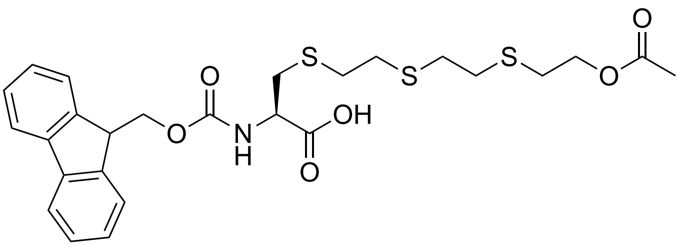

$C_{26}H_{31}NO_6S_3$
MW: 549.71
18b

According to **GPC** using thioether chloride **17** (10.0 mmol) and sat. aq. $NaHCO_3$ (35 mL), CH_3CN (70 mL) and acetone (40 mL) as solvents. Purification via flash column chromatography (Isolera Sfar C18 Duo 120 g, 50 mL min^{-1}, H_2O + 0.1 % FoA/CH_3CN + 0.1 % FoA (A/B), 40 % – 55 % B (25 CV)) afforded the title compound as a white solid (3.81 g, 6.9 mmol, 69 %). ^{1}**H-NMR** (400 MHz, CD_3CN) δ 7.84 (d, J = 7.5 Hz, 2H), 7.68 (d, J = 7.5 Hz, 2H), 7.42 (td, J = 7.5, 1.2 Hz, 2H), 7.34 (tt, J = 7.5, 1.3 Hz, 2H), 6.13 (d, J = 8.3 Hz, 1H), 4.42 – 4.30 (m, 3H), 4.26 (t, J = 6.9 Hz, 1H), 4.14 (t, J = 6.8 Hz, 2H), 3.05 (dd, J = 14.0, 4.8 Hz, 1H), 2.89 (dd, J = 14.0, 7.9 Hz, 1H), 2.74 (d, J = 6.9 Hz, 11H), 1.98 (s, 3H). ^{13}**C-NMR** (101 MHz, CD_3CN) δ 172.3, 171.6, 157.0, 145.1, 145.0, 142.2, 128.7, 128.2, 128.1, 126.2, 121.0, 67.5, 64.4, 54.8, 48.0, 34.3, 33.3, 33.0, 32.7, 32.6, 31.0, 21.1. **LC-HRMS** (Discovery HS C18, ESI/qTOF) calc. for $C_{26}H_{32}NO_6S_3^+$ $[M + H]^+$: 550.1386 m/z; found 550.1394 m/z. **FT-IR** (neat) [cm^{-1}]: 3312 (m), 3050 (w), 2926 (w), 1718 (s), 1689 (vs), 1536 (s), 1449 (m), 1421 (m), 1227 (vs), 1194 (s), 1084 (m), 1031 (s), 985 (m), 759 (m), 737 (vs), 646 (m). **mp**: 50 °C – 52 °C. **R**$_f$ (H_2O +0.1 % AcOH/CH_3CN +0.1 % AcOH, 4/6): 0.36.

Tripeptide (1b)

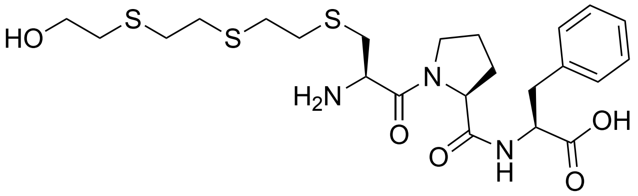

$C_{23}H_{35}N_3O_5S_3$
MW: 529.73
1b

Resin:	H-*L*-Phe-2CT
Scale:	0.4 mmol
Vial:	30 mL
Equivalents:	4.0 equiv
Amino Acids:	Fmoc-Pro-OH (0.5 M in DMF), **18b** (0.5 M in NMP)
Coupling:	DIC and Oxyma (both 0.5 M in DMF), ambient conditions for 60 min
Fmoc Deprotection:	5 % Piperazine in DMF (2 × 9 mL)
Cleavage Cocktail:	TFA/TIPS/CH_2Cl_2 (1/3/96, v/v/v)

Peptide-synthesis according to **GPSPPS-B** using parameters as indicated above. *Cleavage:* In a round-bottomed flask equipped with a stir bar, cleavage cocktail (15 mL) was added and the slurry stirred for 30 min.After filtration through a sintered glass funnel, cleavage cocktail (2×15 mL) was added to the collected resin in the funnel and the resulting slurry let elute for 30 min. The collected filtrates were combined and concentrated under reduced pressure. Purification via flash column chromatography (Isolera Sfar HC C18 Duo 12 g, 12 mL min^{-1}, liquid loading, H_2O + 0.1 % FoA/CH_3CN + 0.1 % FoA, 5 % CH_3CN + 0.1 % FoA (3.0 CV), 5 % – 30 % CH_3CN + 0.1 % FoA (10.0 CV), 30.0 % CH_3CN + 0.1 % FoA (2.0 CV)) and lyophilization afforded the title compound as a white fluffy solid (140 mg, 0.27 mmol, 66 %).

^{1}**H-NMR** (400 MHz, DMSO-d^6/D_2O, 5/1) δ 7.33 – 7.12 (m, 5H), 4.32 (td, J = 4.1, 1.7 Hz, 1H), 4.17 – 4.08 (m, 1H), 3.55 (dd, J = 8.2, 4.5 Hz, 1H), 3.50 (t, J = 6.7 Hz, 2H), 3.43 (dt, J = 11.7, 7.9 Hz, 1H), 3.36 – 3.28 (m, 1H), 3.13 (dd, J = 14.4, 4.6 Hz, 1H), 2.97 (dd, J = 14.0, 4.0 Hz, 1H), 2.92 – 2.83 (m, 2H), 2.75 – 2.62 (m, 9H), 2.57 (t, J = 6.7 Hz, 2H), 2.23 – 2.11 (m, 1H), 1.94 – 1.67 (m, 2H). ^{13}**C-NMR** (101 MHz, DMSO-d^6/D_2O, 5/1) δ 170.9, 169.9, 165.1, 137.1, 130.0, 129.2, 127.5, 61.4, 59.2, 55.8, 55.8, 45.5, 37.0, 34.3, 33.3, 33.2, 32.3, 32.1, 32.1, 28.9, 22.3. **LC-HRMS** (Discovery HS C18, ESI/qTOF) calc. for $C_{23}H_{36}N_3O_5S_3^+$ $[M + H]^+$: 530.1812 m/z; found 530.1811 m/z. **FT-IR** (neat) [cm^{-1}]: 3321 (w), 2911 (w), 2880 (w), 1639 (vs), 1592 (s), 1530 (s), 1453 (m), 1381 (s), 1265 (w), 1197 (m), 1128 (m), 1045 (m), 834 (w), 756 (m), 700 (vs). **mp**: 137.5 °C (decomp.).

Alcohol (S19)

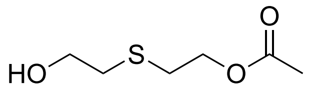

$C_6H_{12}O_3S$
MW: 164.22
S19

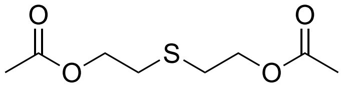

$C_8H_{14}O_4S$
MW: 206.26
S19'

According to **GPF** using diol **TDG** (50.0 mmol). Purification via flash column chromatography (Isolera Sfar HC Duo 100 g, 100 mL min^{-1}, n-Hex/EtOAc, 10 % – 30 % EtOAc (10.0 CV), 30 % – 60 % EtOAc (5.0 CV), 60 % EtOAc (2.0 CV)) afforded the title compound as a colorless oil (3.43 g, 20.8 mmol, 41 %) and undesired bisprotected side-product **S19'** as a colorless oil (1.88 g, 9.1 mmol, 18 %).
^{1}**H-NMR** (400 MHz, $CDCl_3$) δ 4.24 (t, *J* = 6.8 Hz, 2H), 3.76 (t, *J* = 5.9 Hz, 2H), 2.78 (t, *J* = 5.9 Hz, 2H), 2.78 (t, *J* = 6.8 Hz, 2H), 2.25 (s, 1H), 2.08 (s, 3H). ^{13}**C-NMR** (101 MHz, $CDCl_3$) δ 170.9, 63.4, 60.6, 35.6, 30.3, 20.9. **LC-HRMS** (Discovery HS C18, ESI/qTOF) calc. for $C_6H_{13}O_3S^+$ $[M + H]^+$: 165.0580 m/z; found 165.0580 m/z. **FT-IR** (neat) [cm^{-1}]: 3438 (br), 2956 (w), 2929 (w), 2878 (w), 1735 (s), 1383 (m), 1361 (m), 1225 (vs), 1029 (s), 969 (m). **R**$_f$ (n-Hex/EtOAc, 2/1): 0.22.
Bisprotected Side-Product: ^{1}**H-NMR** (400 MHz, $CDCl_3$) δ 4.24 (t, *J* = 6.8 Hz, 4H), 2.80 (t, *J* = 6.8 Hz, 4H), 2.08 (s, 6H). ^{13}**C-NMR** (101 MHz, $CDCl_3$) δ 170.8, 63.4, 30.7, 20.9. **LC-HRMS** (Discovery HS C18, ESI/qTOF) calc. for $C_8H_{14}O_4S^+$ $[M + H]^+$: 207.0686 m/z; found 207.0686 m/z. **FT-IR** (neat) [cm^{-1}]: 2954 (w), 1733 (vs), 1451 (w), 1430 (w), 1381 (m), 1361 (m), 1221 (vs), 1028 (s), 970 (m). **R**$_f$ (n-Hex/EtOAc, 2/1): 0.86.

Alkyl Chloride (S20)

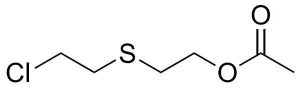

$C_6H_{11}ClO_2S$
MW: 182.66
S20

According to **GPH** using alcohol **S19** (19.1 mmol). Purification via flash column chromatography (Isolera Sfar HC Duo 50 g, 120 mL min^{-1}, n-Hex/EtOAc, 5 % – 29 % EtOAc (14.0 CV)) afforded the title compound as a colorless liquid (3.28 g, 17.9 mmol, 94 %).
^{1}**H-NMR** (400 MHz, $CDCl_3$) δ 4.24 (t, *J* = 6.7 Hz, 2H), 3.70 – 3.61 (m, 2H), 2.96 – 2.88 (m, 2H), 2.80 (t, *J* = 6.7 Hz, 2H), 2.08 (s, 3H). ^{13}**C-NMR** (101 MHz, $CDCl_3$) δ 170.8, 63.6, 43.0, 34.5, 30.8, 20.9. **LC-HRMS** (Discovery HS C18, ESI/qTOF) calc. for $C_{11}H_{14}ClO_2S^+$ $[M + H]^+$: *Not detected due to absent ionization of substance.* **FT-IR** (neat) [cm^{-1}]: 2959 (m), 2924 (m), 2860 (m), 1736 (s), 1381 (m), 1360 (m), 1225 (s), 1106 (s), 1029 (m), 969 (w), 694 (m), 643 (m), 604 (m). **R**$_f$ (n-Hex/EtOAc, 4/1): 0.73.

Cysteine Derivative (18a)

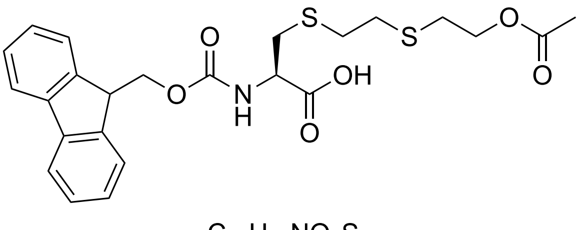

$C_{24}H_{27}NO_6S_2$
MW: 489.60
18a

According to **GPC** using thioether chloride **S20** (10 mmol) and sat. aq. $NaHCO_3$ (37.5 mL), CH_3CN (75 mL) and acetone (37.5 mL) as solvents. The crude product was purified via flash column chromatography (Isolera Sfar C18 Duo 120 g, 50 mL min^{-1}, H_2O + 0.1 % FoA/CH_3CN + 0.1 % FoA (A/B), 35 % – 60 % B (40.0 CV)). Due to co-elution of the product with Fmoc-OSu, the obtained solid was purified via normal phase column chromatography (Isolera Sfar HC Duo 25 g, 150 mL min^{-1}, PhMe/EtOAc + 2 % FoA, 7 % EtOAc + 2 % (7.0 CV), n-Hex + 2 % FoA/EtOAc + 2 % FoA, 0 % EtOAc + 2 % FoA (3 CV), 50 % – 7 % EtOAc + 2 % FoA (3 CV)) and lyophilized to afford the title compound as a yellowish honey (2.53 g, 5.1 mmol, 51 %).
^{1}**H-NMR** (400 MHz, CD_3CN) δ 7.82 (d, *J* = 7.5 Hz, 2H), 7.67 (d, *J* = 7.5 Hz, 2H), 7.41 (t, *J* = 7.5 Hz, 2H), 7.33 (t, *J* = 7.4 Hz, 2H), 6.17 (d, *J* = 8.0 Hz, 1H), 4.47 – 4.31 (m, 3H), 4.24 (t, *J* = 6.9 Hz, 1H), 4.13 (t, *J* = 6.7 Hz, 2H), 3.05 (dd, *J* = 14.0, 4.6 Hz, 1H), 2.90 (dd, *J* = 14.0, 7.7 Hz, 1H), 2.73 (dd, *J* = 12.0, 5.3 Hz, 6H), 1.97 (s, 3H). ^{13}**C-NMR** (101 MHz, CD_3CN) δ 172.6, 171.6, 157.0, 145.1, 145.0, 142.1, 128.7, 128.1, 128.1, 126.2, 121.0, 118.3, 67.5, 64.3, 54.9, 48.0, 34.3, 33.2, 32.7, 30.9, 21.0. **LC-HRMS** (Discovery HS C18, ESI/qTOF) calc. for $C_{24}H_{28}NO_6S_2^+$ $[M + H]^+$: 490.1353 m/z; found 490.1355 m/z. **FT-IR** (neat) [cm^{-1}]: 3319 (m), 3052 (w), 2961 (w), 2926 (w), 1726 (s), 1683 (vs), 1533 (s), 1449 (m), 1421 (m), 1227 (vs), 1207 (s), 1082 (m), 1045 (s), 1031 (s), 757 (m), 737 (vs). **R**$_f$ (n-Hex +0.1 % FoA/EtOAc +2 % FoA, 2/1): 0.34.

Tripeptide (1a)

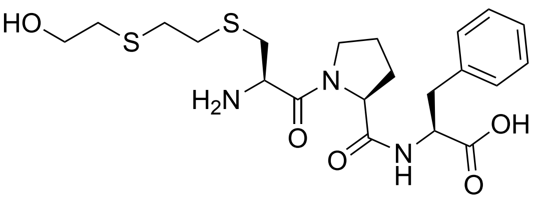

$C_{21}H_{31}N_3O_5S_2$
MW: 469.62
1a

Resin:	H-*L*-Phe-2CT
Scale:	0.4 mmol
Vial:	30 mL
Equivalents:	4.0 equiv
Amino Acids:	Fmoc-Pro-OH (0.5 M in DMF), **18a** (0.5 M in NMP)
Coupling:	DIC and Oxyma (both 0.5 M in DMF), ambient conditions for 60 min
Fmoc Deprotection:	5 % Piperazine in DMF (2 × 9 mL)
Cleavage Cocktail:	TFA/TIPS/CH_2Cl_2 (1/3/96, v/v/v)

Peptide-synthesis according to **GPSPPS-B** using parameters as indicated above. *Cleavage:* In a round-bottomed flask equipped with a stir bar, cleavage cocktail (15 mL) was added and the slurry stirred for 30 min. After filtration through a sintered glass funnel, cleavage cocktail (2×15 mL) was added to the collected resin in the funnel and the resulting slurry let elute for 30 min. This was repeated once. The collected filtrates were combined and concentrated under reduced pressure. Purification via flash column chromatography (Isolera Sfar HC C18 Duo 12 g, 12 mL min^{-1}, liquid loading, H_2O + 0.1 % FoA/CH_3CN + 0.1 % FoA (A/B), 5 % B (4.0 CV), 5 % – 40 % B (25.0 CV)) and lyophilization afforded the title compound as a white fluffy solid (128 mg, 0.27 mmol, 68 %).
^{1}**H-NMR** (400 MHz, D_2O) δ 7.44 – 7.22 (m, 5H), 4.67 – 4.53 (m, 1H), 4.53 – 4.39 (m, 2H), 3.81 – 3.54 (m, 4H), 3.52 – 3.24 (m, 1H), 3.21 – 3.11 (m, 1H), 3.09 – 2.89 (m, 2H), 2.85 (q, *J* = 2.0 Hz, 3H), 2.82 – 2.71 (m, 3H), 2.33 – 2.16 (m, 1H), 2.06 – 1.80 (m, 3H). ^{13}**C-NMR** (101 MHz, D_2O) δ 177.5, 176.9, 172.3, 167.3, 137.9, 137.2, 129.4, 129.1, 128.6, 128.6, 126.9, 60.9, 60.3, 55.7, 50.9, 48.0, 37.3, 33.5, 31.7, 31.4, 31.0, 29.0, 24.6, 21.7. **LC-HRMS** (Discovery HS C18, ESI/qTOF) calc. for $C_{21}H_{32}N_3O_5S_2^+$ [M + H]$^+$: 470.1778 m/z; found 470.1775 m/z. **FT-IR** (neat) [cm^{-1}]: 3331 (w), 2923 (w), 2877 (w), 1640 (vs), 1595 (s), 1532 (s), 1453 (m), 1383 (m), 1271 (w), 1200 (w), 1182 (w), 1128 (w), 1045 (w), 700 (m). **mp**: 143.1 °C (decomp.)

Alcohol (S21)

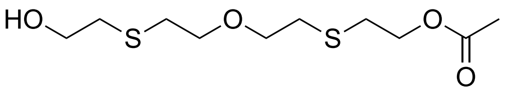

$C_{10}H_{20}O_4S_2$
MW: 268.39
S21

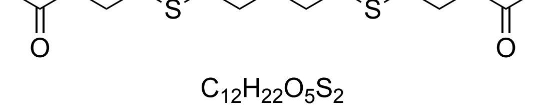

$C_{12}H_{22}O_5S_2$
MW: 310.42
S21'

According to **GPF** using diol 3,9-dithia-6-oxa-undecane-1,11-diol (25.0 mmol). Purification via column chromatography (Isolera Sfar HC Duo 100 g, 100 mL min^{-1}, n-Hex/EtOAc, 10 % – 40 % EtOAc (5.0 CV), 40 % – 70 % EtOAc (8.0 CV), 70 % EtOAc (5.0 CV)) to afford the title compound as a colorless liquid (2.97 g, 11.0 mmol, 44 %) and undesired bis-protected side-product **S21'** as a colorless liquid (1.58 g, 5.0 mmol, 20 %).
^{1}**H-NMR** (400 MHz, $CDCl_3$) δ 4.23 (t, *J* = 6.9 Hz, 2H), 3.75 (t, *J* = 5.9 Hz, 2H), 3.66 (td, *J* = 6.4, 2.2 Hz, 4H), 2.84 – 2.71 (m, 8H), 2.54 (s, 1H), 2.07 (s, 3H). ^{13}**C-NMR** (101 MHz, $CDCl_3$) δ 170.9, 70.9, 70.8, 63.6, 60.9, 36.0, 31.8, 31.5, 31.0, 20.9. **LC-HRMS** (Discovery HS C18, ESI/qTOF) calc. for $C_{10}H_{21}O_4S_2^+$ [M + H]$^+$: 269.0876 m/z; found 269.0876 m/z. **FT-IR** (neat) [cm^{-1}]: 3428 (br), 2920 (w), 2866 (w), 1736 (s), 1381 (m), 1361 (m), 1227 (s), 1104 (m), 1029 (s). **R**$_f$ (n-Hex/EtOAc, 2/1): 0.08.
Bisprotected Side-Product: ^{1}**H-NMR** (400 MHz, $CDCl_3$) δ 4.23 (t, *J* = 6.9 Hz, 4H), 3.65 (t, *J* = 6.5 Hz, 4H), 2.80 (t, *J* = 6.9 Hz, 4H), 2.76 (t, *J* = 6.5 Hz, 4H), 2.07 (s, 6H). ^{13}**C-NMR** (101 MHz, $CDCl_3$) δ 170.83, 70.84, 63.55, 31.82, 30.99, 20.92. **LC-HRMS** (Discovery HS C18, ESI/qTOF) calc. for $C_{12}H_{23}O_5S_2^+$ [M + H]$^+$: 311.0981 m/z;

found 311.0982 m/z. **FT-IR** (neat) [cm^{-1}]: 2924 (w), 2864 (w), 1735 (vs), 1381 (m), 1361 (m), 1222 (vs), 1106 (m), 1026 (s), 969 (m). **R$_f$** (n-Hex/EtOAc, 2/1): 0.35.

Alkyl Chloride (S22)

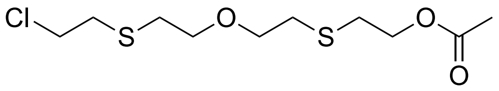

$C_{10}H_{19}ClO_3S_2$
MW: 286.83
S22

According to **GPH** using alcohol **S21** (10.0 mmol). Purification via flash column chromatography (Isolera Sfar HC Duo 50 g, 120 mL min^{-1}, n-Hex/EtOAc, 5 % – 29 % EtOAc (14.0 CV)) afforded the title compound as a colorless liquid (2.55 g, 8.8 mmol, 88 %).
^{1}**H-NMR** (400 MHz, CDCl$_3$) δ 4.23 (t, J = 6.9 Hz, 2H), 3.70 – 3.61 (m, 6H), 2.97 – 2.88 (m, 2H), 2.80 (t, J = 6.9 Hz, 2H), 2.76 (td, J = 6.5, 1.6 Hz, 4H), 2.07 (s, 3H). ^{13}**C-NMR** (101 MHz, CDCl$_3$) δ 170.8, 71.0, 70.9, 63.6, 43.2, 34.8, 32.0, 31.9, 31.0, 20.9. **LC-HRMS** (Discovery HS C18, ESI/qTOF) calc. for $C_{10}H_{20}ClO_3S_2^+$ [M + H]$^+$: 287.0534 m/z; found 287.0534 m/z. **FT-IR** (neat) [cm^{-1}]: 2959 (w), 2923 (w), 2863 (w), 1736 (vs), 1427 (w), 1381 (m), 1361 (m), 1225 (vs), 1105 (s), 1028 (s), 966 (w), 694 (m), 647 (m), 604 (m). **R$_f$** (n-Hex/EtOAc, 4/1): 0.50.

Cysteine Adduct (18c)

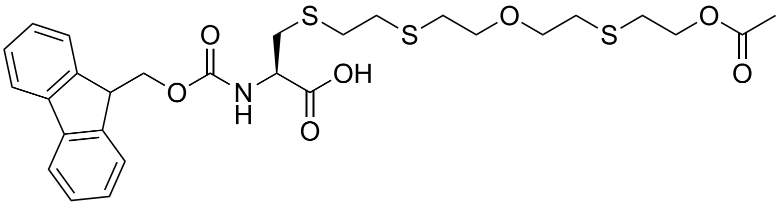

$C_{28}H_{35}NO_7S_3$
MW: 593.77
18c

According to **GPC** using thioether chloride **S22** (4.0 mmol) and sat. aq. NaHCO$_3$ (10 mL), CH$_3$CN (20 mL) and acetone (15 mL) as solvents. Purification via flash column chromatography (Isolera Sfar C18 D 60 g, 50 mL min^{-1}, H$_2$O + 0.1 % FoA/CH$_3$CN + 0.1 % FoA (A/B), 30 % – 40 % B (15 CV), 40 % – 60 % B (15 CV), 60 % B (10 CV)) afforded the title compound as a colorless oil (1.64 g, 2.7 mmol, 69 %).
^{1}**H-NMR** (400 MHz, CDCl$_3$) δ 7.77 (d, J = 7.5 Hz, 2H), 7.61 (d, J = 7.5 Hz, 2H), 7.44 – 7.37 (m, 2H), 7.32 (tt, J = 7.5, 1.3 Hz, 2H), 5.79 (d, J = 7.9 Hz, 1H), 4.71 – 4.62 (m, 1H), 4.41 (d, J = 7.1 Hz, 2H), 4.25 (t, J = 6.9 Hz, 3H), 3.72 – 3.58 (m, 4H), 3.12 (d, J = 4.9 Hz, 2H), 2.87 – 2.64 (m, 9H), 2.08 (s, 3H). ^{13}**C-NMR** (101 MHz, CDCl$_3$) δ 172.8, 171.8, 155.9, 143.8, 143.7, 141.3, 127.8, 127.1, 125.1, 120.0, 71.1, 70.9, 67.3, 63.9, 53.4, 47.1, 34.3, 32.7, 32.5, 31.8, 31.3, 30.9, 21.0. **LC-HRMS** (Discovery HS C18, ESI/qTOF) calc. for $C_{28}H_{36}NO_{67}S_3^+$ [M + H]$^+$: 594.1648 m/z; found 594.1654 m/z. **FT-IR** (neat) [cm^{-1}]: 3322 (w), 3066 (w), 2950 (w), 2923 (w), 2864 (w), 1715 (vs), 1517 (m), 1450 (m), 1421 (w), 1228 (vs), 1104 (m), 1045 (s), 1033 (s), 760 (m), 740 (s), 621 (m). **R$_f$** (H$_2$O +0.1 % AcOH/CH$_3$CN +0.1 % AcOH, 3/7): 0.50.

Tripeptide (1c)

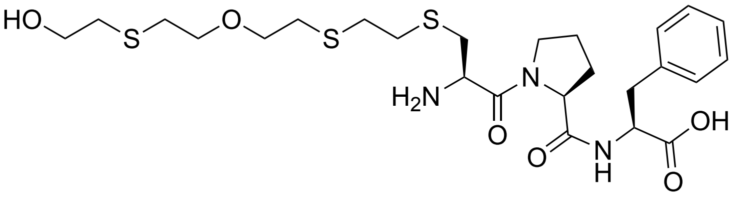

$C_{25}H_{39}N_3O_6S_3$
MW: 573.78

1c

Resin:	H-*L*-Phe-2CT
Scale:	0.4 mmol
Vial:	30 mL
Equivalents:	4.0 equiv
Amino Acids:	Fmoc-Pro-OH (0.5 M in DMF), **18c** (0.5 M in NMP)
Coupling:	DIC and Oxyma (both 0.5 M in DMF), ambient conditions for 60 min
Fmoc Deprotection:	5 % Piperazine in DMF (2 × 9 mL)
Cleavage Cocktail:	TFA/TIPS/CH_2Cl_2 (1/3/96, v/v/v)

Peptide-synthesis according to **GPSPPS-B** using parameters as indicated above. *Cleavage:* In a round-bottomed flask equipped with a stir bar, cleavage cocktail (15 mL) was added and the slurry stirred for 30 min. After filtration through a sintered glass funnel, cleavage cocktail (2×15 mL) was added to the collected resin in the funnel and the resulting slurry let elute for 30 min. The collected filtrates were combined and concentrated under reduced pressure. Residual TIPS was removed by co-evaporation with CH_3CN. Purification via flash column chromatography (Isolera Sfar HC C18 Duo 12 g, 12 mL min^{-1}, liquid loading, H_2O + 0.1 % FoA/CH_3CN + 0.1 % FoA, 5 % CH_3CN + 0.1 % FoA (3.0 CV), 5 % – 30 % CH_3CN + 0.1 % FoA (10.0 CV), 30.0 % CH_3CN + 0.1 % FoA (2.0 CV)), several co-evaporations of the aqueous phase with CH_2Cl_2 and subsequent lyophilization afforded the title compound as a white fluffy solid (117 mg, 0.22 mmol, 55 %).
^{1}**H-NMR** (400 MHz, DMSO-*d*^{6}/D_2O, 5/1) δ 7.54 – 7.34 (m, 5H), 4.53 (td, *J* = 4.1, 1.7 Hz, 1H), 4.44 – 4.29 (m, 1H), 3.77 – 3.67 (m, 7H), 3.67 – 3.48 (m, 2H), 3.35 (dd, *J* = 14.4, 4.7 Hz, 1H), 3.30 – 3.02 (m, 3H), 2.92 – 2.81 (m, 8H), 2.78 (t, *J* = 6.8 Hz, 2H), 2.38 (ddt, *J* = 11.5, 8.3, 4.6 Hz, 1H), 2.14 – 1.89 (m, 3H). ^{13}**C-NMR** (101 MHz, DMSO-*d*^{6}/D_2O, 5/1) δ 171.0, 169.9, 165.1, 137.0, 130.0, 129.2, 127.5, 70.6, 70.5, 61.4, 59.2, 55.8, 55.7, 45.5, 36.9, 34.7, 33.3, 33.2, 32.6, 31.6, 31.3, 28.9, 22.4. **LC-HRMS** (Discovery HS C18, ESI/qTOF) calc. for $C_{25}H_{40}N_3O_6S_3^+$ $[M + H]^+$: 574.2074 m/z; found 574.2081 m/z. **FT-IR** (neat) [cm^{-1}]: 3327 (w), 2950 (w), 2920 (w), 2873 (w), 1639 (vs), 1587 (s), 1532 (s), 1453 (m), 1383 (m), 1361 (m), 1274 (w), 1198 (m), 1105 (m), 1039 (m), 836 (w), 756 (m), 699 (s).

1.3.4.3 Failed Approaches

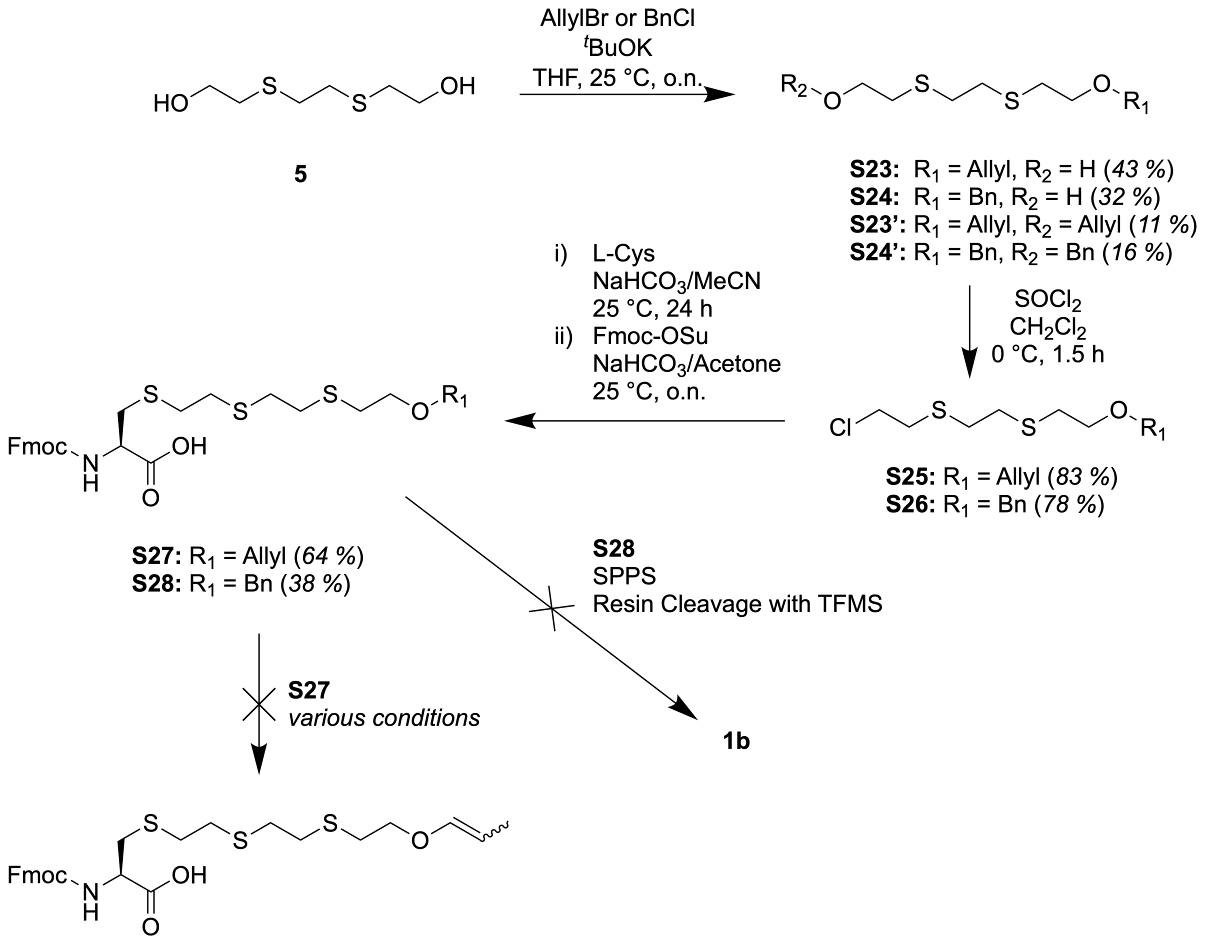

Scheme S5 Synthesis attempt using allyl or benzyl protection of the alcohol. However both failed: Allyl was unsuccessfully converted acid labile vinyl protection using various conditions and, on the other hand, treating the resin with TFMS to remove simoultaneously the benzyl protection lead to full degradation of the peptide.

Alcohol (S23)

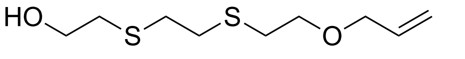

$C_9H_{18}O_2S_2$
MW: 222.36
S23

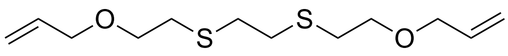

$C_{12}H_{22}O_2S_2$
MW: 262.43
S23'

A heat-gun dried two-necked round-bottomed flask equipped with a stir bar, reflux condenser and septum was charged with diol **5** (10.0 mmol, 1.0 equiv) and evacuated and flushed with Ar three times. After the addition of THF (50 mL), the suspension was stirred until homogeneous and cooled to 0 °C. Then, NaO^{t}Bu (12.0 mmol, 1.2 equiv) was added and the suspension stirred at 0 °C for 10 min. Afterwards the ice bath was removed and the reaction mixture allowed to warm up to room temperature. After the addition of AllylBr (12.0 mmol, 1.2 equiv) the reaction mixture was stirred at 60 °C for 1.5 d. The solution was quenched with 10 % aq. NH_4Cl (40 mL) and extracted with EtOAc (3 × 100 mL). The combined organic layers were dried over Na_2SO_4, concentrated under reduced pressure and purified by column chromatography to afford the title compound. Purification via flash column chromatography (Isolera Sfar HC Duo 25 g, 80 mL min^{-1}, n-Hex/EtOAc, 4 % EtOAc (1.0 CV), 4 % – 10 % EtOAc (6.5 CV), 10 % – 15 % EtOAc (13 CV), 15 % – 60 % EtOAc (6.0 CV), 60 % EtOAc (3.0 CV)) afforded the title compound as a colorless oil (961 mg, 4.32 mmol, 43 %) and undesired bisprotected side-product **S23'** as a colorless oil (296 mg, 1.13 mmol, 11 %).

^{1}**H-NMR** (400 MHz, $CDCl_3$) δ 5.91 (ddt, J = 17.2, 10.4, 5.7 Hz, 1H), 5.29 (dq, J = 17.2, 1.7 Hz, 1H), 5.20 (dq, J = 10.4, 1.4 Hz, 1H), 4.01 (dt, J = 5.6, 1.4 Hz, 2H), 3.74 (q, J = 5.9 Hz, 2H), 3.63 (t, J = 6.6 Hz, 2H), 2.86 – 2.71 (m, 8H). ^{13}**C-NMR** (101 MHz, $CDCl_3$) δ 134.5, 117.3, 72.0, 70.1, 60.6, 35.4, 32.8, 31.9, 31.8. **LC-HRMS** (Discovery HS C18, ESI/qTOF) calc. for $C_9H_{19}O_2S_2^+$ $[M+H]^+$: 223.0821 m/z; found 223.0821 m/z. **FT-IR** (neat) [cm^{-1}]:

3402 (br), 2917 (m), 2857 (m), 1646 (w), 1421 (m), 1346 (w), 1271 (w), 1202 (w), 1091 (vs), 1045 (vs), 1002 (s), 928 (s). **R**$_f$ (n-Hex/EtOAc, 2/1): 0.25.
Bisprotected Side-Product: ^{1}**H-NMR** (400 MHz, $CDCl_3$) δ 5.91 (ddt, *J* = 17.2, 10.3, 5.6 Hz, 2H), 5.28 (dq, *J* = 17.2, 1.6 Hz, 2H), 5.19 (dq, *J* = 10.4, 1.4 Hz, 2H), 4.01 (dt, *J* = 5.6, 1.4 Hz, 4H), 3.61 (t, *J* = 6.7 Hz, 4H), 2.79 (s, 4H), 2.75 (t, *J* = 6.7 Hz, 4H). ^{13}**C-NMR** (101 MHz, $CDCl_3$) δ 134.5, 117.2, 72.0, 70.0, 32.9, 31.7. **LC-HRMS** (Discovery HS C18, ESI/qTOF) calc. for $C_{12}H_{23}O_2S_2^+$ [M + H]$^+$: 263.1134 m/z; found 263.1135 m/z. **FT-IR** (neat) [cm^{-1}]: 3077 (w), 2916 (w), 2851 (m), 1646 (w), 1421 (m), 1346 (m), 1271 (m), 1201 (m), 1092 (vs), 992 (m), 925 (s). **R**$_f$ (n-Hex/EtOAc, 8/1): 0.32.

Alcohol (S24)

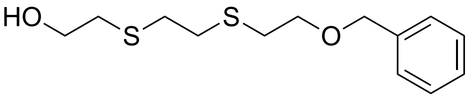

$C_{13}H_{20}O_2S_2$
MW: 272.42
S24

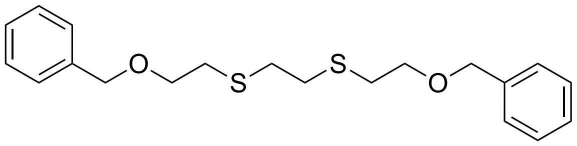

$C_{20}H_{26}O_2S_2$
MW: 362.55
S24'

A heat-gun dried two-necked round-bottomed flask equipped with a stir bar, reflux condenser and septum was charged with diol **5** (10.0 mmol, 1.0 equiv) and evacuated and flushed with Ar three times. After the addition of THF (50 mL), the suspension was stirred until homogeneous and cooled to 0 °C. Then, NaO^{t}Bu (12.0 mmol, 1.2 equiv) was added and the suspension stirred at 0 °C for 10 min. Afterwards the ice bath was removed and the reaction mixture allowed to warm up to room temperature. After the addition of BnCl (12.0 mmol, 1.2 equiv) the reaction mixture was stirred at 60 °C for 1.5 d.
The solution was quenched with 10 % aq. NH_4Cl (40 mL) and extracted with EtOAc (3 × 100 mL). The combined organic layers were dried over Na_2SO_4, concentrated under reduced pressure and purified by column chromatography to afford the title compound. Purification via flash column chromatography (Isolera Sfar HC Duo 25 g, 80 mL min^{-1}, n-Hex/EtOAc, 4 % EtOAc (1.0 CV), 4 % – 10 % EtOAc (6.5 CV), 10 % – 15 % EtOAc (13 CV), 15 % – 60 % EtOAc (6.0 CV), 60 % EtOAc (3.0 CV)) afforded the title compound as a colorless oil (884 mg, 3.25 mmol, 32 %) and undesired bisprotected side-product **S24'** as a colorless oil (583 mg, 1.61 mmol, 16 %).
^{1}**H-NMR** (400 MHz, $CDCl_3$) δ 7.42 – 7.23 (m, 5H), 4.54 (d, *J* = 1.9 Hz, 2H), 3.67 (dtd, *J* = 13.0, 6.2, 2.9 Hz, 4H), 2.83 – 2.72 (m, 6H), 2.70 (td, *J* = 5.9, 1.9 Hz, 2H), 2.25 (t, *J* = 5.2 Hz, 1H). ^{13}**C-NMR** (101 MHz, $CDCl_3$) δ 138.0, 128.4, 127.8, 127.7, 73.2, 70.2, 60.6, 35.3, 32.7, 31.8, 31.8. **LC-HRMS** (Discovery HS C18, ESI/qTOF) calc. for $C_{13}H_{21}O_2S_2^+$ [M + H]$^+$: 273.0977 m/z; found 273.0978 m/z. **FT-IR** (neat) [cm^{-1}]: 3410 (br), 2919 (m), 2860 (m), 1496 (w), 1453 (m), 1423 (m), 1408 (m), 1360 (m), 1288 (m), 1270 (m), 1204 (m), 1096 (s), 1074 (s), 1046 (s), 1028 (m), 946 (w), 739 (m), 699 (m). **R**$_f$ (n-Hex/EtOAc, 2/1): 0.26.
Bisprotected Side-Product: ^{1}**H-NMR** (400 MHz, $CDCl_3$) δ 7.41 – 7.23 (m, 10H), 4.53 (s, 4H), 3.63 (t, *J* = 6.6 Hz, 4H), 2.77 (s, 4H), 2.74 (t, *J* = 6.6 Hz, 4H). ^{13}**C-NMR** (101 MHz, $CDCl_3$) δ 138.1, 128.5, 127.7, 127.7, 73.1, 70.0, 32.7, 31.8. **LC-HRMS** (Discovery HS C18, ESI/qTOF) calc. for $C_{20}H_{27}O_2S_2^+$ [M + H]$^+$: 363.1447 m/z; found 363.1447 m/z. **FT-IR** (neat) [cm^{-1}]: 3088 (w), 3062 (w), 3029 (w), 2916 (m), 2854 (m), 1496 (m), 1453 (m), 1360 (m), 1290 (w), 1204 (m), 1098 (s), 1028 (m), 950 (w), 909 (w), 736 (m), 697 (m). **R**$_f$ (n-Hex/EtOAc, 8/1): 0.27.

Alkyl Chloride (S25)

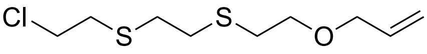

$C_9H_{17}ClOS_2$
MW: 240.80
S25

According to **GPH** using alcohol **S23** (3.4 mmol). Purification via flash column chromatography (Isolera SNAP Ultra 10 g, 36 mL min^{-1}, n-Hex/EtOAc, 0 % – 5 % EtOAc (8.0 CV), 5 % – 15 % EtOAc (3.0 CV), 15 % – 40 % EtOAc (3.0 CV)) afforded the title compound as a colorless liquid (686 mg, 2.86 mmol, 83 %).
^{1}**H-NMR** (400 MHz, $CDCl_3$) δ 5.91 (ddt, *J* = 17.2, 10.3, 5.6 Hz, 1H), 5.29 (dq, *J* = 17.3, 1.7 Hz, 1H), 5.20 (dq, *J* = 10.3, 1.4 Hz, 1H), 4.01 (dt, *J* = 5.7, 1.4 Hz, 2H), 3.70 – 3.59 (m, 4H), 2.93 – 2.85 (m, 2H), 2.80 (s, 4H), 2.75 (t, *J* = 6.5 Hz, 2H). ^{13}**C-NMR** (101 MHz, $CDCl_3$) δ 134.6, 117.4, 72.1, 70.2, 43.2, 34.4, 32.9, 32.6, 31.9. **LC-HRMS** (Discovery HS C18, ESI/qTOF) calc. for $C_9H_{18}ClOS_2^+$ [M + H]$^+$: 241.0482 m/z; found 241.0483 m/z. **FT-IR** (neat) [cm^{-1}]: 3082 (w), 2917 (m), 2848 (m), 1646 (w), 1421 (m), 1346 (w), 1291 (m), 1271 (m), 1200 (m), 1092 (vs), 1042 (m), 992 (m), 926 (s), 687 (m), 650 (m). **R**$_f$ (n-Hex/EtOAc, 30/1): 0.23.

Alkyl Chloride (S26)

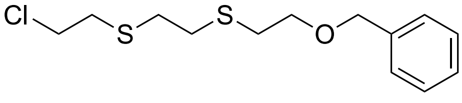

$C_{13}H_{19}ClOS_2$
MW: 290.86
S26

According to **GPH** using alcohol **S24** (2.9 mmol). Purification via flash column chromatography (Isolera SNAP Ultra 10 g, 36 mL min^{-1}, n-Hex/EtOAc, 0 % – 5 % EtOAc (8.0 CV), 5 % – 15 % EtOAc (3.0 CV), 15 % – 40 % EtOAc (3.0 CV)) afforded the title compound as a colorless liquid (654 mg, 2.25 mmol, 78 %).
^{1}**H-NMR** (400 MHz, $CDCl_3$) δ 7.40 – 7.23 (m, 5H), 4.54 (s, 2H), 3.66 (t, *J* = 6.5 Hz, 2H), 3.63 – 3.57 (m, 2H), 2.89 – 2.82 (m, 2H), 2.80 – 2.74 (m, 6H). ^{13}**C-NMR** (101 MHz, $CDCl_3$) δ 138.0, 128.4, 127.7, 127.7, 73.2, 70.1, 43.1, 34.3, 32.7, 32.5, 31.9. **LC-HRMS** (Discovery HS C18, ESI/qTOF) calc. for $C_{13}H_{20}ClOS_2^+$ $[M+H]^+$: 291.0639 m/z; found 291.0640 m/z. 3029 (w), 2919 (w), 2858 (w), 1496 (w), 1453 (m), 1421 (m), 1360 (m), 1291 (m), 1270 (m), 1201 (m), 1098 (s), 1028 (m), 949 (w), 909 (w), 847 (w), 737 (s), 696 (vs), 647 (m), 608 (m). **R**$_f$ (n-Hex/EtOAc, 25/1): 0.31.

Cysteine Derivative (S27)

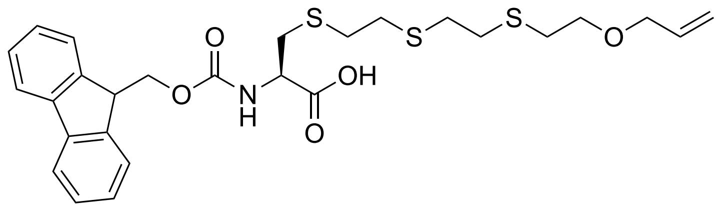

$C_{27}H_{33}NO_5S_3$
MW: 547.74
S27

A round-bottomed flask equipped with a stir bar was charged with *L*-Cys (2.41 mmol, 1.0 equiv) and sat. aq. $NaHCO_3$ (14 mL) and the suspension stirred until homogenous. Then, a solution of **S25** (2.64 mmol, 1.1 equiv) in CH_3CN (14 mL) was added and the reaction mixture stirred at room temperature for 24 h. To the formed white slurry, a solution of Fmoc-OSu (3.60 mmol, 1.5 equiv) in acetone (10 mL) was added dropwise, the pH adjusted between 9 – 10 with additional sat. aq. $NaHCO_3$ if necessary and the solution stirred at room temperature overnight.
The reaction mixture was quenched with 1 M HCl to pH 1 and extracted with EtOAc (3 × 100 mL). The organic layers were combined, dried over Na_2SO_4, filtered and concentrated under reduced pressure. Purification via flash column chromatography (Isolera Sfar C18 Duo 30 g, 25 mL min^{-1}, H_2O + 0.1 % FoA/CH_3CN + 0.1 % FoA, 40 % – 50 % CH_3CN + 0.1 % FoA (20.0 CV), 50 % CH_3CN + 0.1 % FoA (14.5 CV)) and lyophilization afforded the title compound as a white solid (847 mg, 1.55 mmol, 64 %).
^{1}**H-NMR** (400 MHz, CD_3CN) δ 7.83 (d, *J* = 7.6 Hz, 2H), 7.68 (d, *J* = 7.5 Hz, 2H), 7.42 (td, *J* = 7.5, 1.2 Hz, 2H), 7.34 (tt, *J* = 7.4, 1.3 Hz, 2H), 6.15 (d, *J* = 8.3 Hz, 1H), 5.89 (ddt, *J* = 17.2, 10.7, 5.4 Hz, 1H), 5.25 (dh, *J* = 17.2, 1.8 Hz, 1H), 5.17 – 5.09 (m, 1H), 4.43 – 4.29 (m, 3H), 4.25 (t, *J* = 6.9 Hz, 1H), 3.94 (dt, *J* = 5.5, 1.5 Hz, 2H), 3.55 (t, *J* = 6.5 Hz, 2H), 3.05 (dd, *J* = 14.0, 4.8 Hz, 1H), 2.90 (dd, *J* = 14.0, 7.7 Hz, 1H), 2.81 – 2.63 (m, 10H). ^{13}**C-NMR** (101 MHz, CD_3CN) δ 172.3, 156.9, 145.0, 145.0, 142.1, 136.1, 128.7, 128.1, 128.1, 126.2, 121.0, 116.9, 72.2, 70.8, 67.5, 54.8, 47.9, 34.3, 33.3, 33.2, 32.7, 32.5, 32.1. **LC-HRMS** (Discovery HS C18, ESI/qTOF) calc. for $C_{27}H_{34}NO_5S_3^+$ $[M+H]^+$: 548.1594 m/z; found 548.1593 m/z. **FT-IR** (neat) [cm^{-1}]: 3226 (m), 3063 (m), 2926 (m), 1741 (vs), 1680 (m), 1656 (m), 1547 (s), 1450 (m), 1421 (m), 1391 (m), 1344 (m), 1284 (vs), 1247 (m), 1220 (m), 1192 (m), 1157 (m), 1138 (m), 1052 (vs), 1016 (m), 998 (m), 949 (m), 893 (m), 845 (m), 756 (m), 737 (vs), 711 (m), 683 (m). **mp**: 70 °C – 74 °C. **R**$_f$ (H_2O +0.1 % AcOH/CH_3CN +0.1 % AcOH, 3/7): 0.43.

Cysteine Derivative (S28)

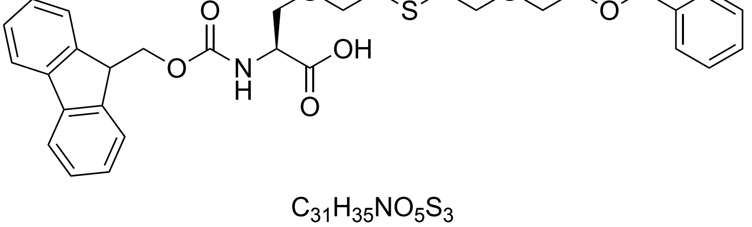

$C_{31}H_{35}NO_5S_3$
MW: 597.80
S28

A round-bottomed flask equipped with a stir bar was charged with *L*-Cys (1.19 mmol, 1.0 equiv) and sat. aq. $NaHCO_3$ (6 mL) and the suspension stirred until homogenous. Then, a solution of **S26** (1.31 mmol, 1.1 equiv) in CH_3CN (6 mL) was added and the reaction mixture stirred at room temperature for 24 h. To the formed white slurry, a solution of Fmoc-OSu (1.78 mmol, 1.5 equiv) in acetone (5 mL) was added dropwise, adjusted to pH 9 – 10 with additional sat. aq. $NaHCO_3$ if necessary and the solution stirred at room temperature overnight.

The reaction mixture was quenched with 1 M HCl to pH 1 and EtOAc (50 mL). The emulsion was transferred into a separatory funnel and brine (4 mL) was added. The phases were separated and the aqueous layer extracted with EtOAc (2 × 50 mL). The combined organic layers were dried over Na_2SO_4, filtered and concentrated under reduced pressure. Purification via flash column chromatography (Isolera Sfar C18 Duo 30 g, 25 mL min^{-1}, H_2O + 0.1 % FoA/CH_3CN + 0.1 % FoA, 40 % – 50 % CH_3CN + 0.1 % FoA (20.0 CV), 50 % CH_3CN + 0.1 % FoA (14.5 CV)) and lyophilization afforded the title compound as a white solid (275 mg, 0.46 mmol, 38 %).
^{1}**H-NMR** (400 MHz, CD_3CN) δ 7.83 (d, J = 7.5 Hz, 2H), 7.68 (d, J = 7.5 Hz, 2H), 7.42 (td, J = 7.5, 1.2 Hz, 2H), 7.37 (– 7.24 (m, 8H), 6.12 (d, J = 8.2 Hz, 1H), 4.48 (s, 2H), 4.41 (– 4.30 (m, 3H), 4.25 (t, J = 6.9 Hz, 1H), 3.61 (t, J = 6.4 Hz, 2H), 3.04 (dd, J = 14.0, 4.8 Hz, 1H), 2.88 (dd, J = 13.9, 7.8 Hz, 1H), 2.73 (d, J = 6.8 Hz, 11H). ^{13}**C-NMR** (101 MHz, CD_3CN) δ 172.3, 156.9, 145.0, 145.0, 142.1, 139.6, 129.3, 128.7, 128.6, 128.5, 128.1, 128.1, 126.2, 121.0, 73.3, 71.0, 67.5, 54.8, 48.0, 34.3, 33.3, 33.2, 32.7, 32.5, 32.2. **LC-HRMS** (Discovery HS C18, ESI/qTOF) calc. for $C_{31}H_{36}NO_5S_3^+$ $[M + H]^+$: 598.1750 m/z; found 598.1765 m/z. **FT-IR** (neat) [cm^{-1}]: 3325 (m), 3037 (w), 2923 (w), 1689 (s), 1533 (m), 1450 (m), 1421 (m), 1268 (m), 1194 (m), 1102 (m), 1084 (m), 1045 (m), 988 (w), 938 (w), 910 (w), 757 (m), 736 (s), 696 (m), 620 (m). **mp**: 53 °C (decomp.). **R**$_f$ (H_2O +0.1 % AcOH/CH_3CN +0.1 % AcOH, 3/7): 0.30.

Bibliography

[1] Federal Office of Public Health (FOPH), Authorisation of Research Projects, can be found under https://www.bag.admin.ch/en/human-research-approval-of-research-projects **2025**.

[2] N. H. Tran, X. Zhang, L. Xin, B. Shan, M. Li, *Proc. Natl. Acad. Sci. U. S. A.* **2017**, *114*, 8247.

[3] N. H. Tran, R. Qiao, L. Xin, X. Chen, C. Liu, X. Zhang, B. Shan, A. Ghodsi, M. Li, *Nat. Methods.* **2019**, *16*, 63.

[4] V. Demichev, C. B. Messner, S. I. Vernardis, K. S. Lilley, M. Ralser, *Nat. Methods.* **2020**, *17*, 41.

[5] R Core Team, *R: A Language and Environment for Statistical Computing*, R Foundation for Statistical Computing, Vienna, Austria **2022**.

[6] B. MacLean, D. M. Tomazela, N. Shulman, M. Chambers, G. L. Finney, B. Frewen, R. Kern, D. L. Tabb, D. C. Liebler, M. J. MacCoss, *Bioinformatics* **2010**, *26*, 966.

[7] H. Wickham, M. Averick, J. Bryan, W. Chang, L. D. McGowan, R. François, G. Grolemund, A. Hayes, L. Henry, J. Hester, M. Kuhn, T. L. Pedersen, E. Miller, S. M. Bache, K. Müller, J. Ooms, D. Robinson, D. P. Seidel, V. Spinu, K. Takahashi, D. Vaughan, C. Wilke, K. Woo, H. Yutani, *Journal of Open Source Software* **2019**, *4*, 1686.

[8] J. D. Storey, A. J. Bass, A. Dabney, D. Robinson, *qvalue: Q-value estimation for false discovery rate control* **2023**, r package version 2.34.0.

[9] A. Liaw, M. Wiener, *R News* **2002**, *2*, 18.

[10] R. Kolde, *pheatmap: Pretty Heatmaps* **2019**, r package version 1.0.12.

[11] H. Wickham, *ggplot2: Elegant Graphics for Data Analysis*, Springer-Verlag New York **2016**.

[12] A. Kassambara, *ggpubr: 'ggplot2' Based Publication Ready Plots* **2023**, r package version 0.6.0.

[13] Y. Tang, M. Horikoshi, W. Li, *The R Journal* **2016**, *8*, 474.

[14] C.-H. Gao, A. Dusa, *ggVennDiagram: A 'ggplot2' Implement of Venn Diagram* **2024**, r package version 1.5.2.

[15] H. Wickham, T. L. Pedersen, D. Seidel, *scales: Scale Functions for Visualization* **2023**, r package version 1.3.0.

[16] E. Neuwirth, *RColorBrewer: ColorBrewer Palettes* **2022**, r package version 1.1-3.

[17] K. Goldmann, M. Lewis, *volcano3D: 3D Volcano Plots and Polar Plots for Three-Class Data* **2024**, https://katrionagoldmann.github.io/volcano3D/index.html, https://github.com/KatrionaGoldmann/volcano3D.

[18] E. C. Meng, T. D. Goddard, E. F. Pettersen, G. S. Couch, Z. J. Pearson, J. H. Morris, T. E. Ferrin, *Protein Sci* **2023**, *32*, e4792.

[19] C. S. Hughes, S. Moggridge, T. Muller, P. H. Sorensen, G. B. Morin, J. Krijgsveld, *Nat. Protoc.* **2019**, *14*, 68.

[20] M. Leutert, R. A. Rodriguez-Mias, N. K. Fukuda, J. Villen, *Mol. Syst. Biol.* **2019**, *15*, e9021.

[21] W. E. Johnson, C. Li, A. Rabinovic, *Biostatistics* **2007**, *8*, 118.

[22] E. Gómez-Caballero, R. Martínez-Álvarez, M. A. Sierra, *J. Org. Chem.* **2018**, *83*, 12432.

[23] F. Sebak, J. Szolomajer, N. Papp, G. K. Toth, A. Bodor, *Front. Biosci. (Landmark Ed)* **2023**, *28*, 127.

[24] S. Mattsson, M. Dahlström, S. Karlsson, *Tetrahedron Lett.* **2007**, *48*, 2497.

[25] M. L. Di Gioia, A. Leggio, A. Le Pera, C. Siciliano, A. Liguori, G. Sindona, *J. Pept. Res.* **2004**, *63*, 383.

[26] I. M. Daubit, J. Wolf, N. Metzler-Nolte, *J. Organomet. Chem.* **2020**, *909*.

[27] K. Nakane, S. Sato, T. Niwa, M. Tsushima, S. Tomoshige, H. Taguchi, M. Ishikawa, H. Nakamura, *J. Am. Chem. Soc.* **2021**, *143*, 7726.

[28] M. Berthet, J. Martinez, I. Parrot, *Biopolymers* **2017**, *108*.

[29] H. E. Revercomb, E. A. Mason, *Anal. Chem.* **1975**, *47*, 970.

[30] B. Chen, Q. Zhang, Z. Ren, T. Zhang, H. Yu, C. Liu, Y. Yang, P. Xu, S. Liu, *Anal. Bioanal. Chem.* **2022**, *414*, 4179.

[31] M. Siegert, *Mass spectrometric detection and characterization of covalent reaction products between the chemical warfare agent sulfur mustard and human serum albumin and small molecules*, Dissertation, Humboldt-Universität zu Berlin **2023**.

[32] D. Noort, A. G. Hulst, L. P. A. de Jong, H. P. Benschop, *Chem. Res. Toxicol.* **1999**, *12*, 715.

[33] F. Gandor, M. Gawlik, H. Thiermann, H. John, *J. Anal. Toxicol.* **2015**, *39*, 270.

[34] A. Richter, M. Siegert, H. Thiermann, H. John, *Anal. Bioanal. Chem.* **2021**, *413*, 4907.

[35] H. John, M. Koller, F. Worek, H. Thiermann, M. Siegert, *Arch. Toxicol.* **2019**, *93*, 1881.

[36] M.-M. Blum, W. Schmeißer, M. Dentzel, H. Thiermann, H. John, *Anal. Bioanal. Chem.* **2024**.

2 Appendix

2.1 Supplementary Tables

Table S10 List of peptides which were identified by de novo sequencing in PEAKS and subsequent justification by identification of the three qualifiers in low-mass dda-PASEF mode. ^{a}Calculated according to Mason-Schamp[29] at 305 K.

Protein	Modification Site	Peptide	Formula	Exact Mass [u]	Precursor [m/z]	z	IM [V s cm^{-1}]	CCS [Å^2]
P01024	E759	SNLDEDIIAEE(HETETE)NIVSR	$C_{81}H_{137}N_{21}O_{32}S_2$	1979.92	990.96	2	1.17	474.30
P01024	E1486	VYAYYNLE(HETETE)ESC(CAM)TR	$C_{79}H_{118}N_{18}O_{26}S_3$	1830.76	916.38	2	1.13	459.00
P02647	E235	VSFLSALEE(HETETE)YTK	$C_{70}H_{111}N_{13}O_{22}S_2$	1549.74	775.87	2	1.04	425.80
P02647	E234	VSFLSALE(HETETE)EYTK	$C_{70}H_{111}N_{13}O_{22}S_2$	1549.74	775.87	2	1.03	419.90
P02768	E396	QNC(CAM)ELFE(HETETE)QLGEYK	$C_{78}H_{120}N_{18}O_{26}S_3$	1820.78	911.39	2	1.13	458.60
P02768	H67	SLH(HETETE)TLFGDK	$C_{52}H_{84}N_{12}O_{15}S_2$	1180.56	591.28	2	0.91	371.10
P02768	H74	SLHTLFGD(HETETE)K	$C_{52}H_{84}N_{12}O_{15}S_2$	1180.56	591.2892	2	0.92	374.7
P02768	D1	D(HETETE)AHKSEVAHR	$C_{53}H_{88}N_{18}O_{17}S_2$	1312.60	438.53	3	0.73	452.70
P02768	D1	D(HETETE)AHK	$C_{25}H_{43}N_7O_8S_2$	633.26	317.63	2	0.66	274.10
P02768	E17	FKDLGEE(HETETE)NFK	$C_{62}H_{95}N_{13}O_{19}S_2$	1389.63	695.82	2	0.99	405.70
P02768	E57	TC(CAM)VADE(HETETE)SAENC(CAM)DK	$C_{62}H_{103}N_{17}O_{28}S_4$	1661.60	831.80	2	1.04	422.10
P02768	E60	TC(CAM)VADESAE(HETETE)NC(CAM)DK	$C_{62}H_{103}N_{17}O_{28}S_4$	1661.60	831.80	2	1.03	420.30
P02768	D63	TC(CAM)VADESAENC(CAM)D(HETETE)K	$C_{62}H_{103}N_{17}O_{28}S_4$	1661.60	831.80	2	1.03	419.40
P02768	E86	ETYGE(HETETE)MADC(CAM)C(CAM)AK	$C_{62}H_{99}N_{15}O_{24}S_5$	1597.56	799.78	2	1.02	416.00
P02768	E167	AAFTE(HETETE)C(CAM)C(CAM)QAADK	$C_{61}H_{98}N_{16}O_{22}S_4$	1534.59	768.30	2	1.02	417.20
P02768	D173	AAFTEC(CAM)C(CAM)QAAD(HETETE)K	$C_{61}H_{98}N_{16}O_{22}S_4$	1534.59	768.30	2	1.02	413.90
P02768	E266	YIC(CAM)E(HETETE)NQDSISSK	$C_{65}H_{109}N_{16}O_{25}S_3$	1609.69	805.85	2	1.02	414.20
P02768	E354	TYE(HETETE)TTLEK	$C_{49}H_{81}N_9O_{18}S_2$	1147.51	574.76	2	0.91	374.10
P02768	E570	ADDKETC(CAM)FAE(HETETE)EGK	$C_{67}H_{106}N_{16}O_{27}S_3$	1662.66	832.33	2	1.06	431.20
P02768	D375	VFD(HETETE)EFKPLVEEPQNLIK	$C_{102}H_{161}N_{21}O_{29}S_2$	2208.12	737.04	3	0.99	604.50
P02768	E376	VFDE(HETETE)FKPLVEEPQNLIK	$C_{102}H_{161}N_{21}O_{29}S_2$	2208.12	737.04	3	0.95	582.70
P02768	E382	VFDEFKPLVE(HETETE)EPQNLIK	$C_{102}H_{161}N_{21}O_{29}S_2$	2208.12	737.04	3	0.91	558.20
P02768	E383	VFDEFKPLVEE(HETETE)PQNLIK	$C_{102}H_{161}N_{21}O_{29}S_2$	2208.12	737.04	3	0.90	550.10
P02787	E328	EGTC(CAM)PE(HETETE)APTDEC(CAM)KPVK	$C_{80}H_{132}N_{20}O_{30}S_4$	1980.83	661.28	3	0.89	543.70
P02787	E352	C(CAM)DE(HETETE)WSVNSVGK	$C_{59}H_{93}N_{15}O_{21}S_3$	1443.58	722.79	2	0.98	402.20
P0C0L5	E1333	GLEEE(HETETE)LQFSLGSK	$C_{69}H_{113}N_{15}O_{24}S_2$	1599.75	800.88	2	1.05	427.80
P0DOY2	E30	AGVE(HETETE)TTTPSK	$C_{47}H_{83}N_{11}O_{18}S_2$	1153.54	577.77	2	0.86	353.00

Table S11 Identified peptide adducts with importance in random forest as well as contribution to PCA (cf. intersect Venn diagram in Figure 3 of manuscript) and their HD and T analogs in different charge states (when found) are listed. [a]Modification Site on the protein, i.e. amino acid and position (without signal sequences and propeptides). [b]30 min gradient. [c]Calculated according to Mason-Schamp[29] at 305 K. [d]Reference for already published modification sites. *CAM: Carbamidomethyl; UniMod:7: Deamidation.*

	Protein	Mod. Site[a]	Peptide	m/z	z	RT[b] [min]	IM [V s cm^{-2}]	CCS[c] [Å^2]	Ref.[d]
1	A0M8Q6	E77	YAASSYLSLTPE^{HETETE}QWK	954.45	2	27.8	1.16	469.18	
2	A0M8Q6	E77	YAASSYLSLTPE^{HETEOETE}QWK	976.46	2	27.9	1.18	476.59	
3	A0M8Q6	E77	YAASSYLSLTPE^{HETE}QWK	924.45	2	25.2	1.15	464.44	
4	O43866	H308	C(CAM)SGEEQSLEQC(CAM)QH^{HETETE}R	637.92	3	11.6	0.88	529.02	
5	P00450	D921	TYSD^{HETETE}HPEKVNK	494.56	3	9.3	0.79	478.21	
6	P00738	C291	YVMLPVADQDQC^{HETETE}IR	907.92	2	24.9	1.14	460.71	
7	P00738	E112	TEGDGVYTLNNE^{HETETE}K	802.35	2	19.4	1.06	427.80	
8	P00738	E112	TEGDGVYTLNNE^{HETEOETE}K	824.37	2	19.9	1.08	436.02	
9	P00738	E112	TEGDGVYTLNNE^{HETE}K	772.35	2	16.2	1.05	424.48	
10	P00738	H319	SPVGVQPILNEH^{HETETE}TFC(CAM)AGMSK	1168.55	2	23.0	1.29	519.42	
11	P00738	H319	SPVGVQPILNEH^{HETETE}TFC(CAM)AGMSK	779.37	3	23.0	1.00	600.67	
12	P00738	E318	SPVGVQPILNE^{HETETE}HTFC(CAM)AGMSK	1168.55	2	24.9	1.30	521.80	
13	P00738	E318	SPVGVQPILNE^{HETETE}HTFC(CAM)AGMSK	779.37	3	24.9	1.00	601.64	
14	P00738	E79	AVGDKLPEC(CAM)EADDGC(CAM)PKPPE^{HETETE}IAHGYVEHSVR	899.91	4	17.7	1.12	902.90	
15	P00738	E79	AVGDKLPEC(CAM)EADDGC(CAM)PKPPE^{HETETE}IAHGYVEHSVR	720.13	5	17.7	1.01	1014.23	
16	P00747	D149	FSPATHPSEGLEENYC(CAM)RNPDNDPQGPWC(CAM)YTTD^{HETETE}PEKR	886.58	5	21.6	1.09	1095.81	
17	P00751	E159	LE^{HETETE}DSVTYHC(CAM)SR	510.88	3	15.2	0.82	497.93	
18	P01008	H65	FATTFYQH^{HETETE}LADSK	846.89	2	21.0	1.15	462.48	
19	P01008	H65	FATTFYQH^{HETETE}LADSK	564.93	3	21.0	0.80	485.87	
20	P01008	H65	FATTFYQH^{HETEOETE}LADSK	868.90	2	21.3	1.18	475.19	
21	P01008	H65	FATTFYQH^{HETEOETE}LADSK	579.61	3	21.7	0.80	485.16	
22	P01008	H65	FATTFYQH^{HETE}LADSK	816.89	2	19.4	1.14	459.30	
23	P01008	H65	FATTFYQH^{HETE}LADSK	544.93	3	19.5	0.79	475.51	
24	P01009	H209	WERPFEVKDTEEEDFH^{HETETE}VDQVTTVK	782.62	4	21.0	1.03	825.66	
25	P01009	H209	WERPFEVKDTEEEDFH^{HETETE}VDQVTTVK	626.29	5	21.0	0.95	956.54	
26	P01009	H209	WERPFEVKDTEEEDFH^{HETEOETE}VDQVTTVK	793.62	4	21.0	1.04	832.41	
27	P01009	H209	WERPFEVKDTEEEDFH^{HETE}VDQVTTVK	767.61	4	20.1	1.04	836.84	
28	P01009	H209	WERPFEVKDTEEEDFH^{HETE}VDQVTTVK	614.29	5	20.1	0.96	968.57	
29	P01009	E324	VFSNGADLSGVTEE^{HETETE}APLK	999.48	2	25.0	1.22	491.40	
30	P01009	E324	VFSNGADLSGVTEE^{HETEOETE}APLK	1021.49	2	25.0	1.23	496.18	
31	P01009	E324	VFSNGADLSGVTEE^{HETE}APLK	969.48	2	22.8	1.21	488.55	
32	P01009	H20	TDTSHHDQDH^{HETETE}PTFNK	648.61	3	10.3	0.87	528.03	
33	P01009	H20	TDTSHHDQDH^{HETETE}PTFNK	486.71	4	10.5	0.80	642.44	
34	P01009	H20	TDTSHHDQDH^{HETEOETE}PTFNK	994.42	2	11.1	1.16	466.21	

Table S11 continued

	Protein	Mod. Site[a]	Peptide	m/z	z	RT [min]	IM [V s cm^{-2}]	CCS[b] [Å^2]	Ref.[c]
35	P01009	H20	TDTSHHDQDH^{HETEOETE}PTFNK	663.28	3	11.2	0.88	532.41	
36	P01009	H20	TDTSHHDQDH^{HETE}PTFNK	628.60	3	8.4	0.86	521.82	
37	P01009	H20	TDTSHHDQDH^{HETE}PTFNK	471.70	4	8.5	0.81	650.84	
38	P01009	D17	TDTSHHD^{HETETE}QDHPTFNK	648.61	3	9.8	0.88	533.77	
39	P01009	D17	TDTSHHD^{HETETE}QDHPTFNK	486.71	4	10.1	0.78	624.60	
40	P01009	D17	TDTSHHD^{HETE}QDHPTFNK	471.70	4	8.2	0.78	630.69	
41	P01009	H16	TDTSHH^{HETETE}DQDHPTFNK	648.61	3	9.5	0.87	527.62	
42	P01009	H16	TDTSHH^{HETETE}DQDHPTFNK	486.71	4	9.9	0.78	627.90	
43	P01009	H16	TDTSHH^{HETEOETE}DQDHPTFNK	663.28	3	10.6	0.90	543.04	
44	P01009	H16	TDTSHH^{HETEOETE}DQDHPTFNK	497.71	4	11.2	0.83	668.08	
45	P01009	H16	TDTSHH^{HETE}DQDHPTFNK	628.60	3	8.2	0.88	530.93	
46	P01009	H16	TDTSHH^{HETE}DQDHPTFNK	471.70	4	8.1	0.79	638.67	
47	P01009	H15	TDTSH^{HETETE}HDQDHPTFNK	972.40	2	9.7	1.14	458.05	
48	P01009	H15	TDTSH^{HETETE}HDQDHPTFNK	648.61	3	10.0	0.88	529.45	
49	P01009	H15	TDTSH^{HETETE}HDQDHPTFNK	486.71	4	10.1	0.78	630.17	
50	P01009	H15	TDTSH^{HETEOETE}HDQDHPTFNK	497.71	4	10.3	0.80	640.80	
51	P01009	H15	TDTSH^{HETE}HDQDHPTFNK	942.40	2	8.0	1.12	451.74	
52	P01009	H15	TDTSH^{HETE}HDQDHPTFNK	628.60	3	8.2	0.87	528.87	
53	P01009	H15	TDTSH^{HETE}HDQDHPTFNK	471.70	4	8.2	0.78	628.00	
54	P01009	H287	SASLH^{HETETE}LPK	508.77	2	15.7	0.89	360.30	
55	P01009	H287	SASLH^{HETEOETE}LPK	530.78	2	16.3	0.91	368.60	
56	P01009	H287	SASLH^{HETE}LPK	478.77	2	13.5	0.89	360.89	
57	P01009	C232	RLGMFNIQHC^{HETETE}KK	546.94	3	16.8	0.86	517.58	
58	P01009	C232	RLGMFNIQHC^{HETETE}KK	410.46	4	16.8	0.71	573.30	
59	P01009	C232	RLGMFNIQHC^{HETETE}K	755.86	2	19.2	1.07	432.49	
60	P01009	C232	RLGMFNIQHC^{HETETE}K	504.24	3	19.5	0.81	491.53	
61	P01009	C232	RLGMFNIQHC^{HETEOETE}KK	561.62	3	17.2	0.85	516.54	
62	P01009	C232	RLGMFNIQHC^{HETEOETE}KK	421.46	4	17.2	0.72	584.69	
63	P01009	C232	RLGMFNIQHC^{HETEOETE}K	518.92	3	19.6	0.83	503.48	
64	P01009	C232	RLGMFNIQHC^{HETE}KK	526.94	3	14.3	0.84	507.36	
65	P01009	C232	RLGMFNIQHC^{HETE}K	484.24	3	16.9	0.81	489.13	
66	P01009	H231	RLGMFNIQH^{HETETE}C(CAM)K	523.25	3	17.6	0.84	507.57	
67	P01009	E266	LQHLENE^{HETETE}LTHDIITK	984.50	2	21.6	1.19	478.77	
68	P01009	E266	LQHLENE^{HETETE}LTHDIITK	656.67	3	21.8	0.94	566.36	
69	P01009	E266	LQHLENE^{HETETE}LTHDIITK	492.75	4	21.7	0.79	637.06	
70	P01009	E266	LQHLENE^{HETE}LTHDIITK	954.50	2	19.7	1.18	473.57	

Table S11 continued

	Protein	Mod. Site[a]	Peptide	m/z	z	RT [min]	IM [V s cm^{-2}]	CCS[b] [Å^2]	Ref.[c]
71	P01009	E266	LQHLENE^{HETE}LTHDIITK	636.67	3	19.7	0.93	563.58	
72	P01009	E266	LQHLENE^{HETE}LTHDIITK	477.75	4	18.7	0.81	655.05	
73	P01009	E264	LQHLE^{HETETE}NELTHDIITK	656.67	3	21.8	0.93	561.56	
74	P01009	E264	LQHLE^{HETETE}NELTHDIITK	492.75	4	20.2	0.81	648.85	
75	P01009	E264	LQHLE^{HETEOETE}NELTHDIITK	671.34	3	20.0	0.96	582.02	
76	P01009	E264	LQHLE^{HETE}NELTHDIITK	636.67	3	18.5	0.92	558.73	
77	P01009	C232	LGMFNIQHC^{HETETE}K	677.81	2	21.5	1.03	414.67	
78	P01009	C232	LGMFNIQHC^{HETETE}K	452.21	3	21.4	0.77	469.51	
79	P01009	C232	LGMFNIQHC^{HETEOETE}K	699.82	2	21.7	1.05	422.24	
80	P01009	C232	LGMFNIQHC^{HETE}K	647.81	2	19.0	1.01	406.99	
81	P01009	C232	LGMFNIQHC^{HETE}K	432.21	3	19.0	0.75	456.80	
82	P01009	H231	LGMFNIQH^{HETETE}C(CAM)K	706.32	2	19.9	1.05	422.67	
83	P01009	H231	LGMFNIQH^{HETETE}C(CAM)K	471.22	3	19.9	0.75	455.50	
84	P01009	D202	D^{HETETE}TEEEDFHVDQVTTVK	1028.45	2	21.2	1.23	494.99	
85	P01009	D202	D^{HETEOETE}TEEEDFHVDQVTTVK	1050.46	2	21.4	1.26	505.47	
86	P01009	D202	D^{HETE}TEEEDFHVDQVTTVK	665.97	3	18.9	0.92	557.42	
87	P01009	H334	AVH^{HETETE}KAVLTIDEK	744.40	2	14.6	1.08	435.35	
88	P01009	H334	AVH^{HETETE}KAVLTIDEK	496.60	3	14.7	0.81	488.23	
89	P01009	H334	AVH^{HETEOETE}KAVLTIDEK	766.41	2	15.4	1.08	435.95	
90	P01009	H334	AVH^{HETEOETE}KAVLTIDEK	511.28	3	15.4	0.83	500.39	
91	P01009	H334	AVH^{HETE}KAVLTIDEK	714.40	2	12.7	1.04	419.71	
92	P01009	H334	AVH^{HETE}KAVLTIDEK	476.60	3	12.7	0.81	491.02	
93	P01011	E263	MEE^{HETETE}VEAMLLPETLKR	976.98	2	27.8	1.25	503.76	
94	P01011	E263	MEE^{HETETE}VEAMLLPETLKR	651.66	3	27.7	0.98	592.86	
95	P01011	E263	MEE^{HETEOETE}VEAMLLPETLKR	666.33	3	27.8	0.98	595.09	
96	P01011	E263	MEE^{HETE}VEAMLLPETLKR	631.66	3	25.3	0.97	584.96	
97	P01023	E686	VGFYE^{HETETE}SDVMGR	712.31	2	24.2	1.01	409.09	
98	P01023	E686	VGFYE^{HETEOETE}SDVMGR	734.32	2	24.5	1.03	417.80	
99	P01023	E686	VGFYE^{HETE}SDVMGR	682.30	2	21.2	1.00	405.79	
100	P01023	E193	TE^{HETETE}HPFTVEEFVLPK	612.97	3	25.4	0.92	556.33	
101	P01023	E193	TE^{HETEOETE}HPFTVEEFVLPK	627.64	3	25.4	0.94	565.30	
102	P01023	H1120	TAQEGDHGSH^{HETETE}VYTK	565.25	3	9.0	0.86	517.84	
103	P01023	H1120	TAQEGDHGSH^{HETE}VYTK	545.25	3	7.4	0.84	509.24	
104	P01023	E439	SFVHLE^{HETETE}PMSHELPC(CAM)GHTQTVQAHYILNGGTLLGLK	810.60	5	24.6	1.09	1095.92	
105	P01023	H704	LVHVEEPH^{HETETE}TETVRK	460.24	4	12.3	0.76	610.38	
106	P01023	H704	LVHVEEPH^{HETEOETE}TETVRK	627.99	3	12.6	0.89	540.63	

Table S11 continued

	**Protein**	**Mod. Site**[a]	**Peptide**	**m/z**	**z**	**RT** [min]	**IM** [V s cm^{-2}]	**CCS**[b] [Å^2]	**Ref.**[c]
107	P01023	H704	LVHVEEPH^{HETE}TETVRK	593.31	3	10.8	0.86	520.35	
108	P01023	H704	LVHVEEPH^{HETE}TETVRK	445.24	4	10.8	0.75	608.46	
109	P01023	E888	DTVIKPLLVEPEGLE^{HETETE}K	648.69	3	27.6	0.96	581.40	
110	P01023	E888	DTVIKPLLVEPEGLE^{HETEOETE}K	994.54	2	27.3	1.24	500.16	
111	P01023	E888	DTVIKPLLVEPEGLE^{HETE}K	942.52	2	24.8	1.19	480.84	
112	P01023	E1431 E1434	DLKPAIVKVYDYYE^{HETETE}TDE^{HETETE}FAIAEYNAPC(CAM)SK	935.93	4	29.0	1.10	882.69	
113	P01023	D1418	D^{HETETE}LKPAIVKVYDYYETDEFAIAEYNAPC(CAM)SK	894.92	4	27.8	1.11	891.81	
114	P01024	E1350	VTIKPAPE^{HETETE}TEK	688.86	2	15.7	1.03	415.44	
115	P01024	E1350	VTIKPAPE^{HETETE}TEK	459.58	3	15.7	0.80	481.90	
116	P01024	E1350	VTIKPAPE^{HETEOETE}TEK	710.87	2	16.4	1.04	419.47	
117	P01024	E1350	VTIKPAPE^{HETE}TEK	439.58	3	12.8	0.80	485.25	
118	P01024	H1431	VSH^{HETETE}SEDDC(CAM)LAFK	786.33	2	16.3	1.06	429.73	
119	P01024	H1431	VSH^{HETETE}SEDDC(CAM)LAFK	524.56	3	16.4	0.80	481.96	
120	P01024	H1431 E1433	VSH^{HETETE}SE^{HETETE}DDC(CAM)LAFK	579.23	3	18.6	0.88	530.39	
121	P01024	E1448	VHQYFNVE^{HETETE}LIQPGAVK	1003.52	2	25.1	1.24	500.39	
122	P01024	E1448	VHQYFNVE^{HETEOETE}LIQPGAVK	1025.53	2	25.3	1.26	507.29	
123	P01024	E1448	VHQYFNVE^{HETE}LIQPGAVK	973.51	2	23.3	1.23	494.89	
124	P01024	H334	SGIPIVTSPYQIH^{HETETE}FTKTPK	1139.60	2	23.1	1.36	546.11	
125	P01024	H334	SGIPIVTSPYQIH^{HETETE}FTKTPK	760.07	3	23.1	1.01	607.36	
126	P01024	H334	SGIPIVTSPYQIH^{HETETE}FTK	976.50	2	24.4	1.22	490.81	
127	P01024	H334	SGIPIVTSPYQIH^{HETETE}FTK	651.34	3	24.5	0.90	544.28	
128	P01024	H334	SGIPIVTSPYQIH^{HETEOETE}FTKTPK	1161.62	2	23.2	1.37	552.65	
129	P01024	H334	SGIPIVTSPYQIH^{HETEOETE}FTKTPK	774.75	3	23.1	1.02	615.58	
130	P01024	H334	SGIPIVTSPYQIH^{HETEOETE}FTKTPK	581.31	4	23.1	0.90	720.77	
131	P01024	H334	SGIPIVTSPYQIH^{HETEOETE}FTK	998.52	2	24.7	1.25	503.50	
132	P01024	H334	SGIPIVTSPYQIH^{HETE}FTKTPK	1109.60	2	21.7	1.33	536.41	
133	P01024	E413	KQELSEAE^{HETETE}QATR	777.37	2	14.5	1.04	420.12	
134	P01024	E413	KQELSEAE^{HETEOETE}QATR	799.38	2	15.2	1.05	422.99	
135	P01024	E413	KQELSEAE^{HETEOETE}QATR	533.26	3	15.3	0.83	499.64	
136	P01024	E413	KQELSEAE^{HETE}QATR	747.37	2	11.8	1.02	411.58	
137	P01024	E970	ILLQGTPVAQMTE^{HETETE}DAVDAER	1161.06	2	28.5	1.27	512.44	
138	P01024	E970	ILLQGTPVAQMTE^{HETEOETE}DAVDAER	1183.08	2	28.4	1.27	513.02	
139	P01024	E970	ILLQGTPVAQMTE^{HETE}DAVDAER	1131.06	2	24.3	1.27	510.60	
140	P01024	E1138	DIC(CAM)EE^{HETETE}QVNSLPGSITK	977.45	2	23.2	1.17	472.25	

Table S11 continued

	Protein	Mod. Site[a]	Peptide	m/z	z	RT [min]	IM [$V\,s\,cm^{-2}$]	CCS[b] [$Å^2$]	Ref.[c]
141	P01024	H897	AAVYHH^{HETETE}FISDGVR	545.93	3	16.4	0.84	506.55	
142	P01024	H897	AAVYHH^{HETE}FISDGVR	525.93	3	14.2	0.84	509.68	
143	P01024	H896	AAVYH^{HETETE}HFISDGVRK	588.63	3	14.6	0.86	521.28	
144	P01024	H896	AAVYH^{HETEOETE}HFISDGVRK	603.30	3	14.9	0.87	526.33	
145	P01024	H896	AAVYH^{HETEOETE}HFISDGVRK	452.73	4	15.1	0.77	619.25	
146	P01024	H896	AAVYH^{HETE}HFISDGVRK	568.63	3	12.1	0.86	517.90	
147	P01859	D191	VVSVLTVVHQD^{HETETE}WLNGKEYK	793.41	3	25.4	1.03	619.87	
148	P01859	C102	C^{HETETE}CVECPPC(CAM)PAPPVAGPSVFLFPPKPK	581.08	5	27.0	0.86	861.51	
149	P01860	H215	WYVDGVEVH^{HETETE}NAK	790.87	2	22.4	1.13	456.96	
150	P01860	H215	WYVDGVEVH^{HETETE}NAK	527.58	3	19.4	0.87	525.21	
151	P01860	H215	WYVDGVEVH^{HETEOETE}NAK	812.88	2	22.6	1.13	456.72	
152	P01860	H215	WYVDGVEVH^{HETEOETE}NAK	542.25	3	19.9	0.89	539.35	
153	P01860	H215	WYVDGVEVH^{HETE}NAK	760.86	2	17.3	1.11	448.24	
154	P01860	E248	VVSVLTVLHQDWLNGKE^{HETETE}YK	798.08	3	27.8	1.02	617.46	
155	P01860	E263	ALPAPIE^{HETETE}KTISK	716.40	2	19.1	1.07	434.22	
156	P01860	E263	ALPAPIE^{HETEOETE}KTISK	738.41	2	19.5	1.09	441.18	
157	P01860	E263	ALPAPIE^{HETE}KTISK	686.40	2	17.3	1.05	422.33	
158	P01861	E198	VVSVLTVLHQDWLNGKE^{HETEOETE}YK	812.76	3	27.8	1.04	625.52	
159	P01861	E198	VVSVLTVLHQDWLNGKE^{HETE}YK	778.08	3	26.2	1.03	619.85	
160	P01861	E262	GFYPSDIAVEWE^{HETETE}SNGQPENNYK	1354.59	2	28.6	1.35	542.52	
161	P01861	E262	GFYPSDIAVEWE^{HETEOETE}SNGQPENNYK	1376.60	2	28.6	1.35	543.44	
162	P01871	E385	YVTSAPMPE^{HETETE}PQAPGR	882.91	2	21.0	1.11	448.12	
163	P01871	E385	YVTSAPMPE^{HETEOETE}PQAPGR	904.92	2	21.5	1.13	454.58	
164	P01871	E385	YVTSAPMPE^{HETE}PQAPGR	852.91	2	18.5	1.08	435.11	
165	P01871	E167	QVGSGVTTDQVQAE^{HETETE}AK	891.42	2	17.6	1.14	459.06	
166	P01871	E167	QVGSGVTTDQVQAE^{HETE}AK	861.42	2	14.9	1.13	454.19	
167	P01871	E84	DVMQGTDE^{HETETE}HVVC(CAM)K	841.36	2	17.4	1.12	452.52	
168	P01876	E176	SAVQGPPE^{HETETE}R	552.76	2	15.0	0.84	342.09	
169	P01876	E176	SAVQGPPE^{HETEOETE}R	574.78	2	15.8	0.88	356.04	
170	P01876	E176	SAVQGPPE^{HETE}R	522.76	2	11.5	0.83	338.21	
171	P02647	E119	WQEEME^{HETETE}LYR	724.31	2	22.8	1.02	413.71	
172	P02647	E116	WQE^{HETETE}EMELYR	724.31	2	24.5	1.05	422.10	
173	P02647	E116	WQE^{HETEOETE}EMELYR	746.32	2	24.8	1.06	429.64	
174	P02647	E241	VSFLSALEE^{HETETE}YTKK	839.93	2	28.4	1.17	471.43	
175	P02647	E241	VSFLSALEE^{HETETE}YTKK	560.29	3	27.9	0.82	496.92	
176	P02647	E241	VSFLSALEE^{HETEOETE}YTKK	861.94	2	28.3	1.18	476.36	

Table S11 continued

	Protein	Mod. Site[a]	Peptide	m/z	z	RT [min]	IM [V s cm^{-2}]	CCS[b] [$\AA^2$]	Ref.[c]
177	P02647	E241	VSFLSALEE^{HETEOETE}YTKK	574.96	3	28.3	0.83	501.11	
178	P02647	E241	VSFLSALEE^{HETE}YTKK	809.92	2	26.3	1.15	465.49	
179	P02647	E241	VSFLSALEE^{HETE}YTKK	540.28	3	26.3	0.82	495.74	
180	P02647	E240	VSFLSALE^{HETETE}EYTKK	560.29	3	29.0	0.80	484.60	
181	P02647	E240	VSFLSALE^{HETE}EYTKK	809.92	2	26.9	1.14	461.79	
182	P02647	E240	VSFLSALE^{HETE}EYTKK	540.28	3	26.9	0.83	502.16	
183	P02647	D30	VKDLATVYVDVLKD^{HETETE}SGR	1021.54	2	23.6	1.32	532.79	
184	P02647	D30	VKDLATVYVDVLKD^{HETETE}SGR	681.36	3	23.9	0.98	592.69	
185	P02647	D30	VKDLATVYVDVLKD^{HETE}SGR	661.36	3	22.8	0.98	591.25	
186	P02647	D26	VKDLATVYVD^{HETETE}VLK	813.95	2	25.2	1.12	452.28	
187	P02647	D26	VKDLATVYVD^{HETETE}VLK	542.97	3	25.2	0.80	482.75	
188	P02647	D26	VKDLATVYVD^{HETEOETE}VLK	835.96	2	25.1	1.14	458.91	
189	P02647	D19	VKD^{HETETE}LATVYVDVLK	813.95	2	24.4	1.13	454.25	
190	P02647	D19	VKD^{HETETE}LATVYVDVLK	542.97	3	24.4	0.86	520.46	
191	P02647	D19	VKD^{HETEOETE}LATVYVDVLK	835.96	2	24.5	1.14	461.27	
192	P02647	D19	VKD^{HETE}LATVYVDVLK	783.94	2	23.1	1.11	446.23	
193	P02647	D19	VKD^{HETE}LATVYVDVLK	522.97	3	23.1	0.85	516.08	
194	P02647	E175	THLAPYSDE^{HETETE}LR	733.34	2	19.8	1.05	424.41	
195	P02647	E175	THLAPYSDE^{HETEOETE}LR	755.36	2	20.1	1.09	439.70	
196	P02647	E175	THLAPYSDE^{HETE}LR	703.34	2	16.9	1.04	421.55	
197	P02647	D174	THLAPYSD^{HETETE}ELR	733.34	2	21.1	1.04	418.04	
198	P02647	H168	TH^{HETETE}LAPYSDELR	733.34	2	17.0	1.03	414.43	
199	P02647	H168	TH^{HETETE}LAPYSDELR	489.23	3	16.8	0.75	453.89	
200	P02647	H168	TH^{HETEOETE}LAPYSDELR	755.36	2	17.4	1.03	416.91	
201	P02647	H168	TH^{HETEOETE}LAPYSDELR	503.91	3	17.2	0.83	503.88	
202	P02647	H168	TH^{HETE}LAPYSDELR	703.34	2	14.8	1.02	410.44	
203	P02647	E145	LHELQE^{HETETE}K	530.76	2	14.5	0.88	356.72	
204	P02647	E145	LHELQE^{HETEOETE}K	552.77	2	15.5	0.89	362.11	
205	P02647	E145	LHELQE^{HETE}K	500.76	2	10.9	0.84	342.02	
206	P02647	H141	LH^{HETETE}ELQEK	530.76	2	11.6	0.87	352.21	
207	P02647	H141	LH^{HETEOETE}ELQEK	552.77	2	12.4	0.93	376.37	
208	P02647	H199	LAEYH^{HETETE}AK	498.24	2	10.2	0.88	354.94	
209	P02647	H199	LAEYH^{HETEOETE}AK	520.25	2	11.2	0.91	367.37	
210	P02647	E197	LAE^{HETETE}YHAK	498.24	2	13.6	0.86	347.26	
211	P02647	E197	LAE^{HETEOETE}YHAK	520.25	2	14.1	0.88	356.13	
212	P02647	E197	LAE^{HETE}YHAK	468.24	2	10.1	0.85	345.24	

Table S11 continued

	Protein	Mod. Site[a]	Peptide	m/z	z	RT [min]	IM [V s cm^{-2}]	CCS[b] [Å^2]	Ref.[c]
213	P02647	E117	KWQEE^{HETETE}MELYR	788.35	2	21.9	1.08	436.03	
214	P02647	E117	KWQEE^{HETEOETE}MELYR	810.37	2	22.1	1.10	444.53	
215	P02647	E117	KWQEE^{HETE}MELYR	758.35	2	19.4	1.06	427.57	
216	P02647	E82	EQLGPVTQEFWDNLE^{HETETE}K	1048.99	2	27.6	1.23	496.19	
217	P02647	E82	EQLGPVTQEFWDNLE^{HETEOETE}K	1071.00	2	27.7	1.24	500.44	
218	P02647	E82	EQLGPVTQEFWDNLE^{HETE}K	1018.99	2	25.0	1.21	487.01	
219	P02647	D26	DLATVYVD^{HETETE}VLK	700.36	2	29.0	1.04	420.85	
220	P02647	E211	ATEHLSTLSE^{HETETE}KAKPALEDLR	594.06	4	18.6	0.92	739.42	
221	P02647	E211	ATEHLSTLSE^{HETETE}K	690.33	2	16.7	1.01	409.48	
222	P02647	E211	ATEHLSTLSE^{HETETE}K	460.56	3	17.1	0.79	476.53	
223	P02647	E211	ATEHLSTLSE^{HETEOETE}KAKPALEDLR	605.07	4	18.8	0.91	732.92	
224	P02647	E211	ATEHLSTLSE^{HETEOETE}K	712.34	2	17.3	1.04	421.03	
225	P02647	E211	ATEHLSTLSE^{HETE}KAKPALEDLR	579.06	4	17.2	0.91	732.88	
226	P02647	E211	ATEHLSTLSE^{HETE}K	660.33	2	13.4	1.00	403.60	
227	P02647	E211	ATEHLSTLSE^{HETE}K	440.56	3	13.4	0.75	453.79	
228	P02647	H205	ATEH^{HETETE}LSTLSEK	690.33	2	13.3	1.03	416.25	
229	P02647	H205	ATEH^{HETETE}LSTLSEK	460.56	3	13.3	0.71	431.12	
230	P02647	H205	ATEH^{HETEOETE}LSTLSEK	712.34	2	14.2	1.05	423.93	
231	P02647	H205	ATEH^{HETEOETE}LSTLSEK	475.23	3	14.2	0.76	461.29	
232	P02647	H205	ATEH^{HETE}LSTLSEK	660.33	2	10.8	0.98	398.10	
233	P02647	H205	ATEH^{HETE}LSTLSEK	440.56	3	10.8	0.72	433.63	
234	P02647	E204	ATE^{HETETE}HLSTLSEK	690.33	2	17.0	1.02	412.12	
235	P02647	E204	ATE^{HETETE}HLSTLSEK	460.56	3	17.0	0.78	473.61	
236	P02647	E204	ATE^{HETEOETE}HLSTLSEK	712.34	2	17.4	1.04	421.08	
237	P02647	E204	ATE^{HETEOETE}HLSTLSEK	475.23	3	14.2	0.80	484.64	
238	P02647	E204	ATE^{HETE}HLSTLSEK	660.33	2	13.6	1.01	406.85	
239	P02647	E204	ATE^{HETE}HLSTLSEK	440.56	3	10.8	0.72	435.30	
240	P02647	E218	AKPALE^{HETETE}DLR	588.81	2	18.6	0.93	377.73	
241	P02647	E218	AKPALE^{HETEOETE}DLR	610.82	2	18.4	0.95	383.94	
242	P02647	E218	AKPALE^{HETE}DLR	558.81	2	15.3	0.89	360.69	
243	P02647	D163	AHVD^{HETETE}ALRTHLAPYSDELR	557.78	4	14.7	0.89	719.57	
244	P02647	D163	AHVD^{HETEOETE}ALRTHLAPYSDELR	568.79	4	15.1	0.93	752.13	
245	P02647	H161	AH^{HETETE}VDALR	473.24	2	12.0	0.82	331.52	
246	P02647	H161	AH^{HETEOETE}VDALR	495.25	2	13.0	0.87	353.33	
247	P02649	D297	VQAAVGTSAAPVPSD^{HETETE}NH	892.92	2	16.0	1.11	447.48	
248	P02649	D297	VQAAVGTSAAPVPSD^{HETEOETE}NH	914.93	2	16.7	1.13	455.43	

Table S11 continued

	Protein	Mod. Site[a]	Peptide	m/z	z	RT [min]	IM [V s cm^{-2}]	CCS[b] [$Å^2$]	Ref.[c]
249	P02649	D297	VQAAVGTSAAPVPSD^{HETE}NH	862.92	2	14.2	1.11	445.80	
250	P02656	E32	DALSSVQE^{HETETE}SQVAQQAR	940.95	2	20.2	1.14	460.55	
251	P02656	E32	DALSSVQE^{HETEOETE}SQVAQQAR	962.96	2	20.6	1.15	462.82	
252	P02656	E32	DALSSVQE^{HETE}SQVAQQAR	910.94	2	17.6	1.12	451.49	
253	P02743	E136	IVLGQE^{HETETE}QDSYGGK	779.37	2	20.1	1.05	423.82	
254	P02743	E136	IVLGQE^{HETE}QDSYGGK	749.37	2	16.9	1.04	421.33	
255	P02749	E321	C(CAM)SYTEDAQC(CAM)IDGTIE^{HETETE}VPK	1125.48	2	22.6	1.24	497.87	
256	P02763	H100	YVGGQEHFAH^{HETETE}LLILR	639.67	3	23.3	0.89	540.52	
257	P02763	H100	YVGGQEHFAH^{HETETE}LLILR	480.00	4	23.4	0.83	670.44	
258	P02763	H100	YVGGQEHFAH^{HETE}LLILR	929.00	2	21.7	1.21	488.19	
259	P02763	E96	YVGGQE^{HETETE}HFAHLLILR	959.00	2	24.4	1.23	496.65	
260	P02763	E96	YVGGQE^{HETETE}HFAHLLILR	639.67	3	23.7	0.92	553.65	
261	P02763	E96	YVGGQE^{HETETE}HFAHLLILR	480.00	4	23.5	0.83	668.13	
262	P02763	E96	YVGGQE^{HETEOETE}HFAHLLILR	981.01	2	23.7	1.23	495.42	
263	P02763	E96	YVGGQE^{HETEOETE}HFAHLLILR	654.34	3	23.3	0.94	566.62	
264	P02763	E96	YVGGQE^{HETEOETE}HFAHLLILR	491.01	4	23.6	0.84	678.94	
265	P02763	E96	YVGGQE^{HETE}HFAHLLILR	929.00	2	22.4	1.22	490.42	
266	P02763	E96	YVGGQE^{HETE}HFAHLLILR	619.67	3	22.4	0.93	562.68	
267	P02763	E96	YVGGQE^{HETE}HFAHLLILR	465.00	4	21.7	0.83	672.16	
268	P02763	E57	TE^{HETETE}DTIFLR	579.78	2	24.4	0.89	359.91	
269	P02763	E57	TE^{HETEOETE}DTIFLR	601.79	2	24.7	0.94	378.79	
270	P02763	E132	NWGLSVYADKPE^{HETETE}TTK	936.95	2	21.9	1.24	498.07	
271	P02763	E132	NWGLSVYADKPE^{HETEOETE}TTK	958.96	2	21.9	1.22	493.35	
272	P02763	E132	NWGLSVYADKPE^{HETEOETE}TTK	639.64	3	22.0	0.91	552.88	
273	P02763	E132	NWGLSVYADKPE^{HETE}TTK	906.95	2	19.8	1.21	487.87	
274	P02763	E132	NWGLSVYADKPE^{HETE}TTK	604.97	3	19.8	0.89	538.14	
275	P02763	D154	IPKSD^{HETETE}VVYTDWKK	581.63	3	16.6	0.88	530.92	
276	P02765	E314	HTFMGVVSLGSPSGE^{HETETE}VSHPR	749.02	3	22.0	1.07	645.94	
277	P02765	E314	HTFMGVVSLGSPSGE^{HETEOETE}VSHPR	763.70	3	22.4	1.09	657.95	
278	P02765	E314	HTFMGVVSLGSPSGE^{HETE}VSHPR	729.02	3	20.3	1.07	645.41	
279	P02766	H31	GSPAINVAVH^{HETETE}VFR	765.90	2	23.0	1.13	454.39	
280	P02766	H31	GSPAINVAVH^{HETETE}VFR	510.94	3	23.4	0.75	455.48	
281	P02766	H31	GSPAINVAVH^{HETEOETE}VFR	787.91	2	23.2	1.15	463.00	
282	P02766	H31	GSPAINVAVH^{HETEOETE}VFR	525.61	3	23.3	0.77	465.55	
283	P02766	H31	GSPAINVAVH^{HETE}VFR	735.90	2	21.5	1.12	451.11	
284	P02766	E42	AADDTWE^{HETETE}PFASGK	779.83	2	25.1	1.04	419.21	

Table S11 continued

	Protein	Mod. Site[a]	Peptide	m/z	z	RT [min]	IM [V s cm^{-2}]	CCS[b] [Å^2]	Ref.[c]
285	P02766	E42	AADDTWE^{HETEOETE}PFASGK	801.84	2	25.1	1.05	422.71	
286	P02768	E167	YKAAFTE^{HETETE}C(CAM)C(CAM)QAADK	913.88	2	13.1	1.14	457.67	
287	P02768	E167	YKAAFTE^{HETETE}C(CAM)C(CAM)QAADK	609.59	3	13.5	0.83	500.99	
288	P02768	C265	YIC^{HETETE}ENQDSISSK	775.83	2	17.1	1.04	421.66	
289	P02768	C265	YIC^{HETEOETE}ENQDSISSK	797.84	2	17.7	1.05	424.24	
290	P02768	C265	YIC^{HETE}ENQDSISSK	745.83	2	13.7	1.03	415.05	
291	P02768	D269	YIC(CAM)ENQD^{HETETE}SISSKLK	924.93	2	17.1	1.18	474.70	
292	P02768	D269	YIC(CAM)ENQD^{HETETE}SISSKLK	616.96	3	17.2	0.92	553.50	
293	P02768	D269	YIC(CAM)ENQD^{HETETE}SISSK	804.34	2	16.4	1.06	426.76	
294	P02768	E266	YIC(CAM)E^{HETETE}NQDSISSK	804.34	2	17.3	1.04	420.82	[30]
295	P02768	E266	YIC(CAM)E^{HETETE}N(UniMod:7)QDSISSK	804.83	2	17.9	1.05	425.34	
296	P02768	E479	VTKC(CAM)C(CAM)TE^{HETETE}SLVNR	544.25	3	13.2	0.81	488.31	
297	P02768	H247	VHTECCH^{HETETE}GDLLEC(CAM)ADDRADLAK	879.04	3	16.3	1.06	636.73	
298	P02768	C245	VHTEC^{HETETE}C(CAM)HGDLLEC(CAM)ADDRADLAK	898.05	3	17.6	1.09	655.68	
299	P02768	C245	VHTEC^{HETETE}C(CAM)HGDLLEC(CAM)ADDRADLAK	673.79	4	17.7	0.97	778.83	
300	P02768	E382	VFDEFKPLVE^{HETETE}EPQNLIK	1105.07	2	28.4	1.30	524.74	
301	P02768	E382	VFDEFKPLVE^{HETEOETE}EPQNLIK	1127.08	2	28.3	1.29	520.01	
302	P02768	E382	VFDEFKPLVE^{HETE}EPQNLIK	1075.07	2	26.5	1.29	518.65	
303	P02768	E376	VFDE^{HETETE}FKPLVEEPQNLIK	1105.07	2	29.2	1.28	516.85	
304	P02768	E376	VFDE^{HETETE}FKPLVEEPQNLIK	737.05	3	29.2	0.99	596.07	
305	P02768	E376	VFDE^{HETE}FKPLVEEPQNLIK	1075.07	2	27.3	1.27	510.64	
306	P02768	E376	VFDE^{HETE}FKPLVEEPQNLIK	717.05	3	27.3	0.99	599.53	
307	P02768	D375	VFD^{HETETE}EFKPLVEEPQNLIK	737.05	3	28.4	0.99	595.96	
308	P02768	D375	VFD^{HETE}EFKPLVEEPQNLIK	1075.07	2	27.3	1.27	511.26	
309	P02768	E354	TYE^{HETETE}TTLEK	574.76	2	17.5	0.92	373.26	
310	P02768	E354	TYE^{HETEOETE}TTLEK	596.78	2	18.2	0.98	397.05	
311	P02768	D471	TPVSD^{HETETE}RVTKC(CAM)C(CAM)TESLVNR	572.27	4	15.2	0.82	660.13	
312	P02768	C62	TC(CAM)VADESAENC^{HETETE}DKSLHTLFGDKLC(CAM)TVATLR	704.53	5	21.8	1.00	1005.90	
313	P02768	C62	TC(CAM)VADESAENC^{HETETE}DKSLHTLFGDK	868.71	3	17.6	1.05	632.15	
314	P02768	C62	TC(CAM)VADESAENC^{HETETE}DKSLHTLFGDK	651.78	4	17.8	0.91	728.15	
315	P02768	H67	TC(CAM)VADESAENC(CAM)DKSLH^{HETETE}TLFGDK	887.71	3	19.9	1.05	633.73	
316	P02768	H67	TC(CAM)VADESAENC(CAM)DKSLH^{HETETE}TLFGDK	666.04	4	20.0	0.94	754.08	
317	P02768	E60	TC(CAM)VADESAE^{HETETE}NC(CAM)DKSLHTLFGDK	887.71	3	17.7	1.05	630.63	
318	P02768	E60	TC(CAM)VADESAE^{HETETE}NC(CAM)DKSLHTLFGDK	666.04	4	18.8	0.99	799.86	
319	P02768	E60	TC(CAM)VADESAE^{HETETE}NC(CAM)DK	831.81	2	13.7	1.05	423.37	
320	P02768	E57	TC(CAM)VADE^{HETETE}SAENC(CAM)DK	831.81	2	14.4	1.05	424.63	

Table S11 continued

	Protein	Mod. Site[a]	Peptide	m/z	z	RT [min]	IM [V s cm^{-2}]	CCS[b] [Å^2]	Ref.[c]
321	P02768	C75	SLHTLFGDKLC^{HETETE}TVATLR	510.52	4	21.3	0.86	695.53	
322	P02768	C75	SLHTLFGDKLC^{HETEOETE}TVATLR	521.52	4	21.6	0.90	721.23	
323	P02768	C75	SLHTLFGDKLC^{HETE}TVATLR	990.03	2	23.4	1.26	505.92	
324	P02768	C75	SLHTLFGDKLC^{HETE}TVATLR	660.35	3	19.9	0.90	545.91	
325	P02768	C75	SLHTLFGDKLC^{HETE}TVATLR	495.52	4	19.9	0.83	668.24	
326	P02768	D72	SLHTLFGD^{HETETE}KLCTVATLR	510.52	4	21.3	0.88	708.46	
327	P02768	D72	SLHTLFGD^{HETETE}KLC(CAM)TVATLR	1048.54	2	24.2	1.31	527.24	
328	P02768	D72	SLHTLFGD^{HETETE}KLC(CAM)TVATLR	524.77	4	24.2	0.88	707.32	
329	P02768	D72	SLHTLFGD^{HETEOETE}KLCTVATLR	521.52	4	21.6	0.88	711.05	
330	P02768	H67	SLH^{HETETE}TLFGDKLC(CAM)TVATLR	1048.54	2	23.5	1.25	502.18	[31]
331	P02768	H67	SLH^{HETETE}TLFGDKLC(CAM)TVATLR	699.36	3	23.5	0.97	585.38	[31]
332	P02768	H67	SLH^{HETETE}TLFGDKLC(CAM)TVATLR	524.77	4	24.2	0.89	720.57	[31]
333	P02768	H67	SLH^{HETETE}TLFGDK	591.29	2	20.4	0.93	375.56	[31]
334	P02768	H67	SLH^{HETETE}TLFGDK	394.53	3	20.3	0.72	439.13	[31]
335	P02768	H67	SLH^{HETEOETE}TLFGDK	613.30	2	20.7	0.95	385.88	
336	P02768	H67	SLH^{HETEOETE}TLFGDK	409.20	3	20.8	0.73	444.12	
337	P02768	H67	SLH^{HETE}TLFGDK	561.29	2	17.5	0.93	376.49	
338	P02768	H67	SLH^{HETE}TLFGDK	374.53	3	16.7	0.72	439.80	
339	P02768	C289 E292	SHC^{HETETE}IAE^{HETETE}VENDEMPADLPSLAADFVESK	812.10	4	25.1	1.03	824.86	
340	P02768	H288	SH^{HETETE}C(CAM)IAEVENDEMPADLPSLAADFVESK	1046.80	3	26.4	1.07	645.30	
341	P02768	E292	S(UniMod:1)HC(CAM)IAE^{HETETE}VENDEMPADLPSLAADFVESK	795.85	4	27.8	1.07	858.15	
342	P02768	E167	RYKAAFTE^{HETETE}C(CAM)C(CAM)QAADK	661.62	3	12.3	0.86	522.08	
343	P02768	E167	RYKAAFTE^{HETETE}C(CAM)C(CAM)QAADK	496.47	4	12.3	0.82	660.19	
344	P02768	E495	RPC(CAM)FSALEVDE^{HETETE}TYVPK	1037.99	2	24.3	1.23	495.09	
345	P02768	E495	RPC(CAM)FSALEVDE^{HETETE}TYVPK	692.33	3	24.3	0.97	587.27	
346	P02768	E153	RHPYFYAPE^{HETETE}LLFFAKR	740.38	3	26.7	1.04	630.12	
347	P02768	E153	RHPYFYAPE^{HETETE}LLFFAKR	555.54	4	26.9	0.87	699.24	
348	P02768	E153	RHPYFYAPE^{HETEOETE}LLFFAKR	566.54	4	26.8	0.90	727.53	
349	P02768	E153	RHPYFYAPE^{HETE}LLFFAKR	540.54	4	25.3	0.86	690.13	
350	P02768	D340	RHPD^{HETETE}YSVVLLLR	544.63	3	26.4	0.86	520.17	
351	P02768	D340	RHPD^{HETEOETE}YSVVLLLR	559.31	3	27.4	0.91	551.79	
352	P02768	H146	RH^{HETETE}PYFYAPELLFFAK	1032.02	2	29.2	1.26	507.63	
353	P02768	H146	RH^{HETETE}PYFYAPELLFFAK	688.35	3	28.9	0.98	590.99	
354	P02768	H146	RH^{HETETE}PYFYAPELLFFAK	516.51	4	28.8	0.81	651.91	

Table S11 continued

	Protein	Mod. Site[a]	Peptide	m/z	z	RT [min]	IM [V s cm^{-2}]	CCS[b] [Å^2]	Ref.[c]
355	P02768	H338	RH^{HETETE}PDYSVVLLLR	816.44	2	23.6	1.13	457.20	[30]
356	P02768	H338	RH^{HETETE}PDYSVVLLLR	544.63	3	23.7	0.90	547.47	[30]
357	P02768	H338 D340	RH^{HETETE}PD^{HETETE}YSVVLLLR	599.31	3	24.4	0.89	535.84	
358	P02768	H146	RH^{HETEOETE}PYFYAPELLFFAK	527.52	4	29.1	0.82	661.77	
359	P02768	H338	RH^{HETEOETE}PDYSVVLLLR	838.45	2	23.6	1.15	463.68	
360	P02768	H338	RH^{HETEOETE}PDYSVVLLLR	559.31	3	23.2	0.88	532.65	
361	P02768	H338 D337	RH^{HETEOETE}PD^{HETEOETE}YSVVLLLR	628.66	3	24.9	0.92	557.73	
362	P02768	H146	RH^{HETE}PYFYAPELLFFAK	1002.02	2	27.9	1.25	502.97	
363	P02768	H146	RH^{HETE}PYFYAPELLFFAK	668.35	3	27.9	0.91	547.61	
364	P02768	H146	RH^{HETE}PYFYAPELLFFAK	501.51	4	28.4	0.81	651.06	
365	P02768	H338	RH^{HETE}PDYSVVLLLR	786.44	2	21.8	1.12	451.22	
366	P02768	H338	RH^{HETE}PDYSVVLLLR	524.63	3	21.8	0.91	553.37	
367	P02768	H338	R(UniMod:1)H^{HETETE}PDYSVVLLLR	558.63	3	25.0	0.91	550.62	
368	P02768	E531	QTALVE^{HETETE}LVK	582.82	2	23.9	0.93	375.70	[30]
369	P02768	E531	QTALVE^{HETEOETE}LVK	604.84	2	24.3	0.96	388.60	
370	P02768	C392	QNC^{HETETE}ELFEQLGEYK	882.89	2	26.9	1.11	448.35	
371	P02768	C392	QNC^{HETEOETE}ELFEQLGEYK	904.90	2	27.1	1.14	458.23	
372	P02768	C392	QNC^{HETE}ELFEQLGEYK	852.88	2	24.5	1.11	446.11	
373	P02768	E400	QNC(CAM)ELFEQLGE^{HETETE}YK	911.40	2	25.3	1.15	464.88	
374	P02768	E396	QNC(CAM)ELFE^{HETETE}QLGEYK	911.40	2	25.1	1.14	458.95	[30]
375	P02768	E393	QNC(CAM)E^{HETETE}LFEQLGEYK	911.40	2	25.4	1.13	457.09	[30]
376	P02768	H105	QEPERNEC(CAM)FLQH^{HETETE}K	939.92	2	14.4	1.13	457.38	[30]
377	P02768	H105	QEPERNEC(CAM)FLQH^{HETETE}K	626.95	3	14.8	0.88	533.29	[30]
378	P02768	H105	QEPERN(UniMod:7)EC(CAM)FLQH^{HETETE}K	627.28	3	15.4	0.90	545.14	
379	P02768	E97	QEPE^{HETETE}RNEC(CAM)FLQHKDDNPNLPR	700.82	4	16.1	1.01	812.88	
380	P02768	E97	QEPE^{HETETE}RNEC(CAM)FLQHKDDNPNLPR	560.86	5	15.7	0.86	868.37	
381	P02768	E95	QE^{HETETE}PERNEC(CAM)FLQHKDDNPNLPR	700.82	4	16.1	1.02	817.27	
382	P02768	E95	QE^{HETETE}PERNEC(CAM)FLQHKDDNPNLPR	560.86	5	16.5	0.88	883.90	
383	P02768	D107	NEC(CAM)FLQHKD^{HETETE}DNPNLPR	541.00	4	16.7	0.83	668.56	[30]
384	P02768	H105	NEC(CAM)FLQH^{HETETE}KDDNPNLPR	541.00	4	17.4	0.85	680.57	[30]
385	P02768	E100	NE^{HETETE}C(CAM)FLQHKDDNPNLPR	1080.98	2	18.0	1.25	502.55	[30]
386	P02768	E100	NE^{HETETE}C(CAM)FLQHKDDNPNLPR	720.99	3	18.8	0.99	595.83	[30]
387	P02768	E100	NE^{HETETE}C(CAM)FLQHKDDNPNLPR	541.00	4	18.3	0.86	689.09	[30]

Table S11 continued

	Protein	Mod. Site[a]	Peptide	m/z	z	RT [min]	IM [V s cm^{-2}]	CCS[b] [Å^2]	Ref.[c]
388	P02768	E100	NE^{HETETE}C(CAM)FLQHK	620.27	2	17.8	0.94	378.71	
389	P02768	E100 H105	NE^{HETETE}C(CAM)FLQH^{HETETE}KDDNPNLPR	775.67	3	20.2	1.00	600.90	
390	P02768	E100 H105	NE^{HETETE}C(CAM)FLQH^{HETETE}KDDNPNLPR	582.00	4	20.2	0.88	710.82	
391	P02768	E131	LVRPEVDVMCTAFHDN(UniMod:7)E^{HETETE}ETFLKK	722.34	4	22.2	1.01	811.23	
392	P02768	E131	LVRPEVDVMCTAFHDN(UniMod:7)E^{HETEOETE}ETFLKK	733.35	4	23.0	0.98	791.37	
393	P02768	E132	LVRPEVDVMC(CAM)TAFHDNEE^{HETETE}TFLKK	981.47	3	24.2	1.18	713.67	[30]
394	P02768	E132	LVRPEVDVMC(CAM)TAFHDNEE^{HETETE}TFLKK	736.35	4	24.1	0.99	795.68	[30]
395	P02768	E132	LVRPEVDVMC(CAM)TAFHDNEE^{HETETE}TFLKK	589.28	5	24.1	0.89	898.53	[30]
396	P02768	E131	LVRPEVDVMC(CAM)TAFHDNE^{HETETE}ETFLKK	981.47	3	25.2	1.18	712.62	
397	P02768	E131	LVRPEVDVMC(CAM)TAFHDNE^{HETETE}ETFLKK	736.35	4	25.2	0.99	794.47	
398	P02768	E131	LVRPEVDVMC(CAM)TAFHDNE^{HETETE}ETFLKK	589.28	5	24.2	0.89	890.78	
399	P02768	E131	LVRPEVDVMC(CAM)TAFHDNE^{HETETE}ETFLK	938.77	3	26.9	1.08	652.73	
400	P02768	E131	LVRPEVDVMC(CAM)TAFHDNE^{HETETE}ETFLK	704.33	4	26.9	0.98	790.59	
401	P02768	E132	LVRPEVDVMC(CAM)TAFHDN(UniMod:7)EE^{HETETE}TFLK	704.58	4	26.2	1.00	802.18	
402	P02768	D129	LVRPEVDVMC(CAM)TAFHD^{HETETE}NEETFLKK	736.35	4	22.7	0.99	797.49	[30]
403	P02768	D129	LVRPEVDVMC(CAM)TAFHD^{HETETE}NEETFLKK	589.28	5	25.2	0.88	883.92	[30]
404	P02768	D129	LVRPEVDVMC(CAM)TAFHD^{HETETE}NEETFLK	704.33	4	24.9	0.98	784.07	[30]
405	P02768	D129	LVRPEVDVMC(CAM)TAFHD^{HETETE}N(UniMod:7)EETFLKK	736.60	4	23.2	0.99	796.24	
406	P02768	D129	LVRPEVDVMC(CAM)TAFHD^{HETETE}N(UniMod:7)EETFLKK	589.48	5	24.2	0.87	870.23	
407	P02768	H128	LVRPEVDVMC(CAM)TAFH^{HETETE}DNEETFLKK	736.35	4	23.4	0.99	796.15	[31]
408	P02768	H128	LVRPEVDVMC(CAM)TAFH^{HETETE}DNEETFLKK	589.28	5	23.5	0.89	891.89	[31]
409	P02768	H128	LVRPEVDVMC(CAM)TAFH^{HETETE}DNEETFLK	1407.65	2	24.4	1.44	580.08	
410	P02768	H128	LVRPEVDVMC(CAM)TAFH^{HETETE}DNEETFLK	938.77	3	24.9	1.07	644.17	
411	P02768	H128	LVRPEVDVMC(CAM)TAFH^{HETETE}DNEETFLK	704.33	4	25.5	0.97	780.85	
412	P02768	H128 E131	LVRPEVDVMC(CAM)TAFH^{HETETE}DNE^{HETETE}ETFLKK	777.36	4	26.5	1.01	807.69	
413	P02768	D56	LVNEVTEFAKTC(CAM)VAD^{HETETE}ESAENC(CAM)DK	931.74	3	21.5	1.13	683.07	
414	P02768	E45	LVN(UniMod:7)E^{HETETE}VTEFAK	657.82	2	23.0	1.00	402.51	
415	P02768	C278	LKEC^{HETETE}C(CAM)EKPLLEK	827.41	2	15.6	1.13	454.13	
416	P02768	C278	LKEC^{HETETE}C(CAM)EKPLLEK	414.21	4	15.7	0.74	598.11	
417	P02768	C279	LKEC(CAM)C^{HETETE}EKPLLEK	827.41	2	15.8	1.12	451.08	
418	P02768	C279	LKEC(CAM)C^{HETETE}EKPLLEK	551.94	3	15.8	0.88	533.75	
419	P02768	E280	LKEC(CAM)C(CAM)E^{HETETE}KPLLEK	855.92	2	16.5	1.11	448.86	

Table S11 continued

	Protein	Mod. Site[a]	Peptide	m/z	z	RT [min]	IM [V s cm^{-2}]	CCS[b] [Å^2]	Ref.[c]
420	P02768	E280	LKEC(CAM)C(CAM)E^{HETETE}KPLLEK	570.95	3	16.8	0.88	532.95	
421	P02768	E280	LKEC(CAM)C(CAM)E^{HETETE}KPLLEK	428.46	4	13.8	0.75	604.27	
422	P02768	E277	LKE^{HETETE}C(CAM)C(CAM)EKPLLEK	570.95	3	13.2	0.92	556.76	
423	P02768	E277	LKE^{HETETE}C(CAM)C(CAM)EKPLLEK	428.46	4	13.0	0.78	628.82	
424	P02768	E188	LDELRDE^{HETETE}GKASSAK	841.91	2	11.4	1.13	457.12	
425	P02768	E188	LDELRDE^{HETETE}GKASSAK	561.61	3	11.4	0.88	529.89	
426	P02768	E188	LDELRDE^{HETEOETE}GKASSAK	863.92	2	12.2	1.15	463.77	
427	P02768	E188	LDELRDE^{HETEOETE}GKASSAK	576.28	3	12.2	0.88	534.50	
428	P02768	E188	LDELRDE^{HETE}GKASSAK	811.91	2	9.4	1.10	444.37	
429	P02768	E188	LDELRDE^{HETE}GKASSAK	541.61	3	9.4	0.86	522.22	
430	P02768	C75	LC^{HETETE}TVATLR	520.77	2	20.8	0.87	352.11	
431	P02768	C75	LC^{HETEOETE}TVATLR	542.78	2	21.4	0.92	371.67	
432	P02768	E354	LAKTYE^{HETETE}TTLEK	730.87	2	14.4	1.06	427.20	
433	P02768	E354	LAKTYE^{HETETE}TTLEK	487.58	3	14.5	0.80	486.93	
434	P02768	E354	LAKTYE^{HETEOETE}TTLEK	752.89	2	15.0	1.07	433.73	
435	P02768	E354	LAKTYE^{HETEOETE}TTLEK	502.26	3	15.0	0.83	503.81	
436	P02768	E354	LAKTYE^{HETE}TTLEK	700.87	2	12.5	1.01	409.57	
437	P02768	E354	LAKTYE^{HETE}TTLEK	467.58	3	12.5	0.79	481.19	
438	P02768	E425	KVPQVSTPTLVE^{HETETE}VSR	902.49	2	21.9	1.17	471.13	[30]
439	P02768	E425	KVPQVSTPTLVE^{HETETE}VSR	601.99	3	22.0	0.82	498.56	[30]
440	P02768	E425	KVPQVSTPTLVE^{HETEOETE}VSR	924.50	2	22.1	1.18	476.70	
441	P02768	E425	KVPQVSTPTLVE^{HETEOETE}VSR	616.67	3	22.3	0.84	509.90	
442	P02768	E425	KVPQVSTPTLVE^{HETE}VSR	872.49	2	19.9	1.15	462.81	
443	P02768	E425	KVPQVSTPTLVE^{HETE}VSR	581.99	3	19.9	0.81	489.87	
444	P02768	H535	KQTALVELVKH^{HETETE}KPK	595.02	3	12.6	0.91	552.66	
445	P02768	H535	KQTALVELVKH^{HETEOETE}KPK	609.69	3	13.0	0.93	563.04	
446	P02768	H535	KQTALVELVKH^{HETEOETE}KPK	457.52	4	16.1	0.77	618.89	
447	P02768	H535	KQTALVELVKH^{HETE}KPK	575.01	3	10.1	0.91	551.39	
448	P02768	E531	KQTALVE^{HETETE}LVK	646.87	2	20.7	0.98	395.43	[30]
449	P02768	E531	KQTALVE^{HETEOETE}LVK	668.88	2	20.8	1.00	405.61	
450	P02768	E531	KQTALVE^{HETE}LVK	616.87	2	18.1	0.97	390.97	
451	P02768	E153	HPYFYAPE^{HETETE}LLFFAKR	688.35	3	29.0	0.98	589.59	
452	P02768	E153	HPYFYAPE^{HETE}LLFFAKR	1002.02	2	27.9	1.32	530.37	
453	P02768	E227	FPKAE^{HETETE}FAEVSK	708.85	2	17.7	1.01	409.88	[30]
454	P02768	E227	FPKAE^{HETETE}FAEVSK	472.90	3	17.8	0.78	471.67	[30]

Table S11 continued

	Protein	Mod. Site[a]	Peptide	m/z	z	RT [min]	IM [V s cm^{-2}]	CCS[b] [Å^2]	Ref.[c]
455	P02768	E227	FPKAE^{HETE}FAEVSK	678.85	2	16.2	1.01	409.32	
456	P02768	E227	FPKAE^{HETE}FAEVSK	452.90	3	16.2	0.79	481.94	
457	P02768	E208	FGE^{HETETE}RAFKAWAVAR	558.29	3	17.6	0.94	570.93	
458	P02768	E208	FGE^{HETETE}RAFKAWAVAR	418.97	4	17.5	0.79	633.81	
459	P02768	E208	FGE^{HETEOETE}RAFKAWAVAR	572.96	3	17.8	0.99	596.69	
460	P02768	E208	FGE^{HETEOETE}RAFKAWAVAR	429.97	4	17.8	0.80	647.52	
461	P02768	E208	FGE^{HETE}RAFKAWAVAR	538.29	3	15.5	0.88	532.73	
462	P02768	E208	FGE^{HETE}RAFKAWAVAR	403.97	4	15.5	0.75	605.38	
463	P02768	C91	ETYGEMADC(CAM)C^{HETETE}AK	771.28	2	17.6	1.04	419.32	
464	P02768	E86	ETYGE^{HETETE}MADC(CAM)C(CAM)AK	799.79	2	17.0	1.03	416.90	
465	P02768	E571	ETC(CAM)FAEE^{HETETE}GKK	681.79	2	13.9	1.01	407.57	
466	P02768	E571	ETC(CAM)FAEE^{HETETE}GKK	454.86	3	12.2	0.75	455.01	
467	P02768	H510	EFNAETFTFH^{HETETE}ADIC(CAM)TLSEKER	903.74	3	24.3	1.11	666.89	
468	P02768	H510	EFNAETFTFH^{HETETE}ADIC(CAM)TLSEKER	678.06	4	24.2	0.97	781.58	
469	P02768	H510	EFNAETFTFH^{HETETE}ADIC(CAM)TLSEK	1212.53	2	25.7	1.37	552.72	
470	P02768	C278	EC^{HETETE}C(CAM)EKPLLEK	706.82	2	16.1	1.02	411.81	
471	P02768	E285	EC(CAM)C(CAM)EKPLLE^{HETETE}K	735.33	2	17.2	1.04	419.81	
472	P02768	E285	EC(CAM)C(CAM)EKPLLE^{HETETE}K	490.56	3	17.2	0.77	464.33	
473	P02768	E280	EC(CAM)C(CAM)E^{HETETE}KPLLEK	735.33	2	17.6	1.05	424.57	
474	P02768	E82	E^{HETETE}TYGEMADC(CAM)C(CAM)AK	799.79	2	19.9	1.04	418.35	
475	P02768	E277	E^{HETETE}CC(CAM)EKPLLEK	471.55	3	16.0	0.83	500.80	
476	P02768	E277	E^{HETETE}C(CAM)C(CAM)EKPLLEK	735.33	2	15.2	1.06	428.21	
477	P02768	E277	E^{HETETE}C(CAM)C(CAM)EKPLLEK	490.56	3	17.6	0.84	507.36	
478	P02768	E333	DVFLGMFLYE^{HETETE}YAR	894.41	2	29.2	1.21	486.47	
479	P02768	E333	DVFLGMFLYE^{HETETE}YAR	596.61	3	28.7	0.84	508.94	
480	P02768	E333	DVFLGMFLYE^{HETEOETE}YAR	916.43	2	29.2	1.22	491.58	
481	P02768	E333	DVFLGMFLYE^{HETEOETE}YAR	611.29	3	28.8	0.87	527.79	
482	P02768	E333	DVFLGMFLYE^{HETE}YAR	864.41	2	27.2	1.19	478.29	
483	P02768	H3	DAH^{HETETE}KSEVAHR	657.31	2	6.9	0.95	385.63	
484	P02768	H3	DAH^{HETETE}KSEVAHR	438.54	3	7.0	0.83	501.40	
485	P02768	H3	DAH^{HETEOETE}KSEVAHR	453.22	3	7.4	0.85	516.91	
486	P02768	D324	D^{HETETE}VFLGMFLYEYAR	894.41	2	28.6	1.18	474.39	[30]
487	P02768	D324	D^{HETETE}VFLGMFLYEYAR	596.61	3	28.8	0.84	506.97	[30]
488	P02768	D1	D^{HETETE}AHKSEVAHR	657.31	2	6.9	0.95	383.21	
489	P02768	D1	D^{HETETE}AHKSEVAHR	438.54	3	7.0	0.75	453.00	
490	P02768	D1	D^{HETETE}AHKSEVAHR	329.16	4	6.7	0.71	574.02	

Table S11 continued

	Protein	Mod. Site[a]	Peptide	m/z	z	RT [min]	IM [$\mathrm{V\,s\,cm^{-2}}$]	CCS[b] [$\mathrm{Å^2}$]	Ref.[c]
491	P02768	D324	D^{HETEOETE}VFLGMFLYEYAR	916.43	2	28.8	1.20	483.00	
492	P02768	D324	D^{HETEOETE}VFLGMFLYEYAR	611.29	3	28.9	0.87	525.46	
493	P02768	D1	D^{HETEOETE}AHKSEVAHR	453.22	3	7.6	0.75	456.75	
494	P02768	D1	D^{HETEOETE}AHKSEVAHR	340.16	4	7.5	0.71	572.82	
495	P02768	D324	D^{HETE}VFLGMFLYEYAR	576.61	3	28.2	0.84	508.64	
496	P02768	C437	C^{HETETE}C(CAM)KHPEAK	568.74	2	7.0	0.89	360.95	
497	P02768	C437	C^{HETETE}C(CAM)KHPEAK	379.50	3	7.3	0.75	455.30	
498	P02768	C361	C(CAM)C^{HETETE}AAADPHEC(CAM)YAK	830.31	2	13.2	1.07	430.45	
499	P02768	C361	C(CAM)C^{HETETE}AAADPHEC(CAM)YAK	553.87	3	13.2	0.90	542.03	
500	P02768	E479	C(CAM)C(CAM)TE^{HETETE}SLVNR	651.77	2	17.8	0.96	389.81	
501	P02768	E442	C(CAM)C(CAM)KHPE^{HETETE}AK	597.25	2	7.4	0.92	373.81	
502	P02768	E442	C(CAM)C(CAM)KHPE^{HETETE}AK	398.50	3	6.9	0.73	441.69	
503	P02768	E208	C(CAM)ASLQKFGE^{HETETE}R	680.31	2	14.7	1.01	409.91	
504	P02768	E556	AVMDDFAAFVE^{HETETE}KC(CAM)C(CAM)K	977.92	2	26.4	1.27	512.64	[30]
505	P02768	E556	AVMDDFAAFVE^{HETETE}KC(CAM)C(CAM)K	652.28	3	26.5	0.90	544.21	[30]
506	P02768	E556	AVMDDFAAFVE^{HETETE}K	753.84	2	28.0	1.03	416.64	[30]
507	P02768	E556	AVMDDFAAFVE^{HETEOETE}K	775.85	2	28.2	1.05	422.77	
508	P02768	E556	AVMDDFAAFVE^{HETE}K	723.84	2	27.4	1.07	432.00	
509	P02768	C34	ALVLIAFAQYLQQC^{HETETE}PFEDHVK	1299.15	2	29.2	1.38	556.85	[32–35]
510	P02768	C34	ALVLIAFAQYLQQC^{HETETE}PFEDHVK	866.44	3	29.2	1.09	657.95	[32–35]
511	P02768	C34	ALVLIAFAQYLQQC^{HETETE}PFEDHVK	650.08	4	29.2	0.86	695.26	[32–35]
512	P02768	C34	ALVLIAFAQYLQQC^{HETEOETE}PFEDHVK	1321.16	2	29.2	1.39	560.29	
513	P02768	C34	ALVLIAFAQYLQQC^{HETEOETE}PFEDHVK	881.11	3	29.2	1.10	662.08	
514	P02768	C34	ALVLIAFAQYLQQC^{HETEOETE}PFEDHVK	661.09	4	29.2	0.88	705.93	
515	P02768	C34	ALVLIAFAQYLQQC^{HETE}PFEDHVK	846.44	3	29.2	1.08	654.10	
516	P02768	D237	AEFAEVSKLVTD^{HETETE}LTK	907.97	2	24.2	1.24	499.82	[31]
517	P02768	D237	AEFAEVSKLVTD^{HETETE}LTK	605.65	3	28.0	0.86	518.59	[31]
518	P02768	D237	AEFAEVSKLVTD^{HETEOETE}LTK	929.98	2	27.6	1.25	504.29	
519	P02768	D237	AEFAEVSKLVTD^{HETEOETE}LTK	620.32	3	27.6	0.88	530.00	
520	P02768	D237	AEFAEVSKLVTD^{HETE}LTK	877.97	2	27.9	1.22	490.60	
521	P02768	E230	AEFAE^{HETETE}VSK	522.74	2	19.0	0.84	339.68	[35,36]
522	P02768	E230	AEFAE^{HETE}VSK	492.74	2	15.3	0.81	329.94	
523	P02768	C567	ADDKETC^{HETETE}FAEEGKK	867.87	2	13.4	1.09	440.39	
524	P02768	C567	ADDKETC^{HETETE}FAEEGKK	578.92	3	13.6	0.84	510.81	
525	P02768	C567	ADDKETC^{HETEOETE}FAEEGKK	889.89	2	14.4	1.12	451.35	

Table S11 continued

	Protein	Mod. Site[a]	Peptide	m/z	z	RT [min]	IM [V s cm^{-2}]	CCS[b] [Å^2]	Ref.[c]
526	P02768	C567	ADDKETC^{HETEOETE}FAEEGKK	593.59	3	14.4	0.87	523.76	
527	P02768	C567	ADDKETC^{HETE}FAEEGKK	837.87	2	10.2	1.08	436.91	
528	P02768	C567	ADDKETC^{HETE}FAEEGKK	558.92	3	10.0	0.85	511.29	
529	P02768	E571	ADDKETC(CAM)FAEE^{HETETE}GKK	896.38	2	13.9	1.15	464.87	
530	P02768	E571	ADDKETC(CAM)FAEE^{HETETE}GKK	597.92	3	13.6	0.86	521.66	
531	P02768	E571	ADDKETC(CAM)FAEE^{HETETE}GK	832.34	2	16.1	1.09	438.02	
532	P02768	E571	ADDKETC(CAM)FAEE^{HETETE}GK	555.23	3	15.6	0.78	474.72	
533	P02768	E570	ADDKETC(CAM)FAE^{HETETE}EGKK	896.38	2	13.5	1.17	471.43	
534	P02768	E570	ADDKETC(CAM)FAE^{HETETE}EGKK	597.92	3	13.5	0.86	519.77	
535	P02768	E565	ADDKE^{HETETE}TC(CAM)FAEEGKK	896.38	2	11.3	1.12	450.13	
536	P02768	E565	ADDKE^{HETETE}TC(CAM)FAEEGKK	597.92	3	11.9	0.87	526.56	
537	P02768	D563	ADD^{HETETE}KETC(CAM)FAEEGKK	597.92	3	11.6	0.86	521.86	
538	P02768	D563	ADD^{HETETE}KETC(CAM)FAEEGK	832.34	2	12.8	1.06	427.30	
539	P02768	D563	ADD^{HETETE}KETC(CAM)FAEEGK	555.23	3	13.1	0.83	501.54	
540	P02768	D173	AAFTEC(CAM)CQAAD^{HETETE}K	739.79	2	18.2	1.03	414.05	
541	P02768	D173	AAFTEC(CAM)C(CAM)QAAD^{HETETE}K	768.30	2	15.9	1.04	421.52	
542	P02768	E167	AAFTE^{HETETE}C(CAM)C(CAM)QAADK	768.30	2	17.5	1.04	418.50	
543	P02768	D183	AAC(CAM)LLPKLD^{HETETE}ELRDEGK	664.67	3	21.9	0.93	563.43	
544	P02768	D183	AAC(CAM)LLPKLD^{HETETE}ELRDEGK	498.75	4	21.8	0.80	645.67	
545	P02768	D183	AAC(CAM)LLPKLD^{HETETE}ELR	781.91	2	23.5	1.13	455.99	
546	P02774	E212	VC(CAM)SQYAAYGE^{HETETE}KK	784.35	2	12.6	1.09	439.83	
547	P02774	E212	VC(CAM)SQYAAYGE^{HETETE}KK	523.24	3	12.7	0.82	495.13	
548	P02774	H339	TH^{HETETE}LPEVFLSK	667.85	2	21.5	1.01	406.52	
549	P02774	H339	TH^{HETE}LPEVFLSK	637.84	2	19.9	0.99	400.45	
550	P02774	H192	H^{HETETE}LSLLTTLSNR	709.88	2	23.2	1.03	417.02	
551	P02774	H192	H^{HETE}LSLLTTLSNR	679.88	2	20.8	1.02	413.78	
552	P02787	E654	YLGEE^{HETETE}YVK	582.77	2	20.5	0.93	375.40	
553	P02787	E654	YLGEE^{HETE}YVK	552.77	2	17.3	0.90	365.79	
554	P02787	H14	WC(CAM)AVSEH^{HETETE}EATK	741.31	2	13.8	1.06	428.15	
555	P02787	H14	WC(CAM)AVSEH^{HETETE}EATK	494.55	3	13.8	0.77	466.47	
556	P02787	D442	SASDLTWD^{HETETE}NLKGK	799.88	2	19.8	1.13	454.14	
557	P02787	D442	SASDLTWD^{HETEOETE}NLKGK	821.89	2	20.2	1.15	462.76	
558	P02787	C577	KPVEEYANC^{HETETE}HLAR	565.27	3	15.8	0.86	520.59	
559	P02787	C577	KPVEEYANC^{HETEOETE}HLAR	579.94	3	16.2	0.86	519.87	
560	P02787	H578	KPVEEYANC(CAM)H^{HETETE}LAR	875.91	2	13.2	1.14	458.44	
561	P02787	H578	KPVEEYANC(CAM)H^{HETETE}LAR	584.27	3	13.2	0.88	534.06	

Table S11 continued

	Protein	Mod. Site[a]	Peptide	m/z	z	RT [min]	IM [V s cm^{-2}]	CCS[b] [Å^2]	Ref.[c]
562	P02787	E573	KPVEE^{HETETE}YANC(CAM)HLAR	584.27	3	14.5	0.87	525.72	
563	P02787	H249	KPVDEYKDC(CAM)HLAQVPSH^{HETETE}TVVAR	905.11	3	15.5	1.05	630.78	
564	P02787	H249	KPVDEYKDC(CAM)HLAQVPSH^{HETETE}TVVAR	543.47	5	15.8	0.90	905.31	
565	P02787	E367	IE^{HETETE}C(CAM)VSAETTEDC(CAM)IAK	945.40	2	19.4	1.16	468.14	
566	P02787	E482	FDEFFSE^{HETETE}GC(CAM)APGSKK	623.93	3	19.8	0.91	551.21	
567	P02787	E333	EGTC(CAM)PE^{HETETE}APTDEC(CAM)KPVK	991.42	2	14.4	1.14	461.26	
568	P02787	E333	EGTC(CAM)PE^{HETETE}APTDEC(CAM)KPVK	661.28	3	14.5	0.90	545.21	
569	P02787	D90	ED^{HETETE}PQTFYYAVAVVK	897.43	2	26.3	1.13	457.21	
570	P02787	D90	ED^{HETEOETE}PQTFYYAVAVVK	919.44	2	26.8	1.15	464.47	
571	P02787	D90	ED^{HETE}PQTFYYAVAVVK	867.43	2	24.7	1.14	459.99	
572	P02787	H598	DKEAC(CAM)VH^{HETETE}KILR	511.60	3	12.5	0.77	467.97	
573	P02787	H207	DGAGDVAFVKH^{HETETE}STIFENLANK	799.72	3	23.5	1.02	615.04	
574	P02787	H249	DC(CAM)HLAQVPSH^{HETETE}TVVAR	927.44	2	16.2	1.21	488.69	
575	P02787	H249	DC(CAM)HLAQVPSH^{HETETE}TVVAR	618.63	3	16.2	0.91	547.73	
576	P02787	H585	APNH^{HETETE}AVVTR	564.79	2	10.3	0.92	373.35	
577	P02787	H585	APNH^{HETEOETE}AVVTR	586.80	2	11.7	0.92	374.19	
578	P02787	H585	APNH^{HETEOETE}AVVTR	391.54	3	11.7	0.76	460.31	
579	P02787	H585	APNH^{HETE}AVVTR	534.78	2	8.2	0.88	356.77	
580	P02787	H585	APNH^{HETE}AVVTR	356.86	3	8.2	0.73	444.43	
581	P02790	D335	EVGTPHGIILD^{HETETE}SVDAAFIC(CAM)PGSSR	888.09	3	28.0	1.03	620.22	
582	P04003	H400	SRPANHC(CAM)VYFYGDEISFSC(CAM)H^{HETETE}ETSR	771.58	4	19.9	1.03	830.82	
583	P04004	H458	SIAQYWLGC(CAM)PAPGH^{HETETE}L	917.43	2	27.6	1.15	465.36	
584	P04114	H2972	LEIQSQVDSQHVGH^{HETETE}SVLTAK	780.73	3	18.1	1.02	615.27	
585	P04114	D1086	ITEVALMGHLSCD^{HETETE}TKEER	732.68	3	21.8	1.02	618.46	
586	P04217	H50	NGVAQEPVH^{HETETE}LDSPAIK	613.64	3	18.5	0.91	551.92	
587	P04217	H50	NGVAQEPVH^{HETEOETE}LDSPAIK	628.32	3	18.9	0.88	532.78	
588	P04217	H50	NGVAQEPVH^{HETE}LDSPAIK	593.64	3	17.2	0.90	544.87	
589	P05090	E37	IPTTFE^{HETETE}NGR	599.78	2	20.1	0.90	365.27	
590	P06396	D636	MD^{HETETE}AHPPR	494.21	2	9.8	0.87	351.78	
591	P07360	E146	SLPVSDSVLSGFE^{HETETE}QR	892.93	2	28.4	1.13	454.38	
592	P07360	E146	SLPVSDSVLSGFE^{HETEOETE}QR	914.94	2	28.3	1.14	460.19	
593	P07360	E146	SLPVSDSVLSGFE^{HETE}QR	862.93	2	25.4	1.10	445.43	
594	P08185	H53	H^{HETETE}LVALSPK	514.79	2	17.1	0.90	364.12	
595	P08603	H559	IDVH^{HETETE}LVPDRK	452.58	3	16.9	0.74	447.62	
596	P08603	H559	IDVH^{HETEOETE}LVPDRK	467.25	3	17.4	0.75	455.94	
597	P08603	H319	HGGLYH^{HETETE}ENMR	459.87	3	11.9	0.79	481.43	

Table S11 continued

	Protein	Mod. Site[a]	Peptide	m/z	z	RT [min]	IM [V s cm^{-2}]	CCS[b] [Å^2]	Ref.[c]
598	P08603	E617	EQVQSC(CAM)GPPPE^{HETETE}LLNGNVK	1065.51	2	22.5	1.21	486.94	
599	P08603	D119	EC(CAM)DTDGWTND^{HETETE}IPIC(CAM)EVVK	1157.99	2	28.8	1.25	502.80	
600	P0C0L4	E1338	GLEEE^{HETETE}LQFSLGSK	800.88	2	28.6	1.07	429.95	
601	P0C0L4	E270	FGLLDE^{HETETE}DGKK	643.31	2	22.7	1.00	403.29	
602	P0C0L5	E1357	GLEEE^{HETEOETE}LQFSLGSK	822.90	2	29.1	1.08	436.42	
603	P0C0L5	E1357	GLEEE^{HETE}LQFSLGSK	770.88	2	25.9	1.05	425.12	
604	P0C0L5	E289	FGLLDE^{HETEOETE}DGKK	665.33	2	23.1	1.02	412.86	
605	P0C0L5	E289	FGLLDE^{HETE}DGKK	613.31	2	19.5	0.97	394.24	
606	P0C0L5	E289	FGLLDE^{HETE}DGKK	409.21	3	19.5	0.73	441.25	
607	P0DOX5	E271	TPEVTCVVVDVSHE^{HETETE}DPEVK	1123.52	2	25.2	1.38	555.61	
608	P0DOX5	E271	TPEVTCVVVDVSHE^{HETETE}DPEVK	749.35	3	24.6	1.02	617.89	
609	P0DOX5	E271	TPEVTCVVVDVSHE^{HETE}DPEVK	1093.52	2	23.3	1.36	547.29	
610	P0DOX5	E271	TPEVTCVVVDVSHE^{HETE}DPEVK	729.35	3	23.2	1.01	612.88	
611	P0DOX5	H270	TPEVTCVVVDVSH^{HETETE}EDPEVK	1123.52	2	22.6	1.35	544.57	
612	P0DOX5	H270	TPEVTCVVVDVSH^{HETEOETE}EDPEVK	1145.54	2	22.9	1.37	551.90	
613	P0DOX5	H270	TPEVTCVVVDVSH^{HETE}EDPEVK	1093.52	2	21.5	1.33	536.12	
614	P0DOX5	H270	TPEVTCVVVDVSH^{HETE}EDPEVK	729.35	3	23.3	1.02	616.46	
615	P0DOX5	D272	TPEVTC(CAM)VVVDVSHED^{HETETE}PEVK	1152.03	2	24.3	1.34	540.87	
616	P0DOX5	D272	TPEVTC(CAM)VVVDVSHED^{HETETE}PEVK	768.36	3	24.4	1.02	615.91	
617	P0DOX5	E271	TPEVTC(CAM)VVVDVSHE^{HETETE}DPEVK	1152.03	2	25.1	1.37	552.37	
618	P0DOX5	E271	TPEVTC(CAM)VVVDVSHE^{HETETE}DPEVK	768.36	3	25.0	1.02	614.38	
619	P0DOX5	E260	TPE^{HETETE}VTC(CAM)VVVDVSHEDPEVK	768.36	3	23.5	1.04	626.02	
620	P0DOX5	D251	THTC(CAM)PPC(CAM)PAPELLGGPSVFLFPPKPKD^{HETETE}TLMISR	956.98	4	28.3	1.15	921.13	
621	P0DOX5	D251	THTC(CAM)PPC(CAM)PAPELLGGPSVFLFPPKPKD^{HETETE}TLMISR	765.79	5	28.0	1.03	1030.28	
622	P0DOX5	E235	THTC(CAM)PPC(CAM)PAPE^{HETETE}LLGGPSVFLFPPKPK	1003.50	3	28.8	1.23	742.83	
623	P0DOX5	E235	THTC(CAM)PPC(CAM)PAPE^{HETETE}LLGGPSVFLFPPKPK	752.88	4	29.0	1.02	816.44	
624	P0DOX5	H226	TH^{HETETE}TC(CAM)PPC(CAM)PAPELLGGPSVFLFPPKPK	1003.50	3	27.4	1.28	770.50	
625	P0DOX5	H226	TH^{HETETE}TC(CAM)PPC(CAM)PAPELLGGPSVFLFPPKPK	752.88	4	27.5	1.04	838.12	
626	P0DOX5	H287	FNWYVDGVEVH^{HETETE}NAKTKPR	581.79	4	19.0	0.85	682.98	
627	P0DOX5	H287	FNWYVDGVEVH^{HETETE}NAK	921.42	2	22.5	1.23	496.37	
628	P0DOX5	H287	FNWYVDGVEVH^{HETETE}NAK	614.62	3	22.5	0.92	553.25	
629	P0DOX5	H287	FNWYVDGVEVH^{HETETE}N(UniMod:7)AK	921.91	2	22.9	1.23	497.55	
630	P0DOX5	H287	FNWYVDGVEVH^{HETETE}N(UniMod:7)AK	614.94	3	23.2	0.90	541.72	
631	P0DOX5	H287	FNWYVDGVEVH^{HETEOETE}NAK	943.43	2	22.9	1.25	504.70	
632	P0DOX5	H287	FNWYVDGVEVH^{HETEOETE}NAK	629.29	3	22.9	0.93	563.38	
633	P0DOX5	H287	FNWYVDGVEVH^{HETEOETE}N(UniMod:7)AK	943.93	2	22.4	1.23	497.65	

Table S11 continued

	Protein	Mod. Site[a]	Peptide	m/z	z	RT [min]	IM [V s cm^{-2}]	CCS[b] [Å^2]	Ref.[c]
634	P0DOX5	H287	FNWYVDGVEVH^{HETE}NAK	891.42	2	20.8	1.23	494.30	
635	P0DOX5	H287	FNWYVDGVEVH^{HETE}NAK	594.62	3	20.8	0.91	551.04	
636	P0DOX5	H287	FNWYVDGVEVH^{HETE}N(UniMod:7)AK	891.91	2	21.4	1.22	490.23	
637	P0DOX5	E285<br>H287	FNWYVDGVE^{HETETE}VH^{HETETE}NAK	1003.44	2	23.9	1.28	516.18	
638	P0DOX5	E285<br>H284	FNWYVDGVE^{HETEOETE}VH^{HETEOETE}NAK	1047.46	2	24.7	1.32	530.13	
639	P0DOX5	E285<br>H280	FNWYVDGVE^{HETE}VH^{HETE}NAK	629.29	3	21.3	0.94	567.09	
640	P0DOX7	E165	VDNALQSGNSQESVTE^{HETETE}QDSK	1150.50	2	16.1	1.25	504.02	
641	P0DOX7	E165	VDNALQSGNSQESVTE^{HETEOETE}QDSK	1172.52	2	16.8	1.26	508.52	
642	P0DOX7	E165	VDNALQSGNSQESVTE^{HETE}QDSK	1120.50	2	13.5	1.23	495.49	
643	P0DOX7	E161	VDNALQSGNSQE^{HETETE}SVTEQDSK	1150.50	2	16.4	1.23	496.43	
644	P0DOX7	E161	VDNALQSGNSQE^{HETEOETE}SVTEQDSK	1172.52	2	17.2	1.25	502.87	
645	P0DOX7	E161	VDNALQSGNSQE^{HETE}SVTEQDSK	1120.50	2	13.9	1.21	486.48	
646	P0DOX7	D1	D^{HETETE}IQMTQSPSTLSASVGDR	1028.97	2	22.1	1.21	488.78	
647	P0DOX7	D1	D^{HETEOETE}IQMTQSPSTLSASVGDR	1050.98	2	22.7	1.23	496.66	
648	P0DOX7	D1	D^{HETE}IQMTQSPSTLSASVGDR	998.97	2	20.1	1.20	484.49	
649	P0DOX7	H189	ADYEKH^{HETETE}K	527.74	2	7.6	0.87	353.27	
650	P0DOX7	H189	ADYEKH^{HETEOETE}K	549.75	2	8.3	0.90	364.41	
651	P0DOY3	E54	ADSSPVKAGVE^{HETETE}TTTPSK	613.63	3	13.0	0.90	543.41	
652	P0DOY3	E54	ADSSPVKAGVE^{HETEOETE}TTTPSK	628.31	3	13.6	0.90	544.39	
653	P0DOY3	E54	ADSSPVKAGVE^{HETE}TTTPSK	593.63	3	11.4	0.88	529.25	
654	P19652	E119	TLMFGSYLDDE^{HETETE}K	791.85	2	27.2	1.08	436.46	
655	P19652	E119	TLMFGSYLDDE^{HETEOETE}K	813.86	2	27.7	1.10	443.70	
656	P19652	E119	TLMFGSYLDDE^{HETE}K	761.84	2	24.8	1.06	429.44	
657	P19652	D129	NWGLSFYAD^{HETETE}KPETTK	640.97	3	26.0	0.90	543.33	
658	P19652	H97	EH^{HETETE}VAHLLFLR	699.87	2	22.1	1.03	416.72	
659	P19652	H97	EH^{HETETE}VAHLLFLR	466.92	3	22.1	0.85	517.88	
660	P19652	H97	EH^{HETEOETE}VAHLLFLR	721.89	2	22.4	1.05	425.67	
661	P19652	H97	EH^{HETE}VAHLLFLR	446.92	3	20.3	0.85	514.82	
662	P19652	E96	E^{HETETE}HVAHLLFLR	466.92	3	21.3	0.86	520.28	
663	P19652	E96	E^{HETEOETE}HVAHLLFLR	481.59	3	22.4	0.87	529.82	
664	P19652	E96	E^{HETE}HVAHLLFLR	446.92	3	19.8	0.84	506.54	
665	P19823	D554	D^{HETETE}KHADPDFTR	455.87	3	11.9	0.78	474.05	
666	P19827	H41	FAH^{HETETE}YVVTSQVVNTANEAR	724.02	3	19.5	1.03	622.83	

Table S11 continued

	Protein	Mod. Site[a]	Peptide	m/z	z	RT [min]	IM [V s cm^{-2}]	CCS[b] [Å^2]	Ref.[c]
667	P19827	H41	FAH^{HETEOETE}YVVTSQVVNTANEAR	738.69	3	19.9	1.05	631.47	
668	P19827	H41	FAH^{HETE}YVVTSQVVNTANEAR	1055.52	2	17.8	1.22	492.97	
669	P19827	H41	FAH^{HETE}YVVTSQVVNTANEAR	704.02	3	17.8	1.03	621.35	
670	P19827	H503	ADVQAH^{HETETE}GEGQEFSITC(CAM)LVDEEEMKK	1005.45	3	23.2	1.21	729.21	
671	P19827	H503	ADVQAH^{HETETE}GEGQEFSITC(CAM)LVDEEEMKK	754.34	4	23.3	1.00	802.36	
672	P29475	H92	GIASETH^{HETETE}VVLILR	524.63	3	22.0	0.90	545.98	
673	P29475	H92	G(UniMod:1)IASETH^{HETETE}VVLILR	538.63	3	23.2	0.86	518.13	
674	P29475	H92	G(UniMod:1)IASETH^{HETEOETE}VVLILR	553.31	3	25.6	0.92	555.83	
675	P29475	H92	G(UniMod:1)IASETH^{HETE}VVLILR	518.63	3	25.0	0.91	550.15	
676	P29475	E90	G(UniMod:1)IASE^{HETETE}THVVLILR	538.63	3	23.5	0.83	502.11	
677	P29475	E90	G(UniMod:1)IASE^{HETEOETE}THVVLILR	553.31	3	23.5	0.93	561.59	
678	P68871	H98	GTFATLSELHC(CAM)DKLH^{HETETE}VDPENFR	688.32	4	22.4	0.98	784.79	
679	P69905	H21	VGAH^{HETETE}AGEYGAEALER	565.26	3	15.3	0.82	497.65	
680	P69905	H21	VGAH^{HETE}AGEYGAEALER	545.26	3	13.4	0.83	504.23	
681	Q14624	E100	AE^{HETETE}AQAQYSAAVAK	736.35	2	15.8	1.03	416.51	
682	Q96PD5	H443	GWH^{HETETE}WVGAHTLGHNSR	626.96	3	16.5	0.92	554.88	
683	Q96PD5	H443	GWH^{HETEOETE}WVGAHTLGHNSR	961.95	2	16.8	1.26	506.70	
684	Q96PD5	H443	GWH^{HETEOETE}WVGAHTLGHNSR	641.63	3	17.0	0.93	562.33	
685	Q96PD5	H443	GWH^{HETEOETE}WVGAHTLGHNSR	481.48	4	17.0	0.80	645.44	

Table S12 Targetlist for prm-PASEF measurement using a 15 min elution gradient in the nLC (short gradient). All peptides originate from HSA. [a]Optimized collisional energy (CE).

	Peptide	Precursor [m/z]	Charge	Δm/z	RT [min]	RT Range [min]	IM Range [V s cm^{-2}]	CE[a] [eV]
1	TC^{CAM}VADESAENC^{CAM}D^{HETETE}K	831.81	2	3	7.70	2.00	0.992 – 1.092	37.20
2	ADDKETC^{CAM}FAEE^{HETETE}GKK	597.92	3	3	7.90	2.00	0.827 – 0.927	33.30
3	TC^{CAM}VADE^{HETETE}SAENC^{CAM}DK	831.81	2	3	7.90	2.00	1.015 – 1.115	40.70
4	QEPERNEC^{CAM}FLQH^{HETETE}K	626.95	3	3	8.10	2.00	0.855 – 0.955	34.40
5	Q$^{UniMod:7}$EPERNEC^{CAM}FLQH^{HETETE}KDDNPNLPR	701.07	4	3	8.50	2.00	0.900 – 1.000	38.70
6	QEPERNEC^{CAM}FLQHKDD^{HETETE}NPNLPR	560.86	5	3	8.50	2.00	0.836 – 0.936	26.10
7	AAFTEC^{CAM}C^{CAM}QAAD^{HETETE}K	768.30	2	3	8.50	2.00	1.000 – 1.100	35.00
8	LD^{HETETE}ELRDEGK	619.79	2	3	8.60	2.00	0.893 – 0.993	38.40
9	LKEC^{CAM}C^{CAM}E^{HETETE}KPLLEK	570.95	3	3	8.70	2.00	0.832 – 0.932	31.00

Table S12 continued

	Peptide	Precursor [m/z]	Charge	Δm/z	RT [min]	RT Range [min]	IM Range [V s cm^{-2}]	CE^{a} [eV]
10	YIC^{CAM}ENQD^{HETETE}SISSKLK	616.96	3	3	8.80	2.00	0.873 – 0.973	27.60
11	YIC^{CAM}E^{HETETE}NQDSISSK	804.34	2	3	9.10	2.00	0.995 – 1.095	39.90
12	AAFTE^{HETETE}C^{CAM}C^{CAM}QAADK	768.30	2	3	9.10	2.00	1.000 – 1.100	37.50
13	TYE^{HETETE}TTLEK	574.76	2	3	9.10	2.00	0.879 – 0.979	27.80
14	NE^{HETETE}C^{CAM}FLQHK	620.27	2	3	9.20	2.00	0.882 – 0.982	33.00
15	C^{CAM}C^{CAM}TE^{HETETE}SLVNR	651.77	2	3	9.30	2.00	0.923 – 1.023	37.00
16	SLH^{HETETE}TLFGDK	394.53	3	3	9.80	2.00	0.684 – 0.784	36.20
17	AAC^{CAM}LLPKLD^{HETETE}ELRDEGK	664.67	3	3	10.40	2.00	0.872 – 0.972	35.10
18	KVPQVSTPTLVE^{HETETE}VSR	602.00	3	3	10.50	2.00	0.786 – 0.886	47.00
19	LVRPEVDVMC^{CAM}TAFH^{HETETE}DNEETFLKK	589.28	5	3	10.50	2.00	0.853 – 0.953	35.00
20	LVRPEVDVMC^{CAM}TAFHD^{HETETE}NEETFLKK	736.35	4	3	10.60	2.00	0.922 – 1.022	32.00
21	LVNEVTE^{HETETE}FAK	657.33	2	3	10.80	2.00	0.932 – 1.032	29.90
22	LVRPEVDVMC^{CAM}TAFHDNEE^{HETETE}TFLKK	736.35	4	3	10.80	2.00	0.937 – 1.037	32.60
23	RH^{HETETE}PDYSVVLLLR	544.63	3	3	11.20	2.00	0.861 – 0.961	29.60
24	EFNAETFTFH^{HETETE}ADIC^{CAM}TLSEKER	903.74	3	3	11.20	2.00	1.061 – 1.161	44.90
25	LVRPE^{HETETE}VDVMC^{CAM}TAFHDNEETFLKK	736.35	4	3	11.30	2.00	0.938 – 1.038	32.60
26	RPC^{CAM}FSALEVDE^{HETETE}TYVPK	692.33	3	3	11.30	2.00	0.930 – 1.030	29.80
27	LVRPEVDVMC^{CAM}TAFHD^{HETETE}NEETFLK	704.33	4	3	11.40	2.00	0.919 – 1.019	29.40
28	LVRPEVDVMC^{CAM}TAFHDNE^{HETETE}ETFLKK	736.35	4	3	11.40	2.00	0.931 – 1.031	29.40
29	QNC^{CAM}ELFE^{HETETE}QLGEYK	911.40	2	3	11.70	2.00	1.092 – 1.192	43.60
30	D^{HETETE}VFLGMFLYEYAR	894.41	2	3	12.80	2.00	1.118 – 1.218	25.00

Table S13 Targetlist for prm-PASEF measurement using a 30 min elution gradient in the nLC (long gradient). All peptides originate from HSA. [a]Optimized collisional energy (CE).

	Peptide	Precursor [m/z]	Charge	Δm/z	RT [min]	RT Range [min]	IM Range [V s cm^{-2}]	CE^{a} [eV]
1	LDELRDE^{HETETE}GKASSAK	561.61	3	3	11.00	2.00	0.852 – 0.952	31.80
2	ADD^{HETETE}KETC^{CAM}FAEEGKK	597.92	3	3	11.20	2.00	0.824 – 0.924	30.70
3	TC^{CAM}VADESAENC^{CAM}D^{HETETE}K	831.81	2	3	12.80	2.00	0.992 – 1.092	37.20
4	ADDKETC^{CAM}FAEE^{HETETE}GKK	597.92	3	3	13.20	2.00	0.827 – 0.927	33.30
5	TC^{CAM}VADESAE^{HETETE}NC^{CAM}DK	831.81	2	3	13.20	2.00	1.009 – 1.109	40.40
6	TC^{CAM}VADE^{HETETE}SAENC^{CAM}DK	831.81	2	3	13.80	2.00	1.015 – 1.115	40.70

Table S13 continued

	Peptide	Precursor [m/z]	Charge	Δm/z	RT [min]	RT Range [min]	IM Range [V s cm^{-2}]	CE^{a} [eV]
7	QEPERNEC^{CAM}FLQH^{HETETE}K	626.95	3	3	14.60	2.00	0.855 – 0.955	34.40
8	AAFTEC^{CAM}C^{CAM}QAAD^{HETETE}K	768.30	2	3	15.40	2.00	1.000 – 1.100	35.00
9	LD^{HETETE}ELRDEGK	619.79	2	3	15.60	2.00	0.893 – 0.993	38.40
10	QEPERNEC^{CAM}FLQHKDD^{HETETE}NPNLPR	560.86	5	3	16.10	2.00	0.836 – 0.936	26.10
11	LKEC^{CAM}C^{CAM}E^{HETETE}KPLLEK	570.95	3	3	16.20	2.00	0.832 – 0.932	31.00
12	YIC^{CAM}ENQD^{HETETE}SISSKLK	616.96	3	3	16.70	2.00	0.873 – 0.973	27.60
13	TYE^{HETETE}TTLEK	574.76	2	3	17.00	2.00	0.879 – 0.979	27.80
14	AAFTE^{HETETE}C^{CAM}C^{CAM}QAADK	768.30	2	3	17.00	2.00	1.000 – 1.100	37.50
15	YIC^{CAM}E^{HETETE}NQDSISSK	804.34	2	3	17.00	2.00	0.995 – 1.095	39.90
16	NE^{HETETE}C^{CAM}FLQHK	620.27	2	3	17.20	2.00	0.882 – 0.982	33.00
17	C^{CAM}C^{CAM}TE^{HETETE}SLVNR	651.77	2	3	17.30	2.00	0.923 – 1.023	37.00
18	TC^{CAM}VADESAENC^{HETETE}DKSLHTLFGDK	868.71	3	3	17.50	2.00	1.003 – 1.103	42.70
19	SLH^{HETETE}TLFGDK	591.29	2	3	19.70	2.00	0.900 – 1.000	36.20
20	AAC^{CAM}LLPKLD^{HETETE}ELRDEGK	664.67	3	3	21.40	2.00	0.872 – 0.972	35.10
21	KVPQVSTPTLVE^{HETETE}VSR	902.49	2	3	21.60	2.00	1.115 – 1.215	47.00
22	LVNEVTE^{HETETE}FAK	657.33	2	3	21.80	2.00	0.932 – 1.032	29.90
23	LVRPEVDVMC^{CAM}TAFHD^{HETETE}NEETFLKK	736.35	4	3	22.40	2.00	0.922 – 1.022	32.00
24	LVRPEVDVMC^{CAM}TAFH^{HETETE}DNEETFLKK	736.35	4	3	23.10	2.00	0.935 – 1.035	35.00
25	RH^{HETETE}PDYSVVLLLR	544.63	3	3	23.50	2.00	0.861 – 0.961	29.60
26	LVRPEVDVMC^{CAM}TAFHDNEE^{HETETE}TFLKK	736.35	4	3	23.90	2.00	0.937 – 1.037	32.60
27	LVRPEVDVMC^{CAM}TAFHDNE^{HETETE}ETFLKK	589.28	5	3	23.90	2.00	0.856 – 0.956	29.40
28	RPC^{CAM}FSALEVDE^{HETETE}TYVPK	692.33	3	3	24.00	2.00	0.930 – 1.030	29.80
29	EFNAETFTFH^{HETETE}ADIC^{CAM}TLSEKER	903.74	3	3	24.10	2.00	1.061 – 1.161	44.90
30	LVRPE^{HETETE}VDVMC^{CAM}TAFHDNEETFLKK	736.35	4	3	24.40	2.00	0.938 – 1.038	32.60
31	NYAEAKD^{HETETE}VFLGMFLYEYAR	616.79	4	3	24.40	2.00	0.871 – 0.971	32.50
32	LVRPEVDVMC^{CAM}TAFHD^{HETETE}NEETFLK	704.33	4	3	24.60	2.00	0.919 – 1.019	29.40
33	QNC^{CAM}ELFE^{HETETE}QLGEYK	911.40	2	3	24.60	2.00	1.092 – 1.192	43.60
34	LVRPEVDVMC^{CAM}TAFH^{HETETE}DNEETFLK	938.77	3	3	24.60	2.00	1.026 – 1.126	43.50
35	QNC^{CAM}E^{HETETE}LFEQLGEYK	911.40	2	3	25.00	2.00	1.111 – 1.211	44.40
36	SHC^{CAM}IAEVEND^{HETETE}EMPADLPSLAADFVESK	1046.80	3	3	27.20	2.00	1.027 – 1.127	38.60
37	SHC^{CAM}IAEVENDE^{HETETE}MPADLPSLAADFVESK	1046.80	3	3	27.50	2.00	1.023 – 1.123	43.50
38	D^{HETETE}VFLGMFLYEYAR	596.61	3	3	28.50	2.00	0.806 – 0.906	25.00

2.2 NMR Spectra

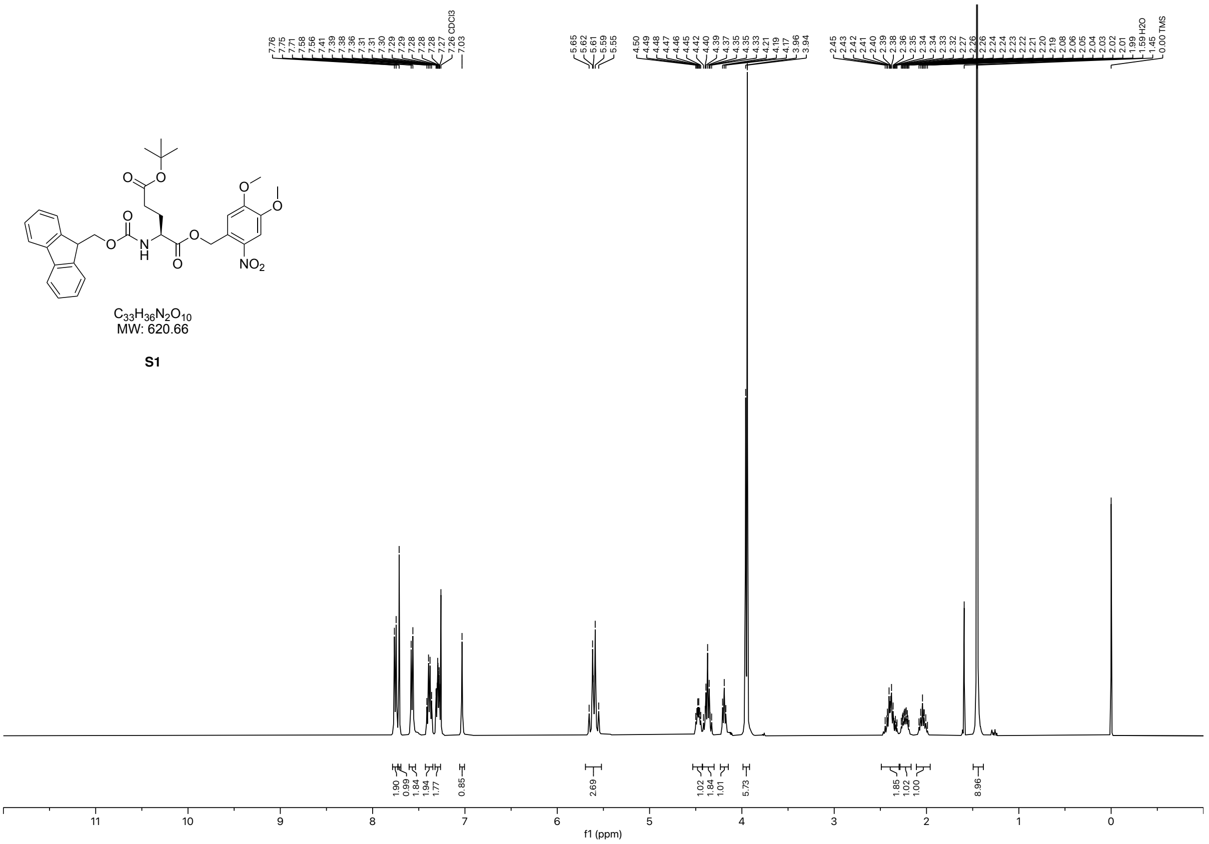

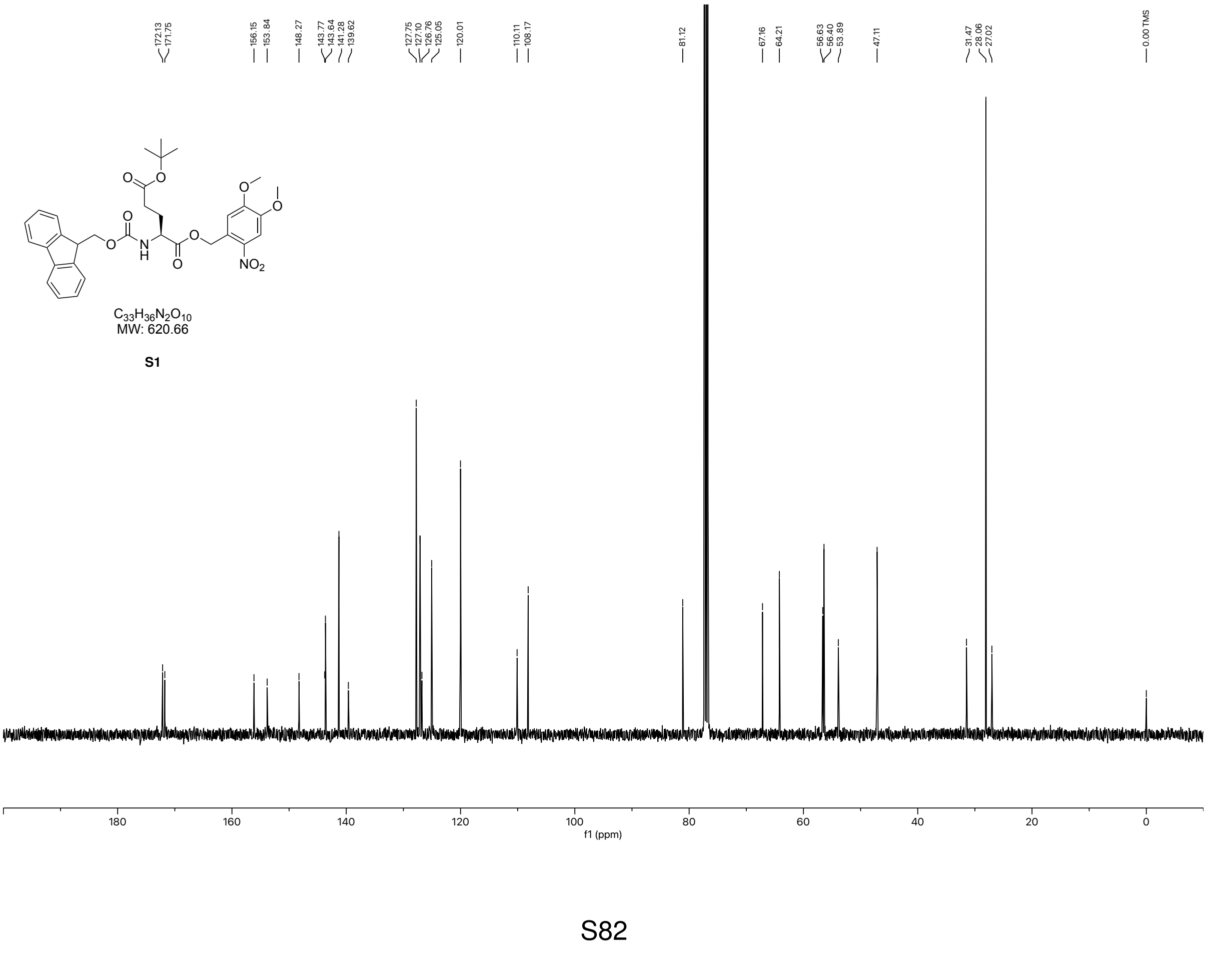

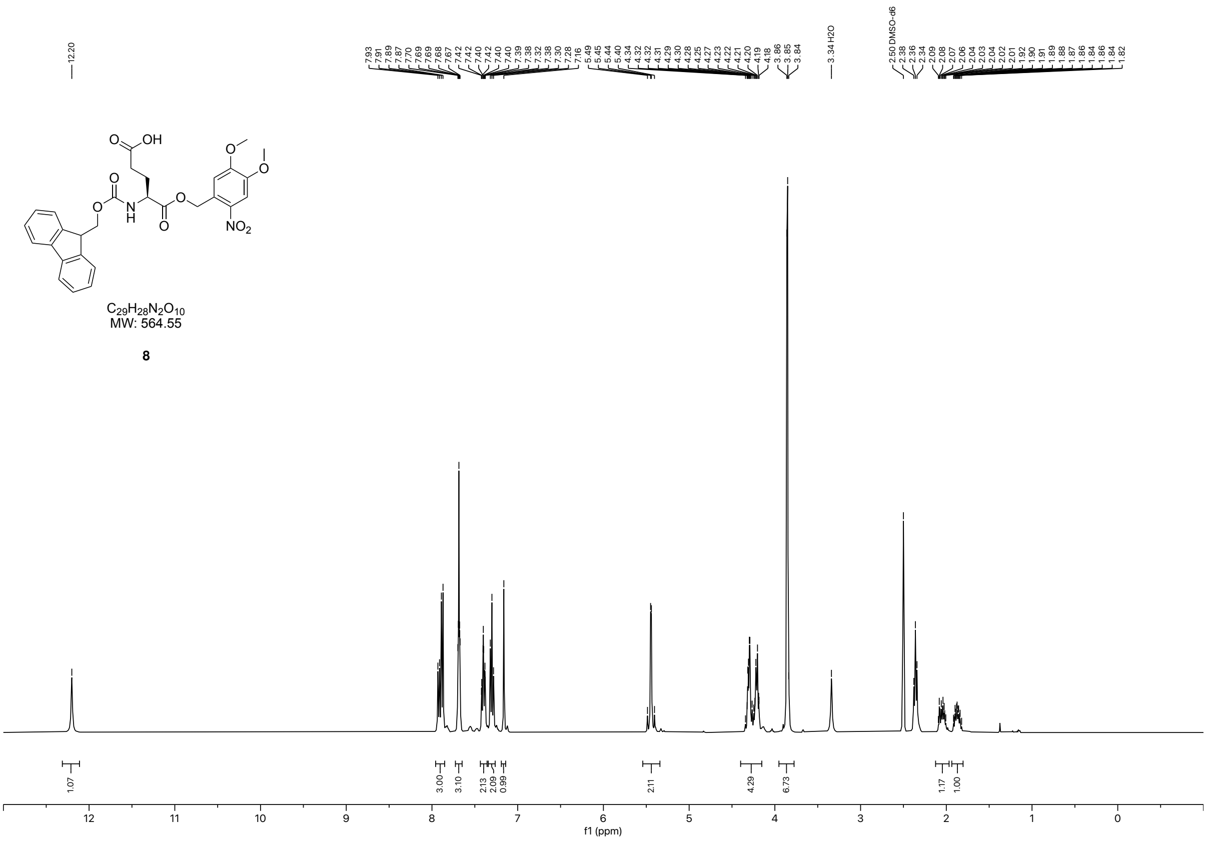
12.20
7.93 7.91 7.89 7.87 7.70 7.69 7.69 7.68 7.67 7.42 7.42 7.40 7.42 7.40 7.40 7.39 7.38 7.32 7.38 7.30 7.28 7.16
5.49 5.45 5.44 5.40 4.34 4.32 4.32 4.31 4.29 4.30 4.28 4.27 4.25 4.23 4.22 4.21 4.20 4.19 4.18 3.86 3.85 3.84
3.34 H2O
2.50 DMSO-d6
2.38 2.36 2.34 2.09 2.08 2.07 2.06 2.04 2.03 2.04 2.02 2.01 1.92 1.90 1.91 1.89 1.88 1.87 1.86 1.84 1.86 1.84 1.82
$C_{29}H_{28}N_2O_{10}$
MW: 564.55
8
1.07
3.00
3.10
2.13
2.09
0.99
2.11
4.29
6.73
1.17
1.00
f1 (ppm)

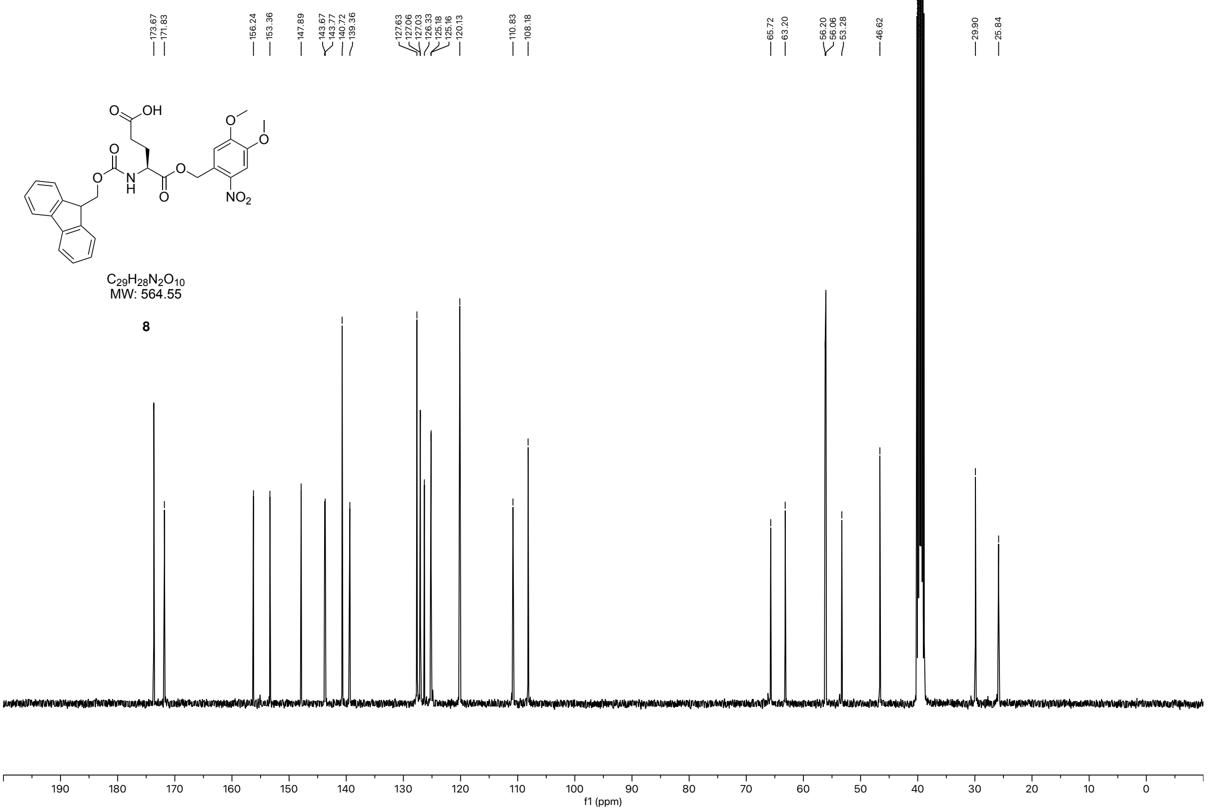
173.67 171.83
156.24 153.36
147.89
143.67 143.77 140.72 139.36
127.63 127.06 127.03 126.33 125.18 125.16 120.13
110.83 108.18
65.72 63.20
56.20 56.06 53.28
46.62
29.90 25.84
$C_{29}H_{28}N_2O_{10}$
MW: 564.55
8
f1 (ppm)

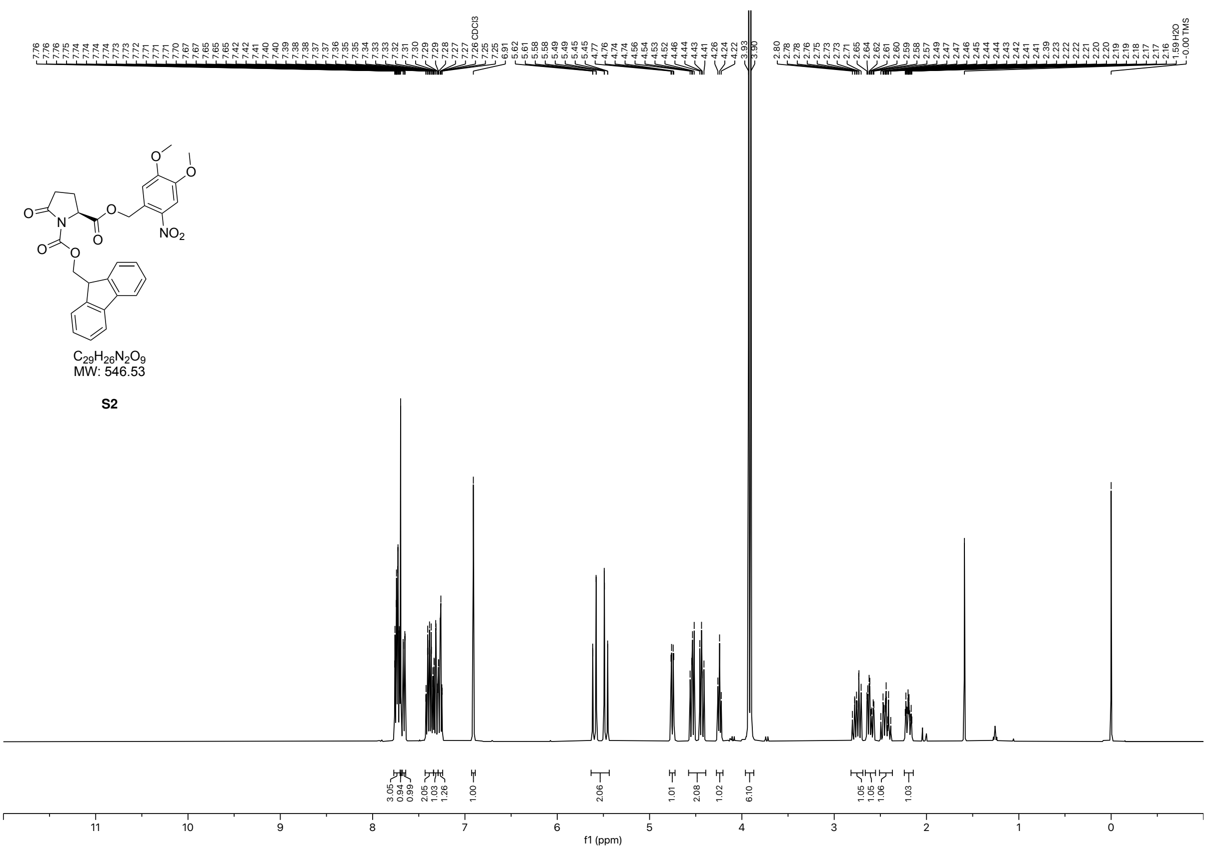
7.76
7.76
7.75
7.74
7.74
7.74
7.73
7.73
7.72
7.71
7.71
7.71
7.70
7.67
7.66
7.65
7.65
7.42
7.41
7.40
7.40
7.39
7.38
7.38
7.37
7.37
7.36
7.35
7.35
7.34
7.33
7.33
7.32
7.31
7.30
7.29
7.29
7.28
7.27
7.26 CDCl3
7.25
7.25
6.91
5.62
5.61
5.58
5.58
5.49
5.49
5.45
5.45
4.77
4.76
4.74
4.74
4.56
4.54
4.53
4.52
4.46
4.44
4.43
4.41
4.26
4.24
4.22
3.93
3.90
2.80
2.78
2.76
2.75
2.73
2.73
2.71
2.65
2.64
2.62
2.61
2.60
2.59
2.58
2.57
2.49
2.47
2.47
2.46
2.45
2.44
2.44
2.43
2.42
2.41
2.41
2.39
2.23
2.22
2.22
2.21
2.20
2.20
2.19
2.19
2.18
2.17
2.16
1.59 H2O
-0.00 TMS
C29H26N2O9
MW: 546.53
S2
3.05
0.94
0.99
2.05
1.03
1.26
1.00
2.06
1.01
2.08
1.02
6.10
1.05
1.05
1.06
1.03
f1 (ppm)

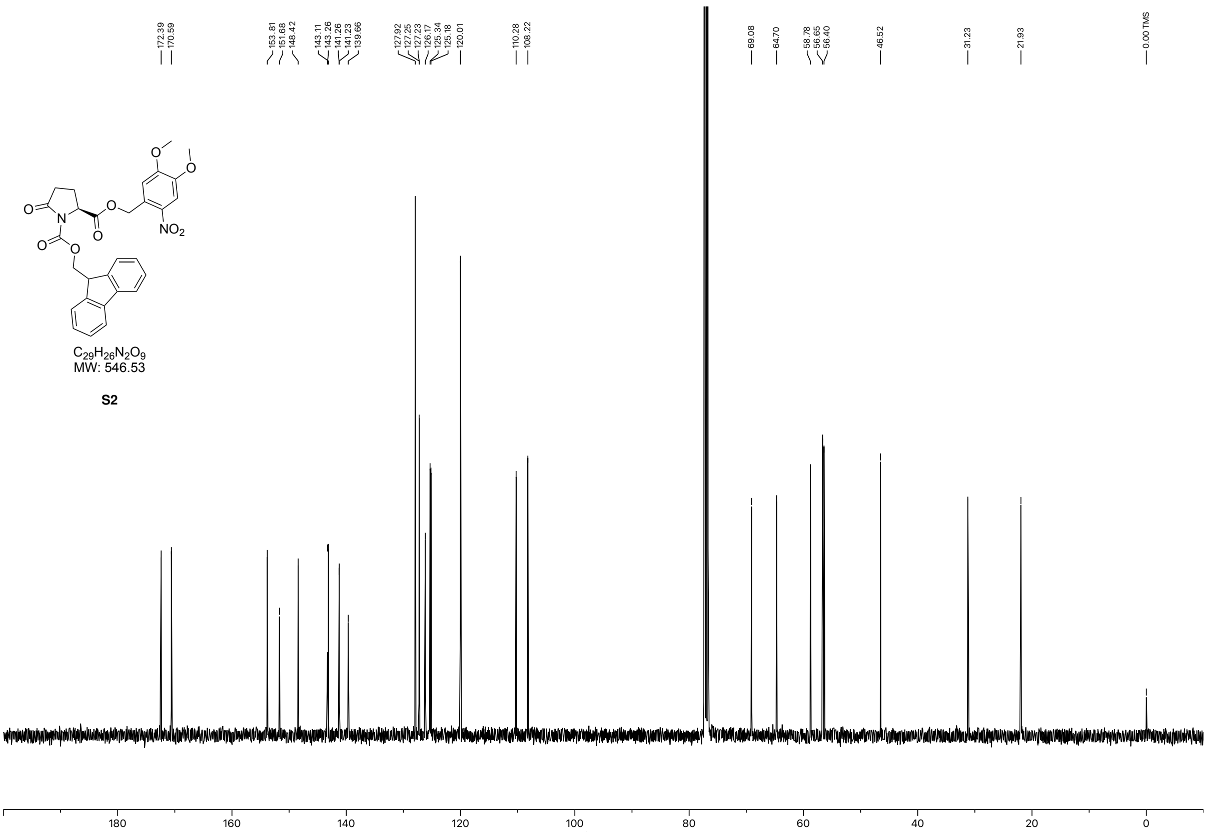
172.39
170.59
153.81
151.68
148.42
143.11
143.26
141.26
141.23
139.66
127.92
127.25
127.23
126.17
125.34
125.18
120.01
110.28
108.22
69.08
64.70
58.78
56.65
56.40
46.52
31.23
21.93
0.00 TMS
C29H26N2O9
MW: 546.53
S2
f1 (ppm)

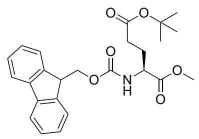

$C_{25}H_{29}NO_6$
MW: 439.51

S3

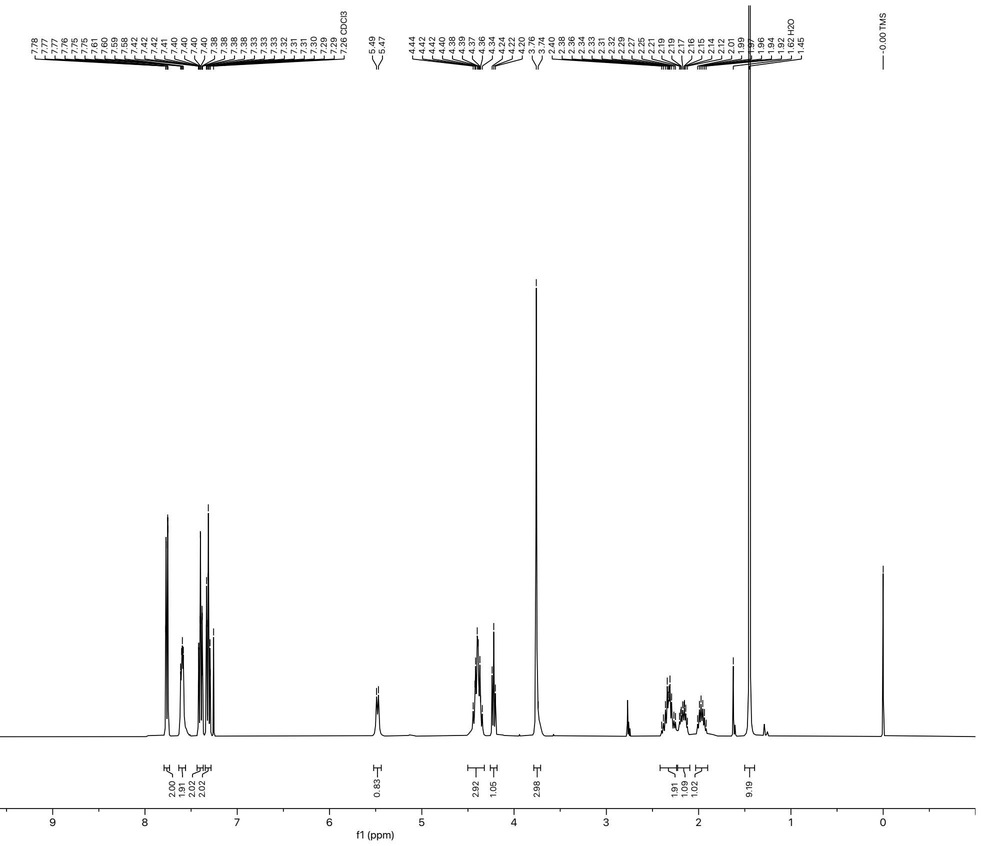

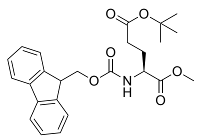

$C_{25}H_{29}NO_6$
MW: 439.51

S3

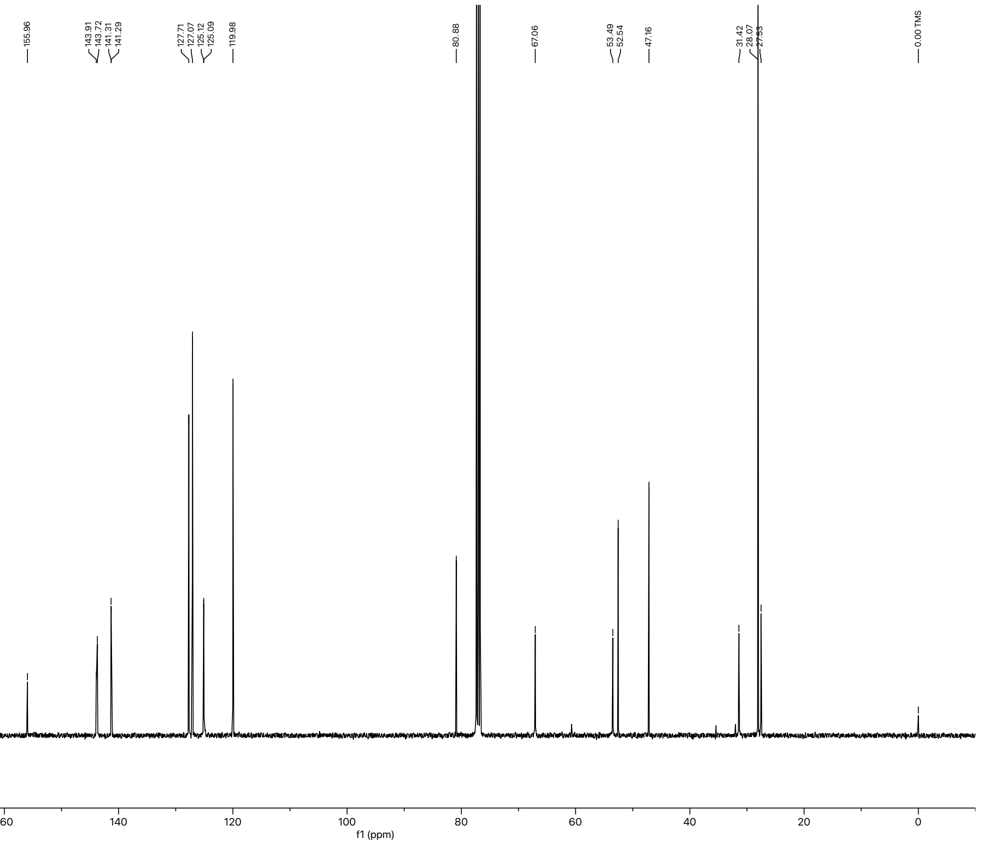

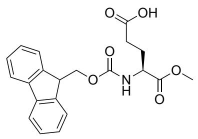

$C_{21}H_{21}NO_6$
MW: 383.40

S4

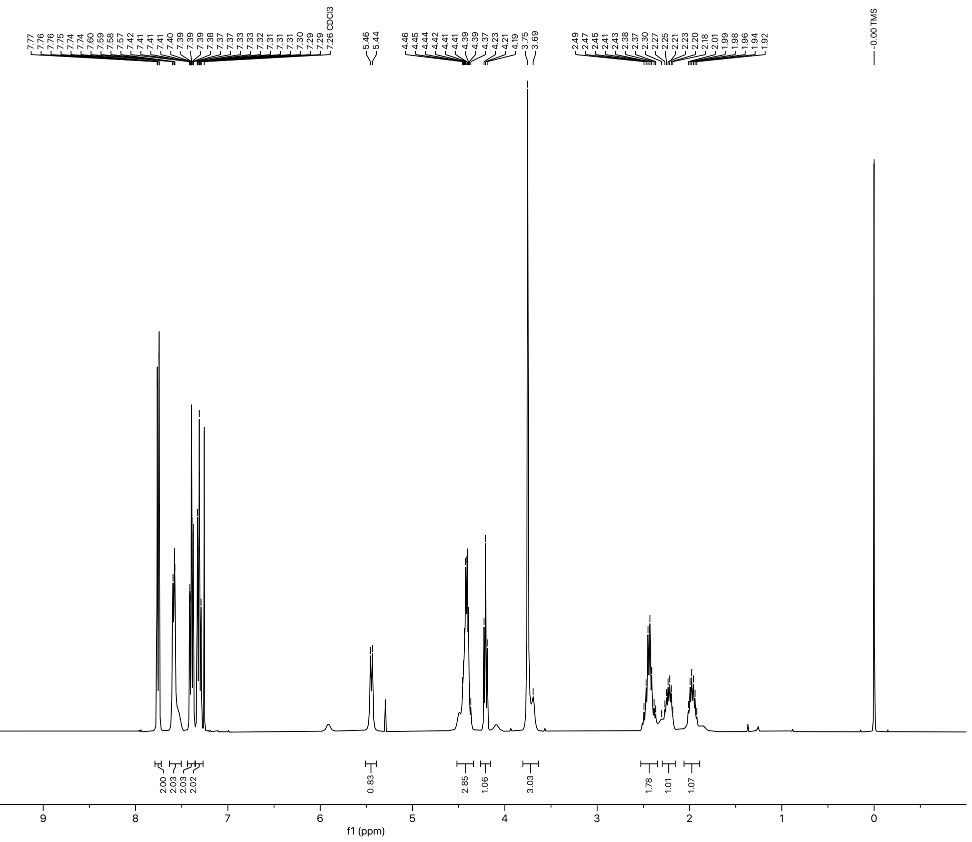

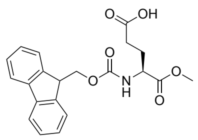

$C_{21}H_{21}NO_6$
MW: 383.40

S4

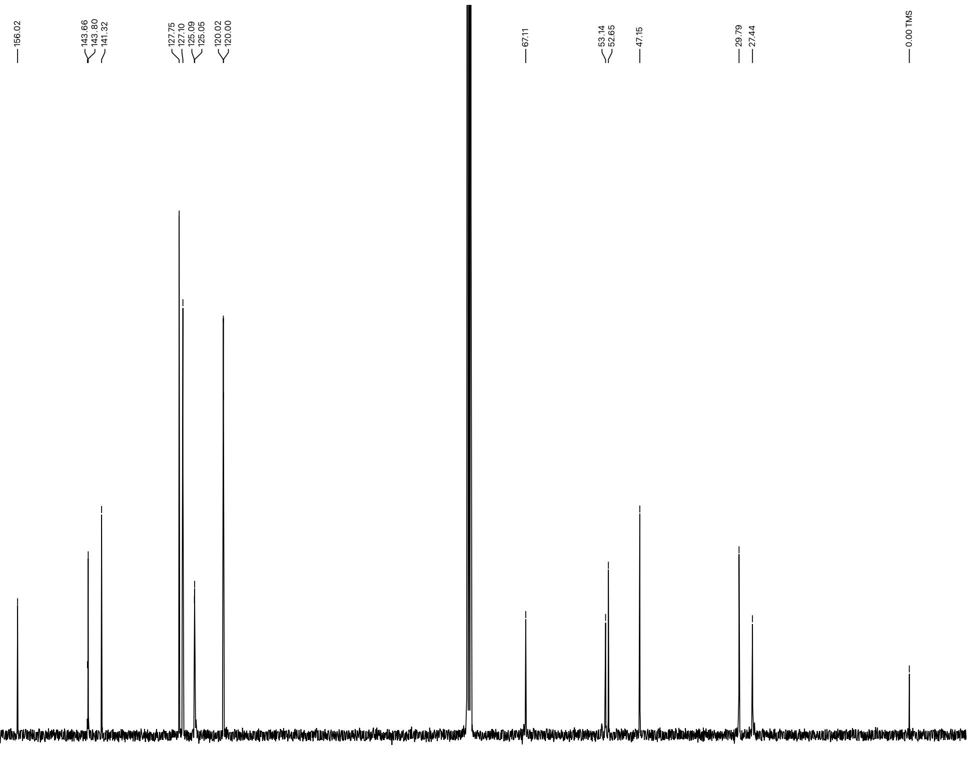

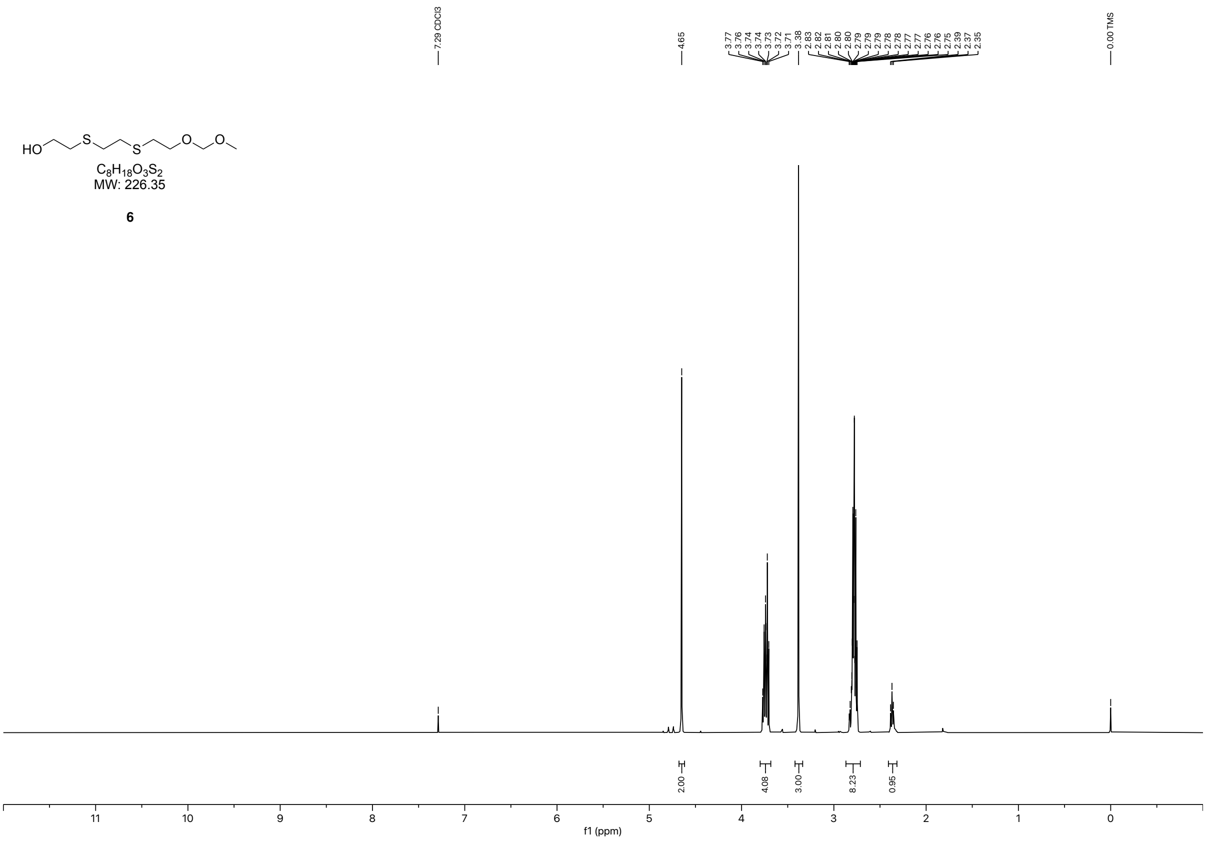

$C_8H_{18}O_3S_2$
MW: 226.35

6

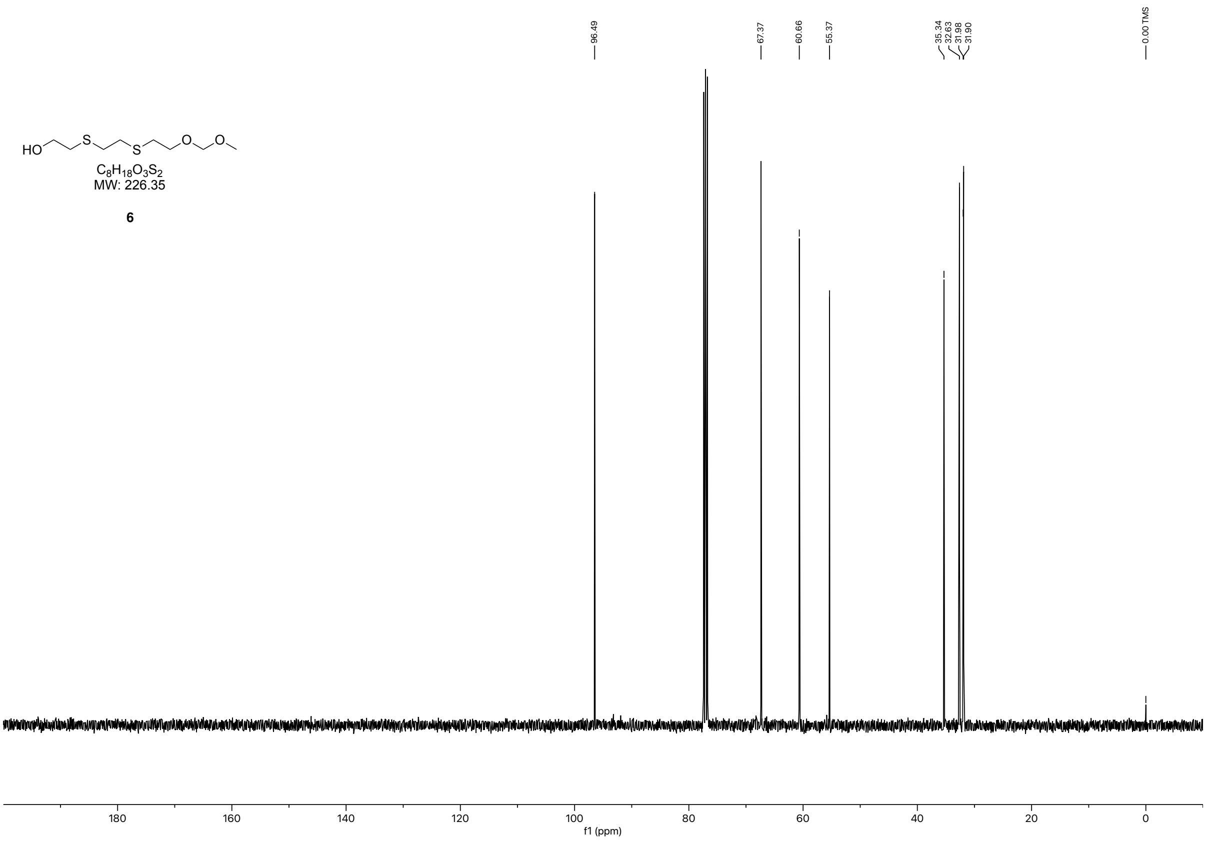

$C_8H_{18}O_3S_2$
MW: 226.35

6

$C_{10}H_{22}O_4S_2$
MW: 270.40

6'

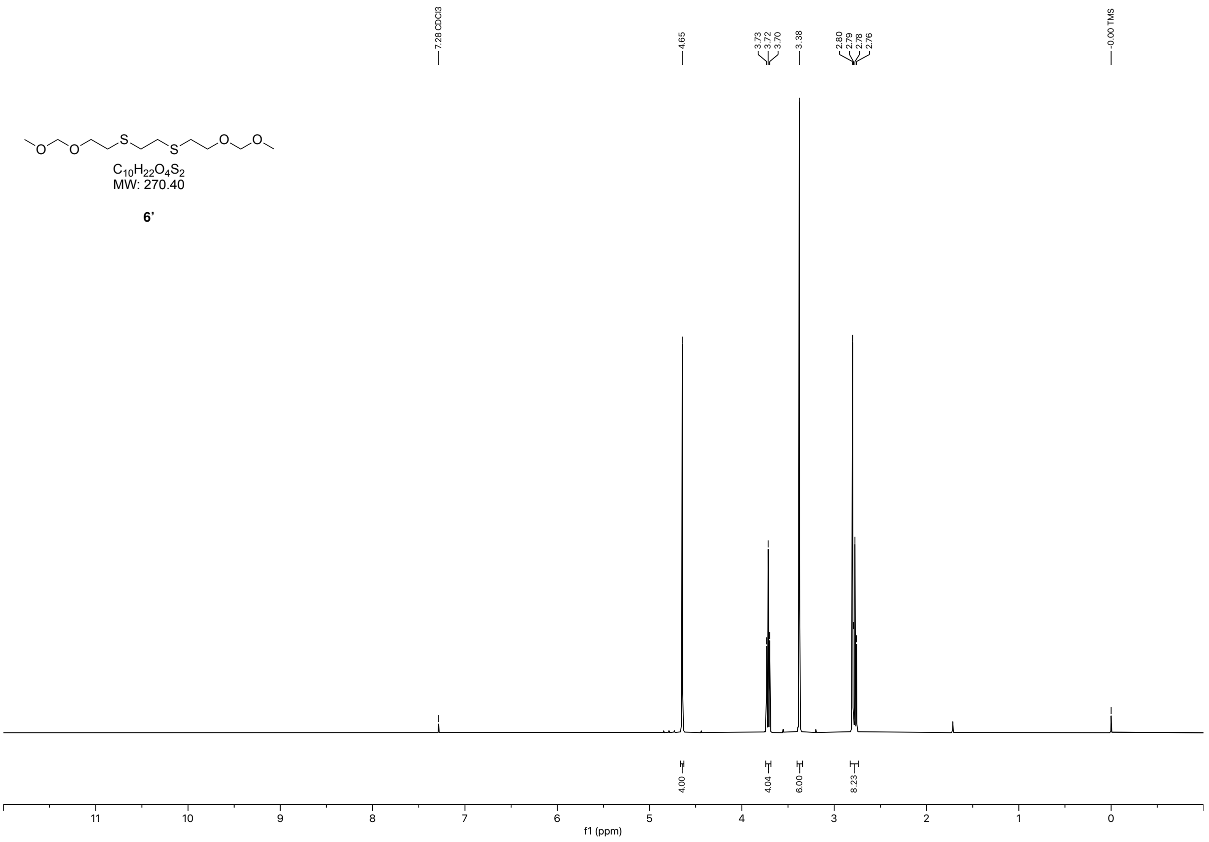

$C_{10}H_{22}O_4S_2$
MW: 270.40

6'

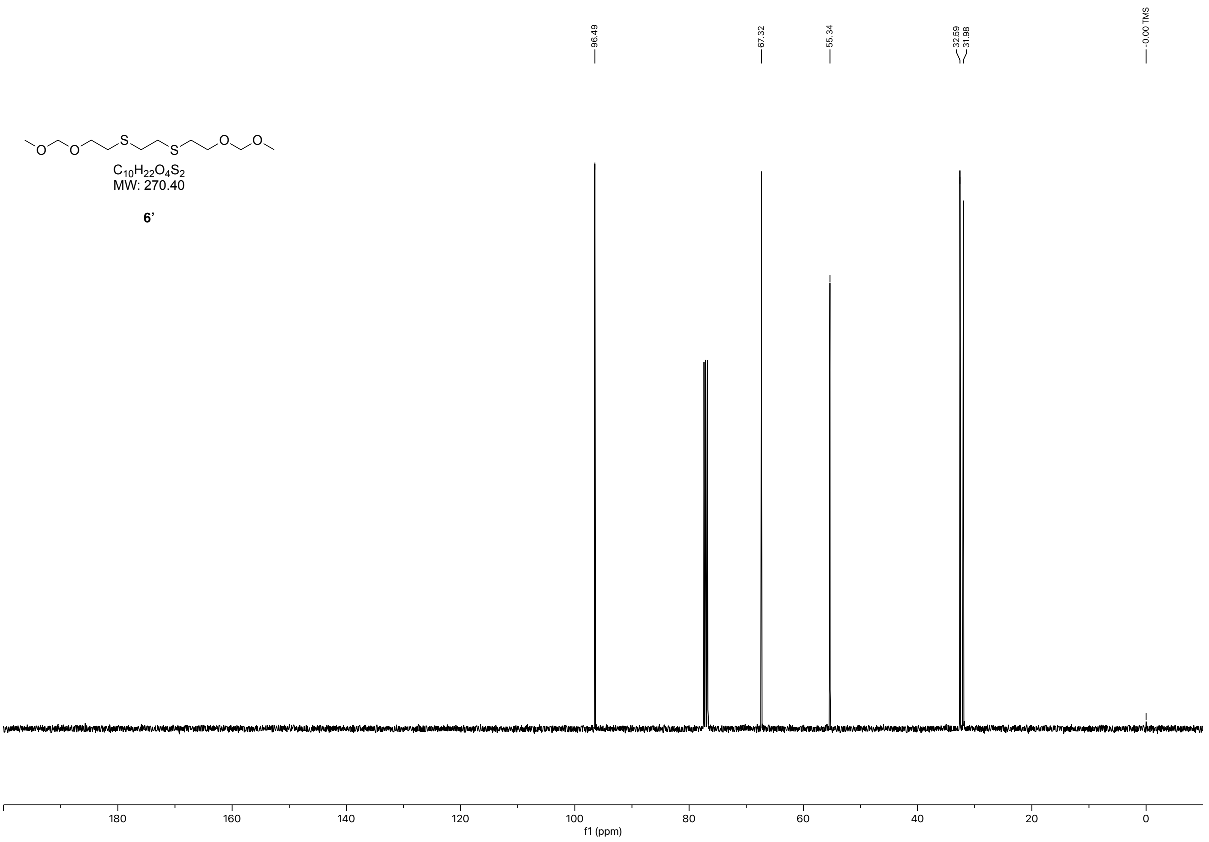

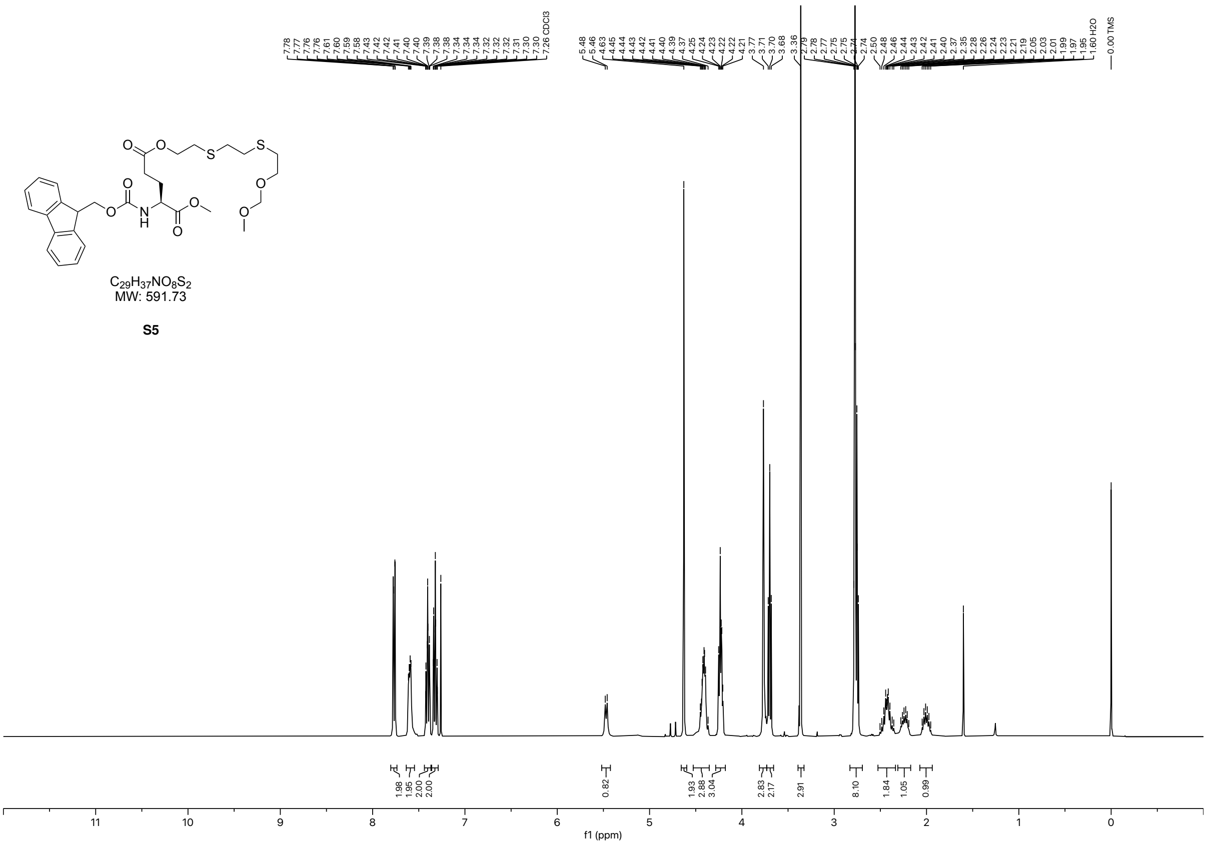
7.78
7.77
7.76
7.61
7.60
7.59
7.58
7.43
7.42
7.42
7.41
7.40
7.40
7.39
7.38
7.38
7.34
7.34
7.34
7.32
7.32
7.32
7.31
7.30
7.26 CDCl3
5.48
5.46
4.63
4.45
4.44
4.43
4.42
4.41
4.40
4.39
4.37
4.25
4.24
4.24
4.23
4.22
4.22
4.21
3.77
3.71
3.70
3.68
3.36
2.79
2.77
2.75
2.75
2.74
2.50
2.48
2.46
2.44
2.43
2.42
2.41
2.40
2.37
2.35
2.28
2.26
2.24
2.23
2.21
2.19
2.05
2.03
2.01
1.99
1.97
1.95
1.60 H2O
0.00 TMS
C29H37NO8S2
MW: 591.73
S5
1.98
1.95
2.00
2.00
0.82
1.93
2.88
3.04
2.83
2.17
2.91
8.10
1.84
1.05
0.99
11
10
9
8
7
6
5
4
3
2
1
0
f1 (ppm)

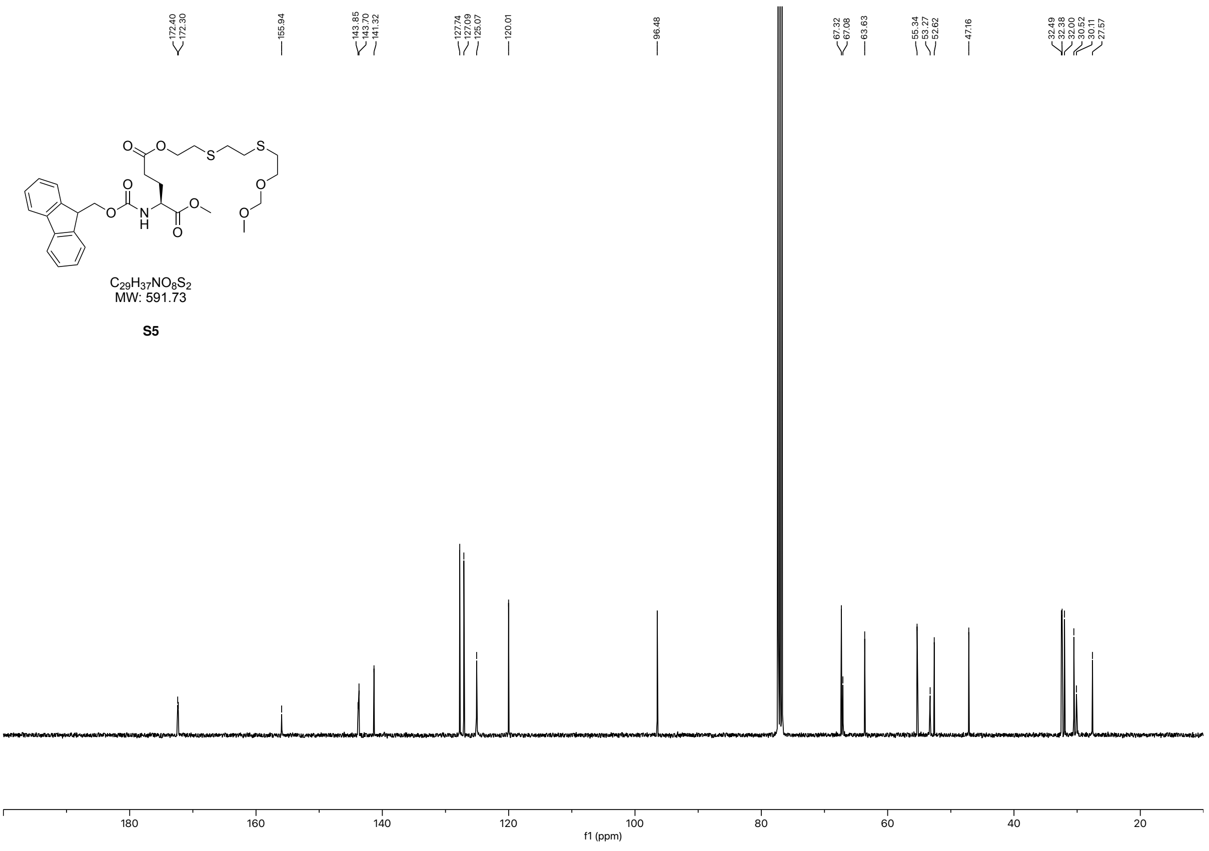
172.40
172.30
155.94
143.85
143.70
141.32
127.74
127.09
125.07
120.01
96.48
67.32
67.08
63.63
55.34
53.27
52.62
47.16
32.49
32.38
32.00
30.52
30.11
27.57
C29H37NO8S2
MW: 591.73
S5
180
160
140
120
100
80
60
40
20
f1 (ppm)

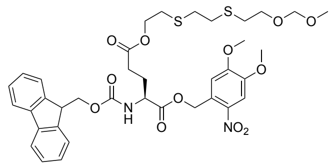

$C_{37}H_{44}N_{2}O_{12}S_{2}$
MW: 772.88

9

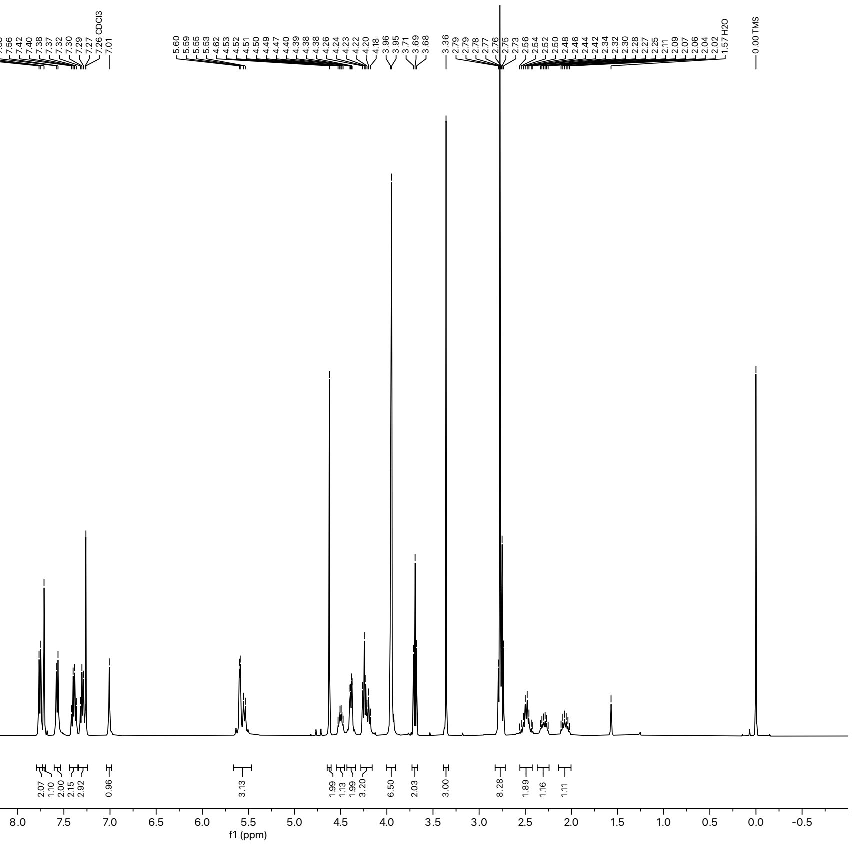

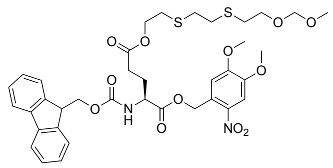

$C_{37}H_{44}N_{2}O_{12}S_{2}$
MW: 772.88

9

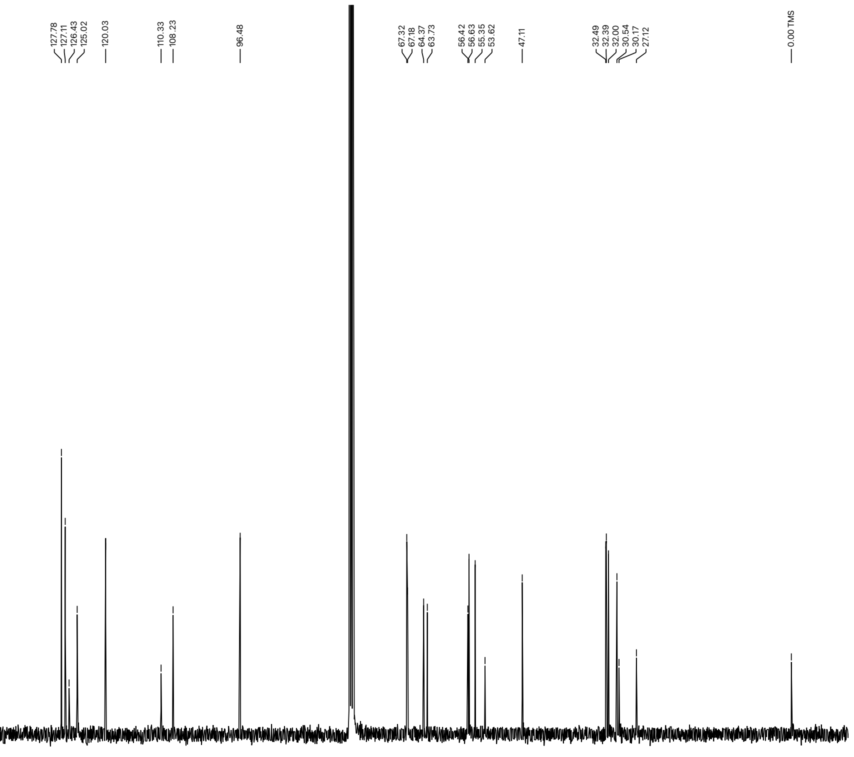

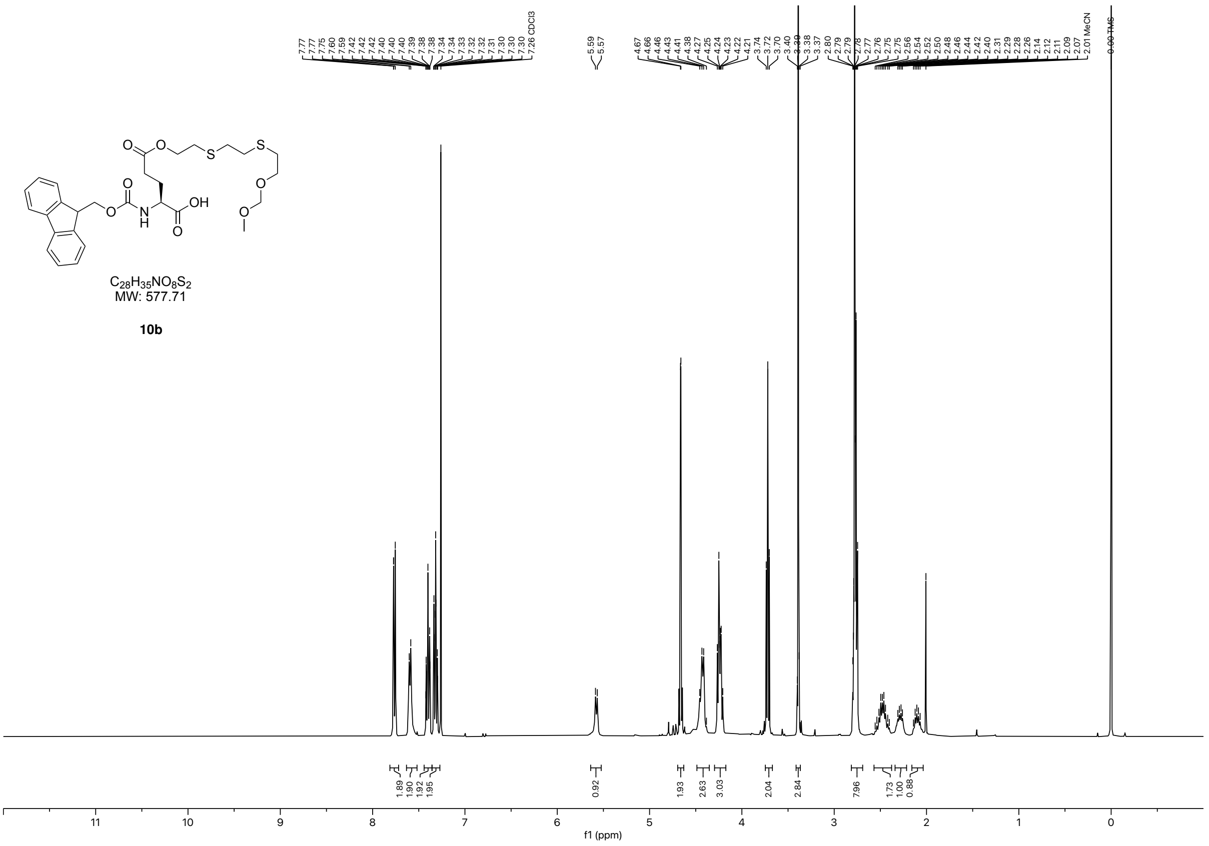
7.77
7.77
7.75
7.60
7.59
7.42
7.42
7.42
7.40
7.40
7.40
7.39
7.38
7.38
7.34
7.34
7.33
7.32
7.32
7.31
7.30
7.30
7.30
7.26 CDCl3
5.59
5.57
4.67
4.66
4.46
4.43
4.41
4.38
4.27
4.25
4.24
4.23
4.22
4.21
3.74
3.72
3.70
3.40
3.39
3.38
3.37
2.80
2.79
2.79
2.78
2.77
2.76
2.75
2.75
2.56
2.54
2.52
2.50
2.48
2.46
2.44
2.42
2.40
2.31
2.29
2.28
2.26
2.14
2.12
2.11
2.09
2.07
2.01 MeCN
0.00 TMS
O
O
S
S
O
O
O
N
H
OH
O
O
O
$C_{28}H_{35}NO_8S_2$
MW: 577.71
10b
1.89
1.90
1.92
1.95
0.92
1.93
2.63
3.03
2.04
2.84
7.96
1.73
1.00
0.88
11
10
9
8
7
6
5
4
3
2
1
0
f1 (ppm)

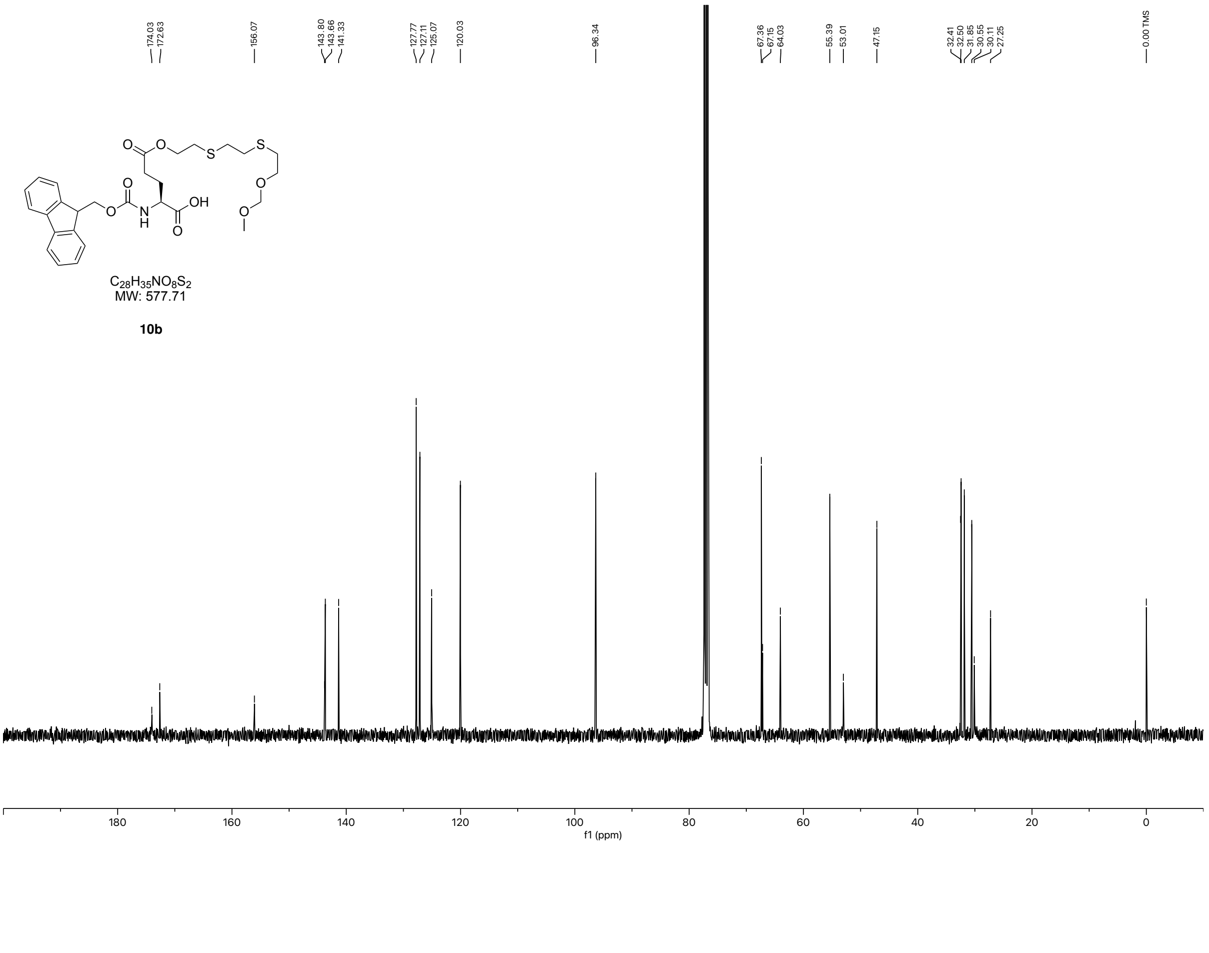
174.03
172.63
156.07
143.80
143.66
141.33
127.77
127.11
125.07
120.03
96.34
67.36
67.15
64.03
55.39
53.01
47.15
32.41
32.50
31.85
30.55
30.11
27.26
0.00 TMS
$C_{28}H_{35}NO_8S_2$
MW: 577.71
10b
180
160
140
120
100
80
60
40
20
0
f1 (ppm)

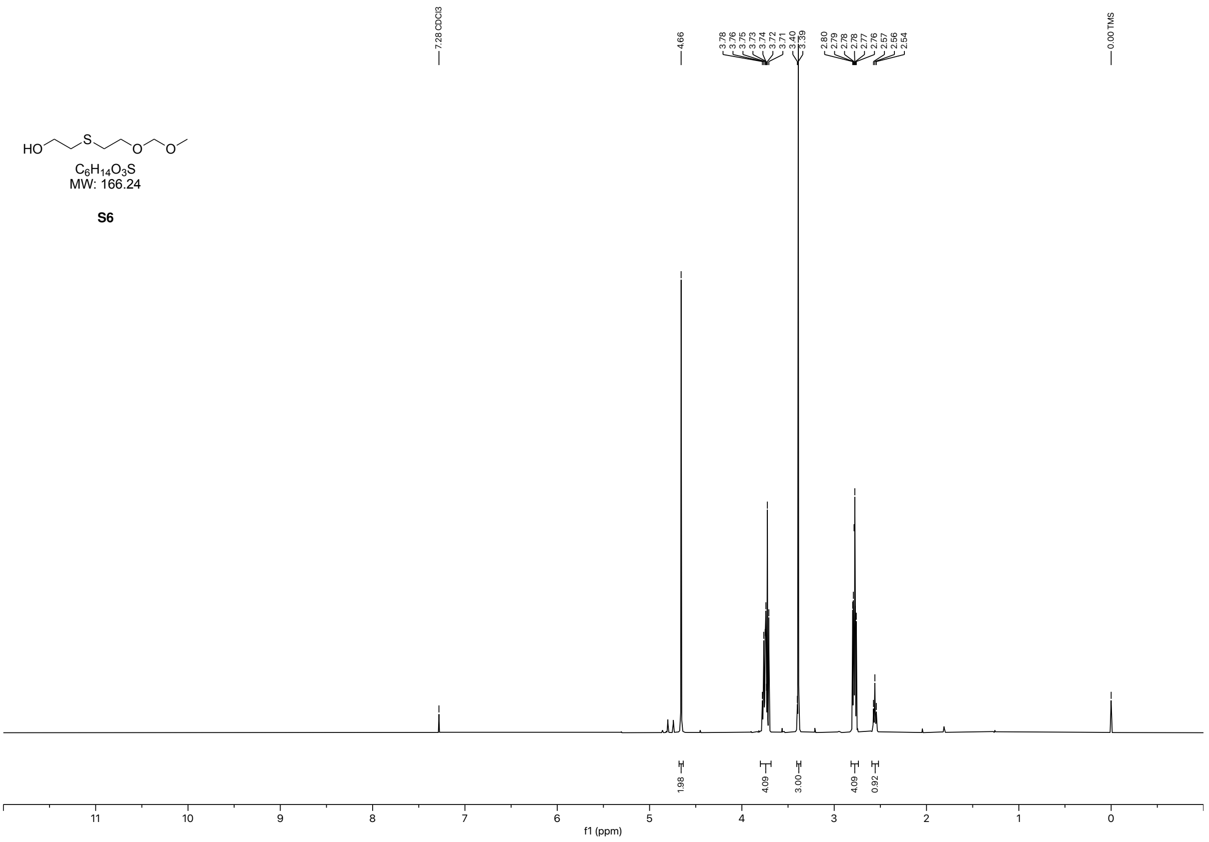
7.28 CDCl3
4.66
3.78
3.76
3.75
3.73
3.74
3.72
3.71
3.40
3.39
2.80
2.79
2.78
2.78
2.77
2.76
2.57
2.56
2.54
0.00 TMS
HO
S
O
O
$C_6H_{14}O_3S$
MW: 166.24
S6
1.98
4.09
3.00
4.09
0.92
11
10
9
8
7
6
5
4
3
2
1
0
f1 (ppm)

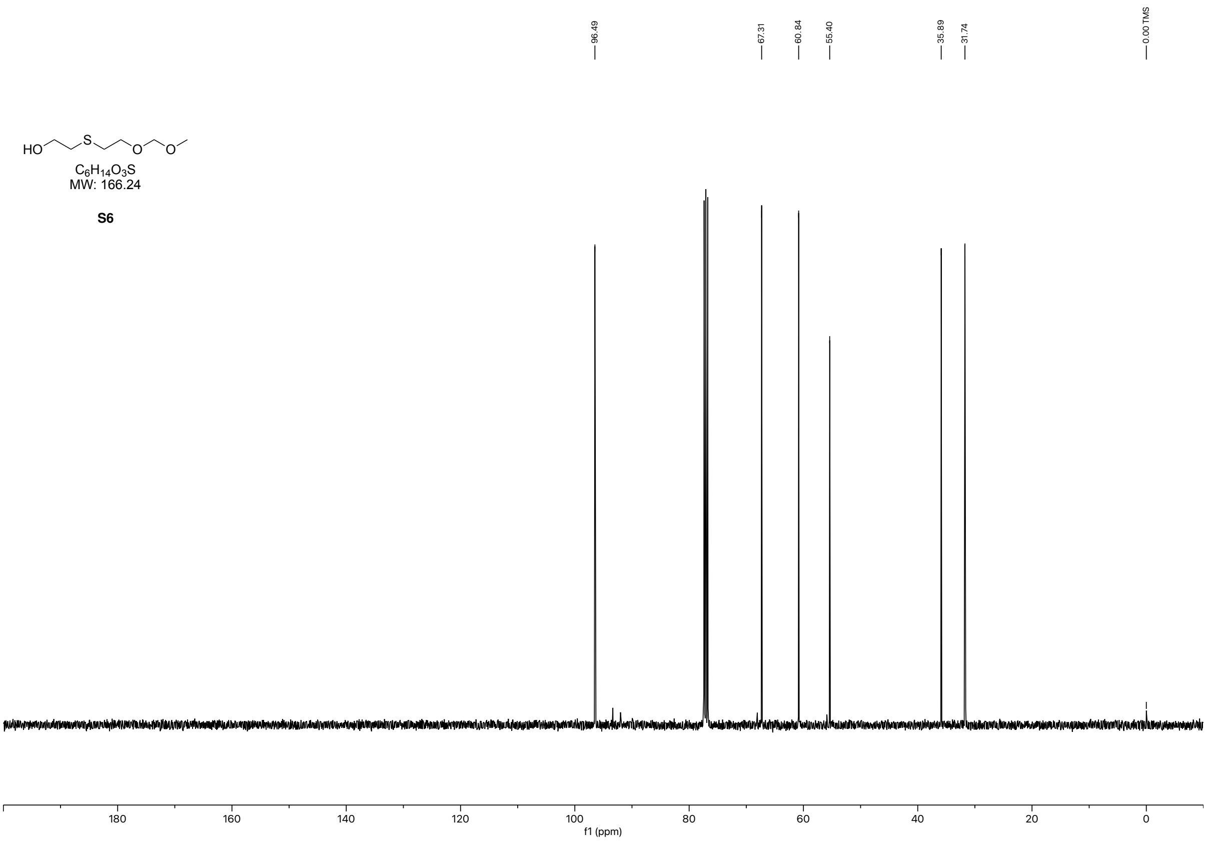
96.49
67.31
60.84
55.40
35.89
31.74
0.00 TMS
HO
S
O
O
$C_6H_{14}O_3S$
MW: 166.24
S6
180
160
140
120
100
80
60
40
20
0
f1 (ppm)

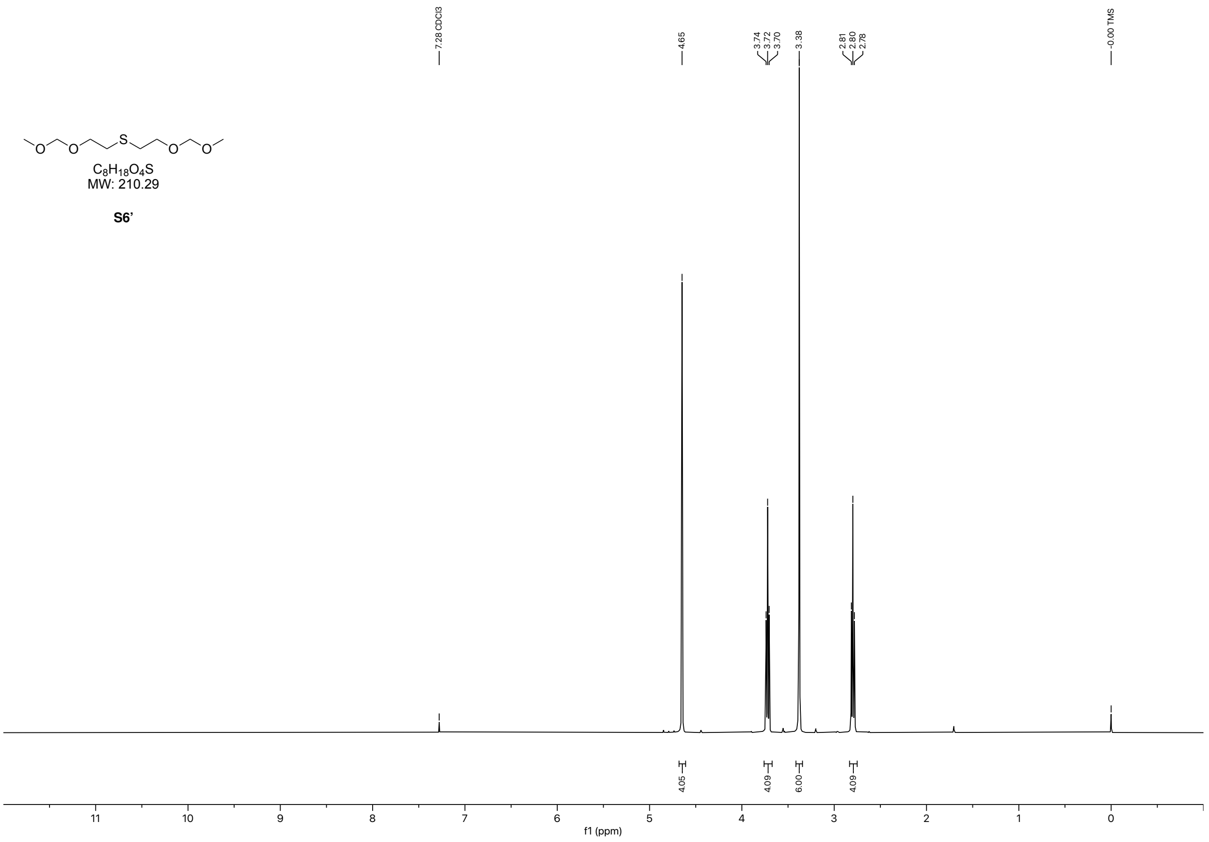
7.28 CDCl3
4.65
3.74
3.72
3.70
3.38
2.81
2.80
2.78
0.00 TMS
$C_8H_{18}O_4S$
MW: 210.29
S6'
4.05
4.09
6.00
4.09
f1 (ppm)

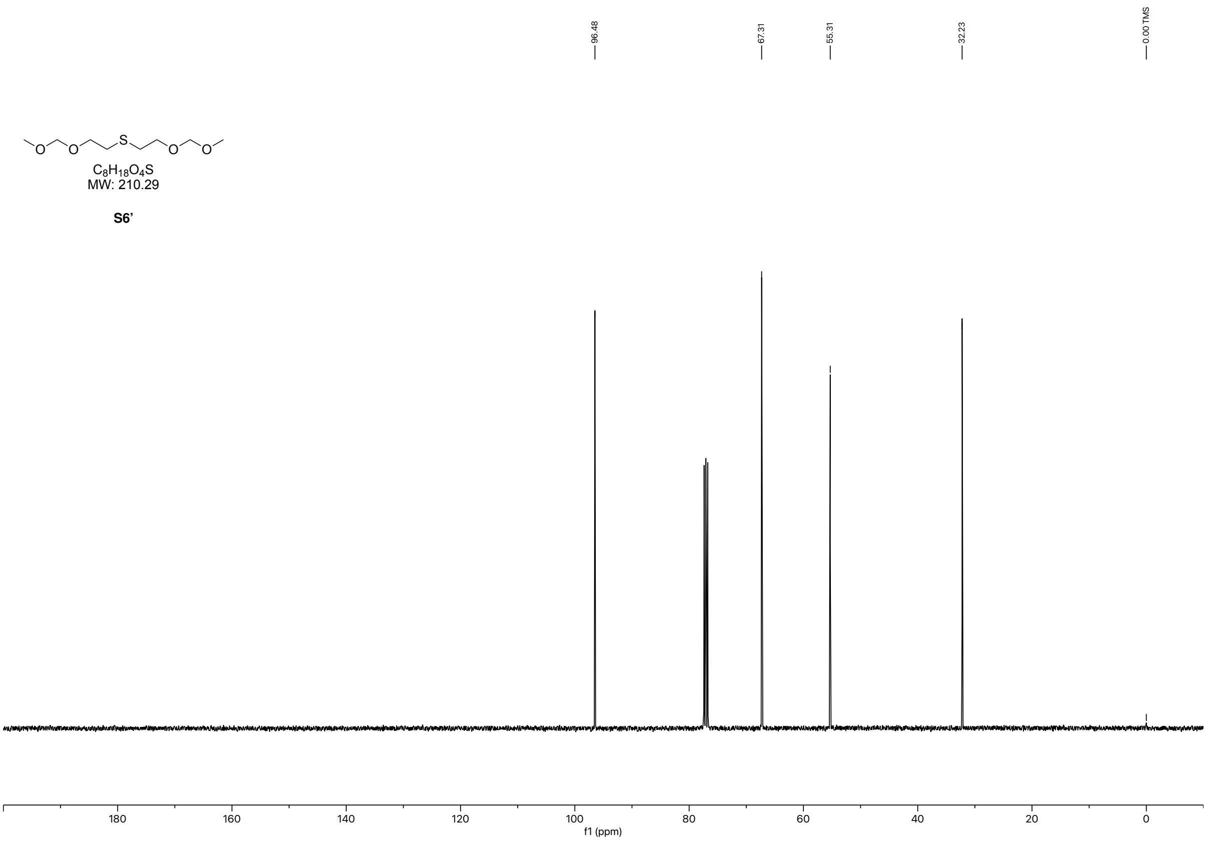
96.48
67.31
55.31
32.23
0.00 TMS
$C_8H_{18}O_4S$
MW: 210.29
S6'
f1 (ppm)

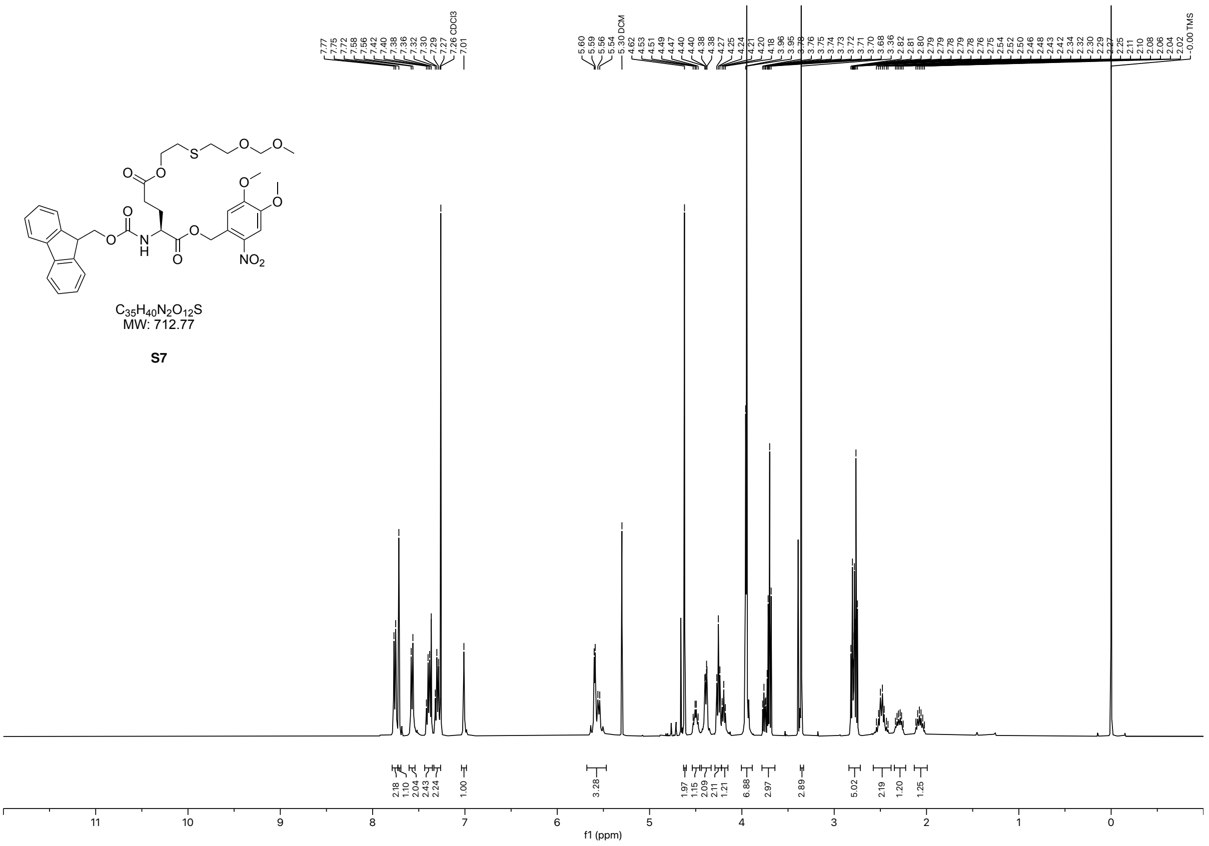
C35H40N2O12S
MW: 712.77
S7
f1 (ppm)

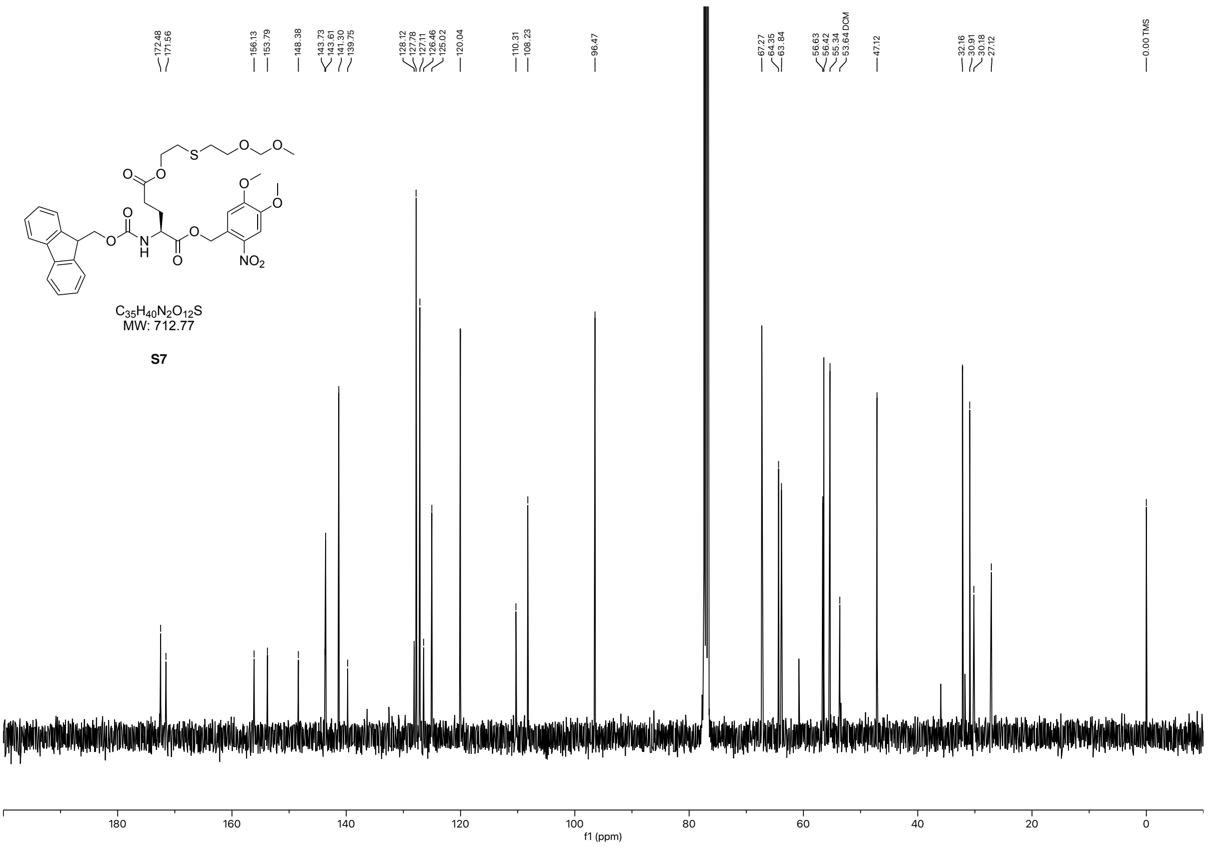
C35H40N2O12S
MW: 712.77
S7
f1 (ppm)

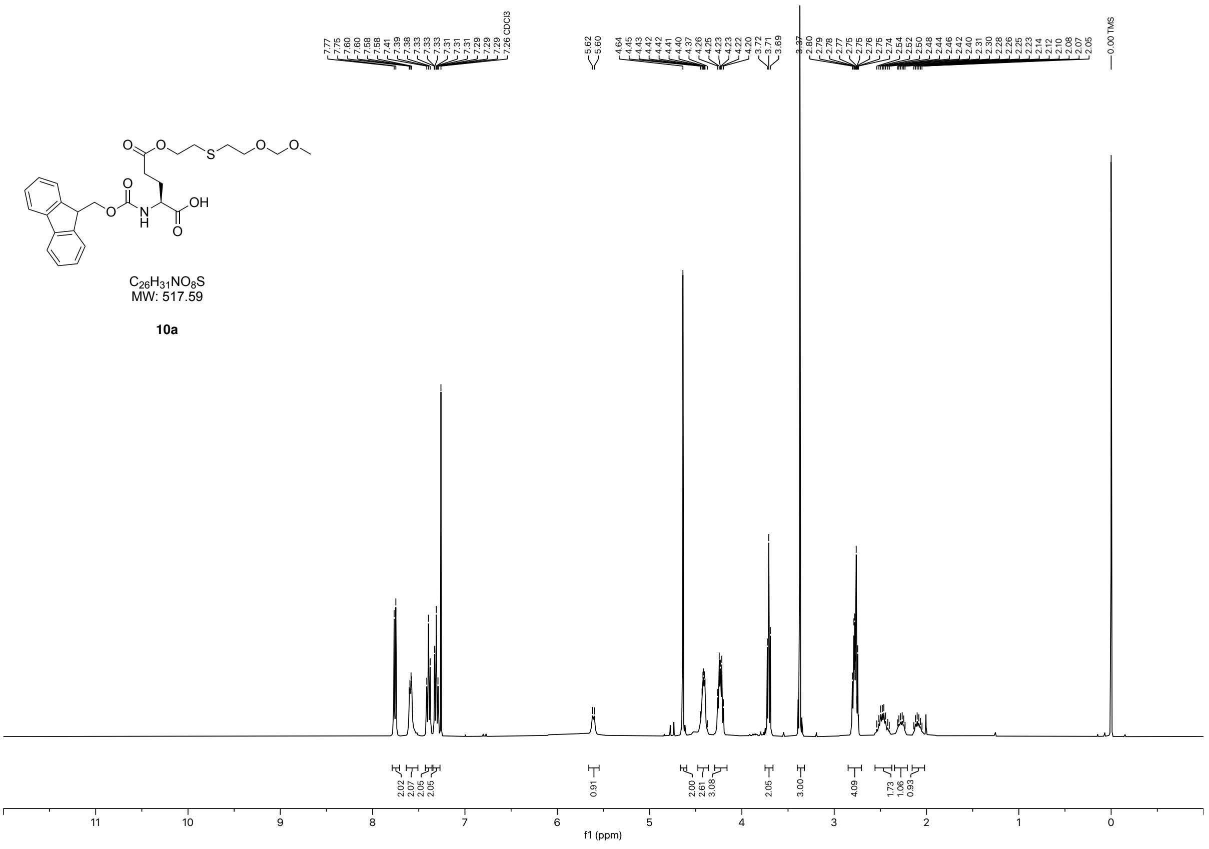
$C_{26}H_{31}NO_8S$
MW: 517.59
10a
CDCl3
0.00 TMS
f1 (ppm)

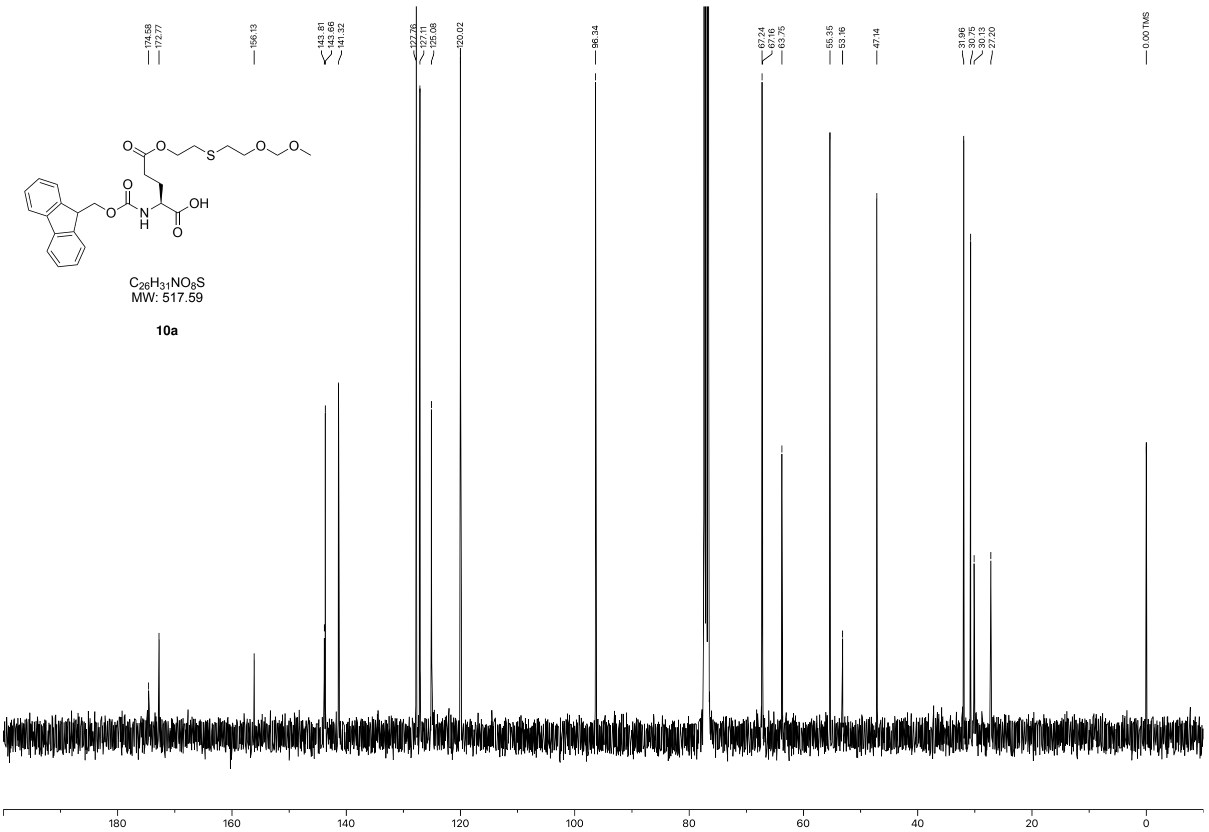
$C_{26}H_{31}NO_8S$
MW: 517.59
10a
0.00 TMS
f1 (ppm)

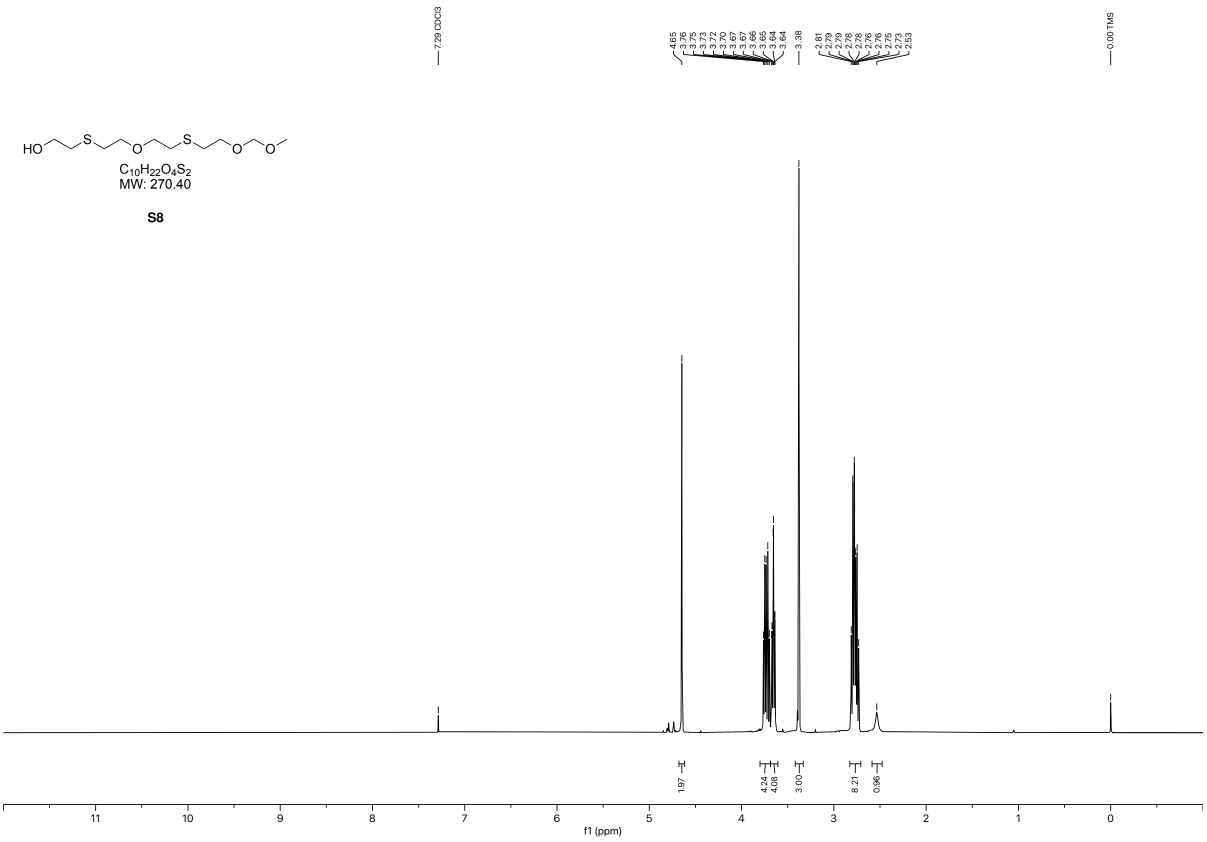
HO S O S O O
$C_{10}H_{22}O_4S_2$
MW: 270.40
S8
7.29 CDCl3
4.65
3.76
3.75
3.73
3.72
3.70
3.67
3.67
3.66
3.65
3.64
3.64
3.38
2.81
2.79
2.79
2.78
2.78
2.76
2.76
2.75
2.73
2.53
0.00 TMS
1.97
4.24
4.08
3.00
8.21
0.96
11
10
9
8
7
6
5
4
3
2
1
0
f1 (ppm)

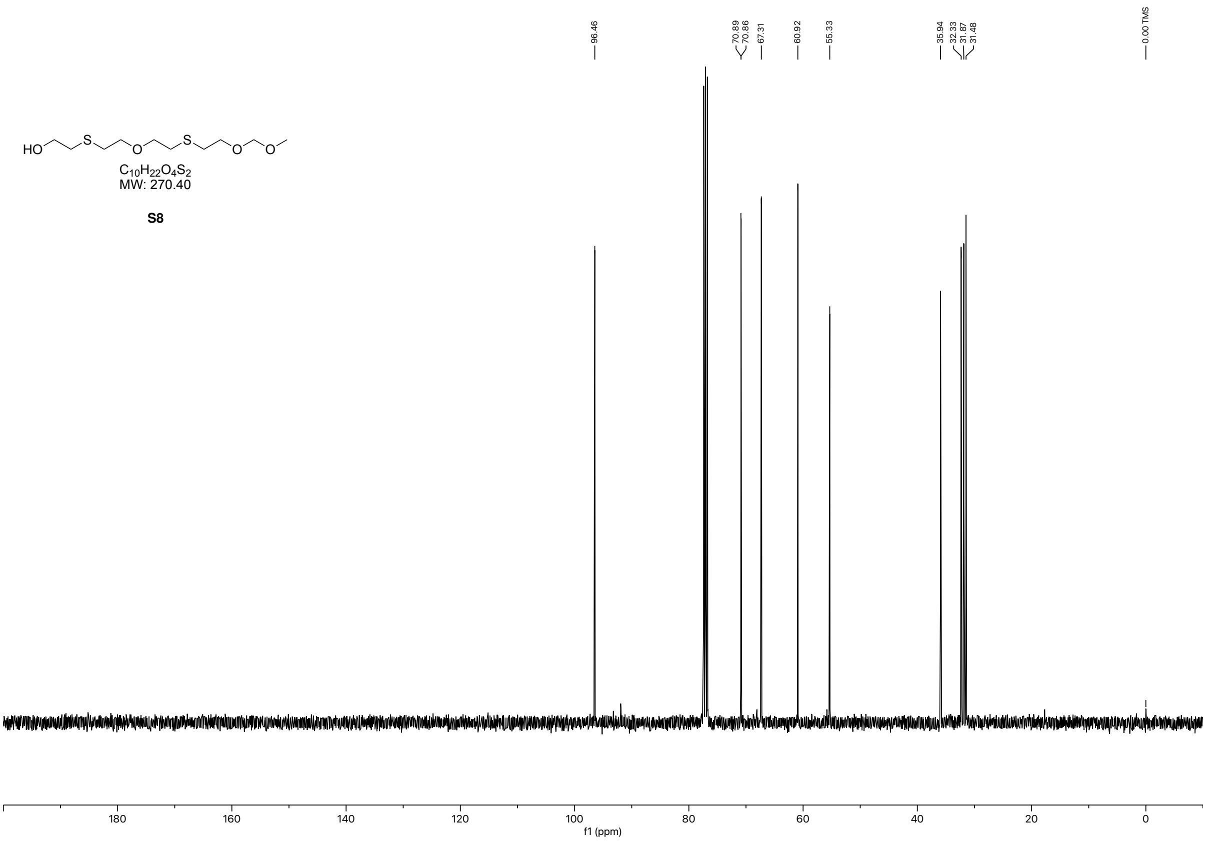
HO S O S O O
$C_{10}H_{22}O_4S_2$
MW: 270.40
S8
96.46
70.89
70.86
67.31
60.92
55.33
35.94
32.33
31.87
31.48
0.00 TMS
180
160
140
120
100
80
60
40
20
0
f1 (ppm)

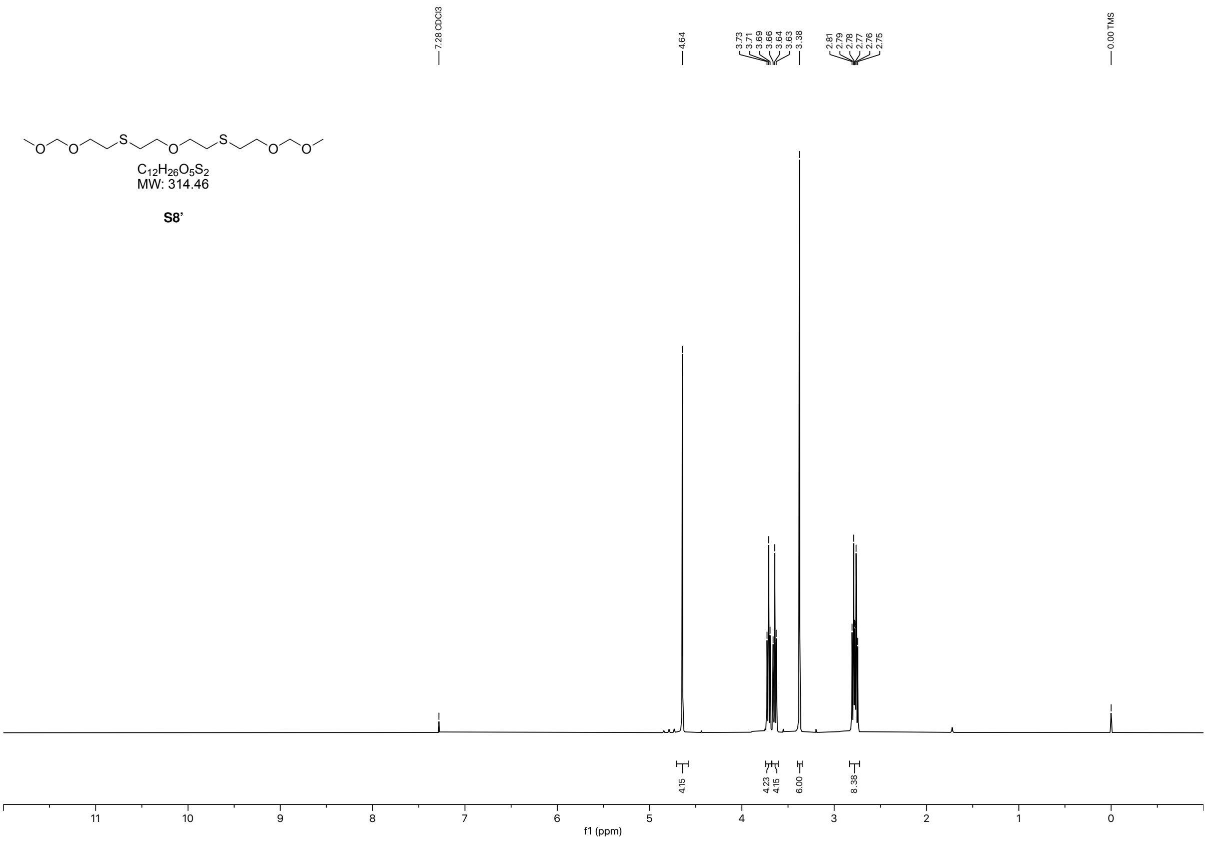
7.28 CDCl3
4.64
3.73
3.71
3.69
3.66
3.64
3.63
3.38
2.81
2.79
2.78
2.77
2.76
2.75
0.00 TMS
$C_{12}H_{26}O_5S_2$
MW: 314.46
S8'
4.15
4.23
4.15
6.00
8.38
11 10 9 8 7 6 5 4 3 2 1 0
f1 (ppm)

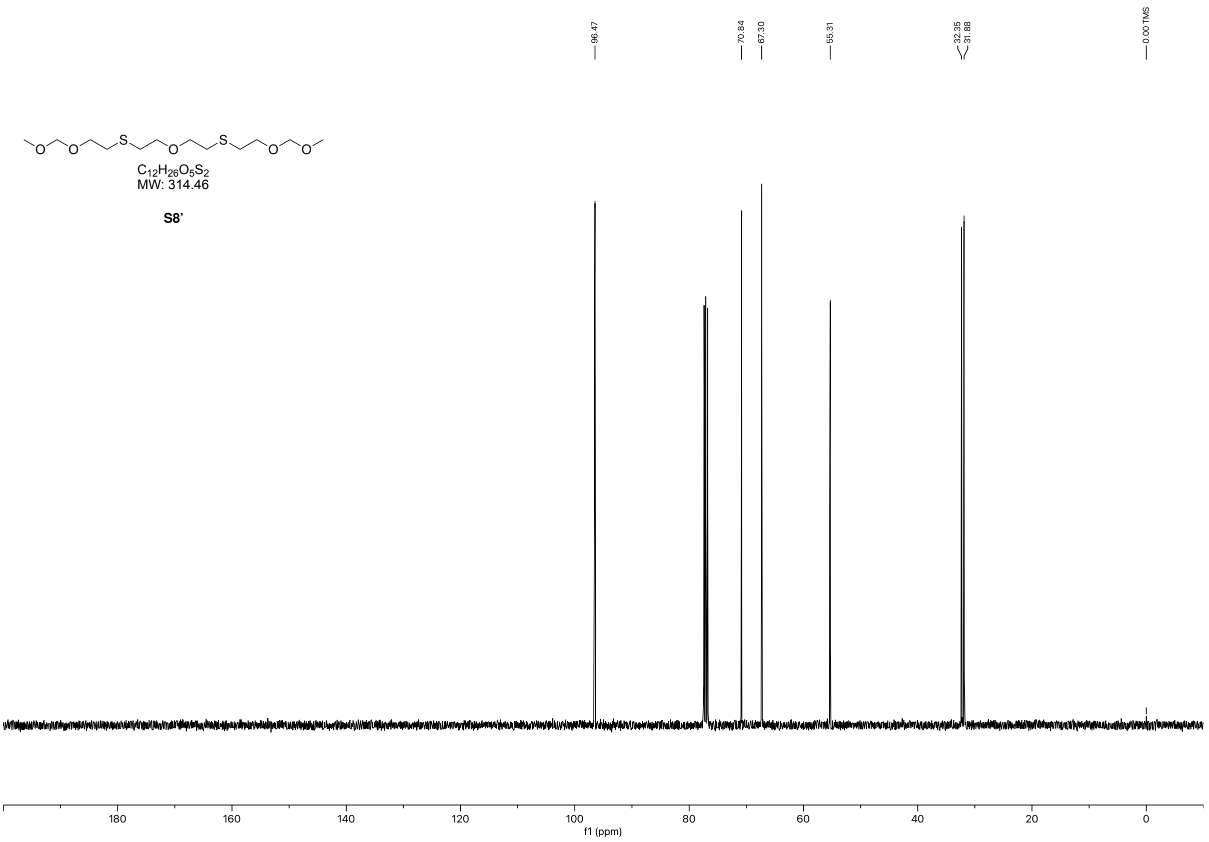
96.47
70.84
67.30
55.31
32.35
31.88
0.00 TMS
$C_{12}H_{26}O_5S_2$
MW: 314.46
S8'
180 160 140 120 100 80 60 40 20 0
f1 (ppm)

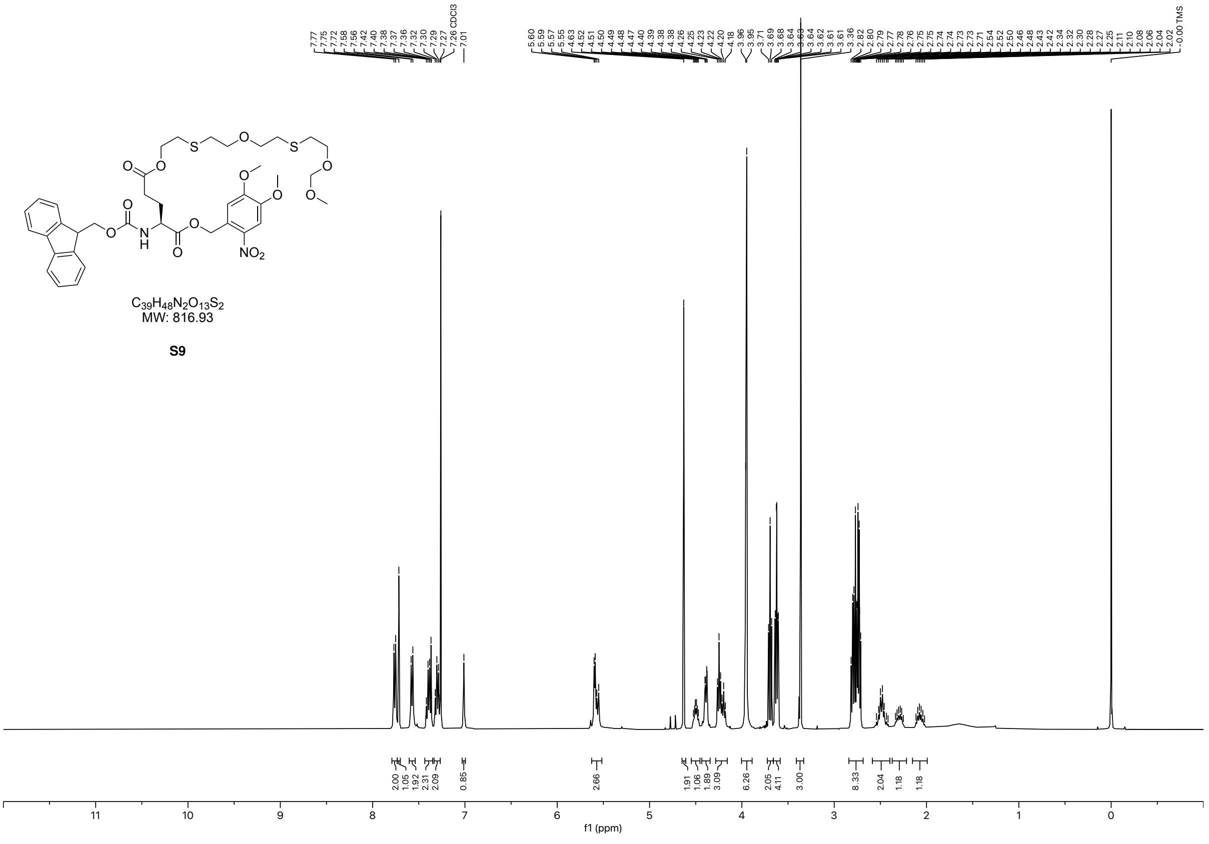
NO2
C39H48N2O13S2
MW: 816.93
S9
f1 (ppm)

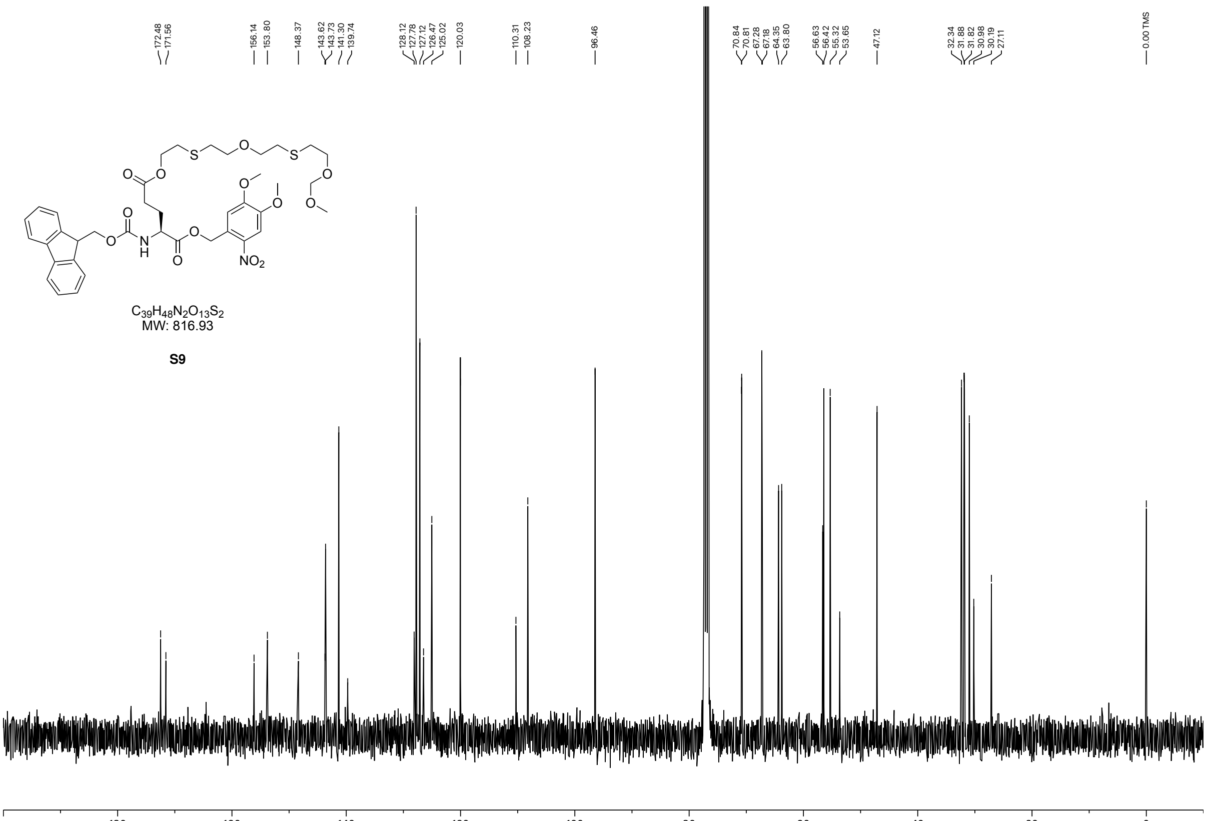
172.48
171.56
156.14
153.80
148.37
143.62
142.73
141.30
139.74
128.12
127.78
127.12
126.47
125.02
120.03
110.31
108.23
96.46
70.84
70.81
67.28
67.18
64.35
63.80
56.63
56.42
55.32
53.65
47.12
32.34
31.88
31.82
30.98
30.19
27.11
0.00 TMS
NO2
C39H48N2O13S2
MW: 816.93
S9
f1 (ppm)

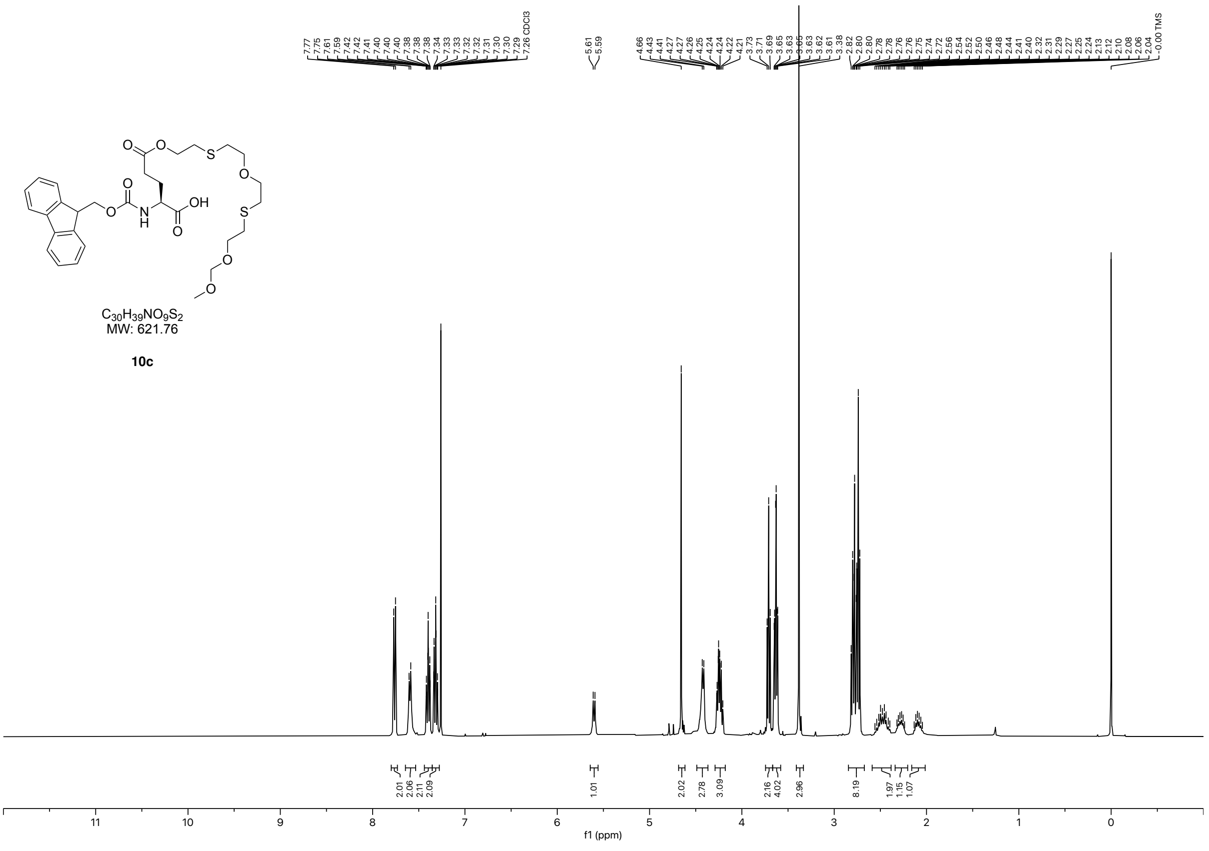

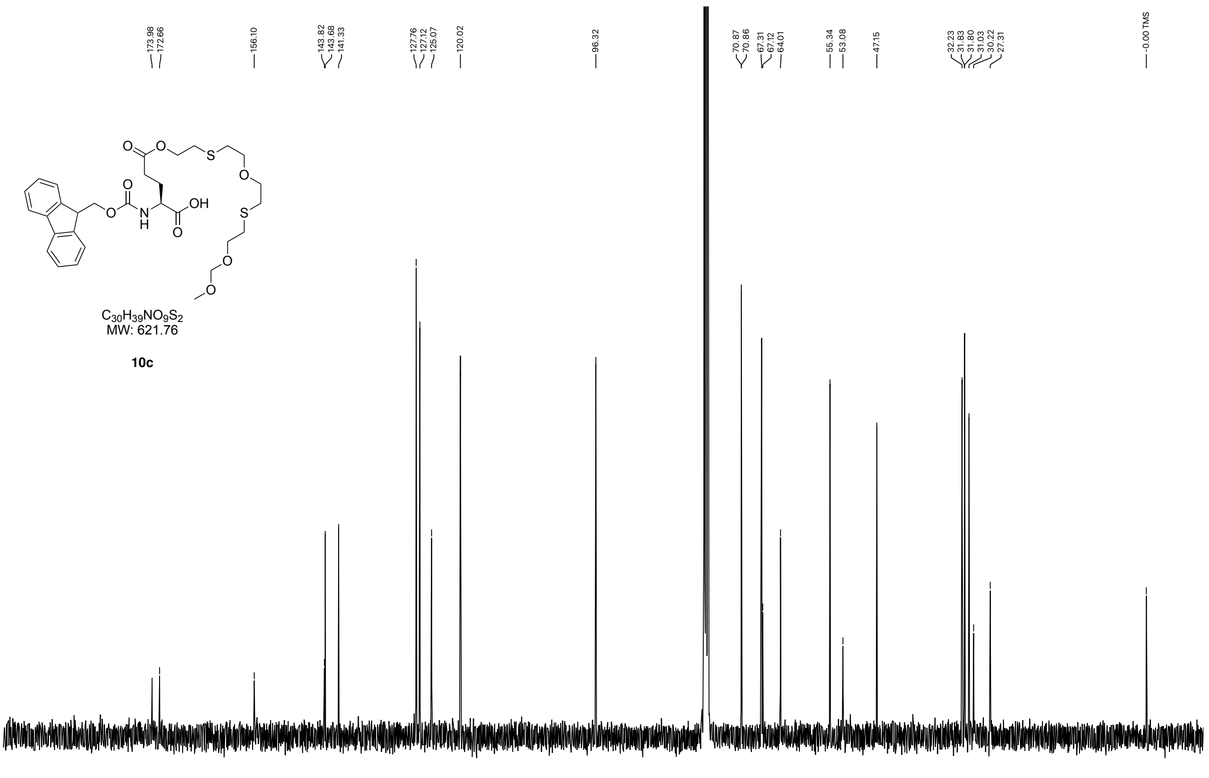

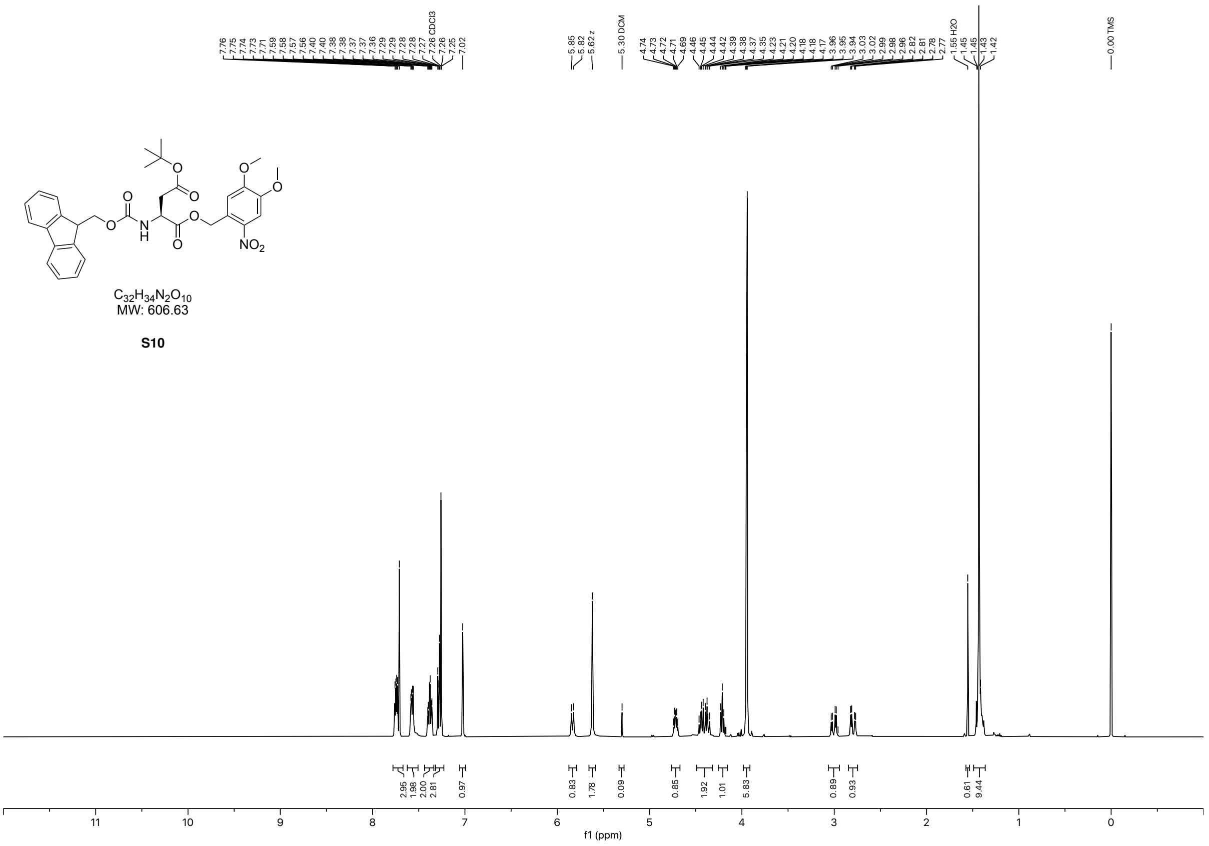
7.76 7.75 7.74 7.73 7.71 7.59 7.58 7.57 7.56 7.40 7.40 7.38 7.38 7.37 7.37 7.36 7.29 7.29 7.28 7.28 7.27 7.26 CDCl3 7.26 7.25 7.02
5.85 5.82 5.62 z
5.30 DCM
4.74 4.73 4.72 4.71 4.69 4.46 4.45 4.44 4.42 4.39 4.38 4.37 4.35 4.23 4.21 4.20 4.18 4.18 4.17 3.96 3.95 3.94 3.03 3.02 2.99 2.98 2.96 2.82 2.81 2.78 2.77
1.55 H2O 1.45 1.45 1.43 1.42
0.00 TMS
$C_{32}H_{34}N_2O_{10}$
MW: 606.63
S10
2.95 1.98 2.00 2.81 0.97 0.83 1.78 0.09 0.85 1.92 1.01 5.83 0.89 0.93 0.61 9.44
11 10 9 8 7 6 5 4 3 2 1 0
f1 (ppm)

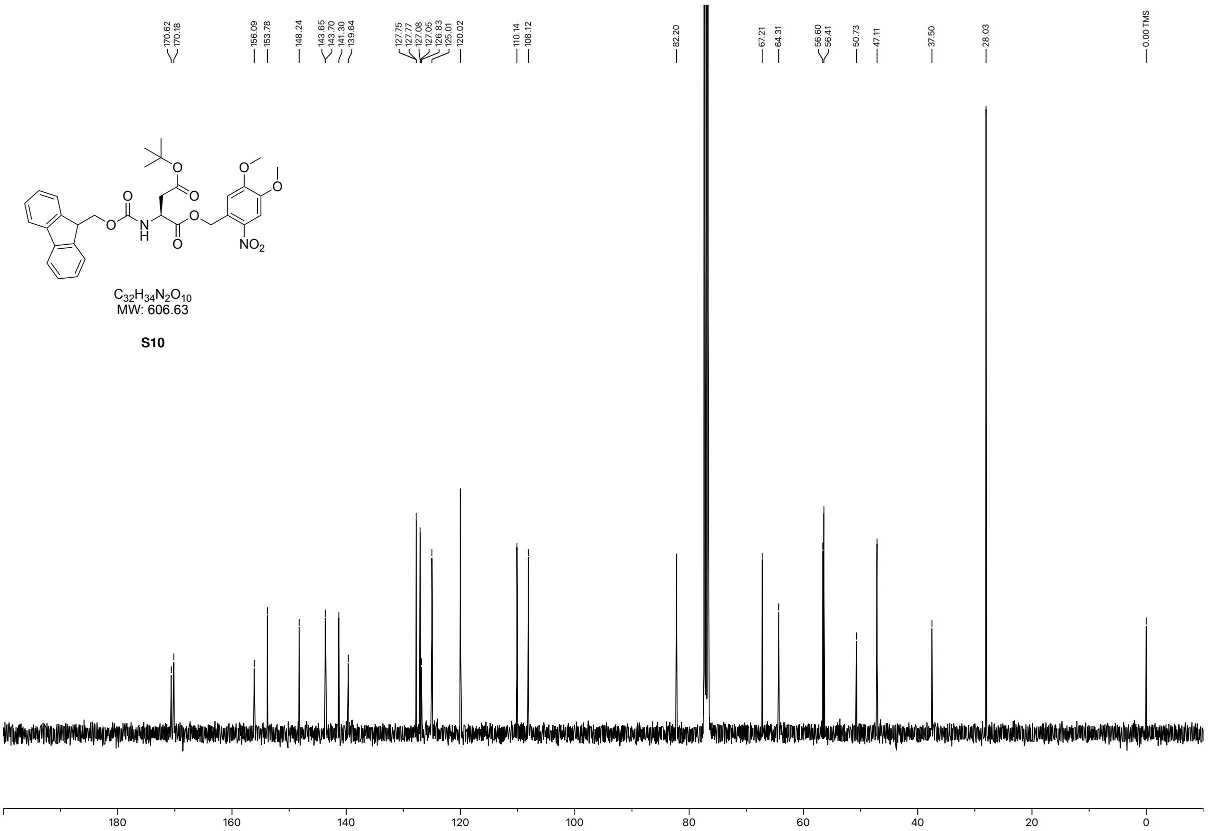
170.62 170.18
156.09 153.78 148.24 143.65 143.70 141.30 139.64
127.75 127.77 127.08 127.05 126.83 125.01 120.02
110.14 108.12
82.20
67.21 64.31 56.60 56.41 50.73 47.11 37.50 28.03
0.00 TMS
$C_{32}H_{34}N_2O_{10}$
MW: 606.63
S10
180 160 140 120 100 80 60 40 20 0
f1 (ppm)

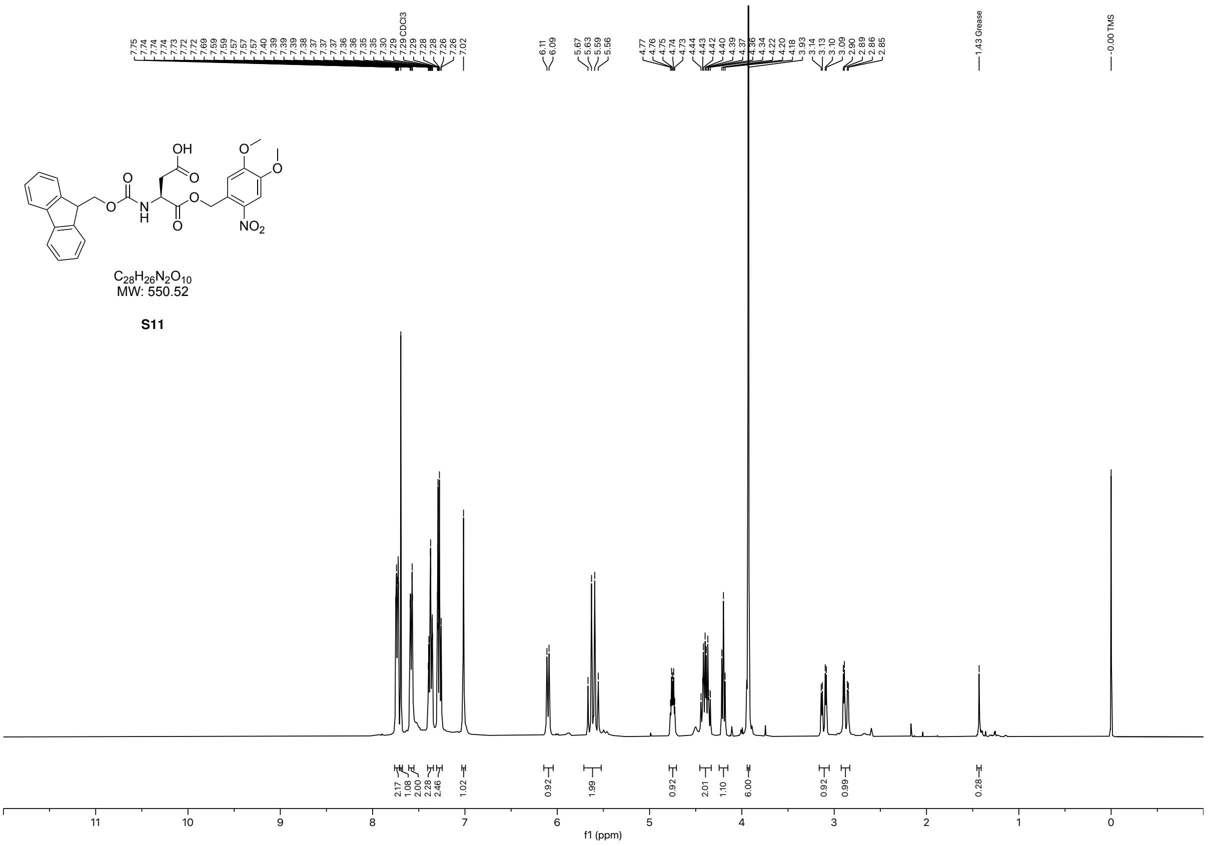
7.75 7.74 7.74 7.74 7.73 7.72 7.72 7.69 7.59 7.59 7.57 7.57 7.57 7.57 7.40 7.39 7.39 7.39 7.38 7.37 7.37 7.37 7.36 7.36 7.35 7.35 7.30 7.29 CDCl3 7.29 7.28 7.28 7.26 7.26 7.02
6.11 6.09
5.67 5.63 5.59 5.56
4.77 4.76 4.75 4.74 4.73 4.44 4.43 4.42 4.40 4.39 4.37 4.36 4.34 4.22 4.20 4.18 3.93
3.14 3.13 3.10 3.09 2.90 2.89 2.86 2.85
1.43 Grease
0.00 TMS
OH
O
NO2
N
H
$C_{28}H_{26}N_2O_{10}$
MW: 550.52
S11
2.17 1.08 2.00 2.28 2.46 1.02 0.92 1.99 0.92 2.01 1.10 6.00 0.92 0.99 0.28
f1 (ppm)

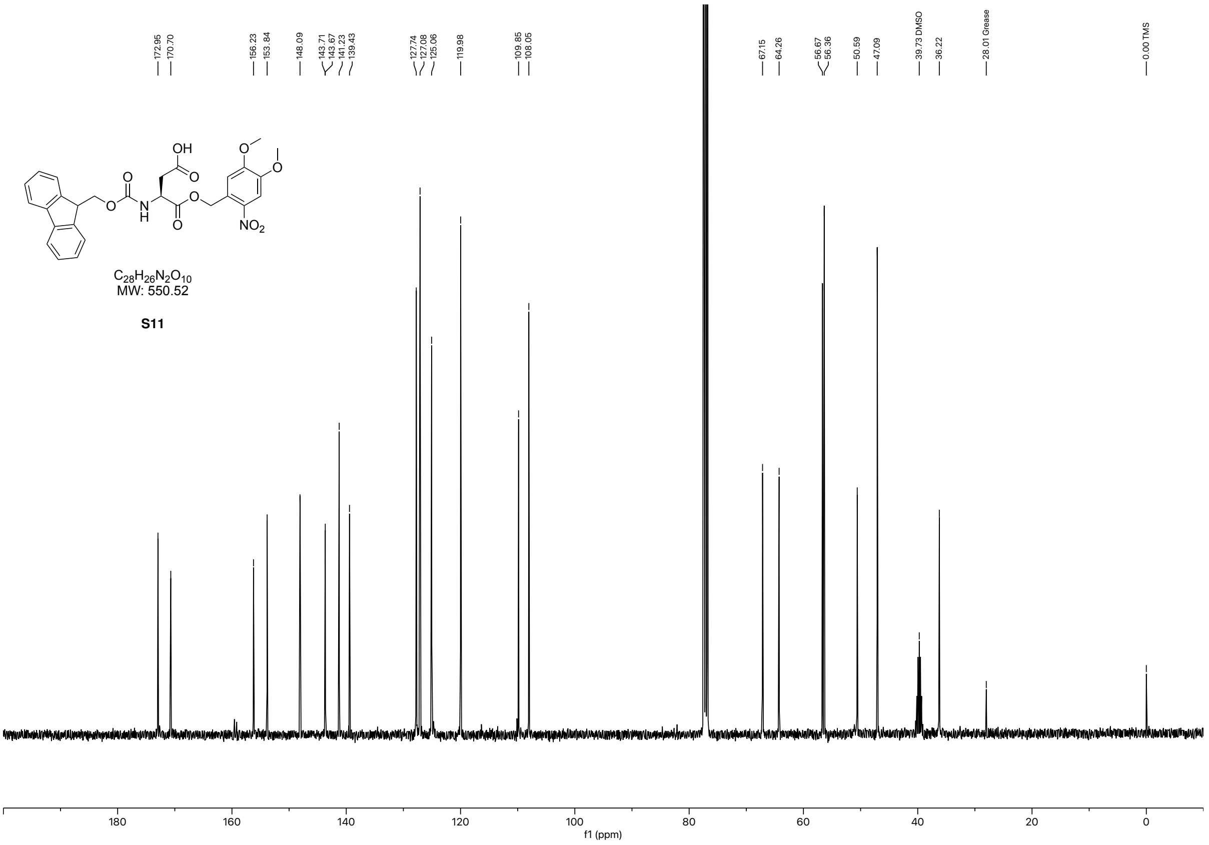
172.95 170.70
156.23 153.84
148.09
143.71 143.67 141.23 139.43
127.74 127.08 125.06
119.98
109.85 108.05
67.15 64.26
56.67 56.36
50.59 47.09
39.73 DMSO
36.22
28.01 Grease
0.00 TMS
OH
O
NO2
N
H
$C_{28}H_{26}N_2O_{10}$
MW: 550.52
S11
f1 (ppm)

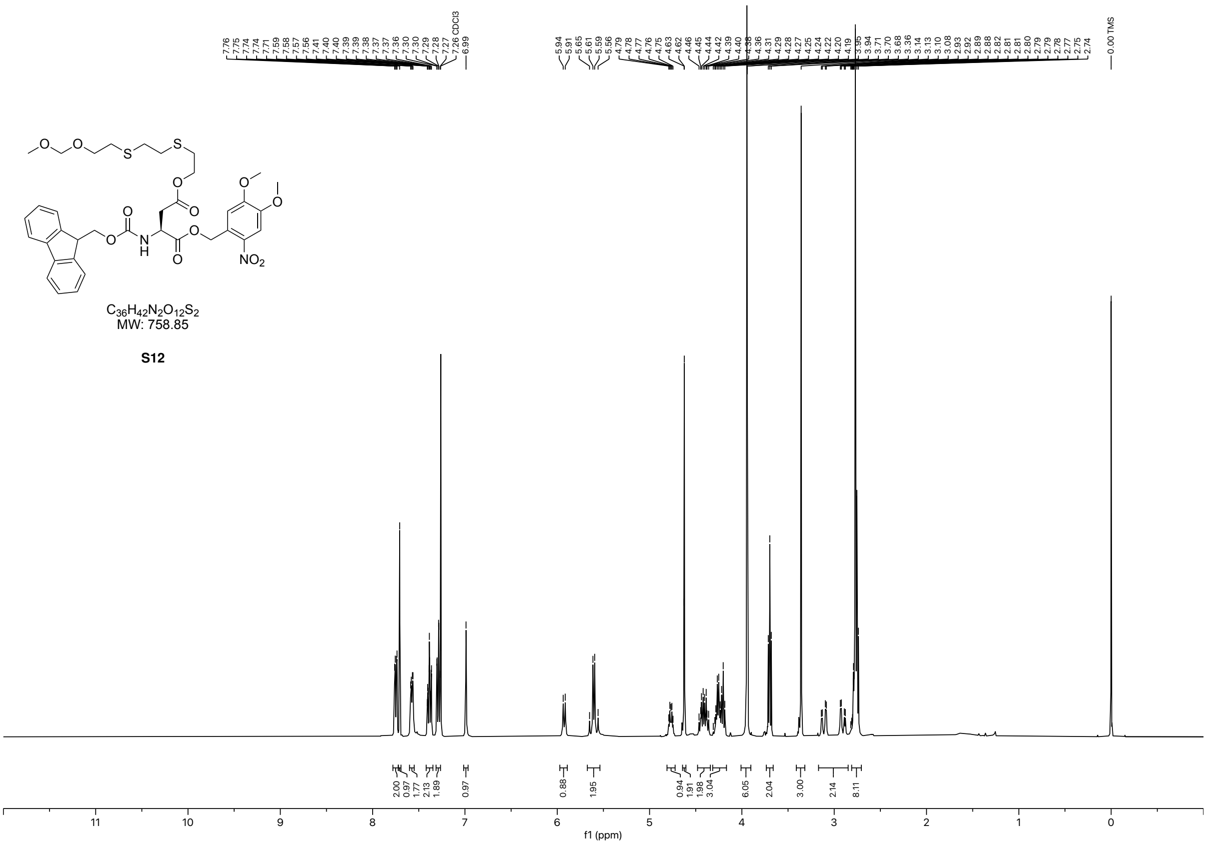

$C_{36}H_{42}N_2O_{12}S_2$
MW: 758.85

S12

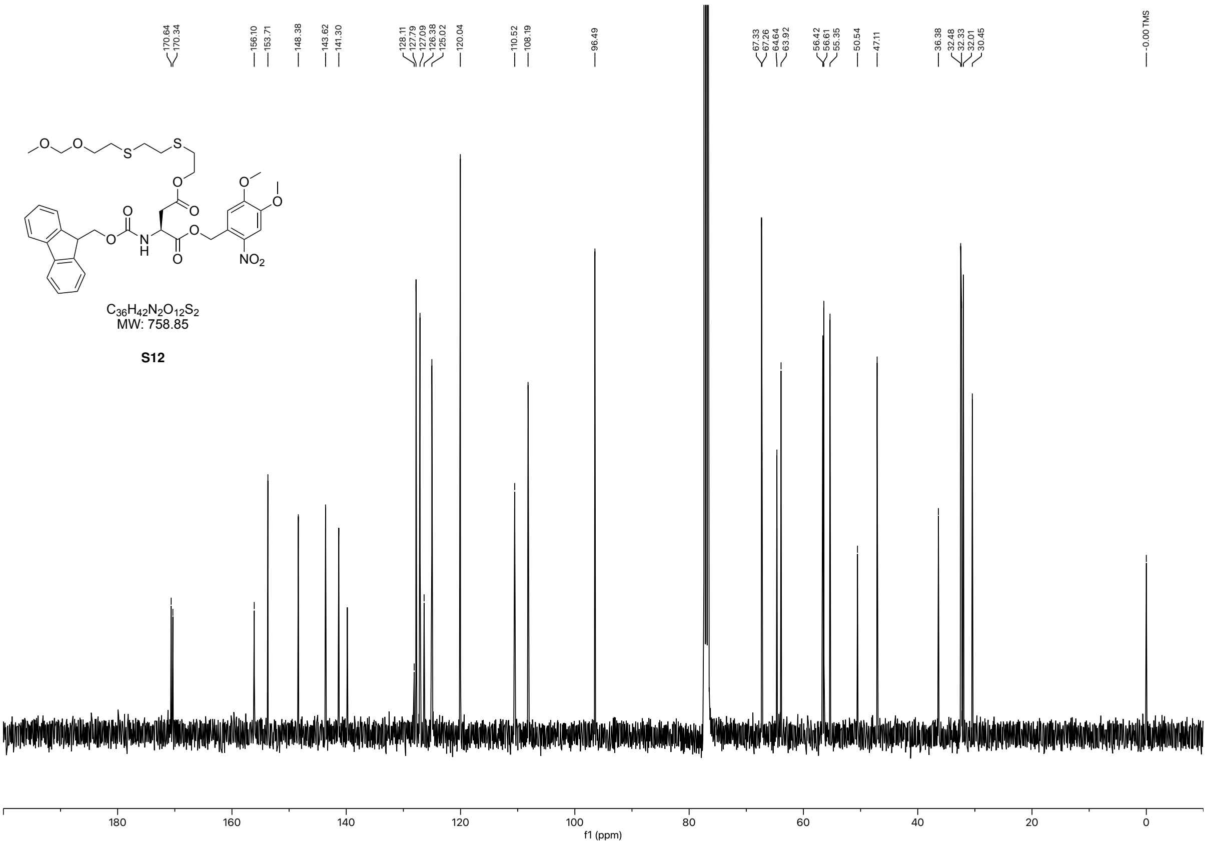

$C_{36}H_{42}N_2O_{12}S_2$
MW: 758.85

S12

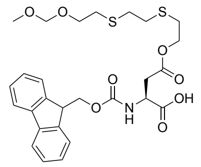

C$_{27}$H$_{33}$NO$_8$S$_2$
MW: 563.68

13

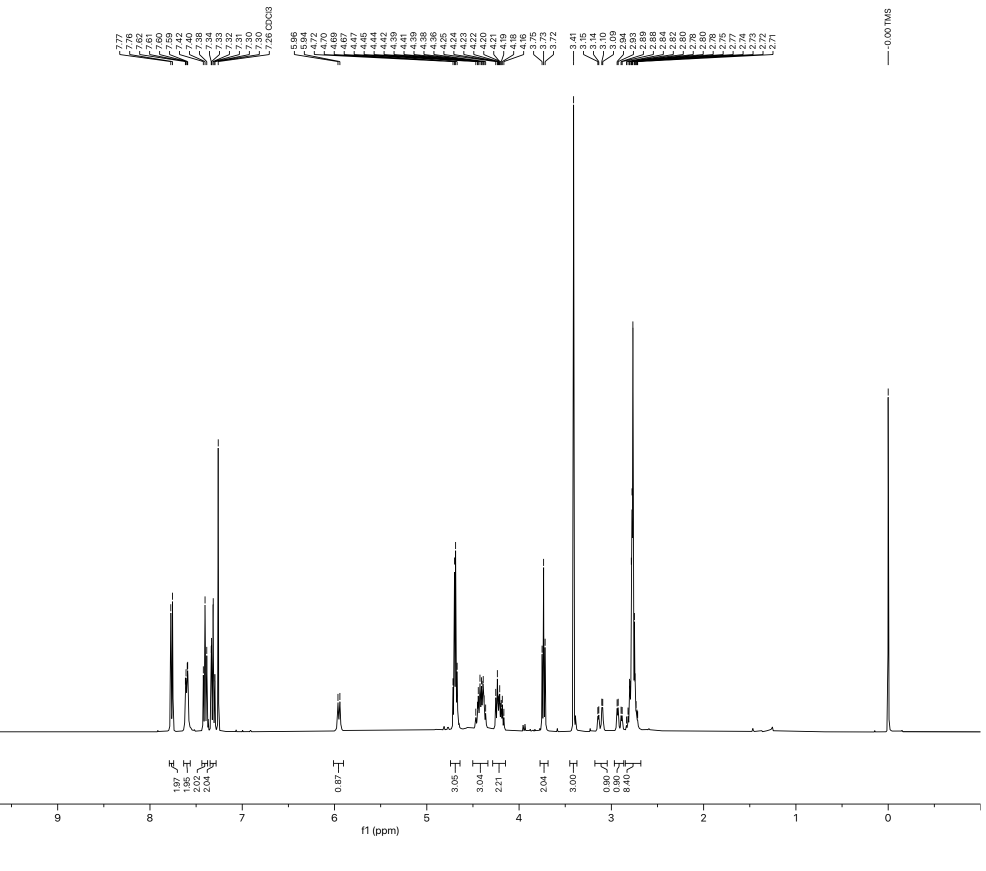

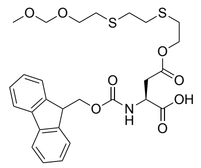

C$_{27}$H$_{33}$NO$_8$S$_2$
MW: 563.68

13

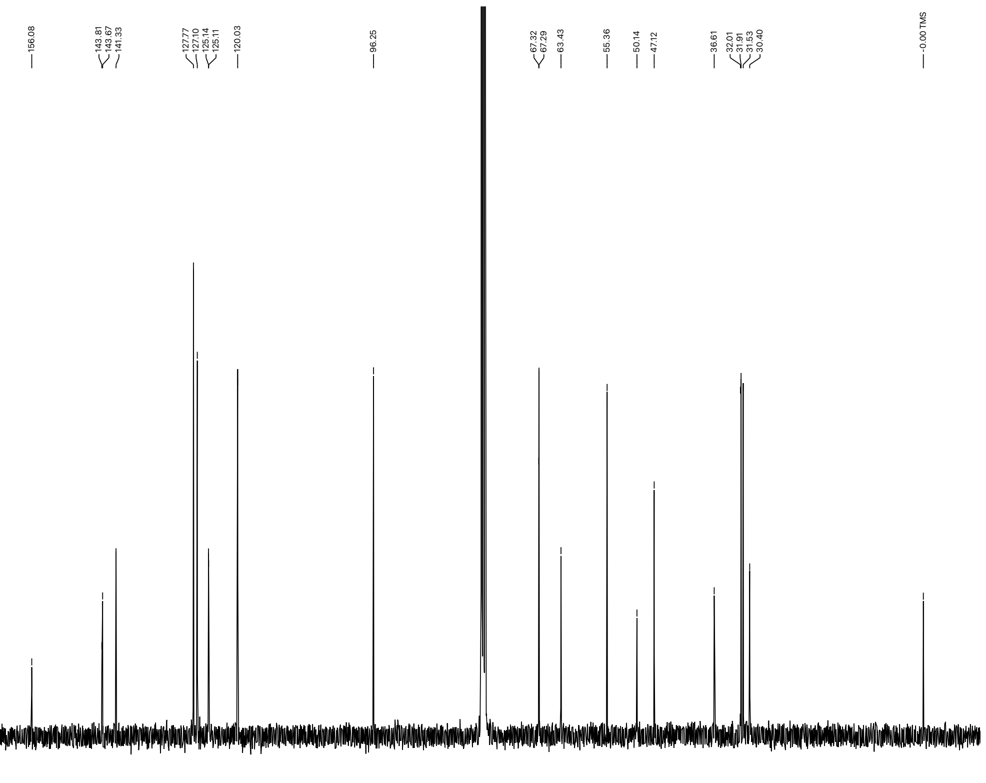

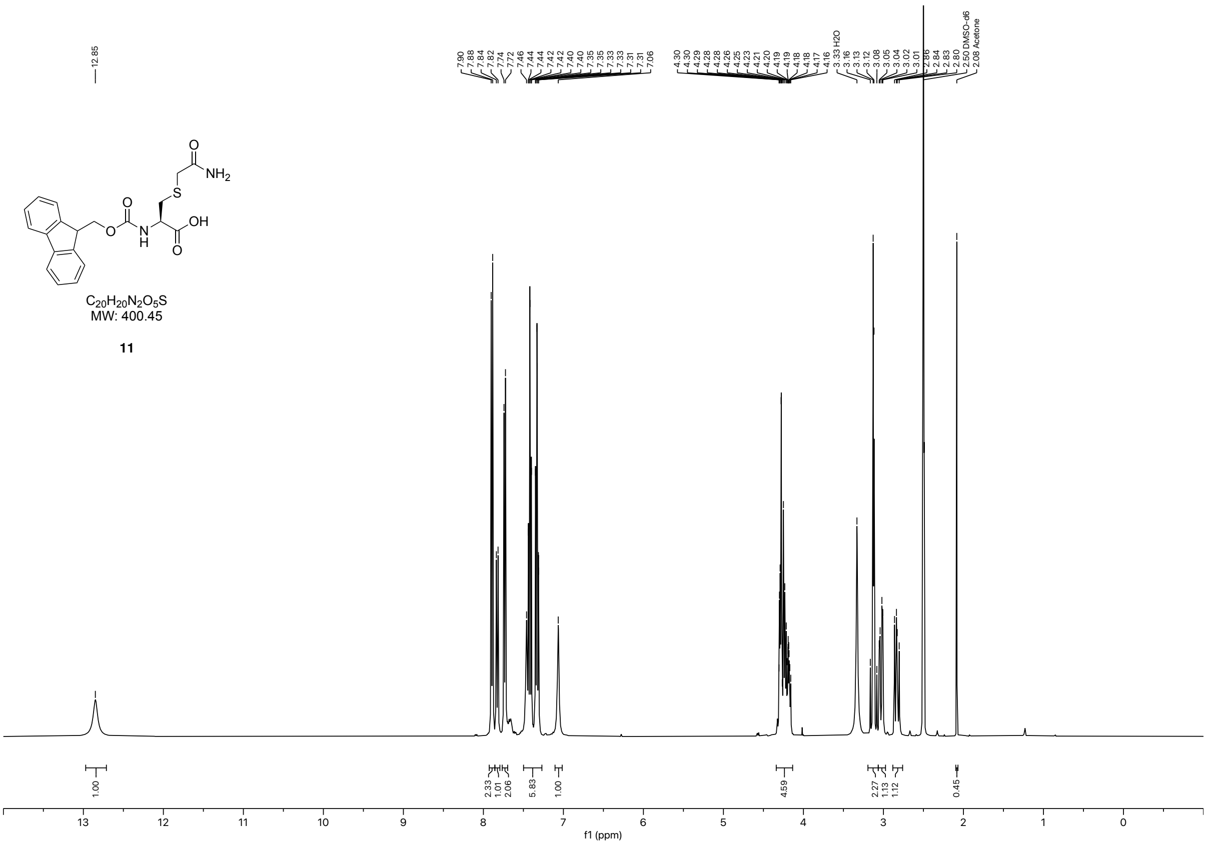

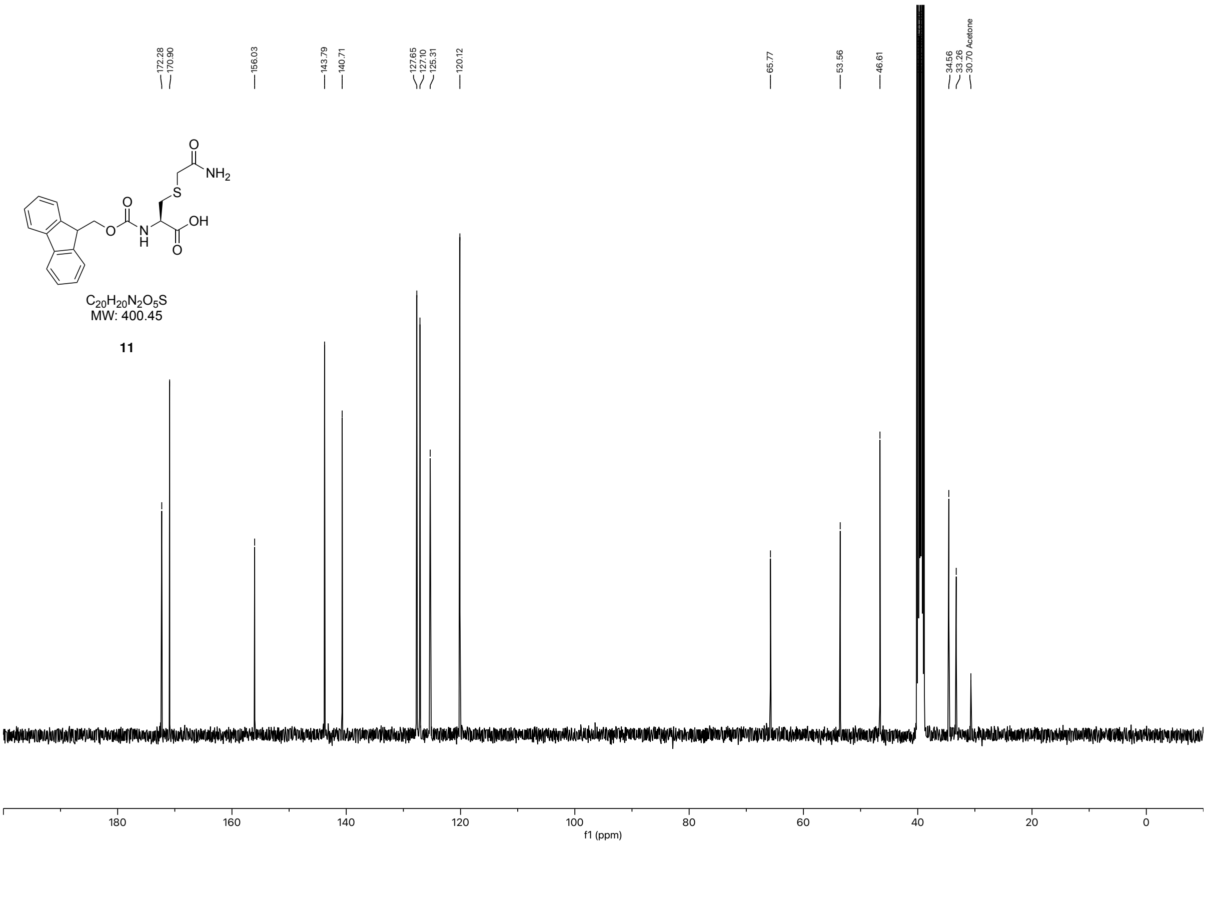

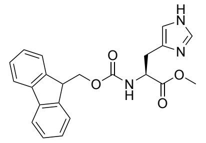

$C_{22}H_{21}N_3O_4$
MW: 391.43

S13

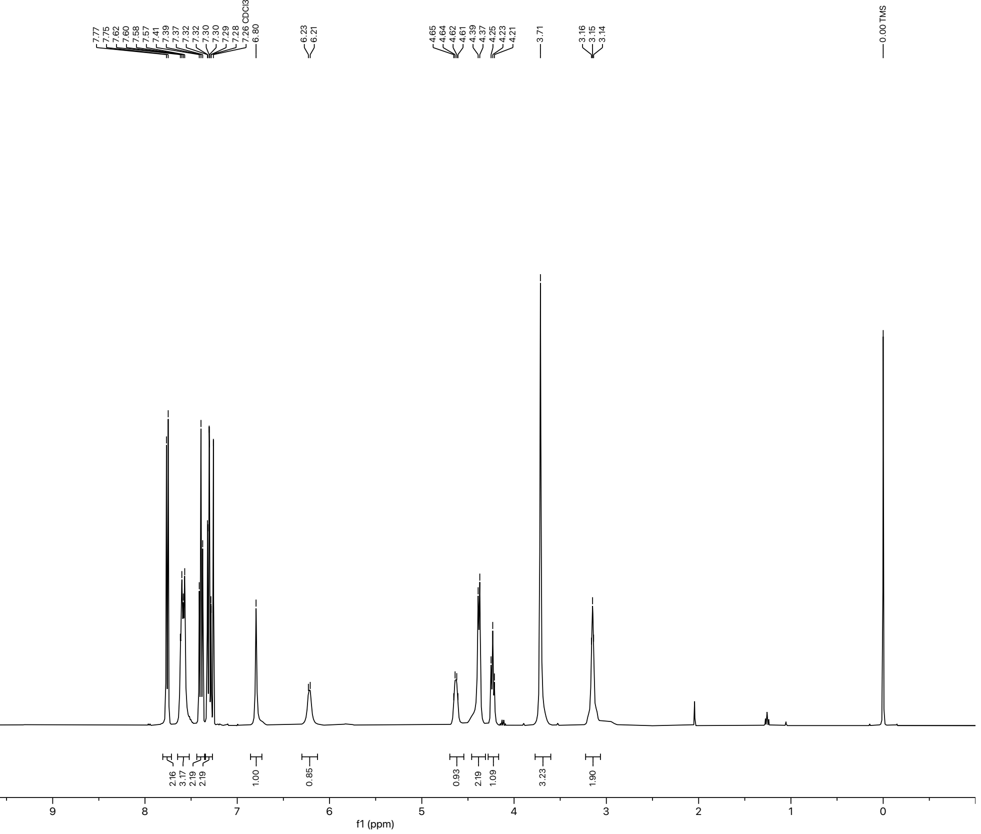

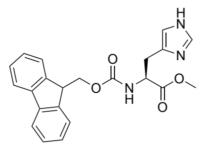

$C_{22}H_{21}N_3O_4$
MW: 391.43

S13

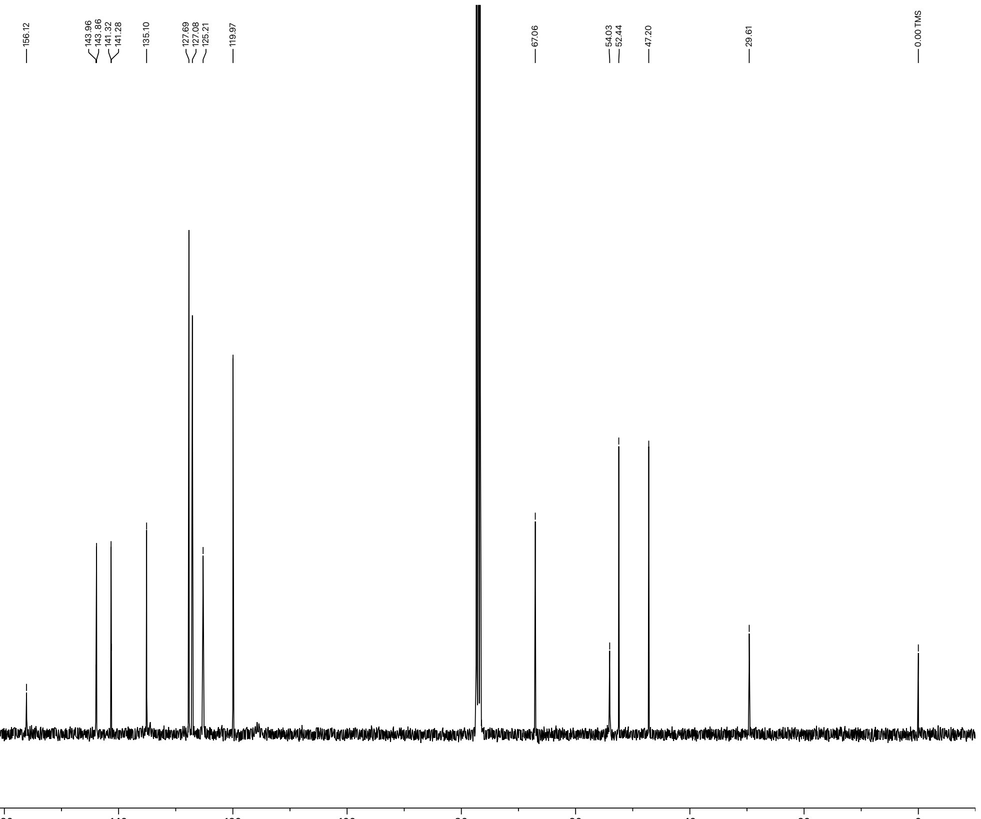

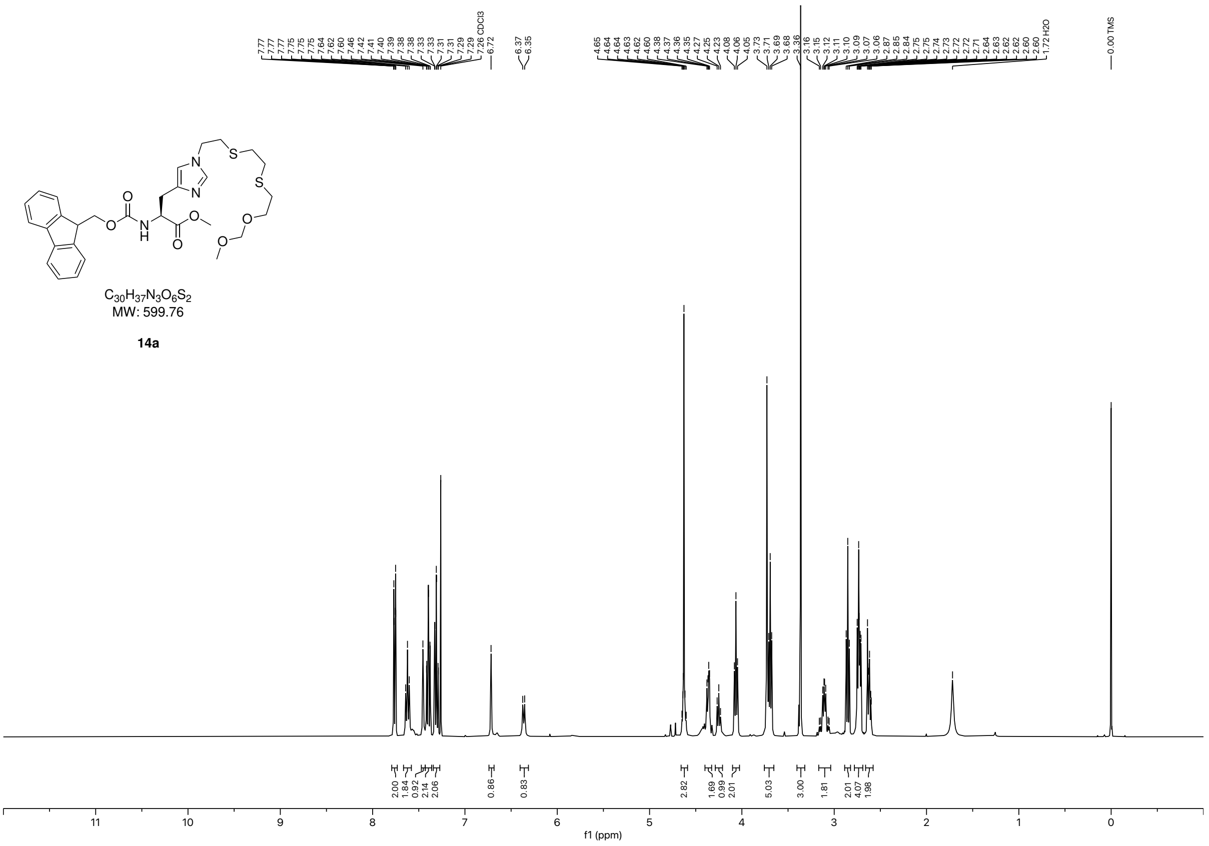
C30H37N3O6S2
MW: 599.76
14a
f1 (ppm)

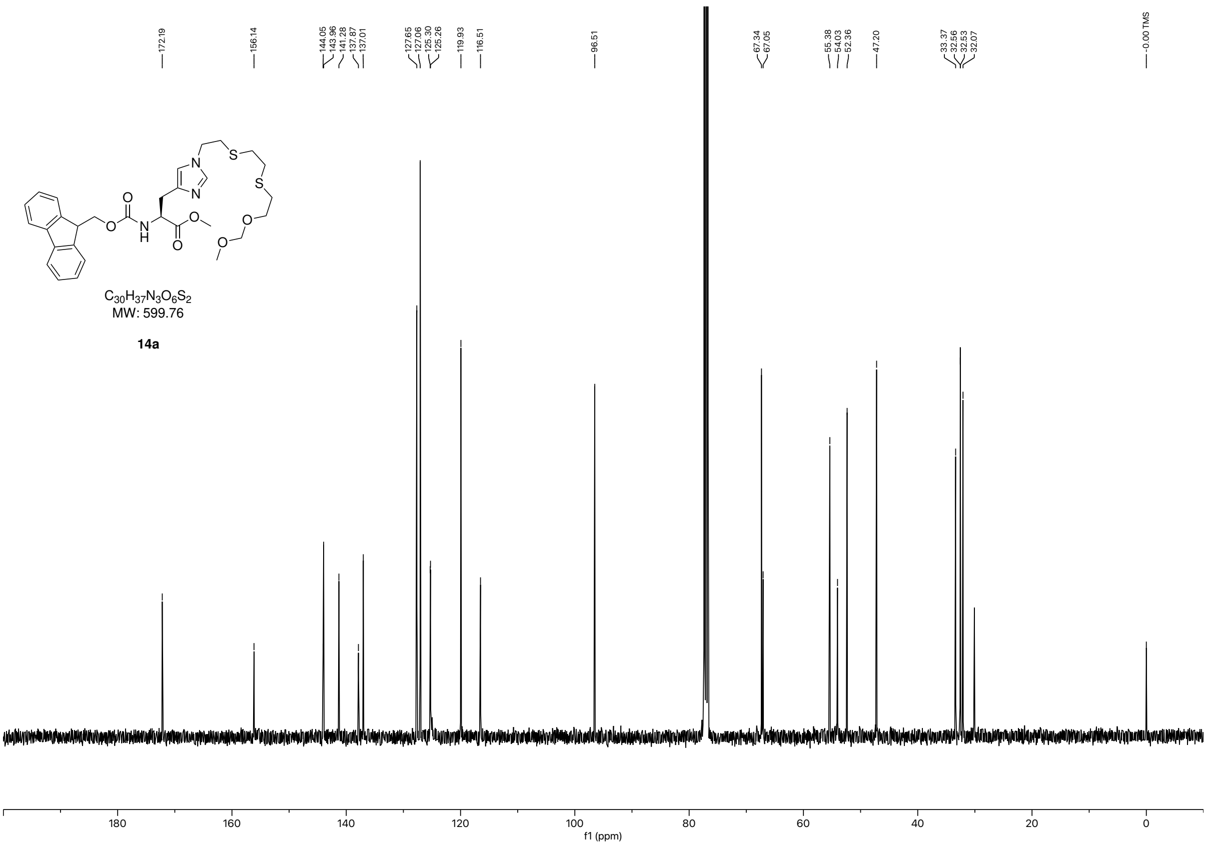
C30H37N3O6S2
MW: 599.76
14a
f1 (ppm)

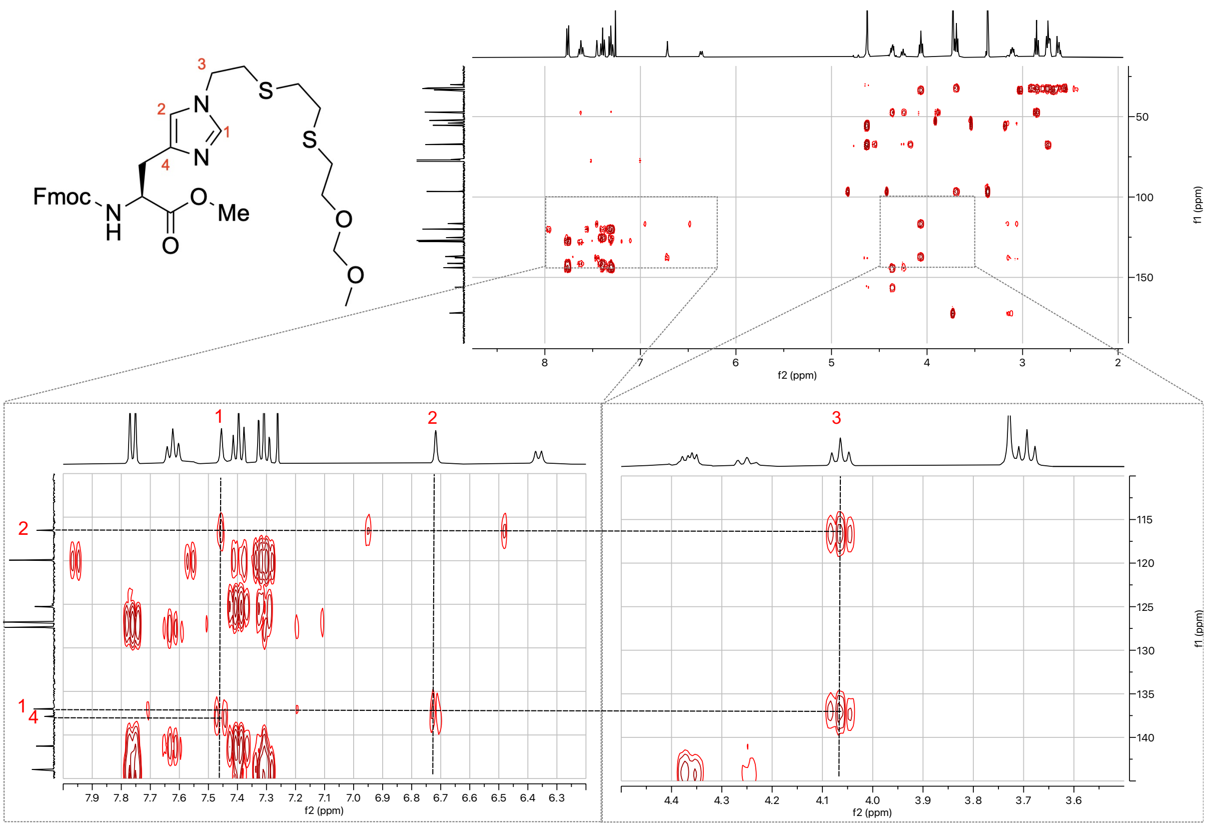

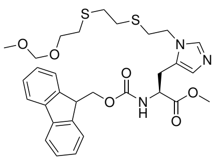

$C_{30}H_{37}N_3O_6S_2$
MW: 599.76

14b

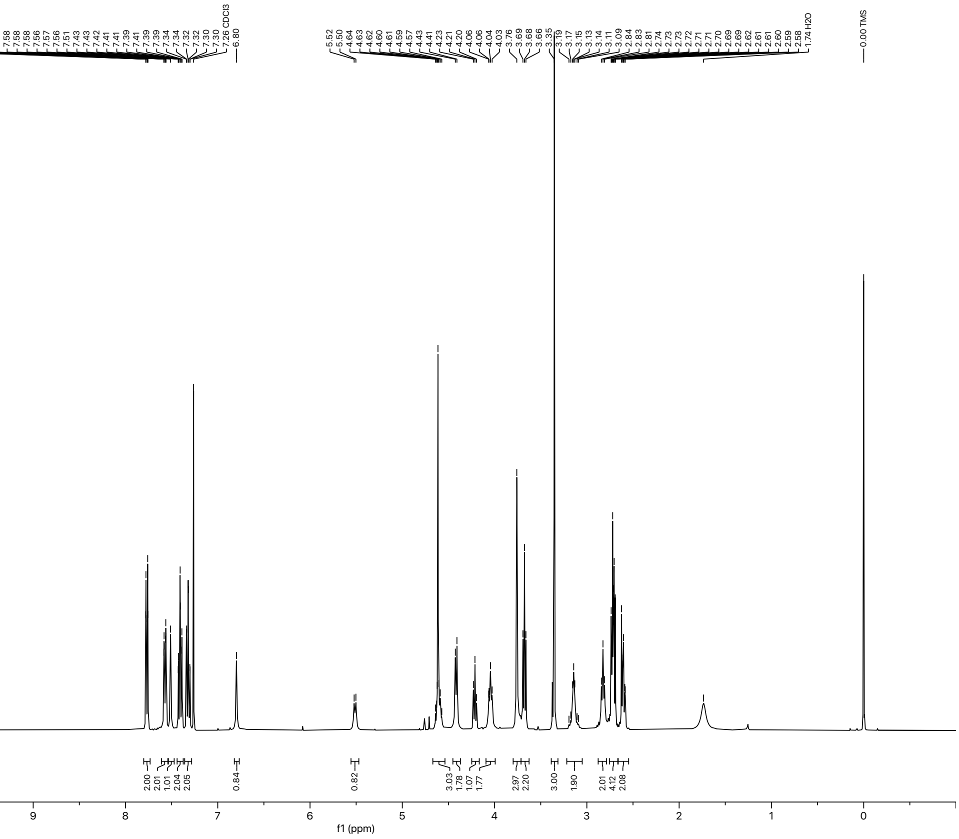

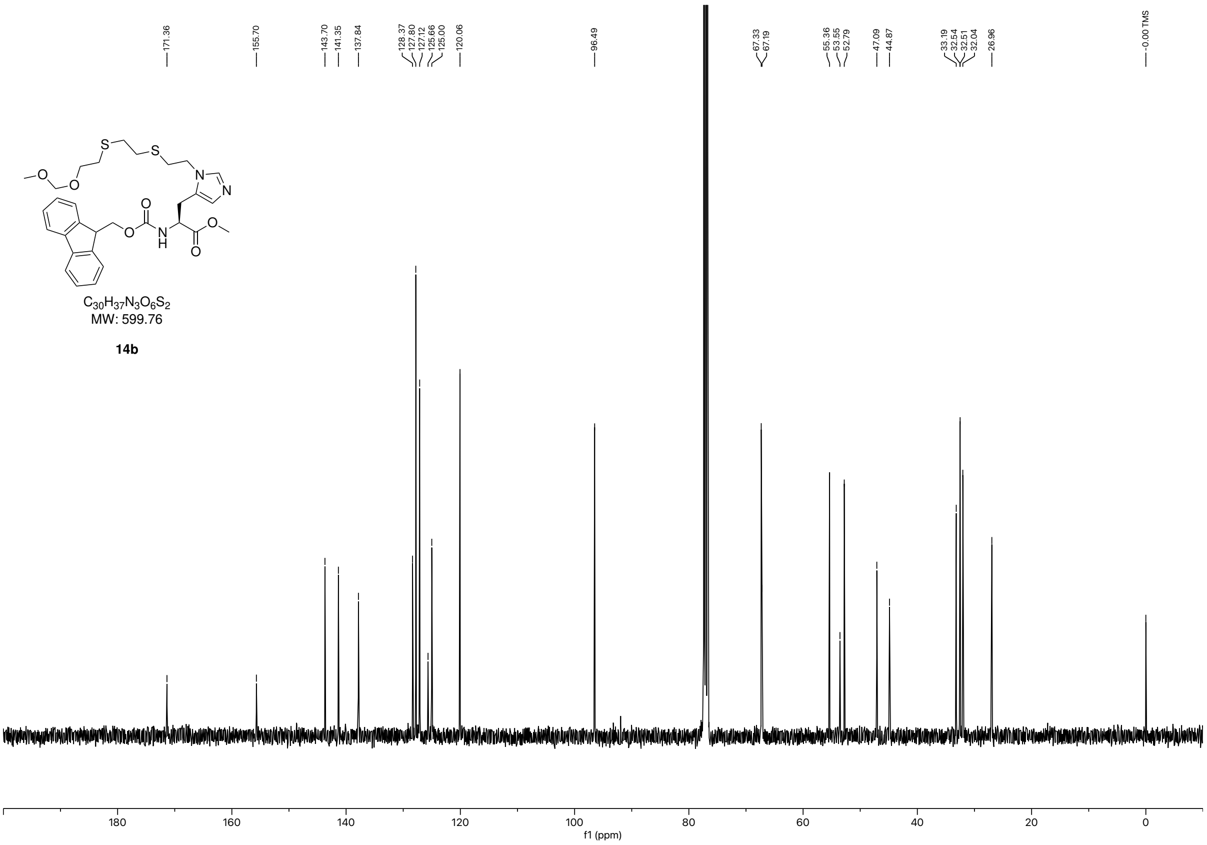
171.36
155.70
143.70
141.35
137.84
128.37
127.80
127.12
125.66
125.00
120.06
96.49
67.33
67.19
55.36
53.55
52.79
47.09
44.87
33.19
32.54
32.51
32.04
26.96
0.00 TMS
$C_{30}H_{37}N_3O_6S_2$
MW: 599.76
14b
f1 (ppm)

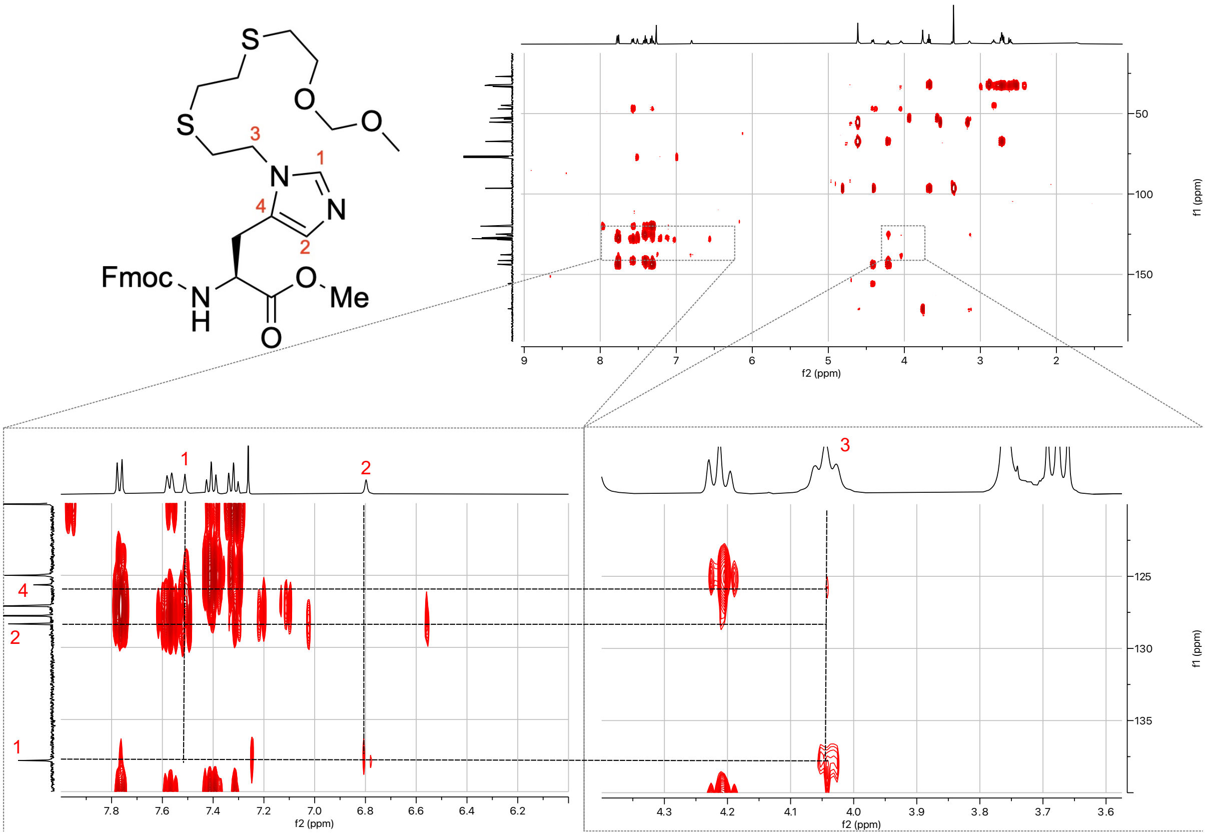
Fmoc
Me
f2 (ppm)
f1 (ppm)

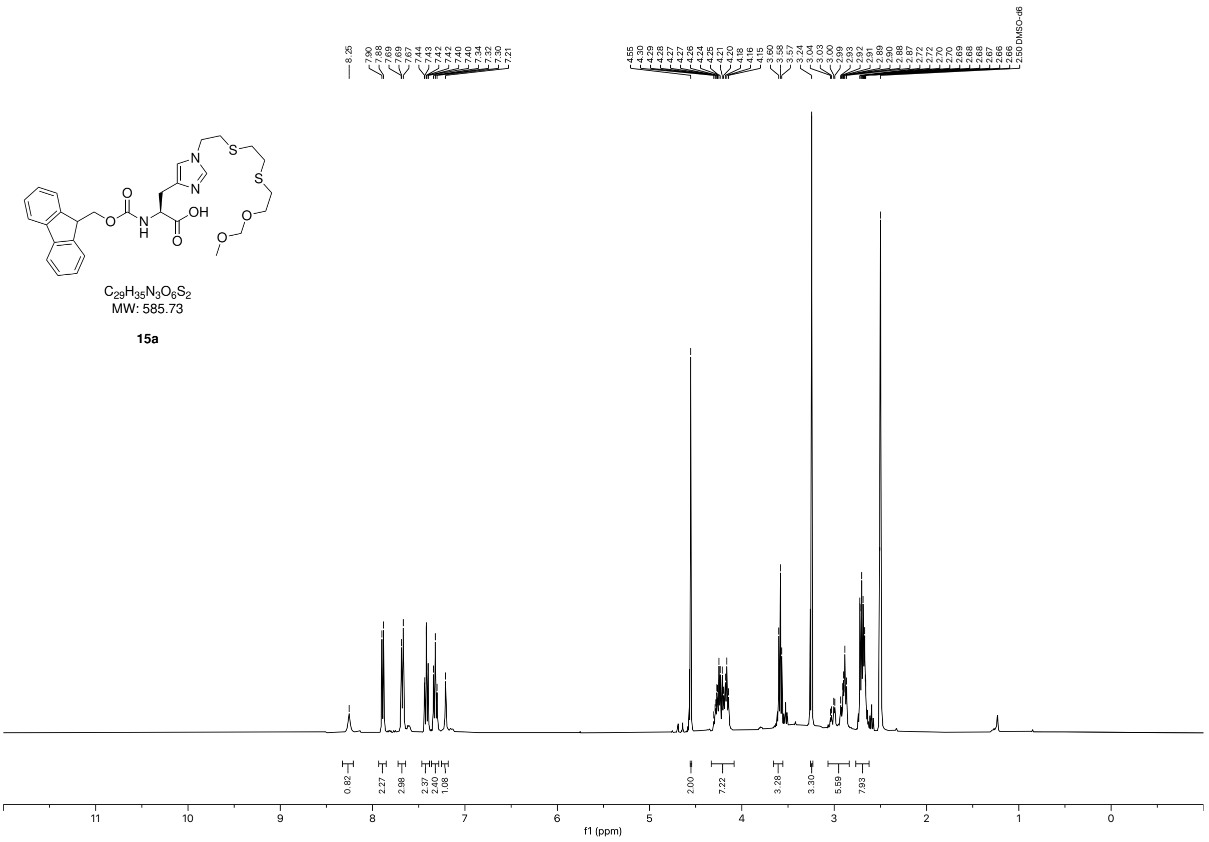

$C_{29}H_{35}N_3O_6S_2$
MW: 585.73

15a

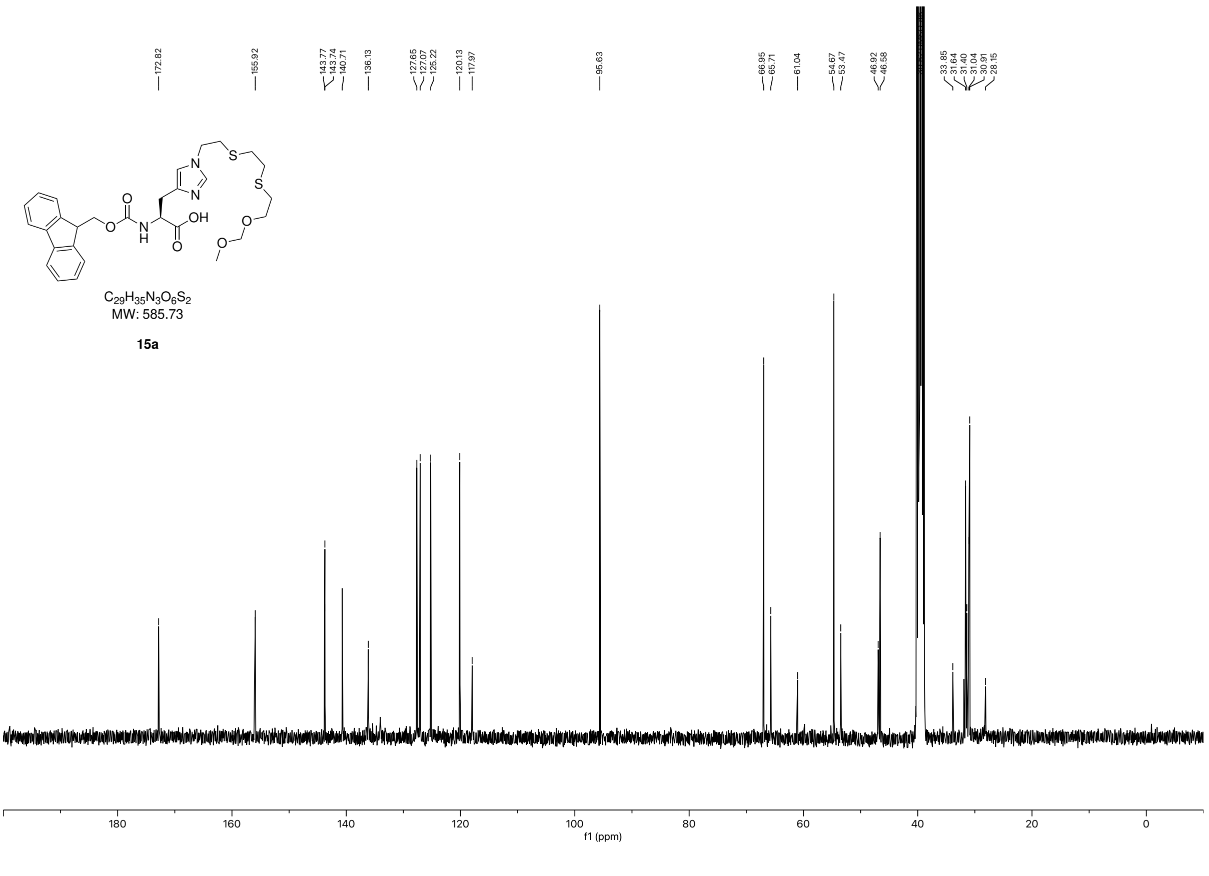

$C_{29}H_{35}N_3O_6S_2$
MW: 585.73

15a

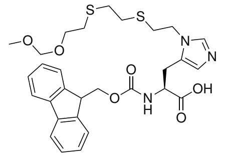

$C_{29}H_{35}N_3O_6S_2$
MW: 585.73

15b

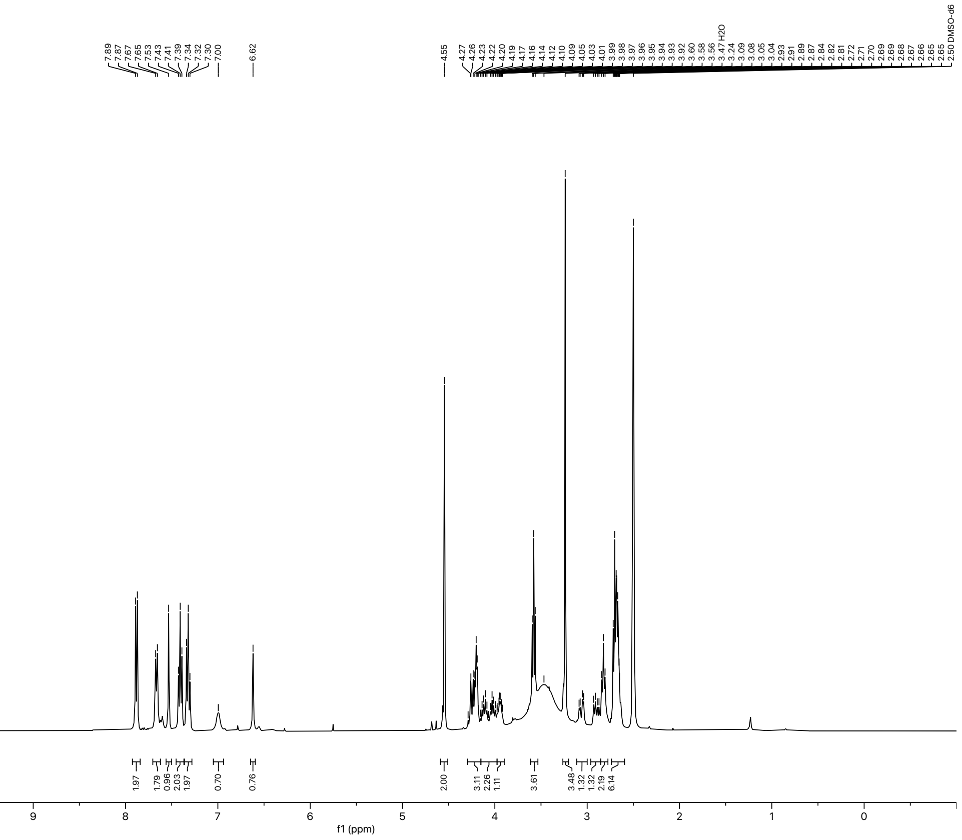

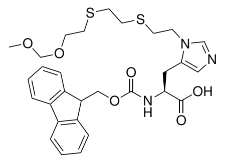

$C_{29}H_{35}N_3O_6S_2$
MW: 585.73

15b

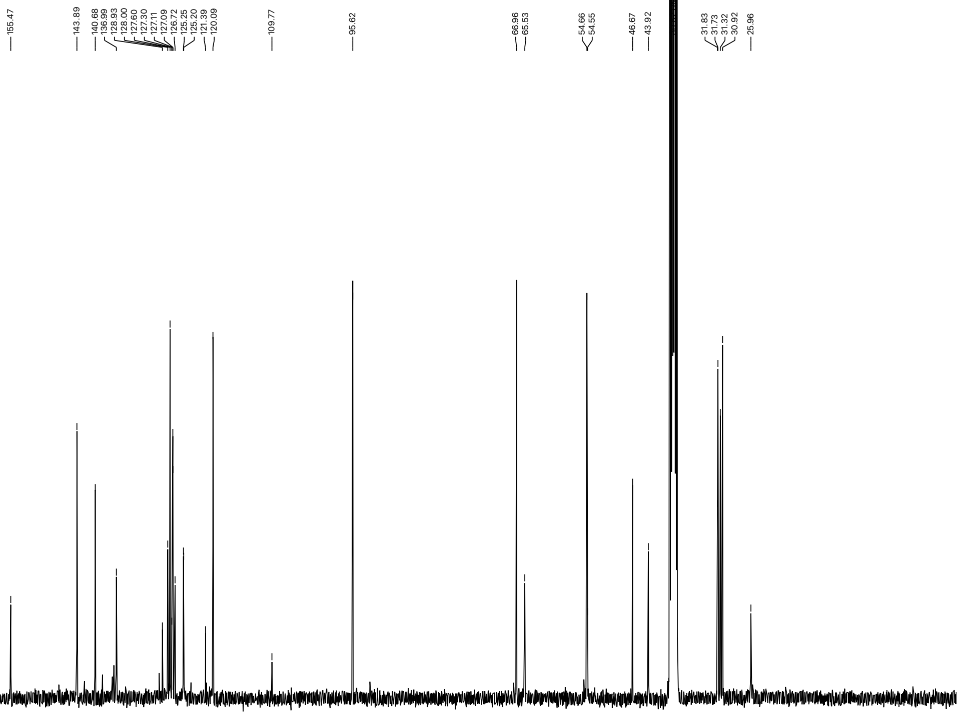

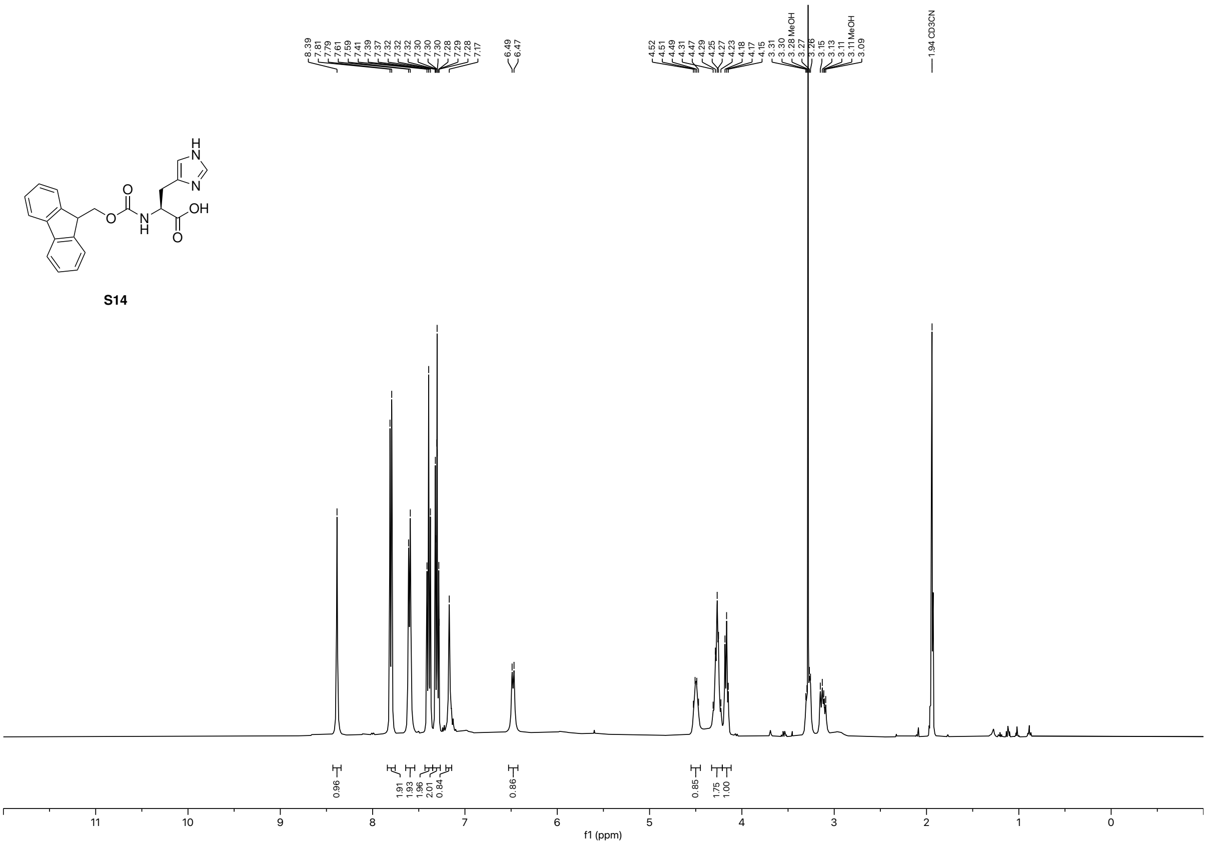

8.39 7.81 7.79 7.61 7.59 7.41 7.39 7.37 7.32 7.32 7.32 7.30 7.30 7.30 7.29 7.28 7.17
6.49 6.47
4.52 4.51 4.49 4.31 4.47 4.29 4.25 4.27 4.23 4.18 4.17 4.15 3.31 3.30 3.28 MeOH 3.27 3.26 3.15 3.13 3.11 3.11 MeOH 3.09
1.94 CD3CN
S14
0.96 1.91 1.93 1.96 2.01 0.84 0.86 0.85 1.75 1.00
11 10 9 8 7 6 5 4 3 2 1 0
f1 (ppm)

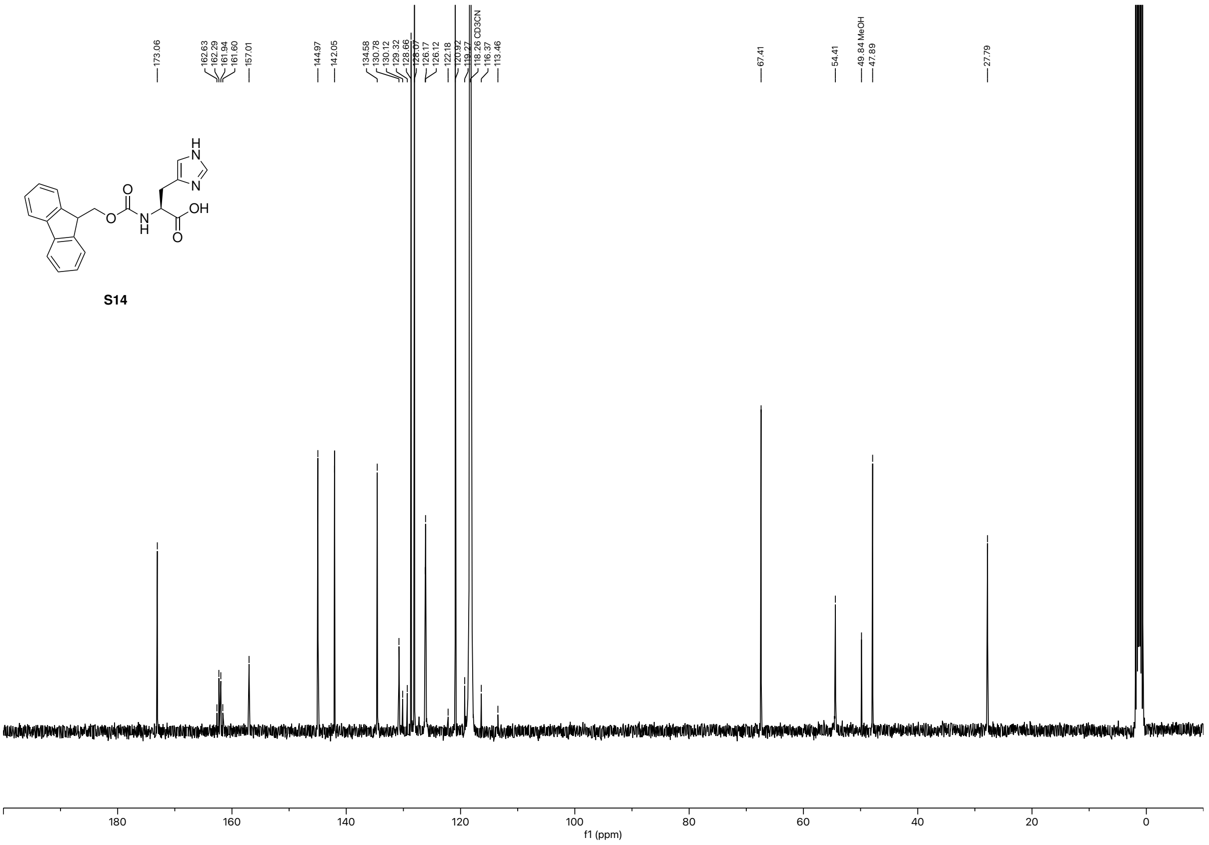

173.06
162.63 162.29 161.94 161.60 157.01
144.97 142.05
134.58 130.78 130.12 129.32 128.66 128.07 126.17 126.12 122.18 120.92 119.27 118.26 CD3CN 116.37 113.46
67.41
54.41 49.84 MeOH 47.89
27.79
S14
180 160 140 120 100 80 60 40 20 0
f1 (ppm)

$C_8H_{17}ClO_2S_2$
MW: 244.79

S15

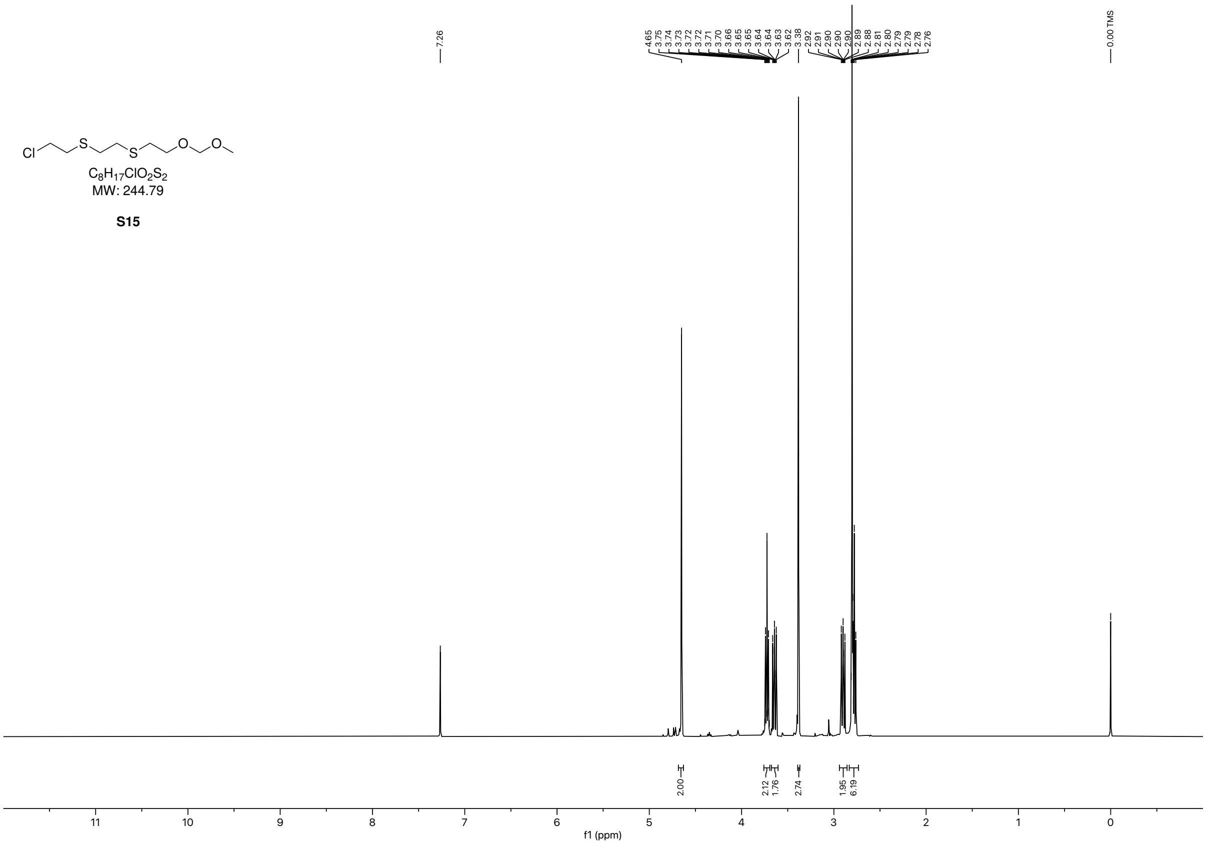

$C_8H_{17}ClO_2S_2$
MW: 244.79

S15

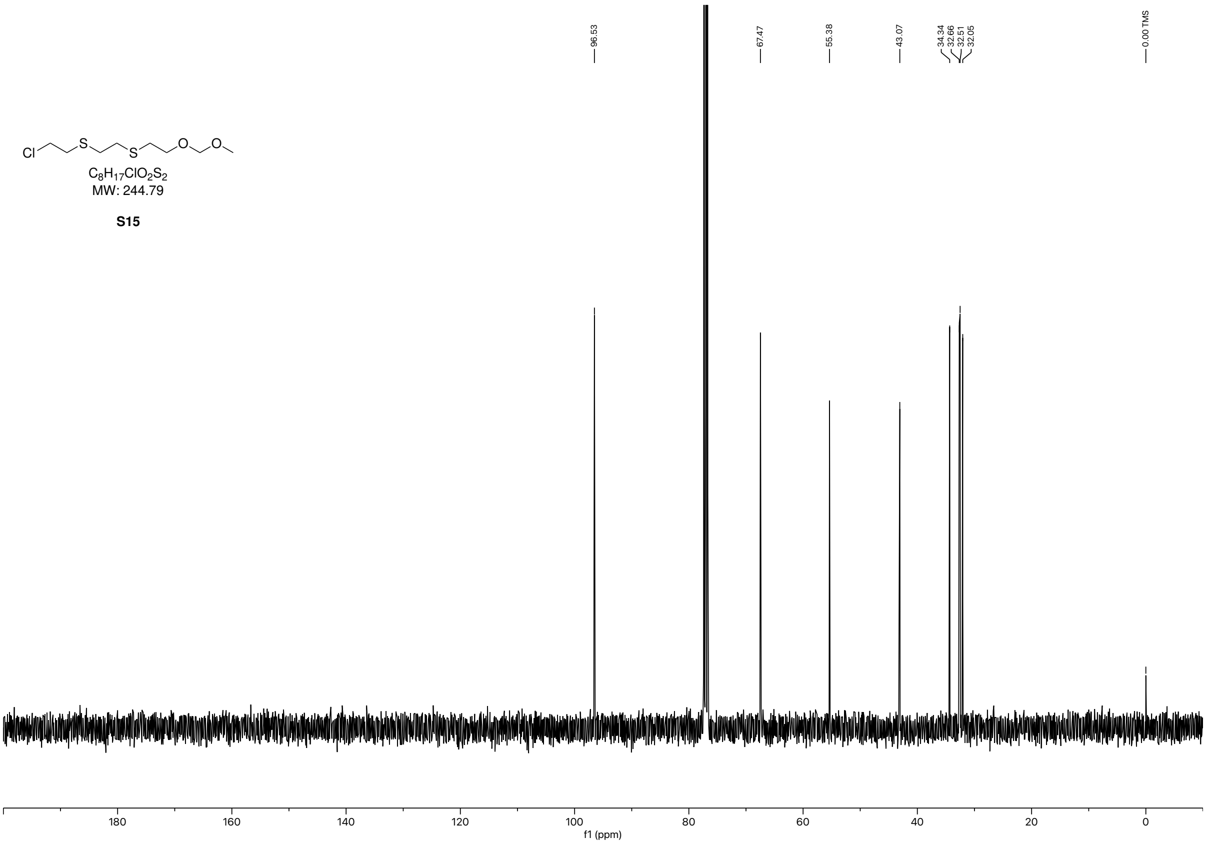

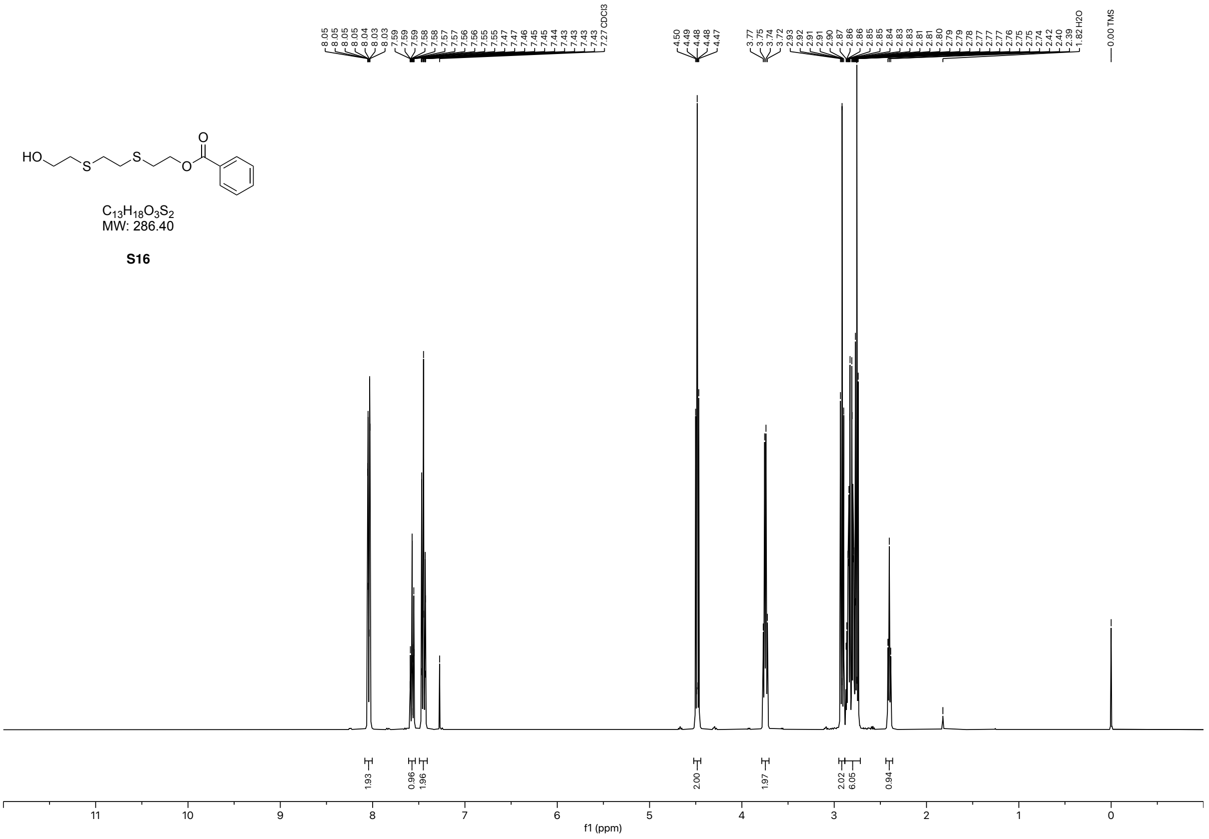
HO
S
S
O
O
$C_{13}H_{18}O_3S_2$
MW: 286.40
S16
f1 (ppm)

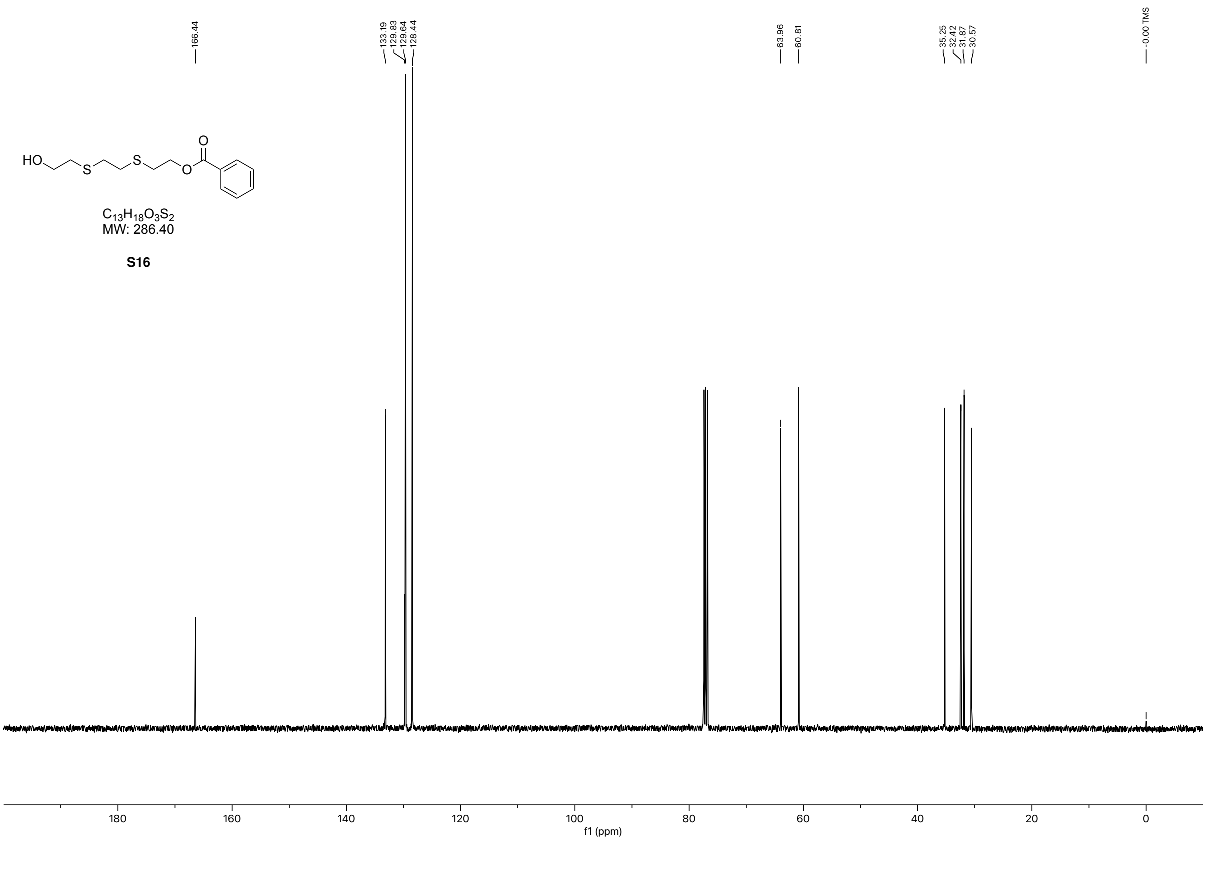
HO
S
S
O
O
$C_{13}H_{18}O_3S_2$
MW: 286.40
S16
f1 (ppm)

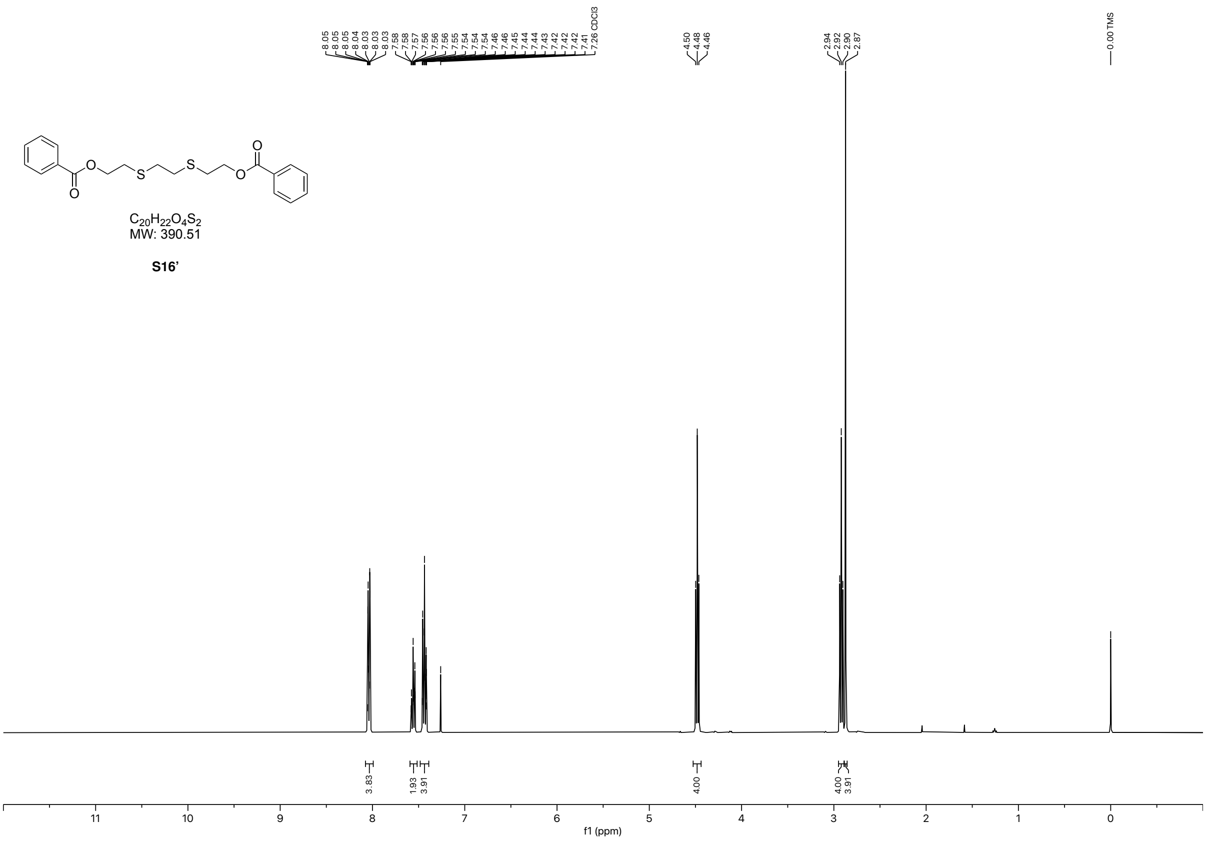

8.05 8.05 8.05 8.04 8.03 8.03 8.03 7.58 7.58 7.57 7.56 7.56 7.56 7.55 7.54 7.54 7.54 7.46 7.46 7.45 7.44 7.44 7.43 7.42 7.42 7.42 7.41 7.26 CDCl3
4.50 4.48 4.46
2.94 2.92 2.90 2.87
0.00 TMS
$C_{20}H_{22}O_4S_2$
MW: 390.51
S16'
3.83 1.93 3.91 4.00 4.00 3.91
f1 (ppm)

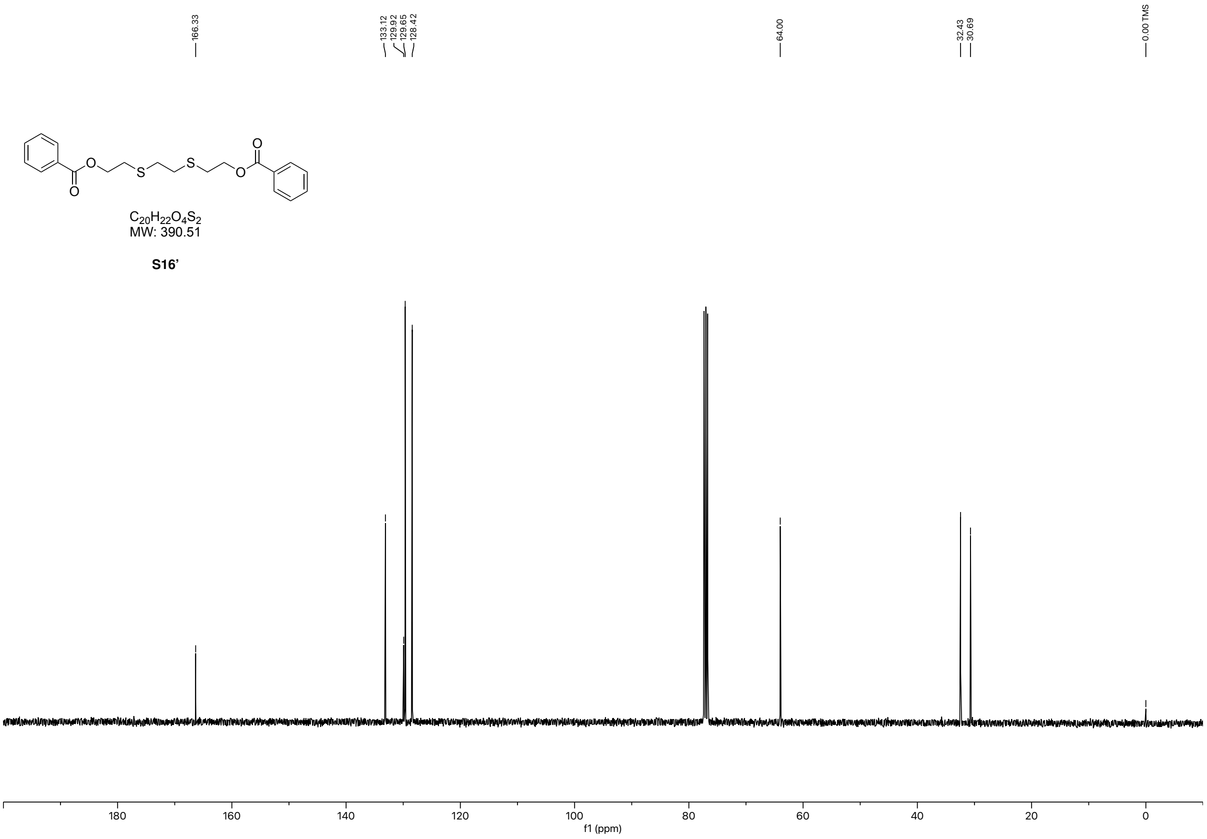

166.33
133.12 129.92 129.65 128.42
64.00
32.43 30.69
0.00 TMS
$C_{20}H_{22}O_4S_2$
MW: 390.51
S16'
f1 (ppm)

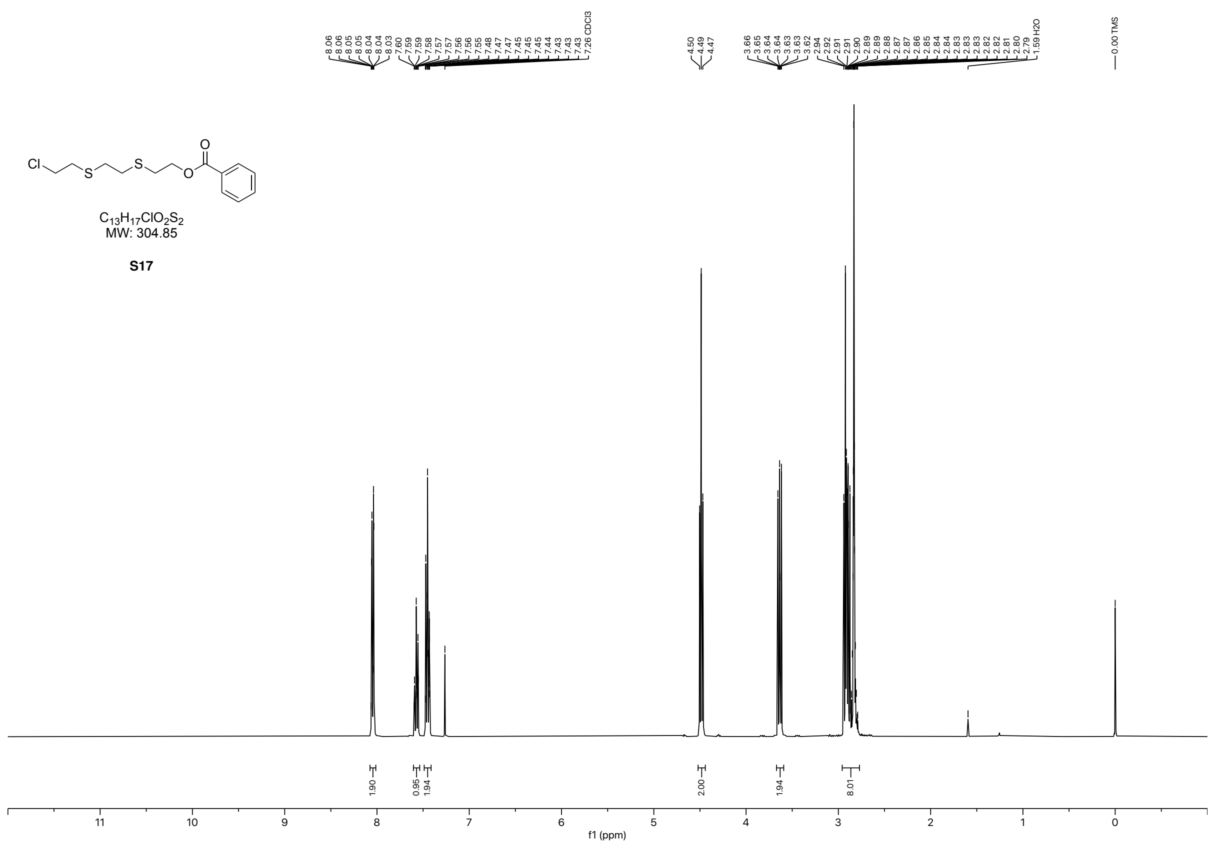

$C_{13}H_{17}ClO_2S_2$
MW: 304.85
S17
f1 (ppm)

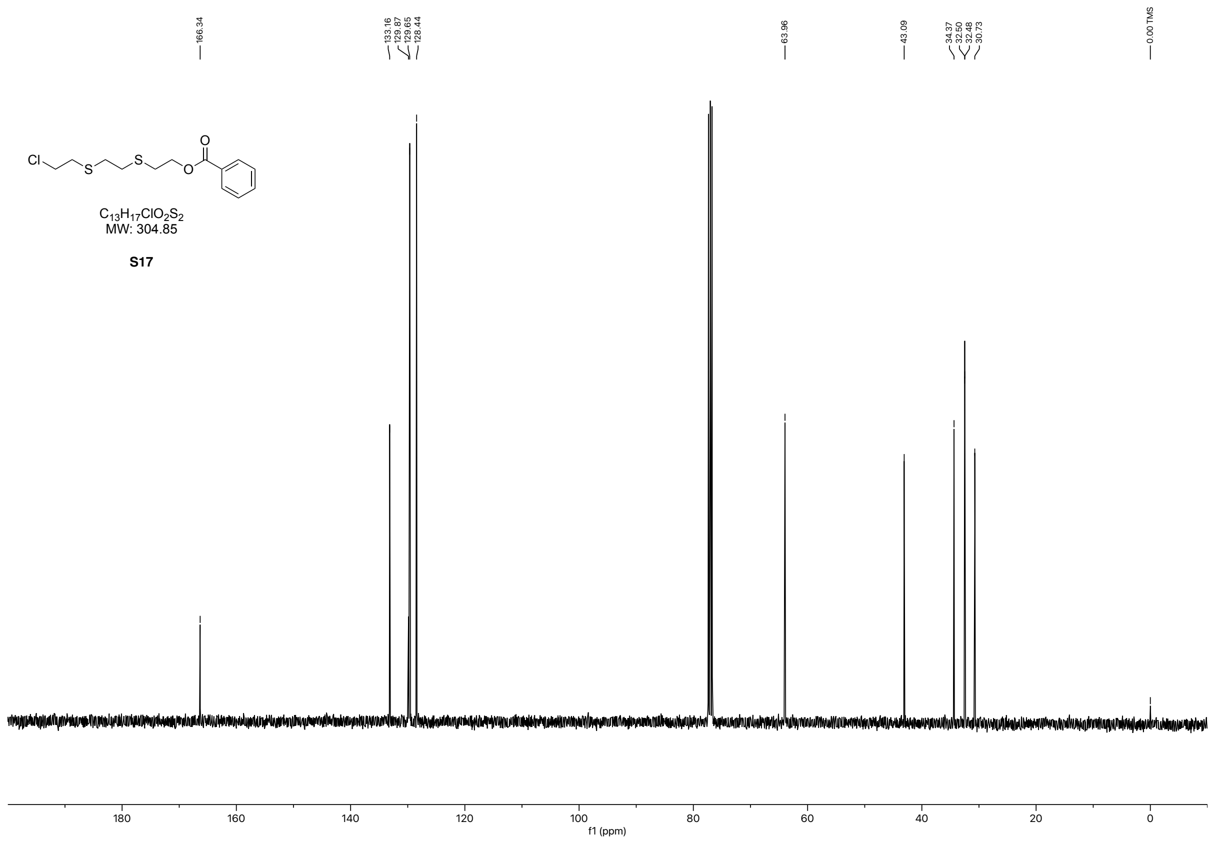

$C_{13}H_{17}ClO_2S_2$
MW: 304.85
S17
f1 (ppm)

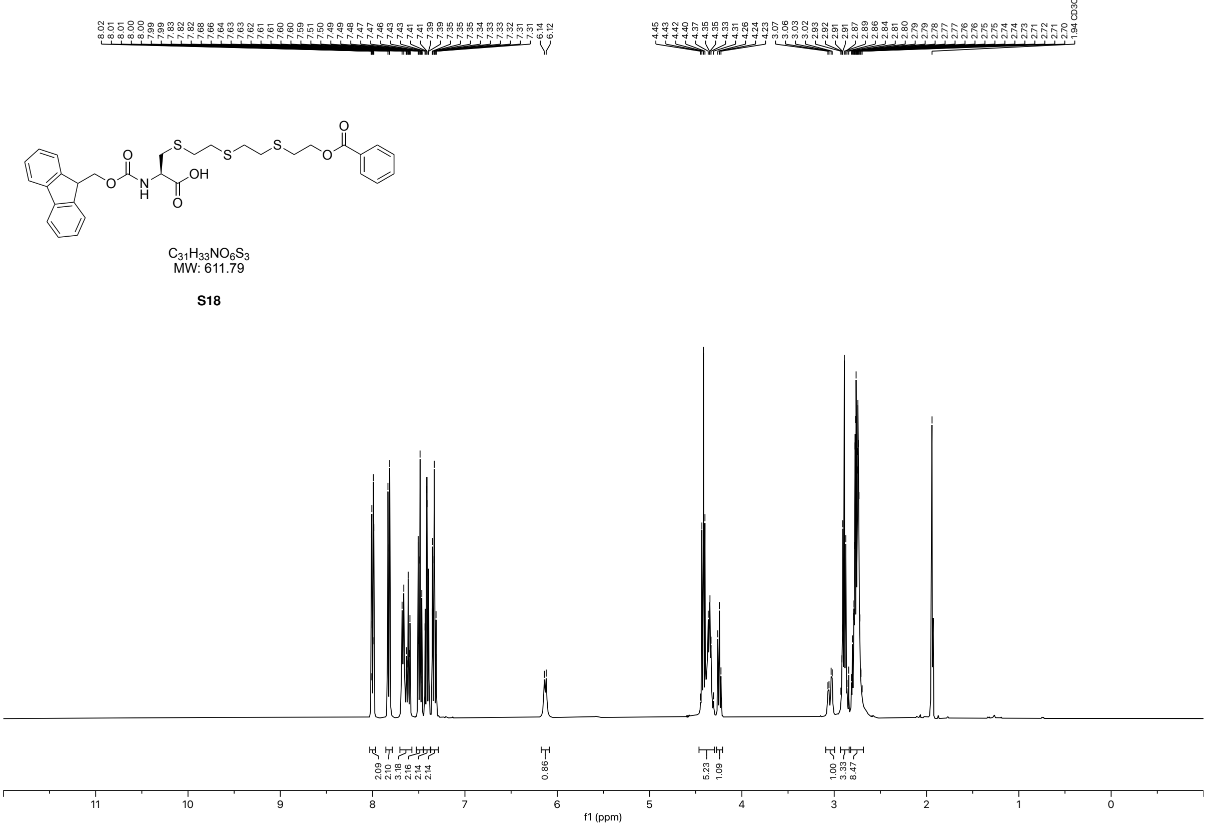
8.02 8.01 8.01 8.00 8.00 7.99 7.99 7.83 7.82 7.82 7.68 7.66 7.64 7.63 7.63 7.62 7.61 7.61 7.60 7.59 7.51 7.50 7.49 7.49 7.48 7.47 7.47 7.46 7.43 7.43 7.41 7.41 7.39 7.39 7.35 7.35 7.35 7.34 7.33 7.33 7.32 7.31 7.31 6.14 6.12
4.45 4.43 4.42 4.40 4.37 4.35 4.35 4.33 4.31 4.26 4.24 4.23 3.07 3.06 3.03 3.02 2.93 2.92 2.91 2.91 2.87 2.89 2.86 2.84 2.81 2.80 2.79 2.79 2.78 2.77 2.77 2.76 2.76 2.75 2.75 2.74 2.74 2.73 2.72 2.72 2.71 2.70 1.94 CD3CN
O
S
S
S
O
O
N
H
OH
O
O
O
$C_{31}H_{33}NO_6S_3$
MW: 611.79
S18
2.09 2.10 3.18 2.16 2.14 2.14
0.86
5.23 1.09
1.00 3.33 8.47
11 10 9 8 7 6 5 4 3 2 1 0
f1 (ppm)

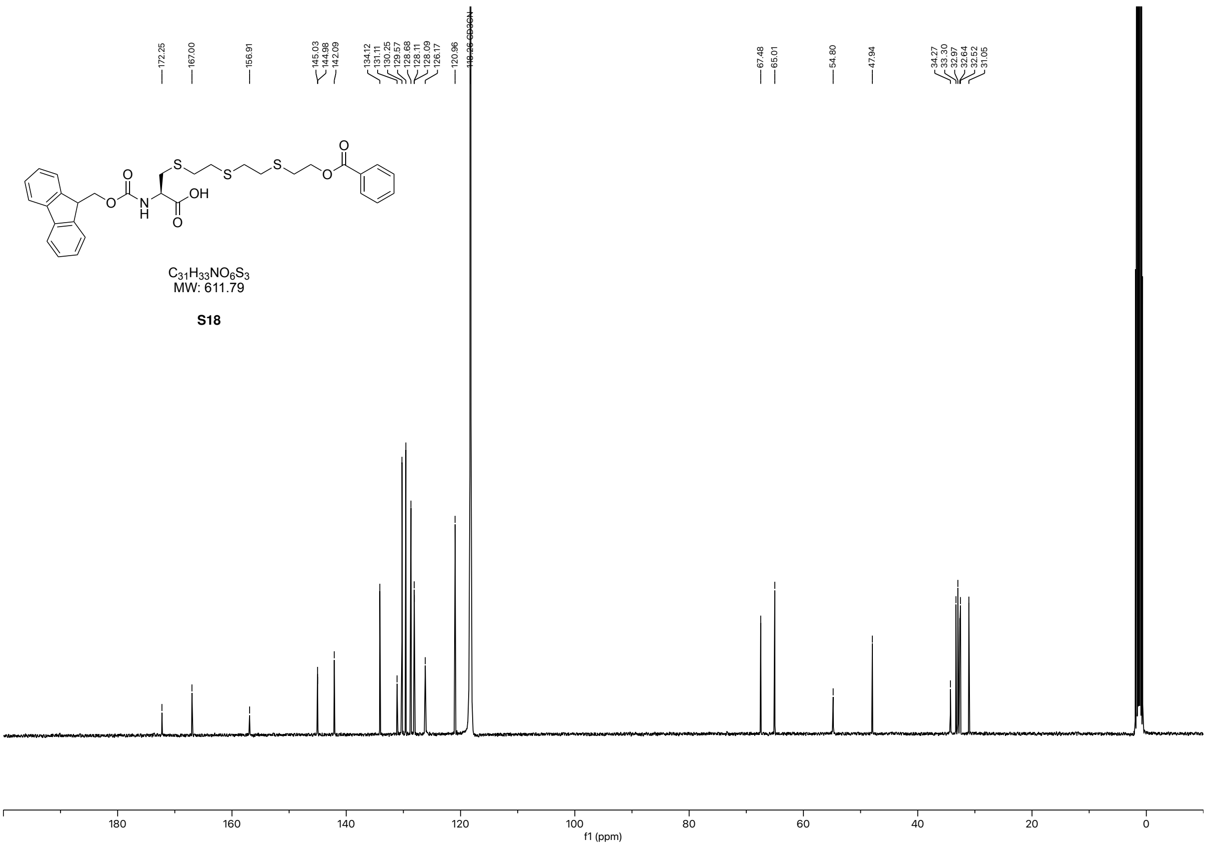
172.25
167.00
156.91
145.03 144.98 142.09
134.12 131.11 130.25 129.57 128.68 128.11 128.09 126.17
120.96
118.26 CD3CN
67.48 65.01
54.80
47.94
34.27 33.30 32.97 32.64 32.52 31.05
$C_{31}H_{33}NO_6S_3$
MW: 611.79
S18
180 160 140 120 100 80 60 40 20 0
f1 (ppm)

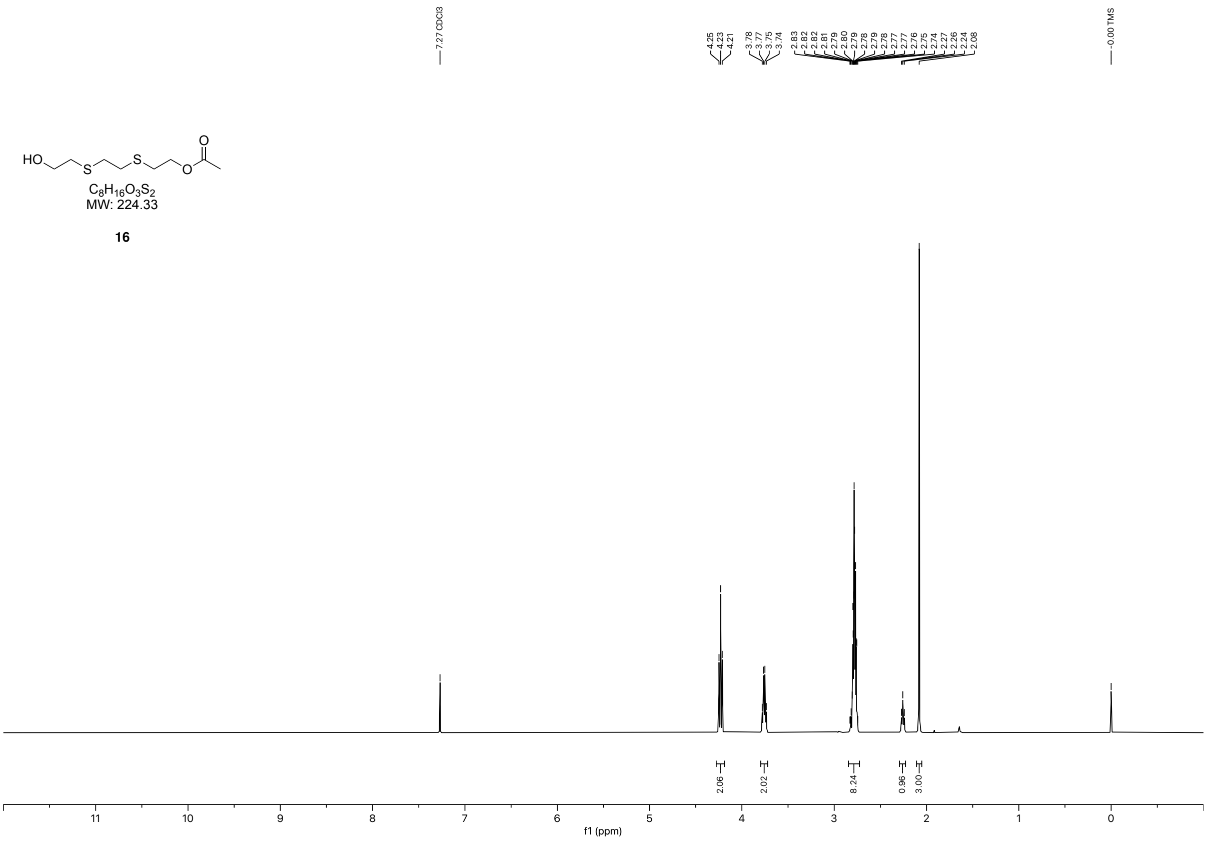
7.27 CDCl3
4.25
4.23
4.21
3.78
3.77
3.75
3.74
2.83
2.82
2.82
2.81
2.79
2.80
2.79
2.78
2.79
2.78
2.78
2.77
2.77
2.76
2.75
2.74
2.27
2.26
2.24
2.08
0.00 TMS
HO
S
S
O
O
C8H16O3S2
MW: 224.33
16
2.06
2.02
8.24
0.96
3.00
f1 (ppm)

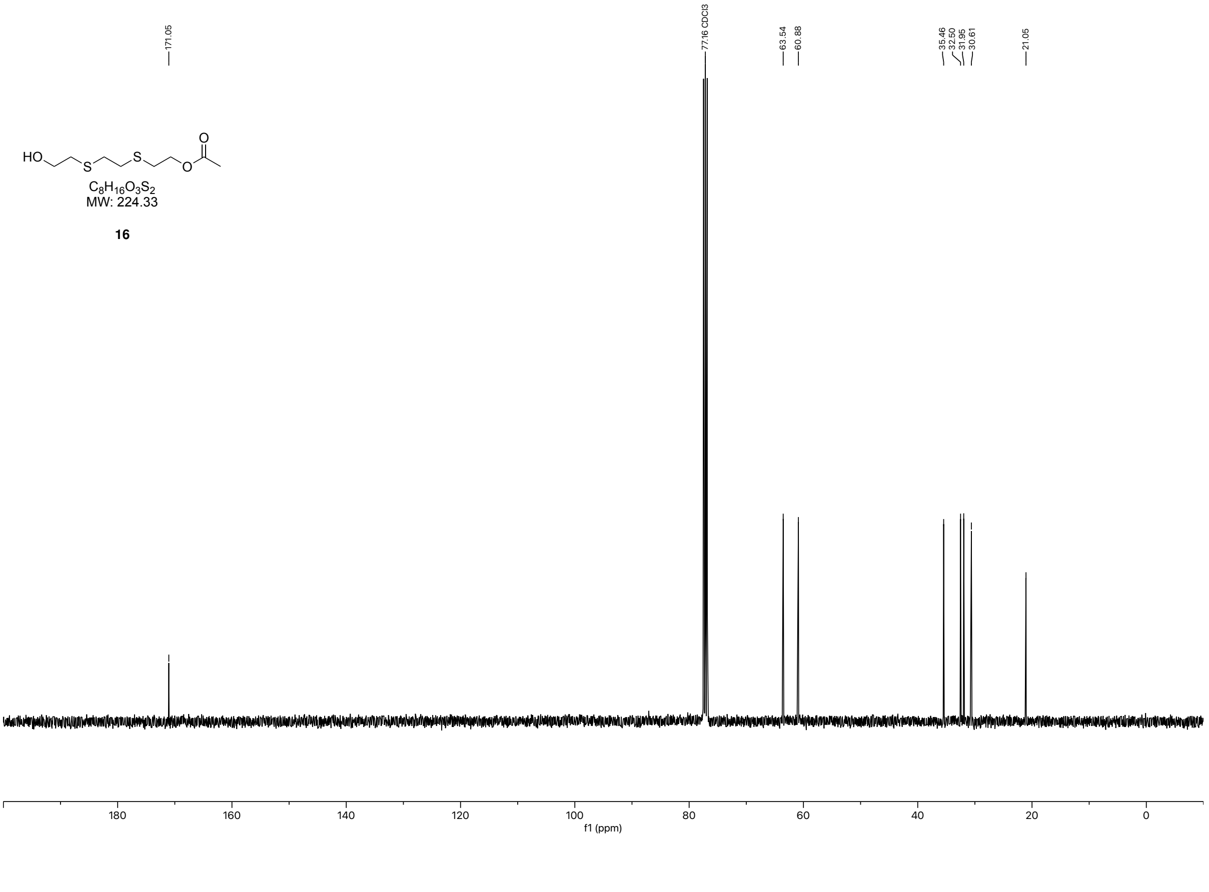
171.05
77.16 CDCl3
63.54
60.88
35.46
32.50
31.95
30.61
21.05
HO
S
S
O
O
C8H16O3S2
MW: 224.33
16
f1 (ppm)

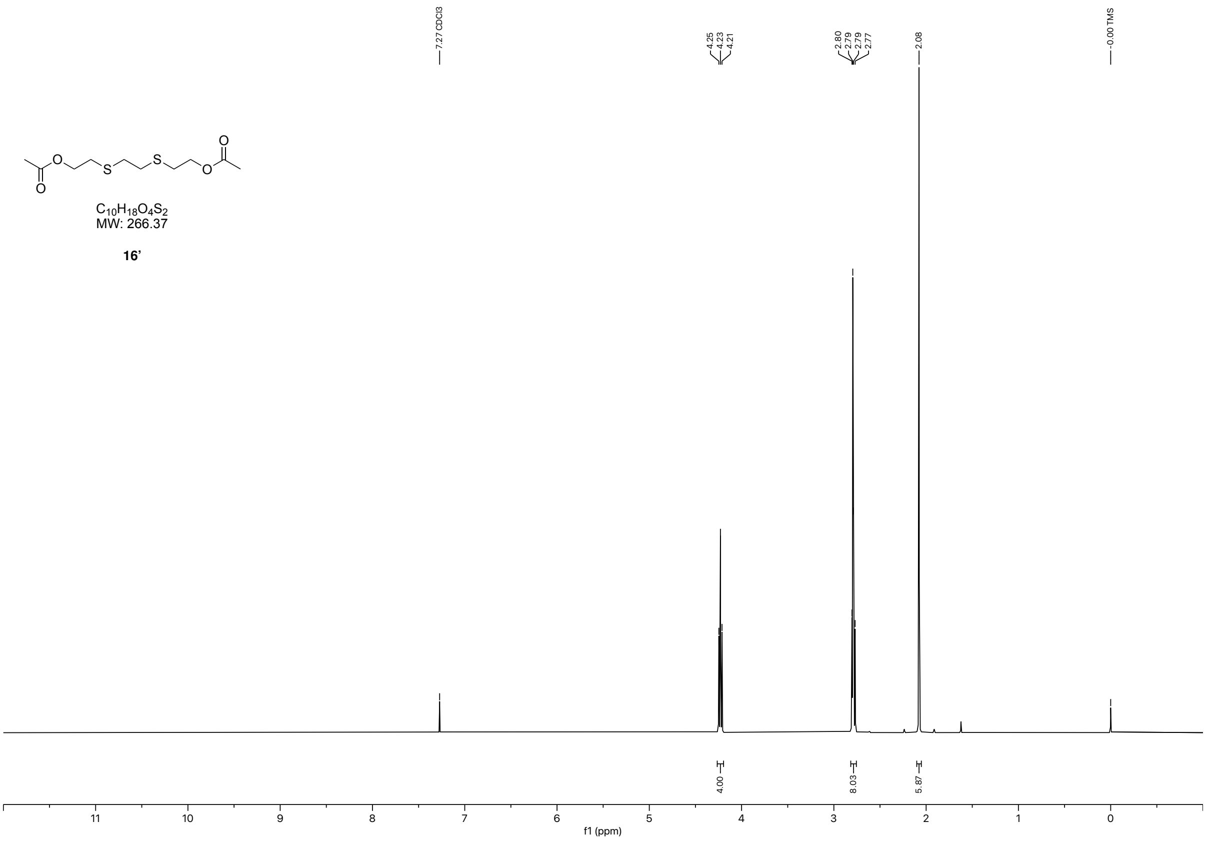

$C_{10}H_{18}O_4S_2$
MW: 266.37

16'

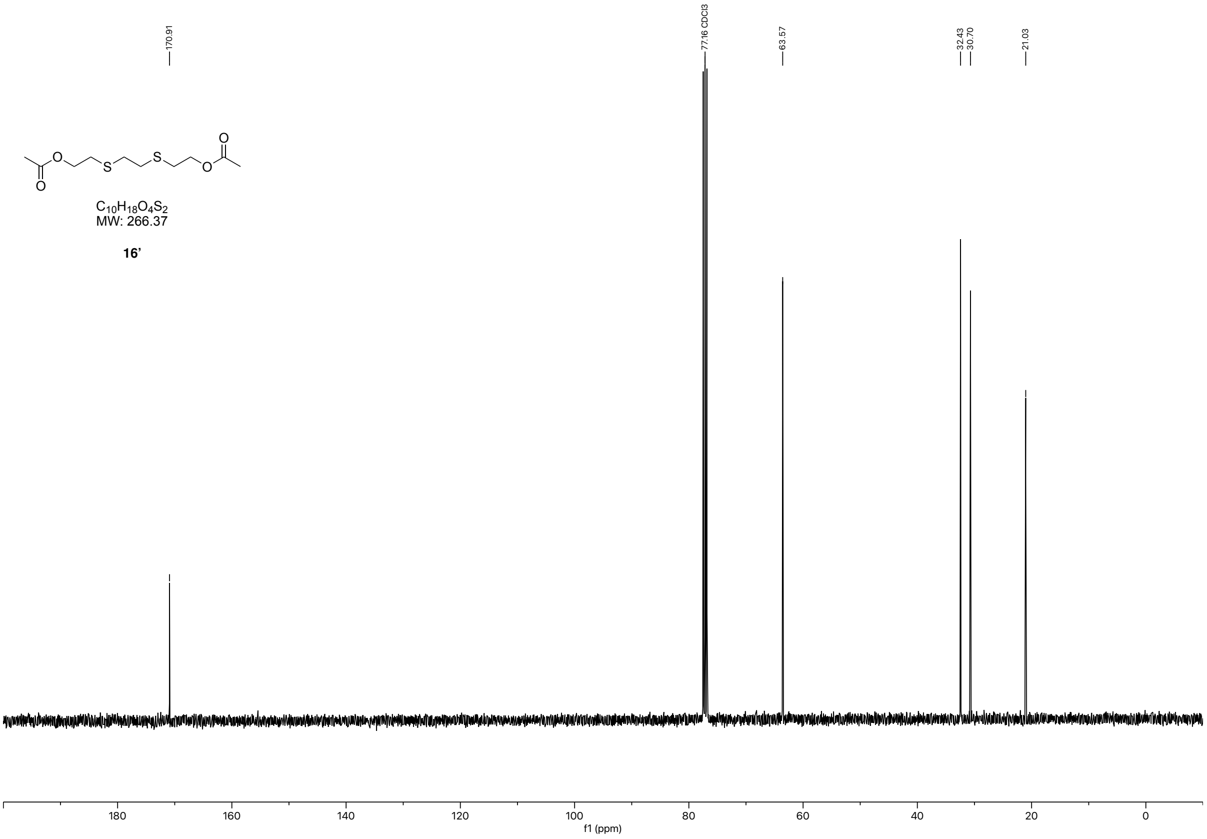

$C_{10}H_{18}O_4S_2$
MW: 266.37

16'

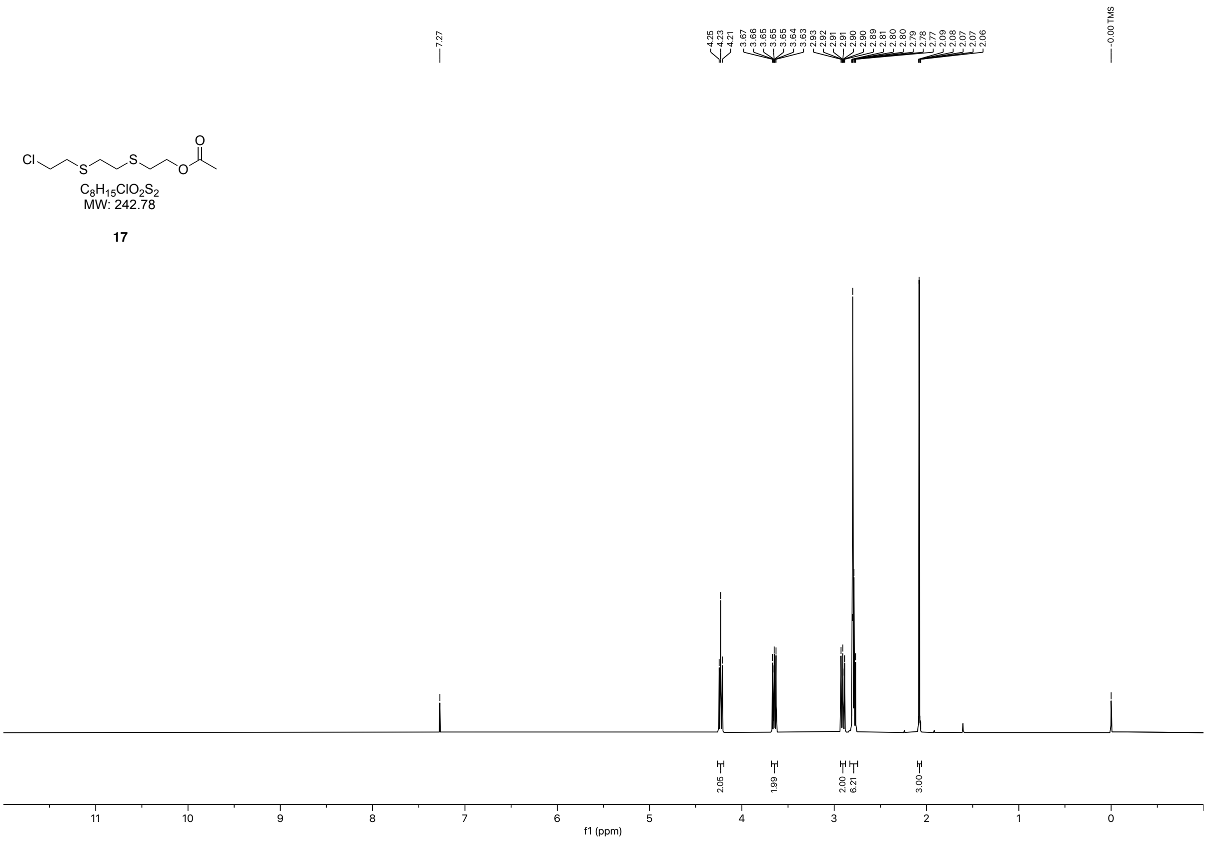
7.27
0.00 TMS
Cl
S
S
O
O
$C_8H_{15}ClO_2S_2$
MW: 242.78
17
2.05
1.99
2.00
6.21
3.00
f1 (ppm)

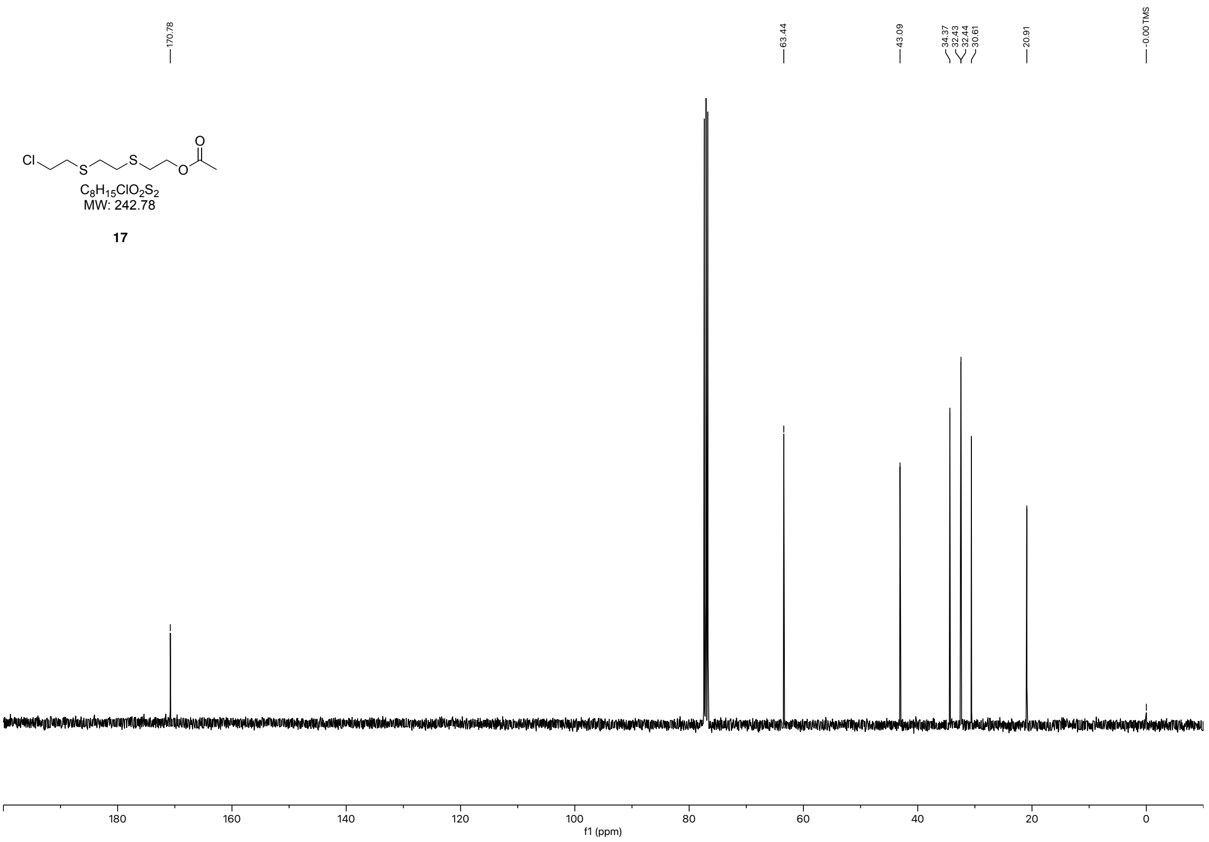
170.78
63.44
43.09
34.37
32.43
32.44
30.61
20.91
0.00 TMS
Cl
S
S
O
O
$C_8H_{15}ClO_2S_2$
MW: 242.78
17
f1 (ppm)

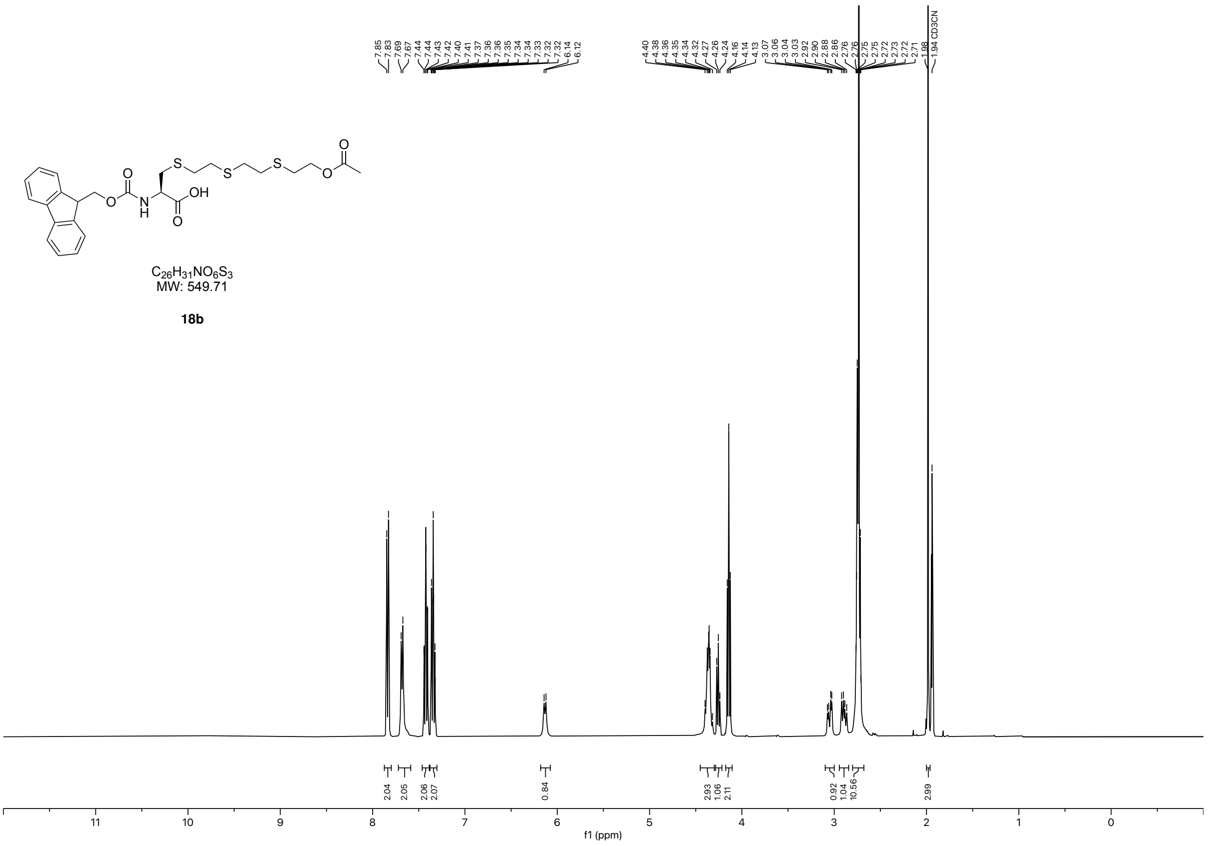
7.85
7.83
7.69
7.67
7.44
7.44
7.43
7.42
7.40
7.41
7.37
7.36
7.36
7.35
7.34
7.34
7.33
7.32
7.32
6.14
6.12
4.40
4.38
4.36
4.35
4.34
4.32
4.27
4.26
4.24
4.16
4.14
4.13
3.07
3.06
3.04
3.03
2.92
2.90
2.88
2.86
2.76
2.76
2.75
2.75
2.73
2.72
2.72
2.71
1.98
1.94 CD3CN
O
O
N
H
S
S
S
O
O
OH
O
$C_{26}H_{31}NO_6S_3$
MW: 549.71
18b
2.04
2.05
2.06
2.07
0.84
2.93
1.06
2.11
0.92
1.04
10.56
2.99
11
10
9
8
7
6
5
4
3
2
1
0
f1 (ppm)

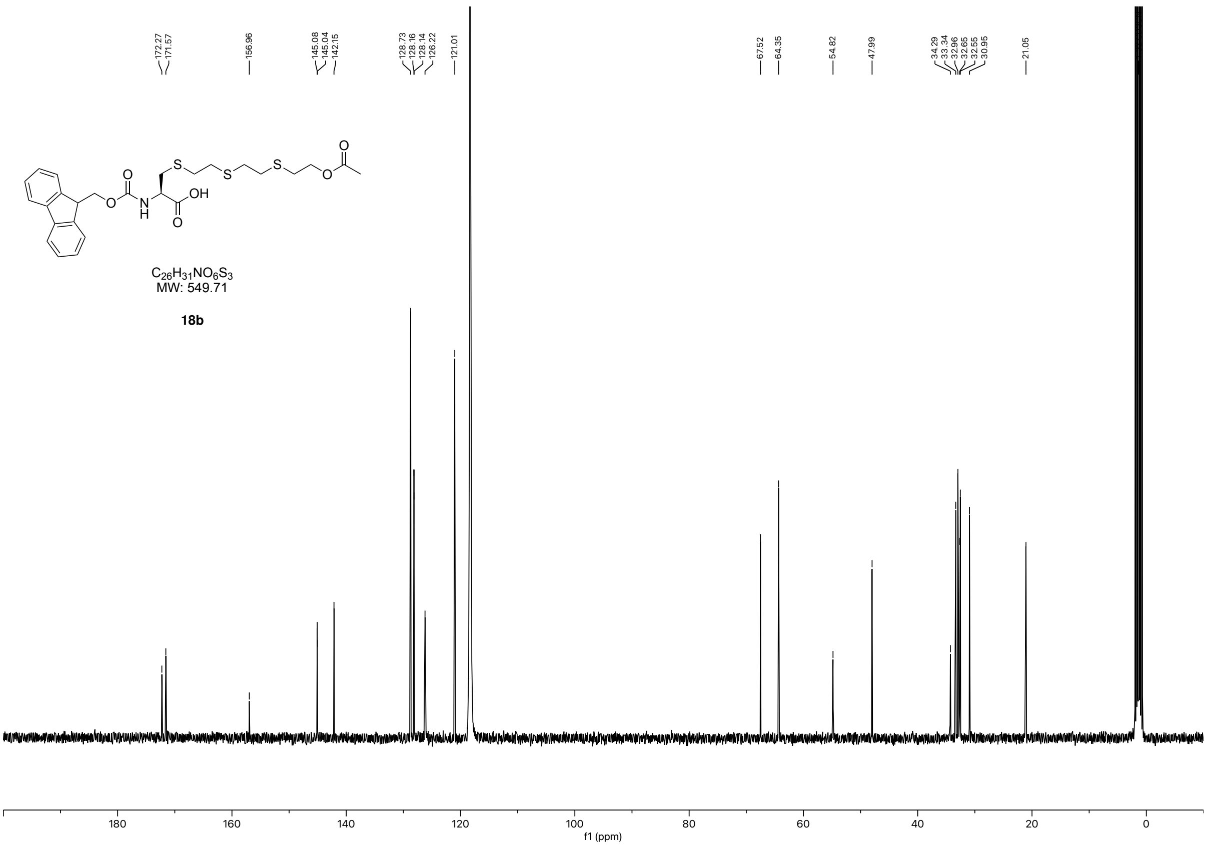
172.27
171.57
156.96
145.08
145.04
142.15
128.73
128.16
128.14
126.22
121.01
67.52
64.35
54.82
47.99
34.29
33.34
32.96
32.65
32.55
30.95
21.05
O
O
N
H
S
S
S
O
O
OH
O
$C_{26}H_{31}NO_6S_3$
MW: 549.71
18b
180
160
140
120
100
80
60
40
20
0
f1 (ppm)

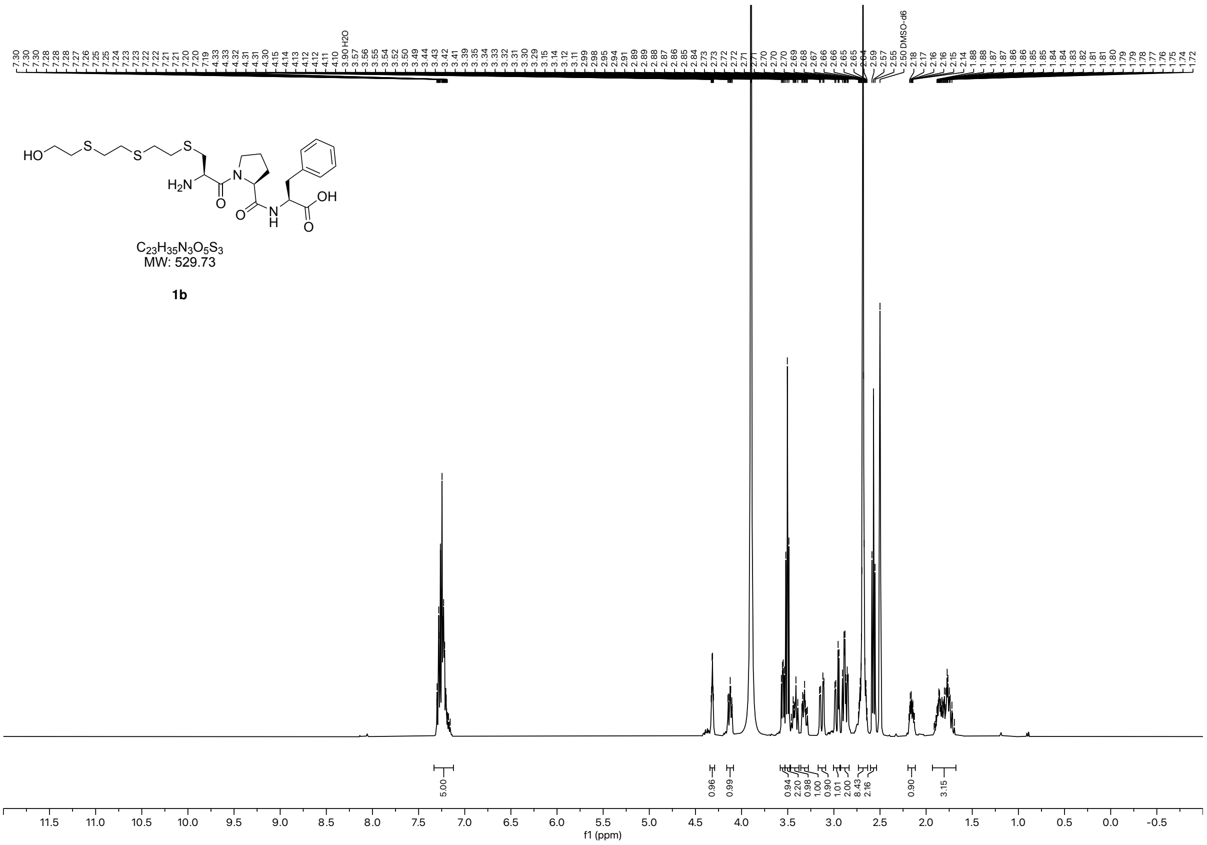
HO
S
S
S
H_2N
O
N
O
N
H
OH
O
$C_{23}H_{35}N_3O_5S_3$
MW: 529.73
1b
3.90 H2O
2.50 DMSO-d6
f1 (ppm)

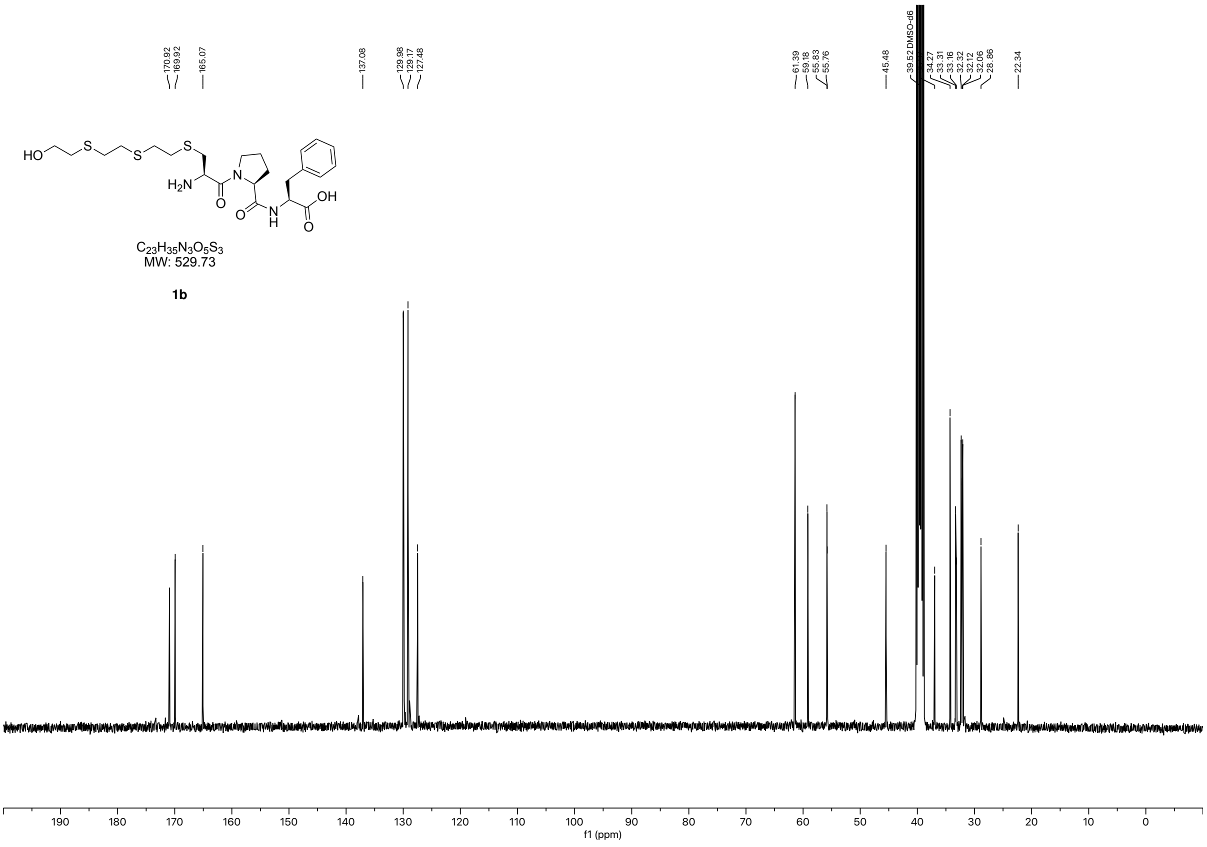
170.92
169.92
165.07
137.08
129.98
129.17
127.48
61.39
59.18
55.83
55.76
45.48
39.52 DMSO-d6
34.27
33.31
33.16
32.32
32.12
32.06
28.86
22.34
HO
S
S
S
H_2N
O
N
O
N
H
OH
O
$C_{23}H_{35}N_3O_5S_3$
MW: 529.73
1b
f1 (ppm)

$C_6H_{12}O_3S$
MW: 164.22

S19

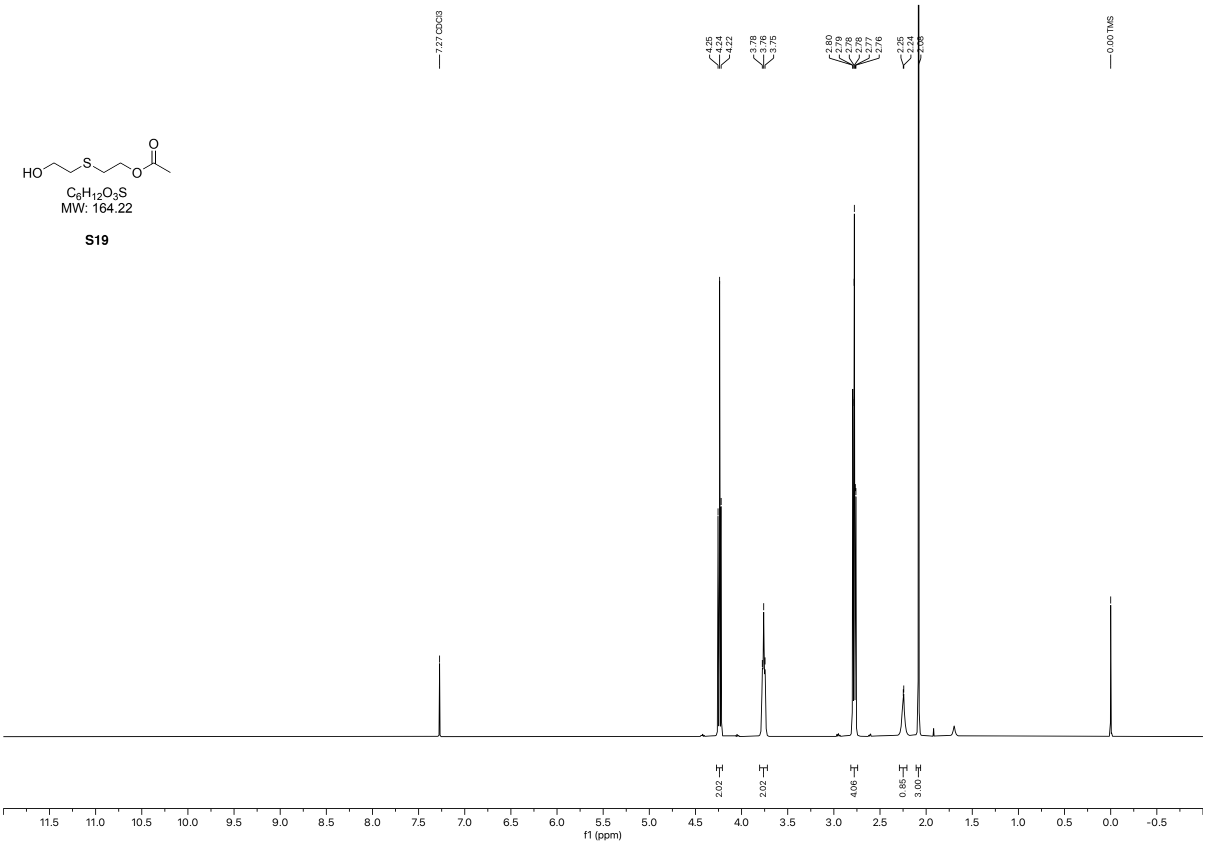

$C_6H_{12}O_3S$
MW: 164.22

S19

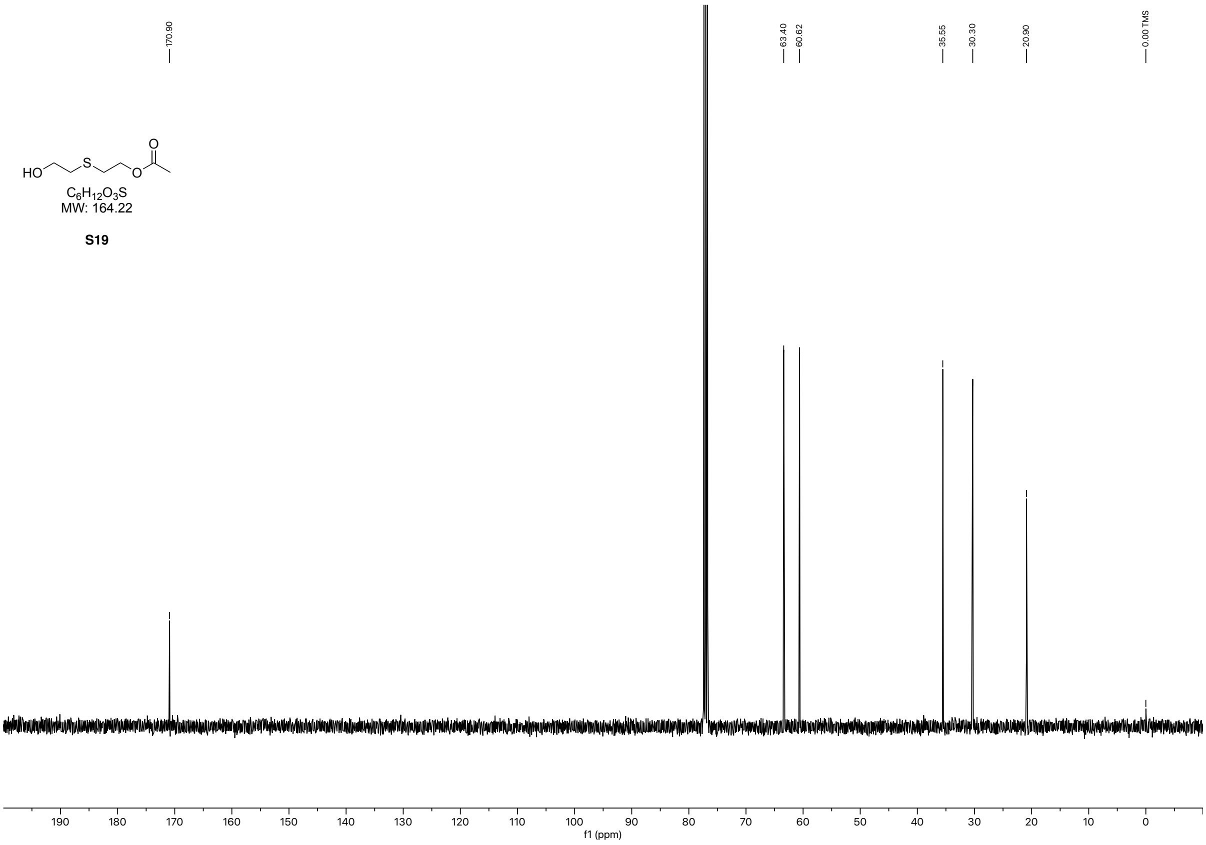

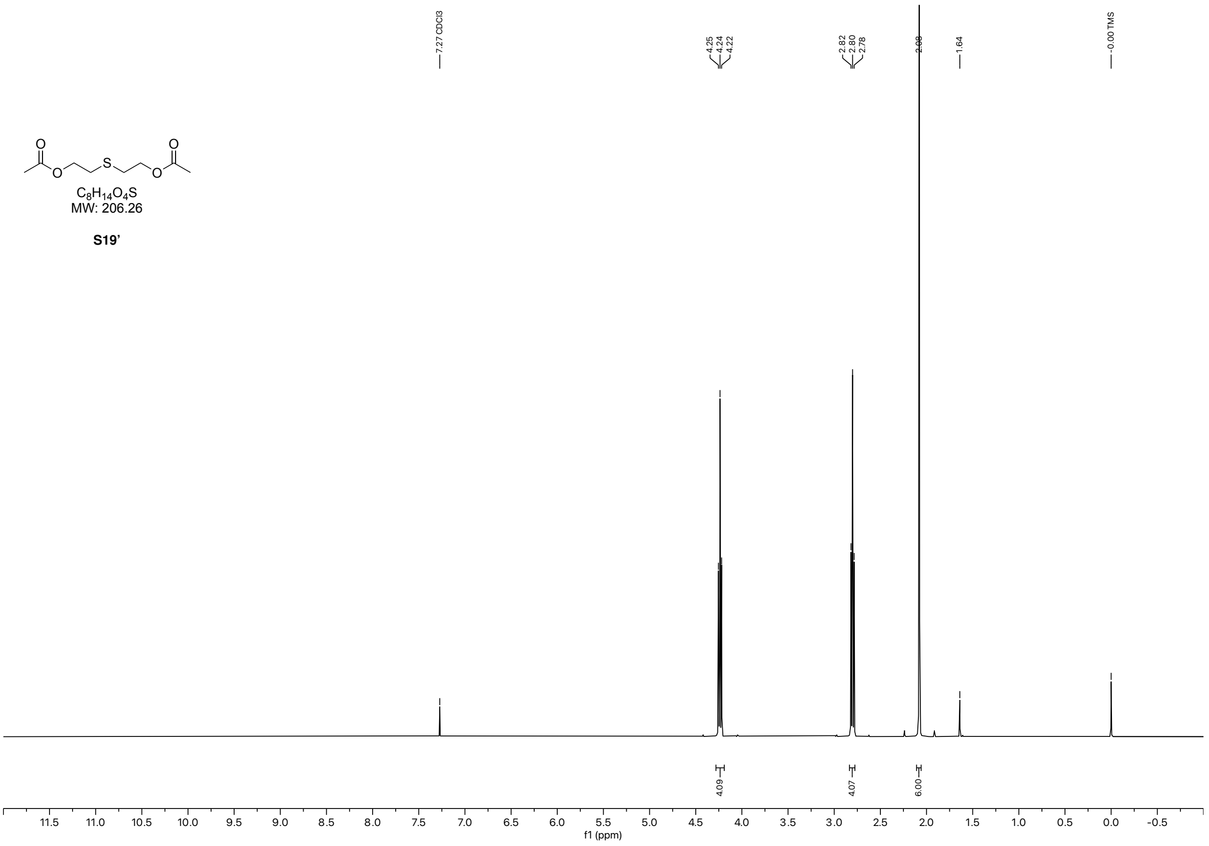
7.27 CDCl3
4.25
4.24
4.22
2.82
2.80
2.78
2.08
1.64
0.00 TMS
$C_8H_{14}O_4S$
MW: 206.26
S19'
4.09
4.07
6.00
11.5 11.0 10.5 10.0 9.5 9.0 8.5 8.0 7.5 7.0 6.5 6.0 5.5 5.0 4.5 4.0 3.5 3.0 2.5 2.0 1.5 1.0 0.5 0.0 -0.5
f1 (ppm)

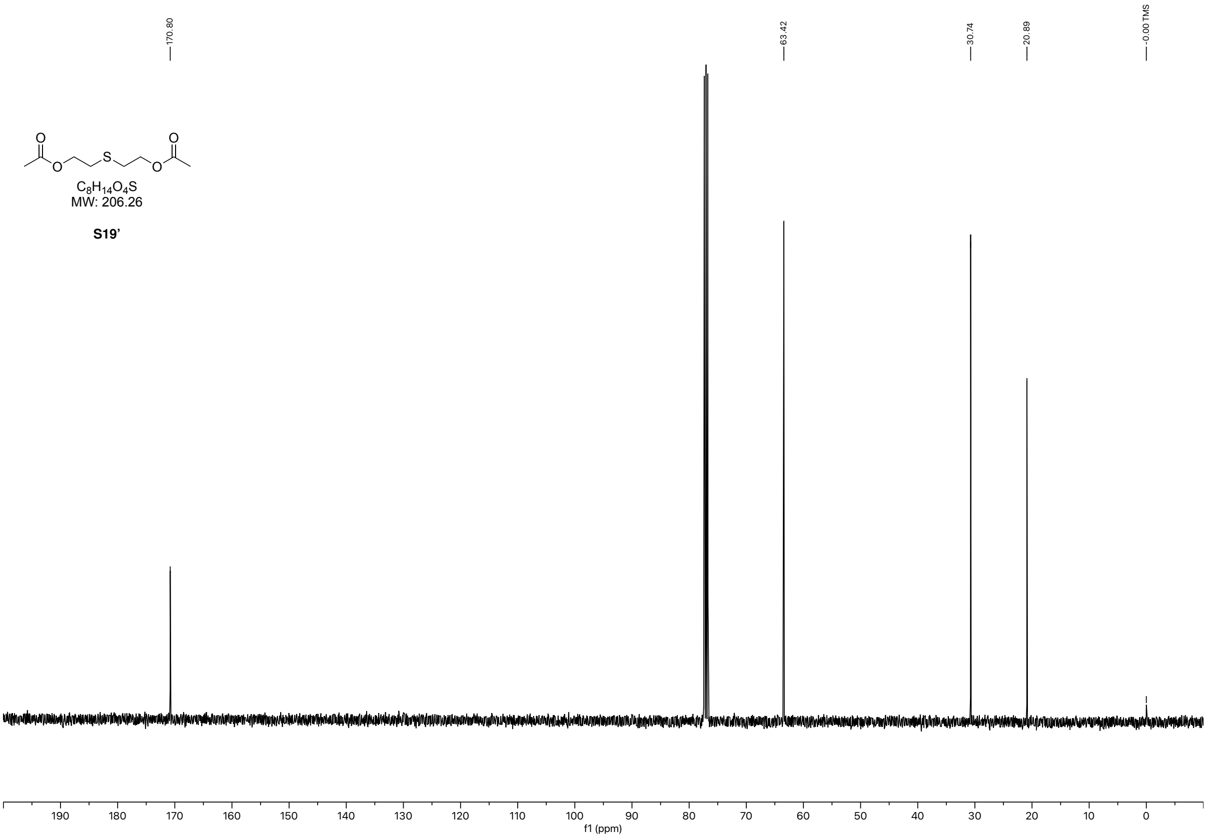
170.80
63.42
30.74
20.89
0.00 TMS
$C_8H_{14}O_4S$
MW: 206.26
S19'
190 180 170 160 150 140 130 120 110 100 90 80 70 60 50 40 30 20 10 0
f1 (ppm)

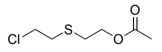

$C_6H_{11}ClO_2S$
MW: 182.66

S20

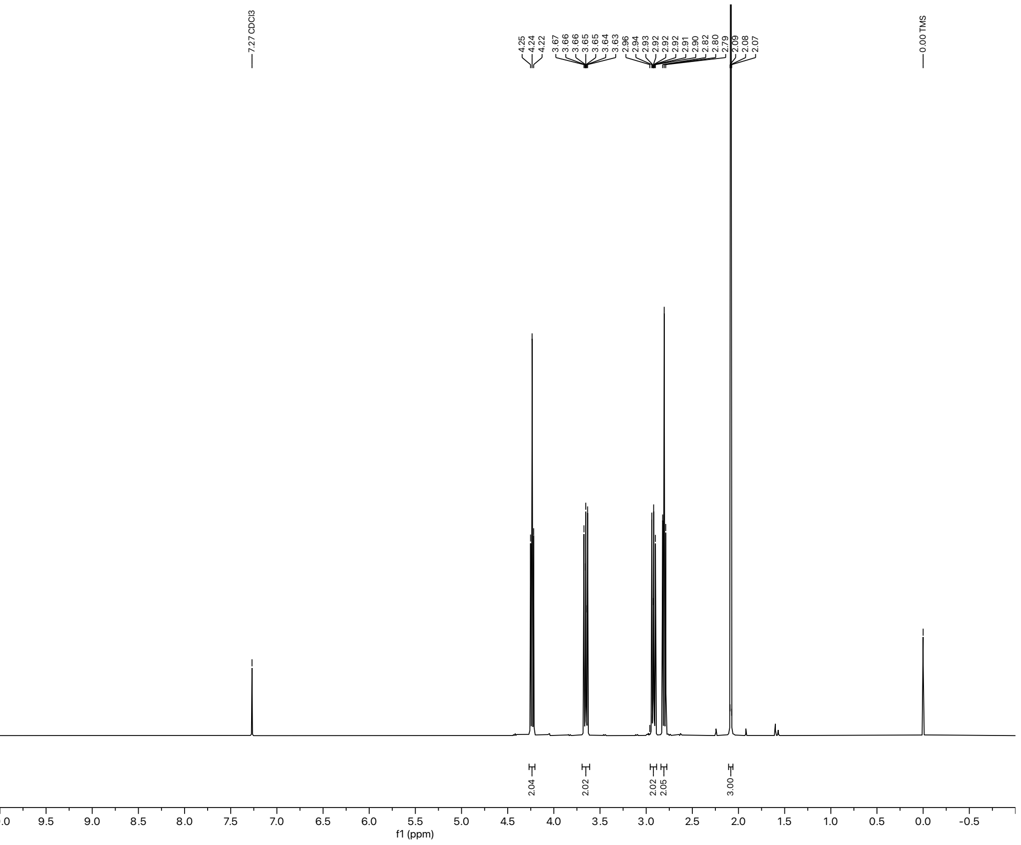

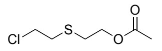

$C_6H_{11}ClO_2S$
MW: 182.66

S20

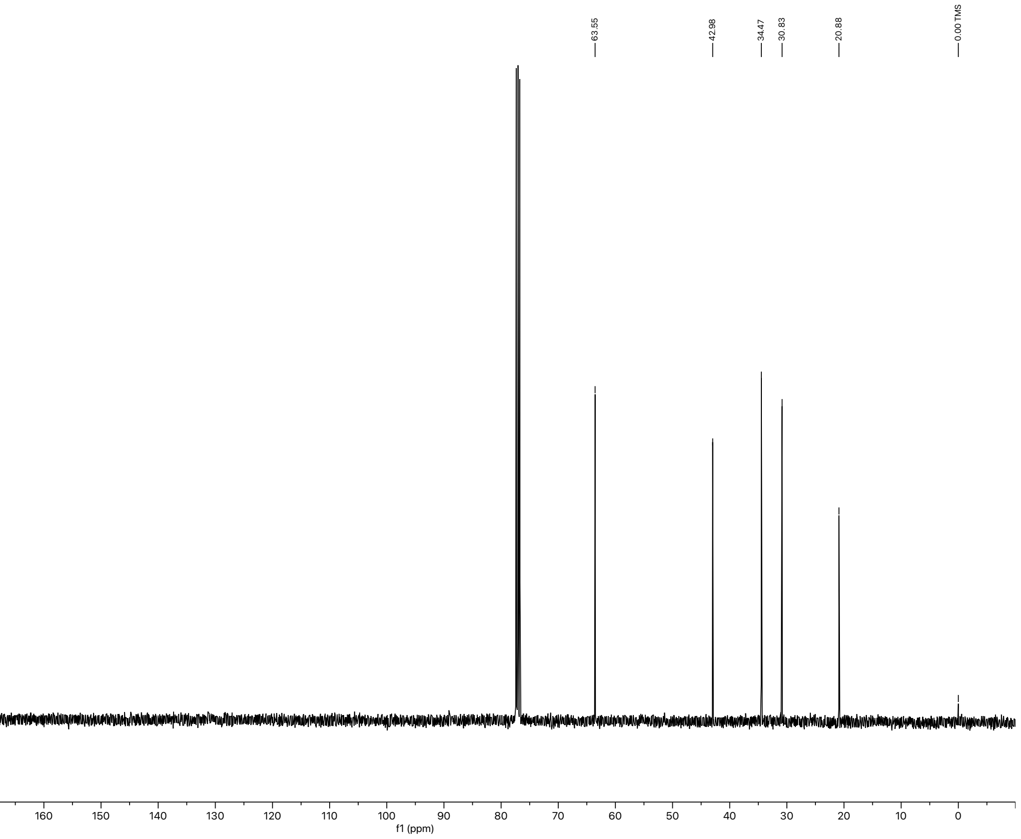

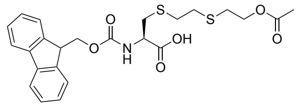

$C_{24}H_{27}NO_6S_2$
MW: 489.60

18a

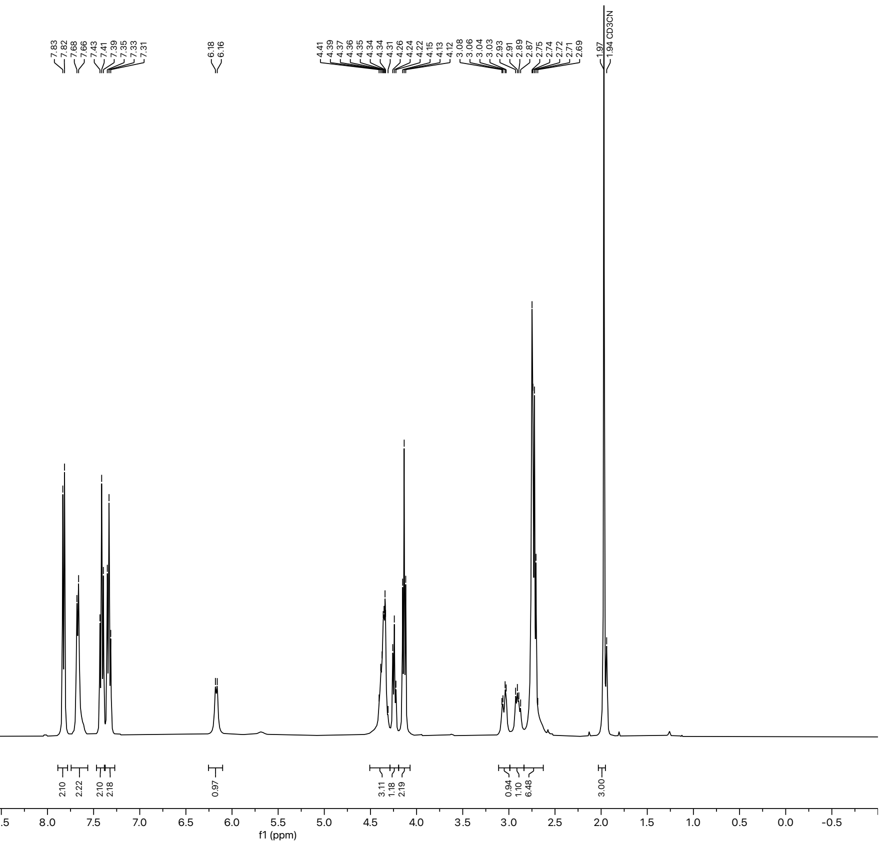

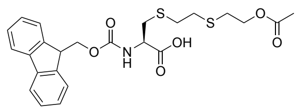

$C_{24}H_{27}NO_6S_2$
MW: 489.60

18a

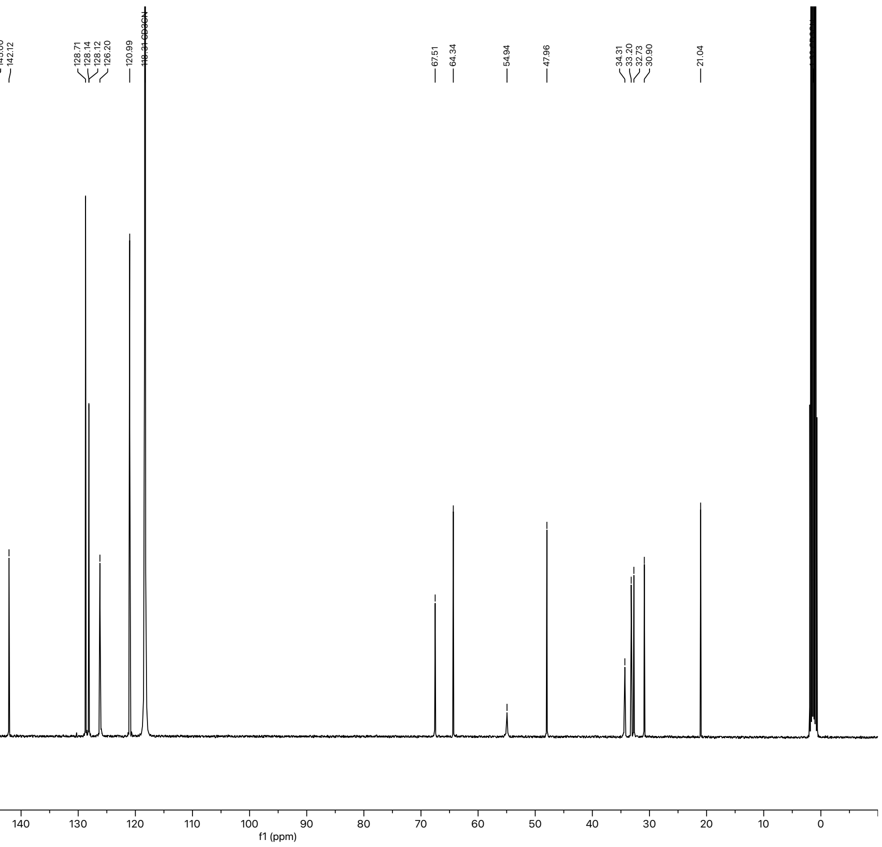

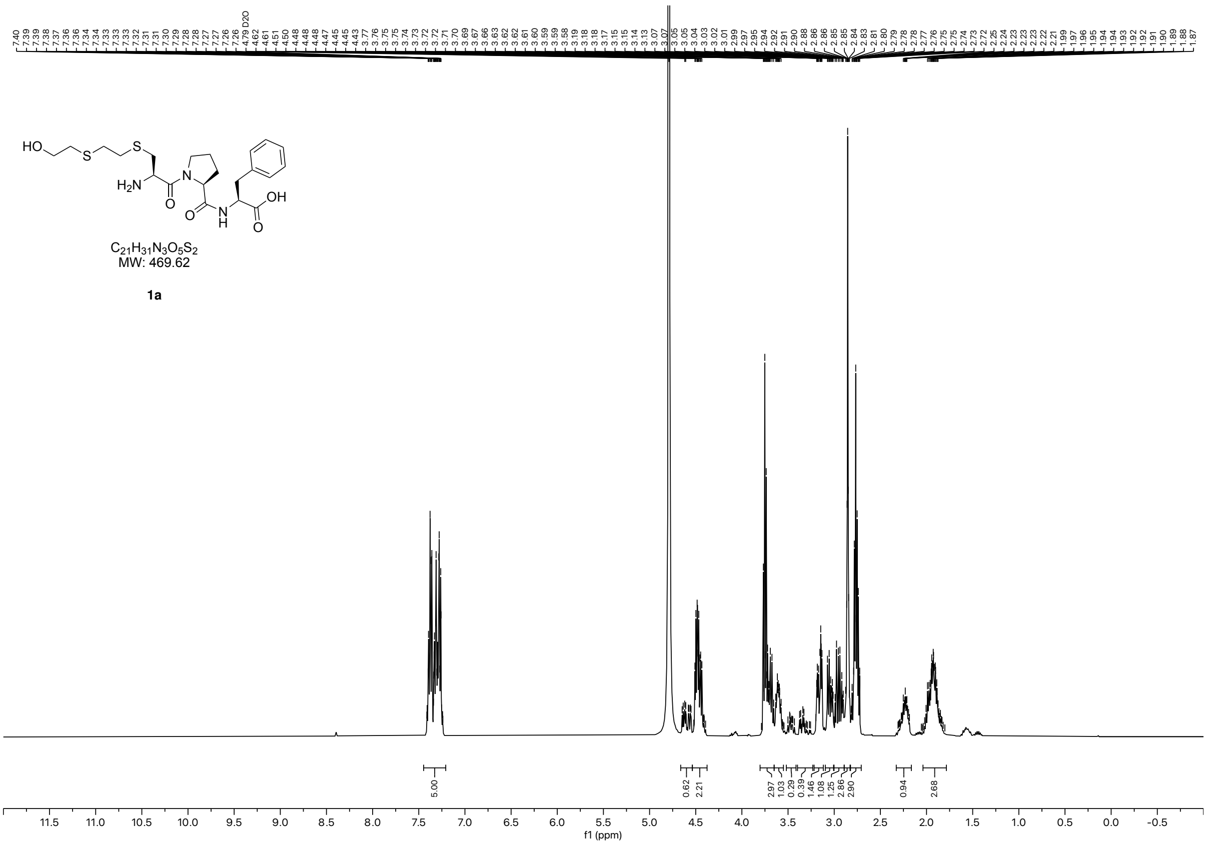

HO
S
S
H_2N
N
O
O
N
H
OH
O
$C_{21}H_{31}N_3O_5S_2$
MW: 469.62
1a
5.00
0.62
2.21
2.97
1.03
0.29
0.39
1.46
1.08
1.25
2.86
2.90
0.94
2.68
11.5 11.0 10.5 10.0 9.5 9.0 8.5 8.0 7.5 7.0 6.5 6.0 5.5 5.0 4.5 4.0 3.5 3.0 2.5 2.0 1.5 1.0 0.5 0.0 -0.5
f1 (ppm)

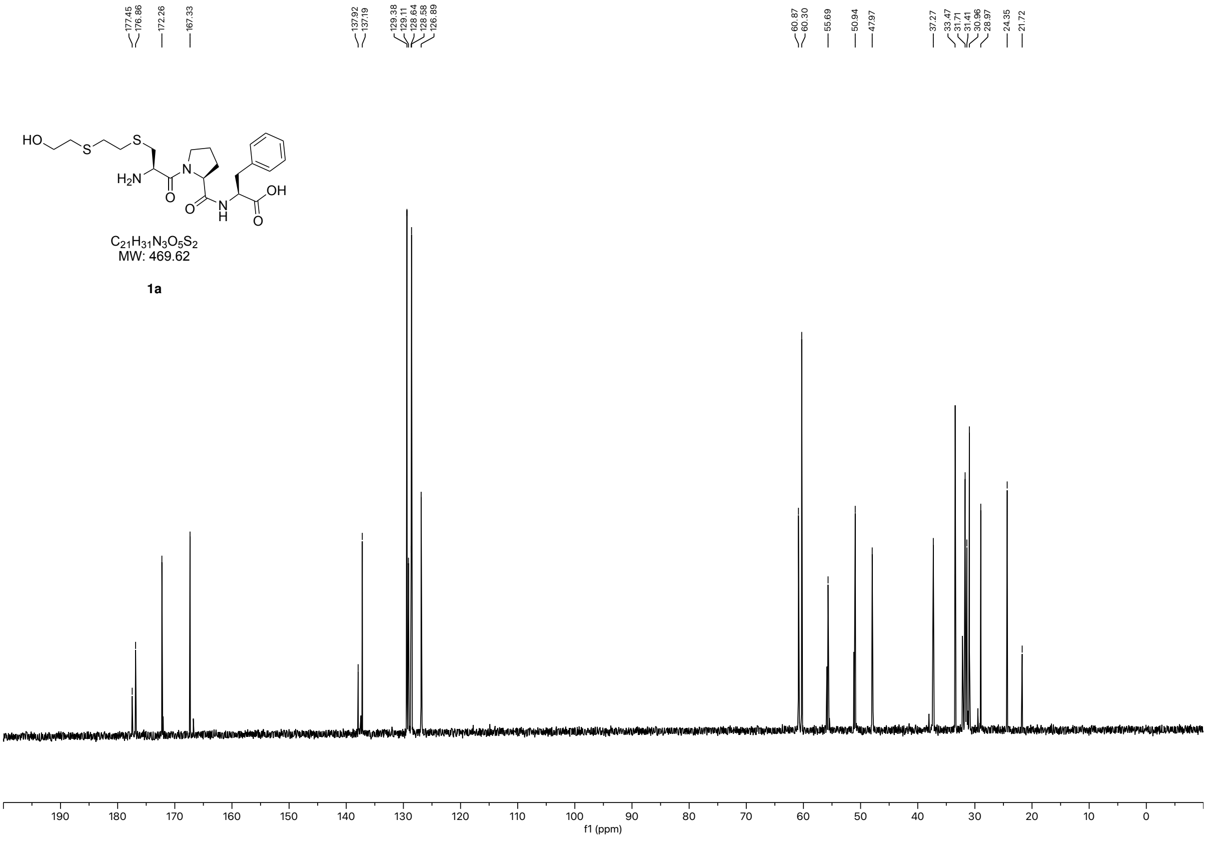

177.45
176.86
172.26
167.33
137.92
137.19
129.38
129.11
128.64
128.58
126.89
60.87
60.30
55.69
50.94
47.97
37.27
33.47
31.71
31.41
30.96
28.97
24.35
21.72
HO
S
S
H_2N
N
O
O
N
H
OH
O
$C_{21}H_{31}N_3O_5S_2$
MW: 469.62
1a
190 180 170 160 150 140 130 120 110 100 90 80 70 60 50 40 30 20 10 0
f1 (ppm)

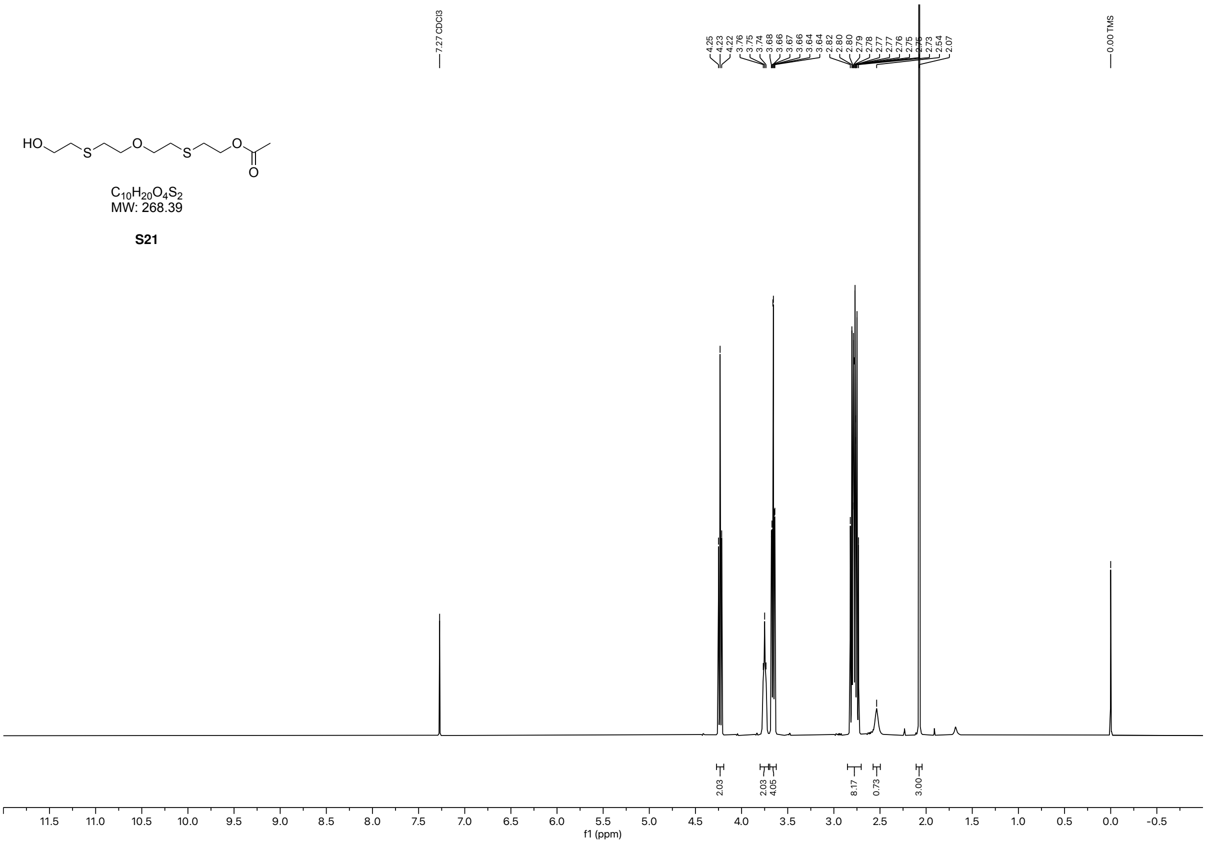
7.27 CDCl3
4.25
4.23
4.22
3.76
3.75
3.74
3.68
3.66
3.67
3.66
3.64
3.64
2.82
2.80
2.80
2.79
2.78
2.77
2.77
2.76
2.75
2.73
2.54
2.07
0.00 TMS
HO S O S O O
$C_{10}H_{20}O_4S_2$
MW: 268.39
S21
2.03
2.03
4.05
8.17
0.73
3.00
11.5 11.0 10.5 10.0 9.5 9.0 8.5 8.0 7.5 7.0 6.5 6.0 5.5 5.0 4.5 4.0 3.5 3.0 2.5 2.0 1.5 1.0 0.5 0.0 -0.5
f1 (ppm)

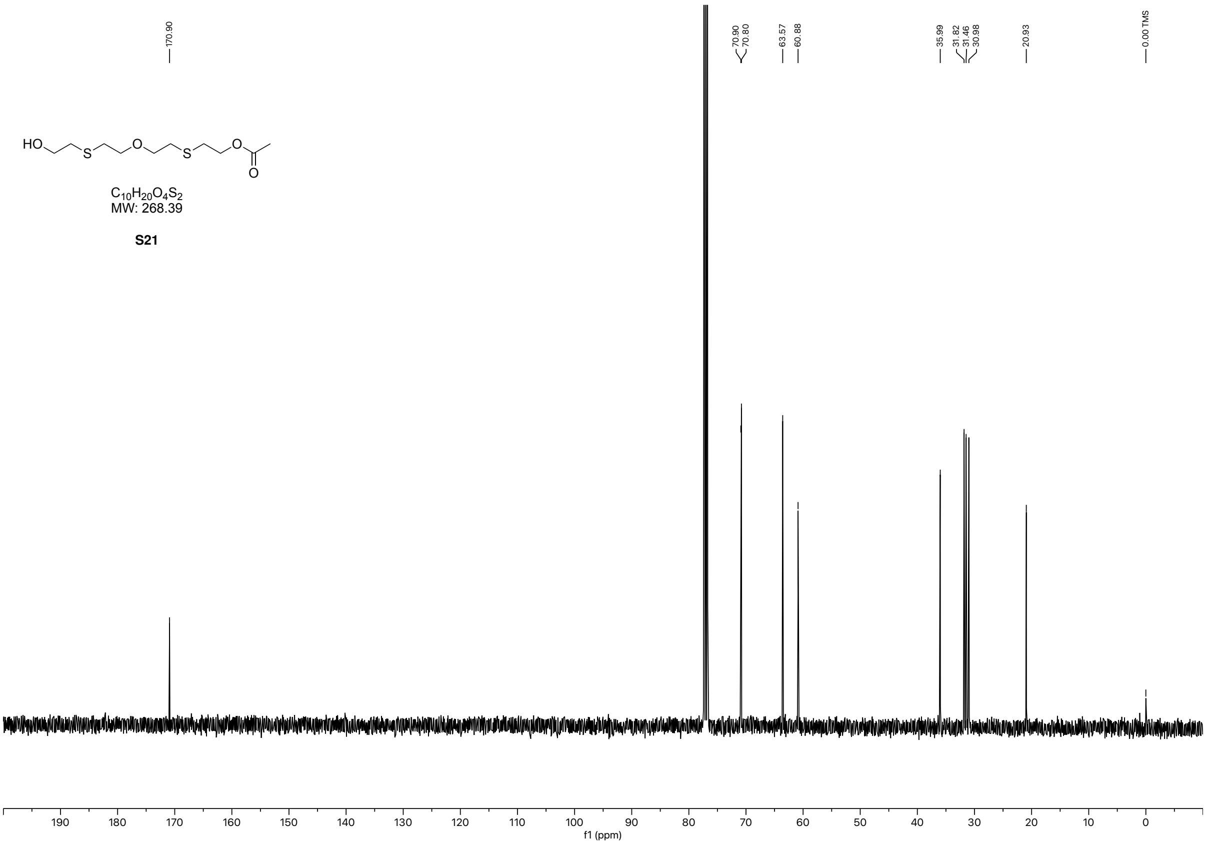
170.90
70.90
70.80
63.57
60.88
35.99
31.82
31.46
30.98
20.93
0.00 TMS
HO S O S O O
$C_{10}H_{20}O_4S_2$
MW: 268.39
S21
190 180 170 160 150 140 130 120 110 100 90 80 70 60 50 40 30 20 10 0
f1 (ppm)

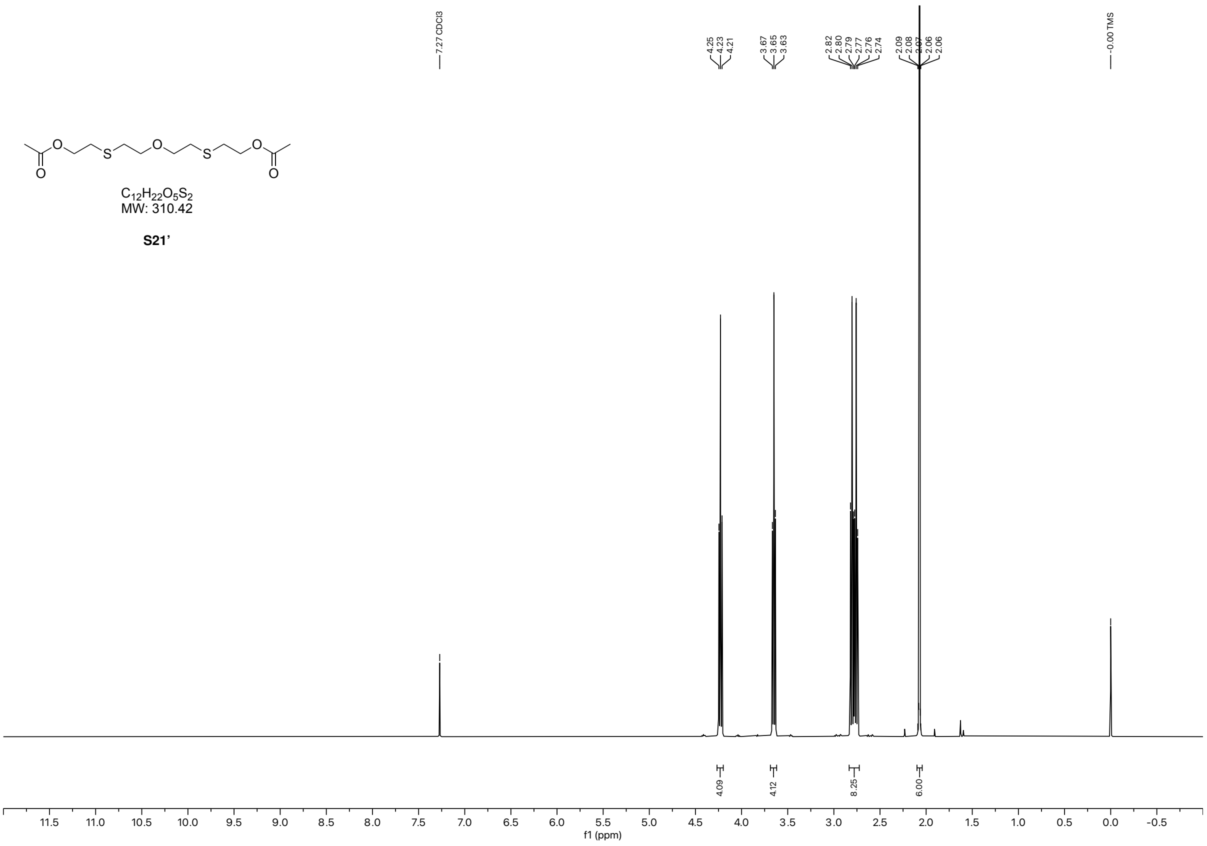

7.27 CDCl3
4.25
4.23
4.21
3.67
3.65
3.63
2.82
2.80
2.79
2.77
2.76
2.74
2.09
2.08
2.06
2.06
0.00 TMS
$C_{12}H_{22}O_5S_2$
MW: 310.42
S21'
4.09
4.12
8.25
6.00
f1 (ppm)

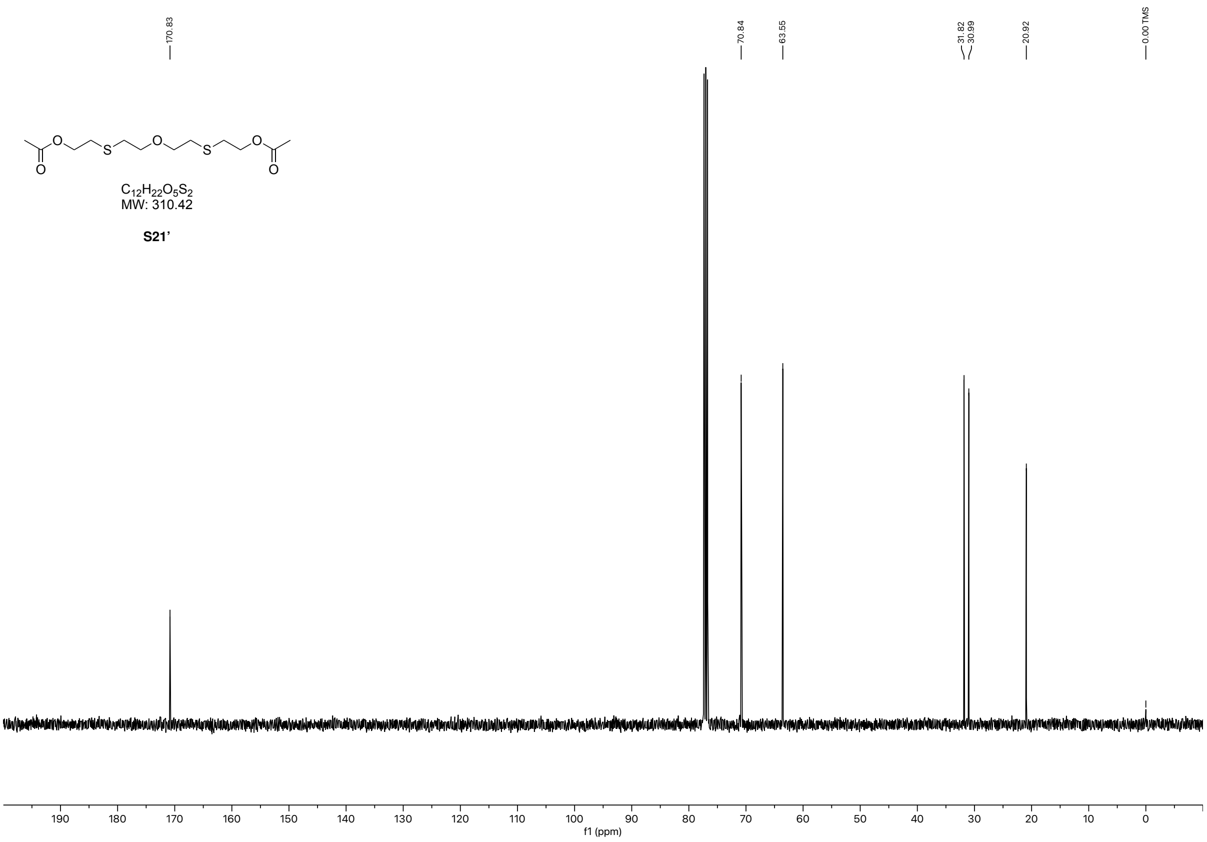

170.83
70.84
63.55
31.82
30.99
20.92
0.00 TMS
$C_{12}H_{22}O_5S_2$
MW: 310.42
S21'
f1 (ppm)

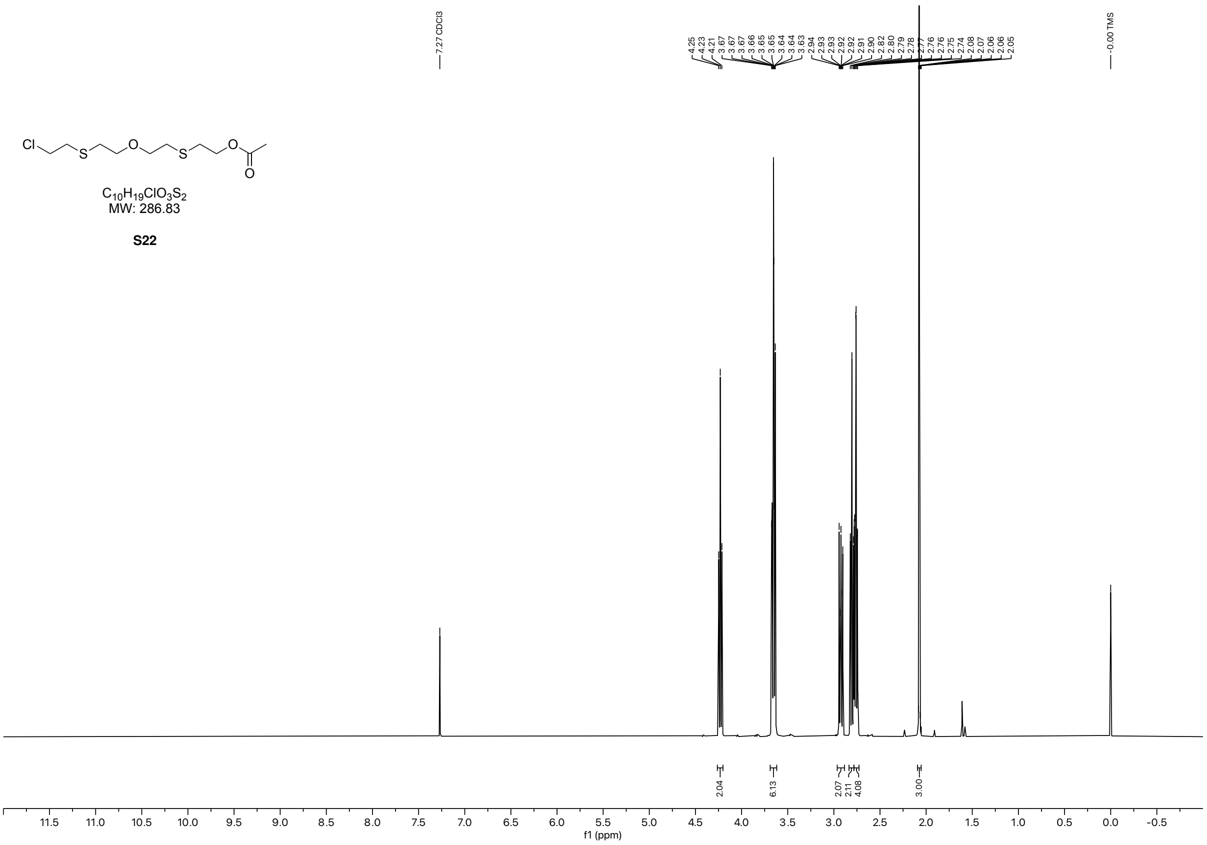

$C_{10}H_{19}ClO_3S_2$
MW: 286.83

S22

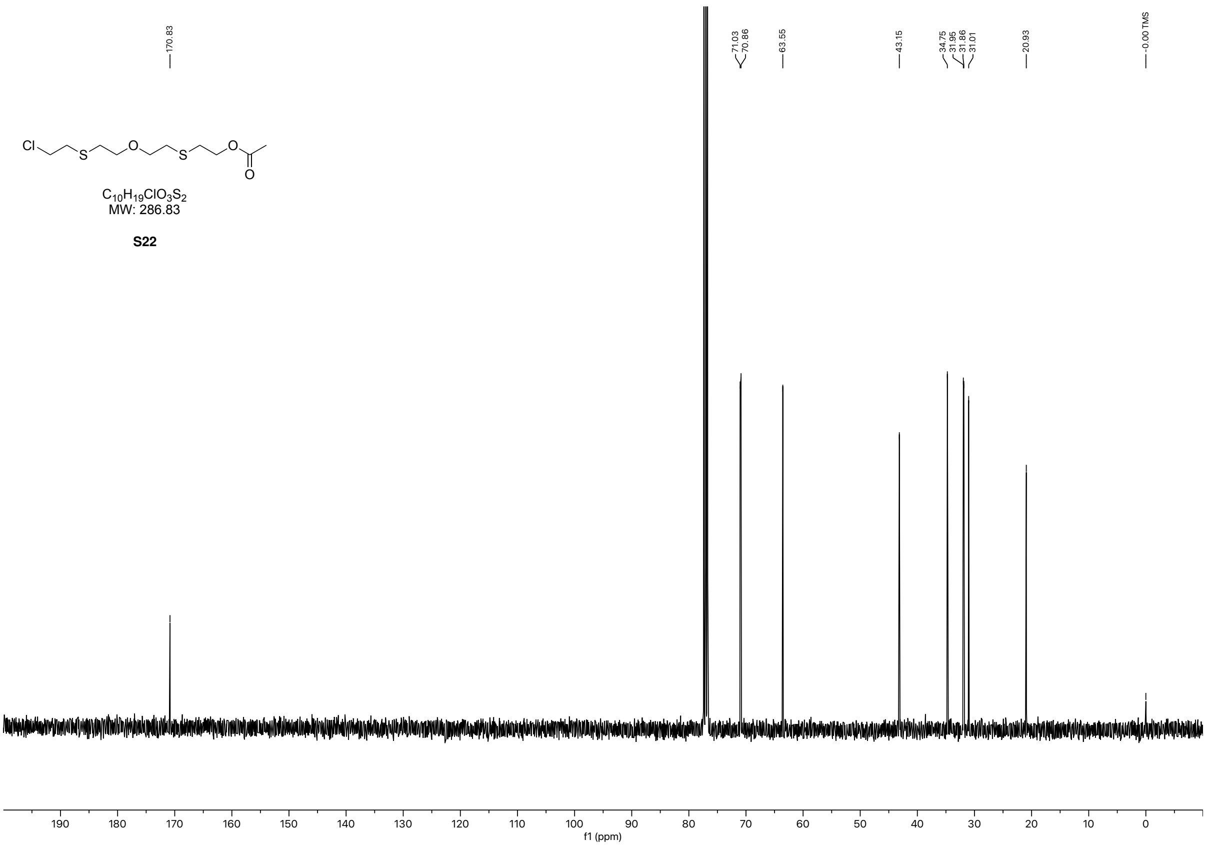

$C_{10}H_{19}ClO_3S_2$
MW: 286.83

S22

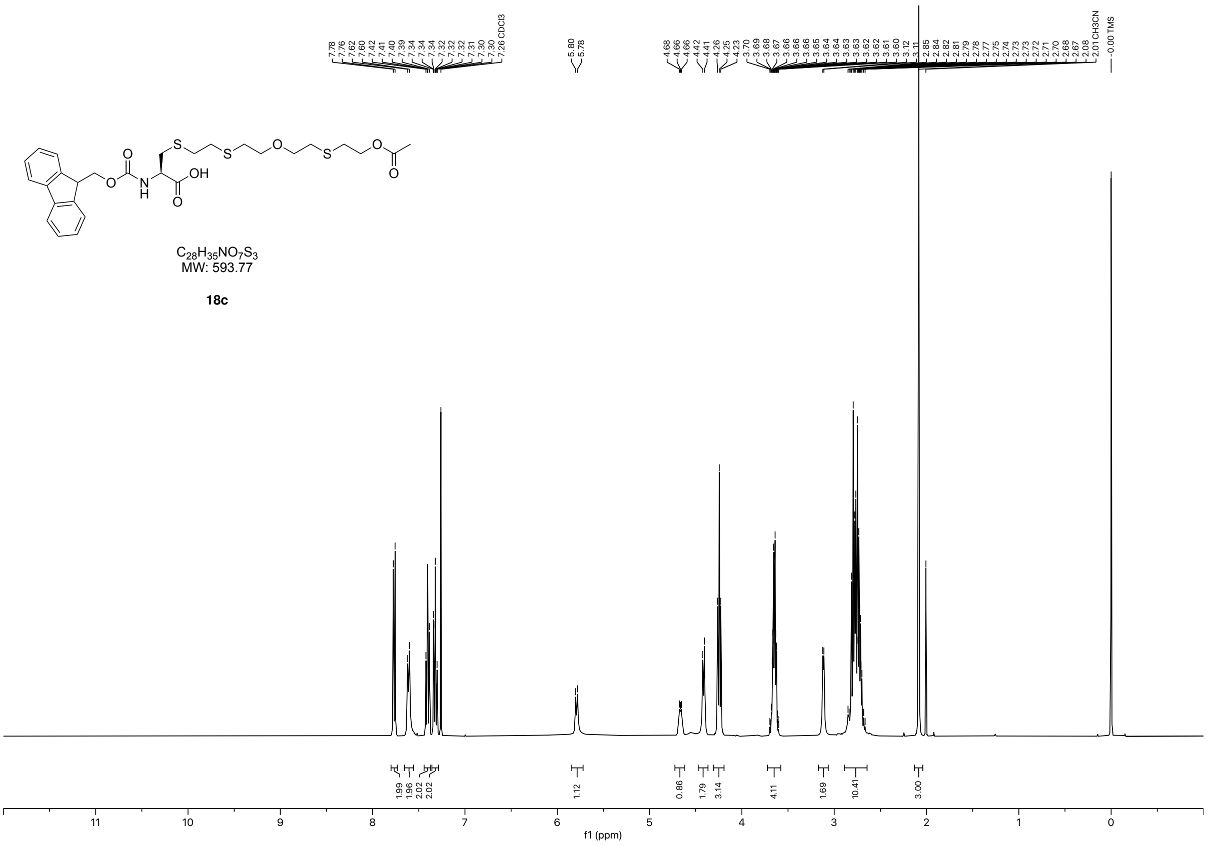

$C_{28}H_{35}NO_7S_3$
MW: 593.77

18c

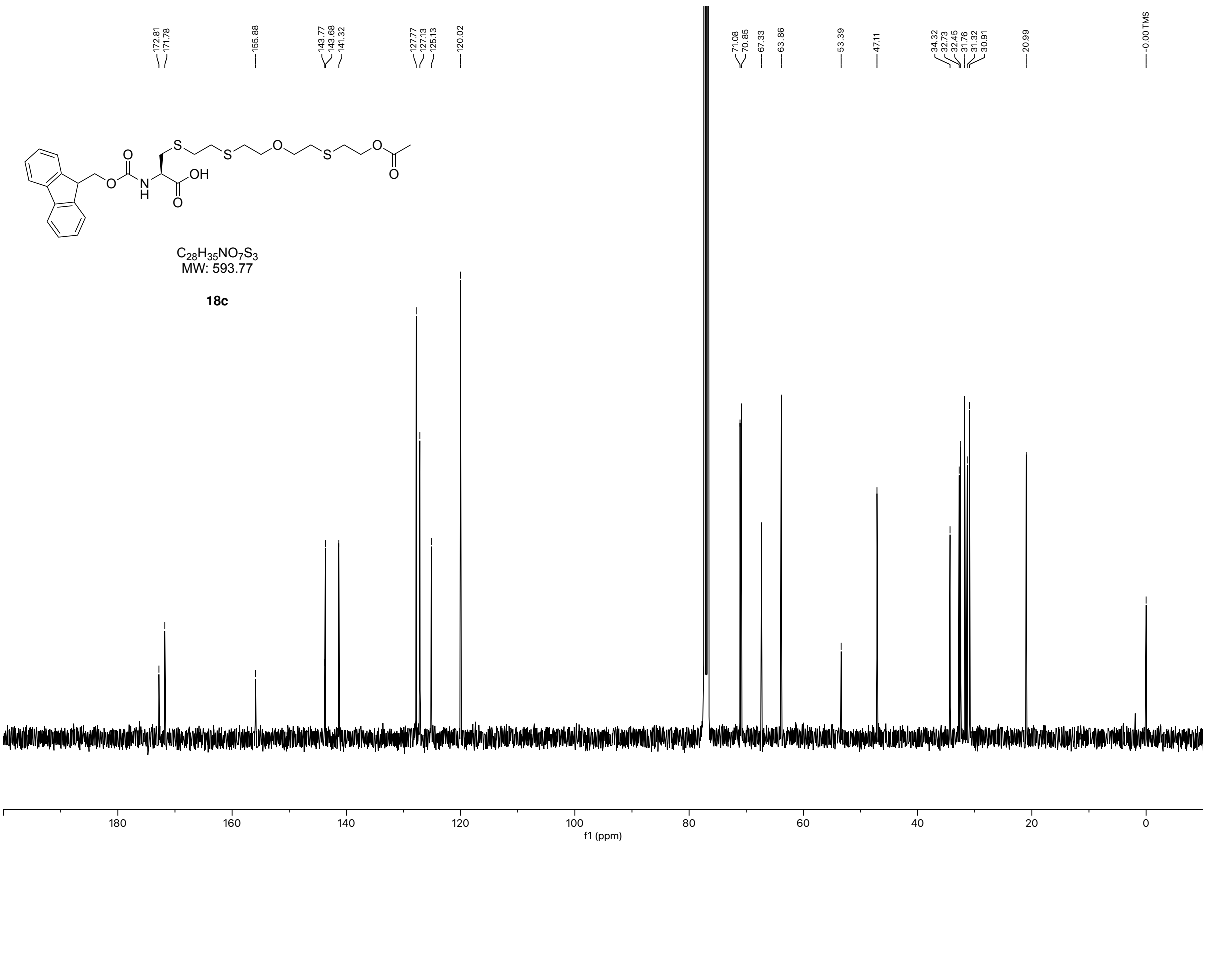

$C_{28}H_{35}NO_7S_3$
MW: 593.77

18c

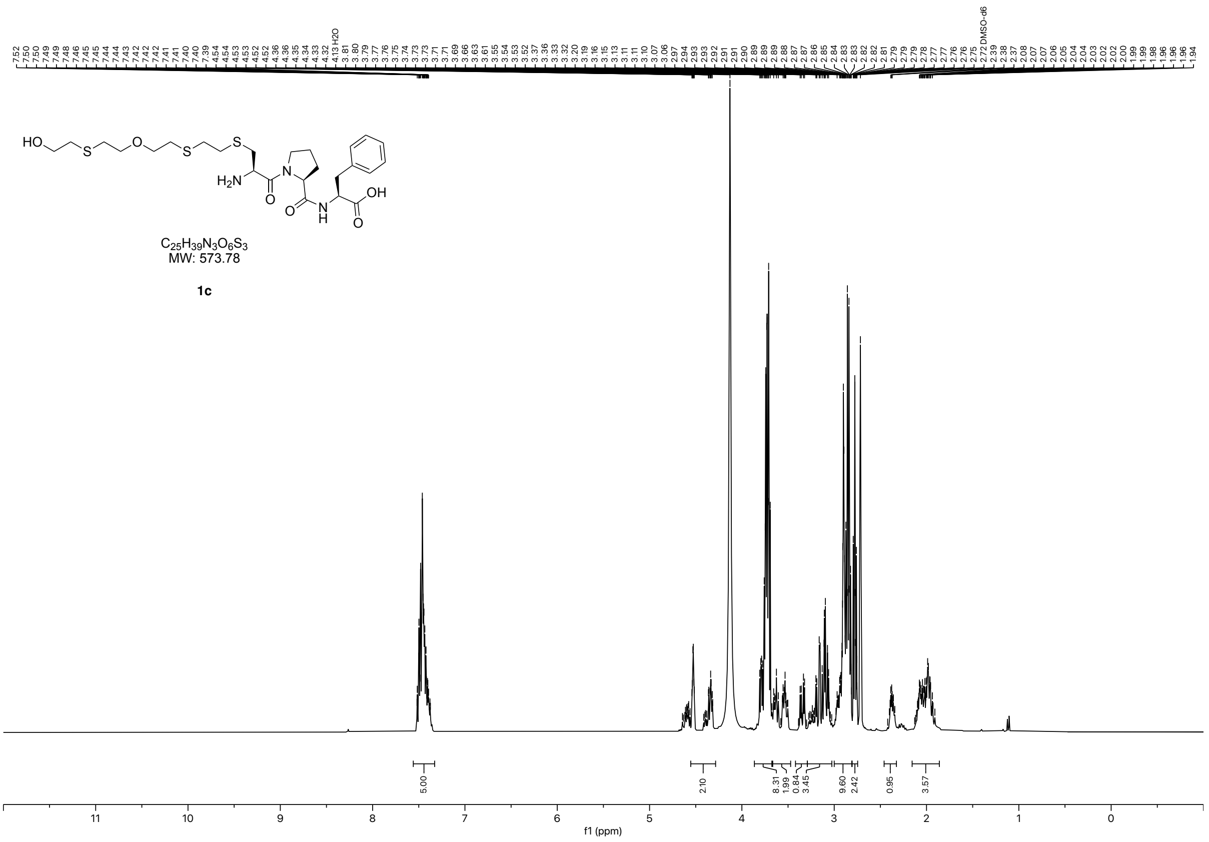

$C_{25}H_{39}N_3O_6S_3$
MW: 573.78
1c
f1 (ppm)

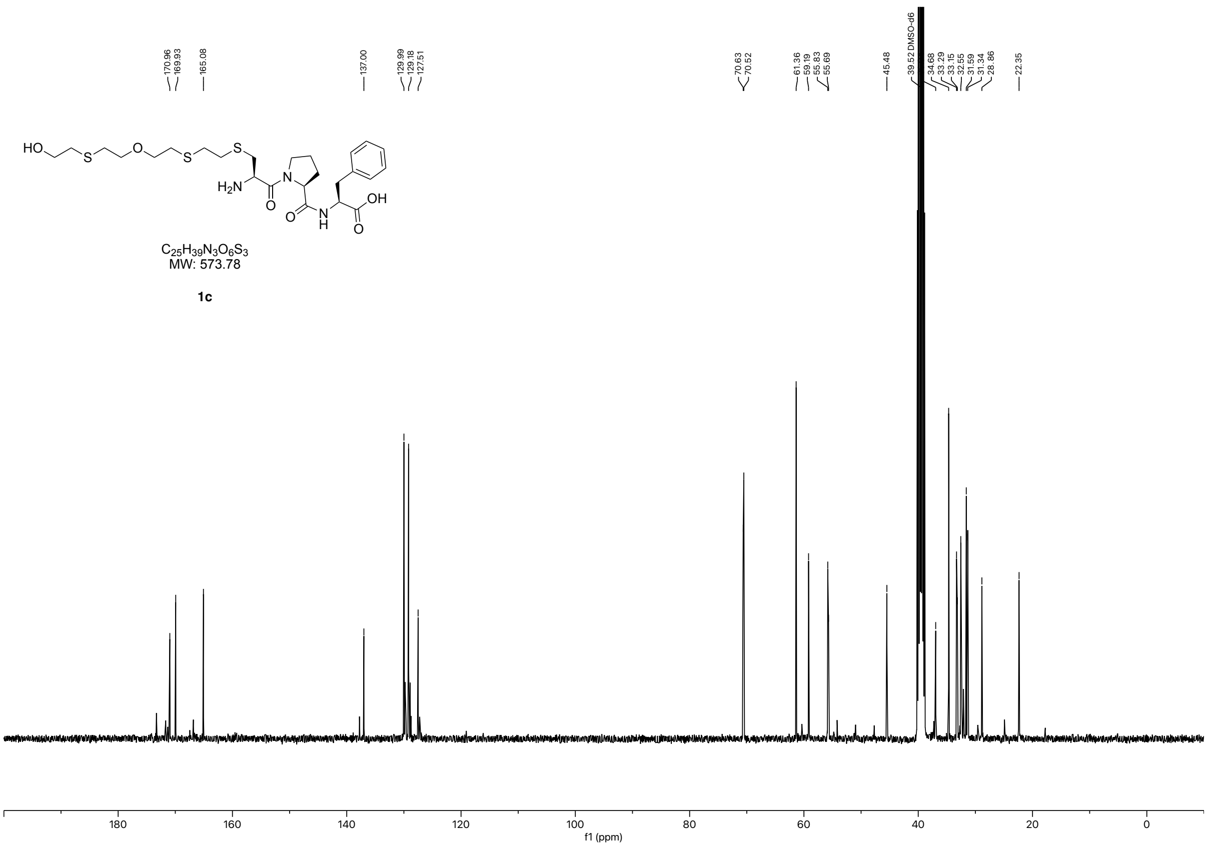

170.96
169.93
165.08
137.00
129.99
129.18
127.51
70.63
70.52
61.36
59.19
55.83
55.69
45.48
39.52 DMSO-d6
34.68
33.29
33.15
32.55
31.59
31.34
28.86
22.35
$C_{25}H_{39}N_3O_6S_3$
MW: 573.78
1c
f1 (ppm)

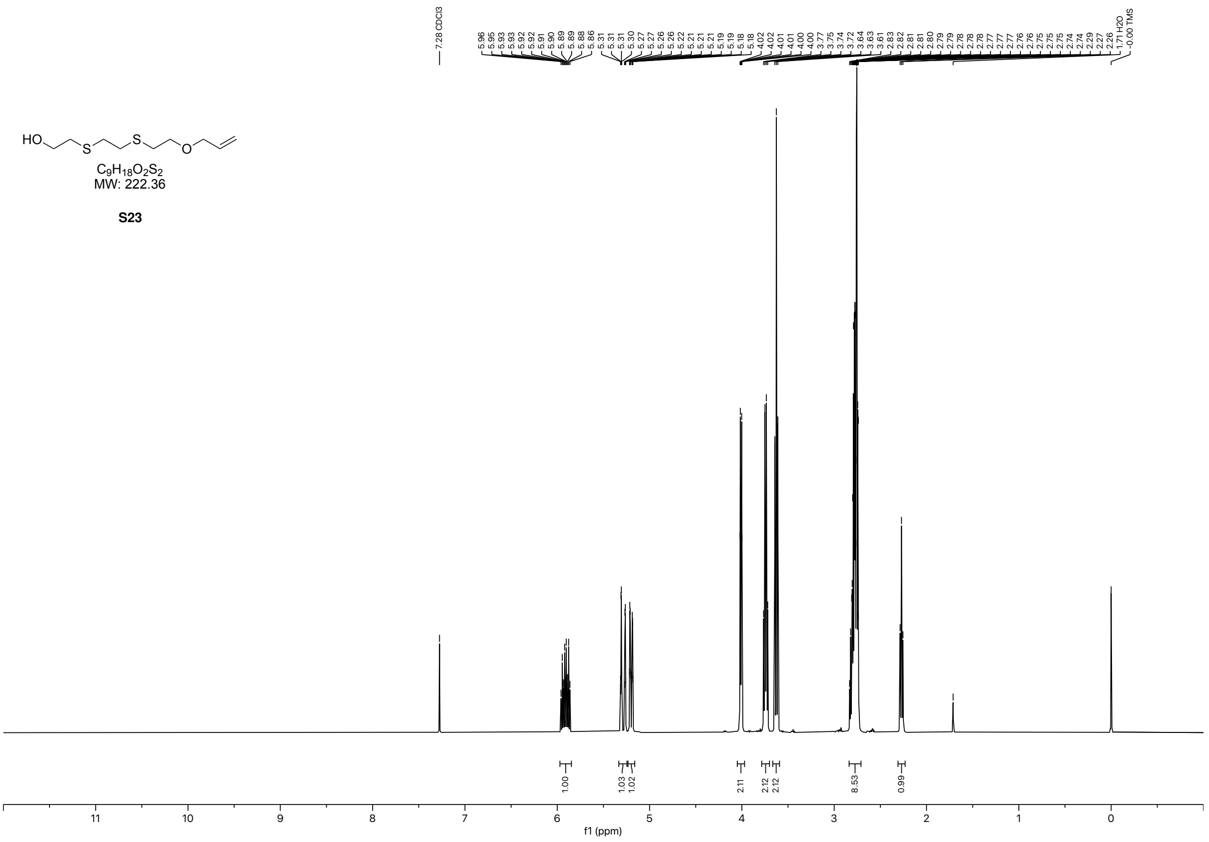
HO S S O
$C_9H_{18}O_2S_2$
MW: 222.36
S23
7.28 CDCl3
1.71 H2O
-0.00 TMS
1.00
1.03
1.02
2.11
2.12
2.12
8.53
0.99
f1 (ppm)

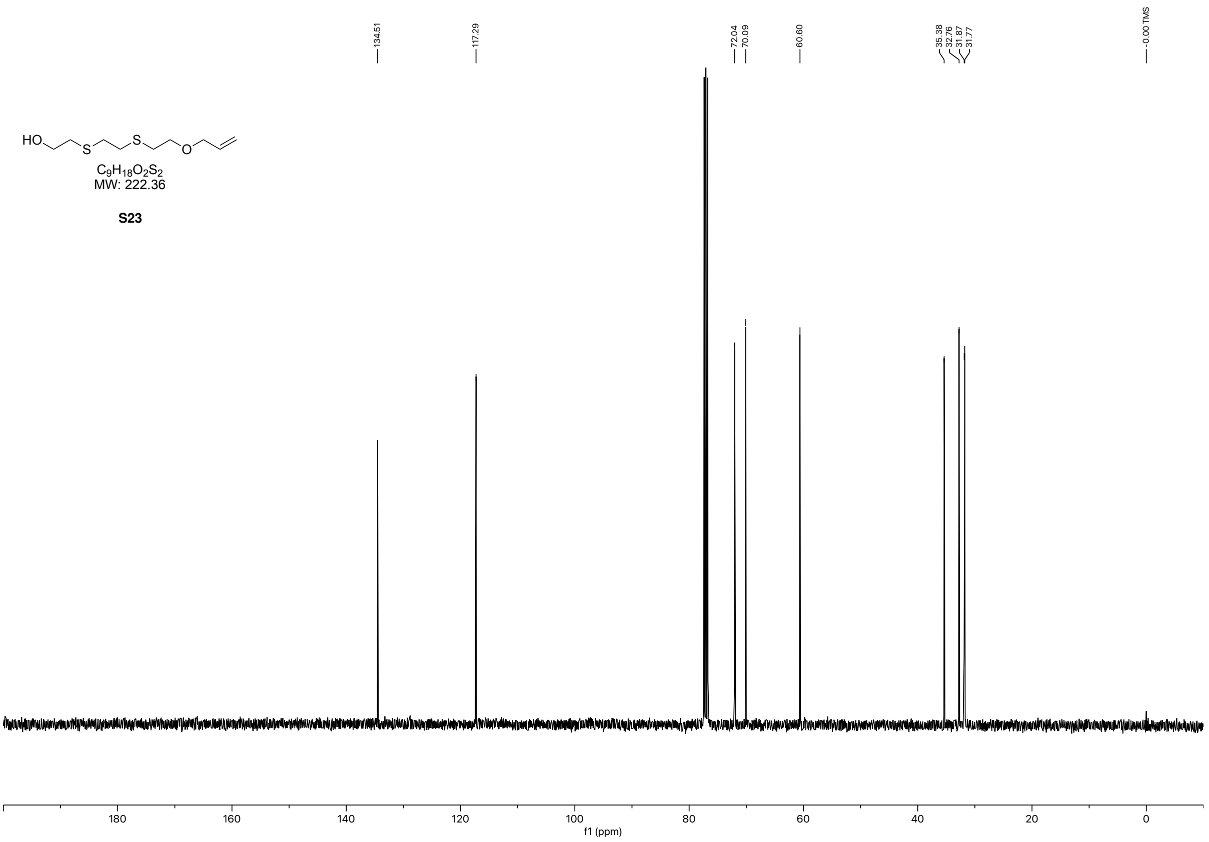
HO S S O
$C_9H_{18}O_2S_2$
MW: 222.36
S23
134.51
117.29
72.04
70.09
60.60
35.38
32.76
31.87
31.77
-0.00 TMS
f1 (ppm)

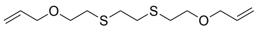

$C_{12}H_{22}O_2S_2$
MW: 262.43

S23'

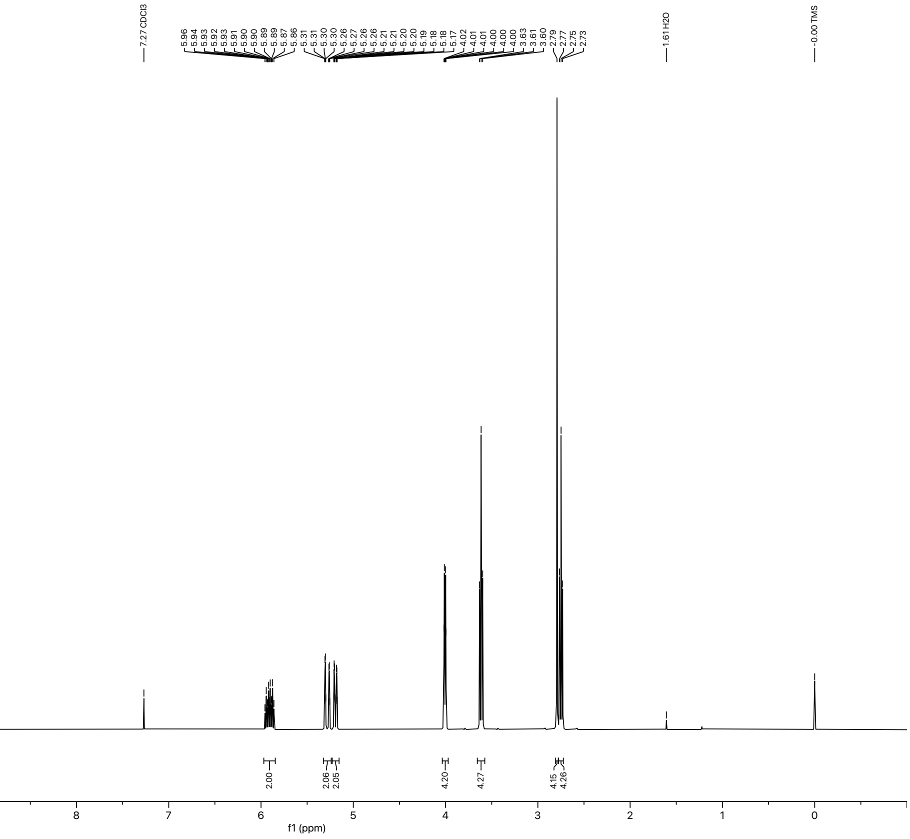

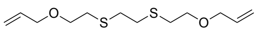

$C_{12}H_{22}O_2S_2$
MW: 262.43

S23'

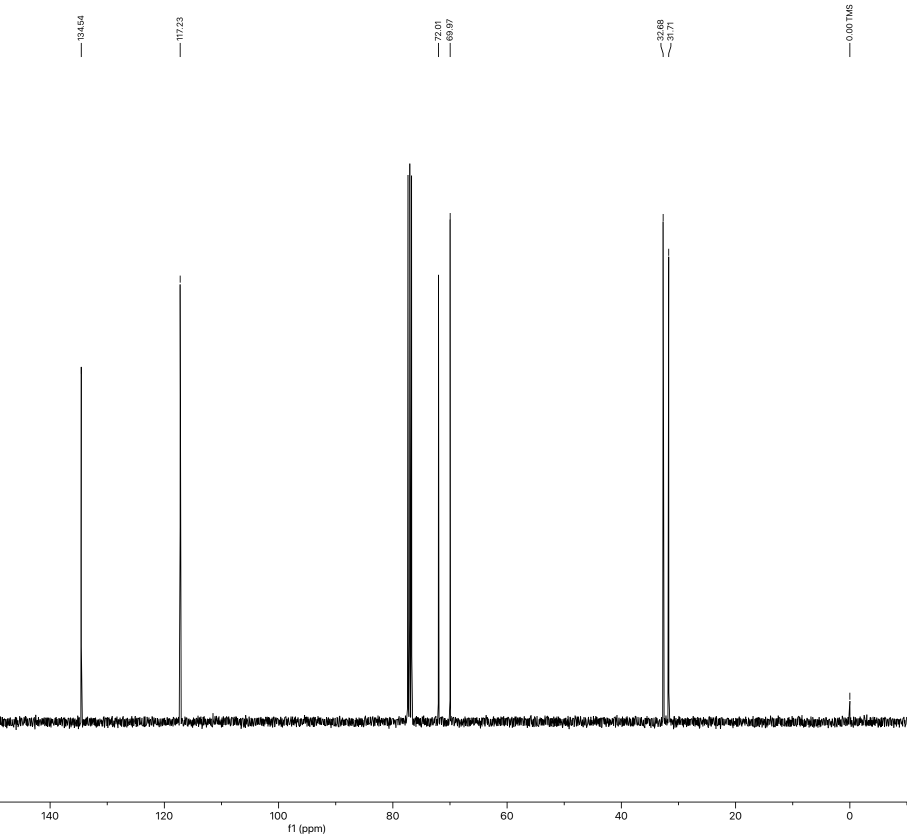

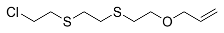

$C_9H_{17}ClOS_2$
MW: 240.80

S25

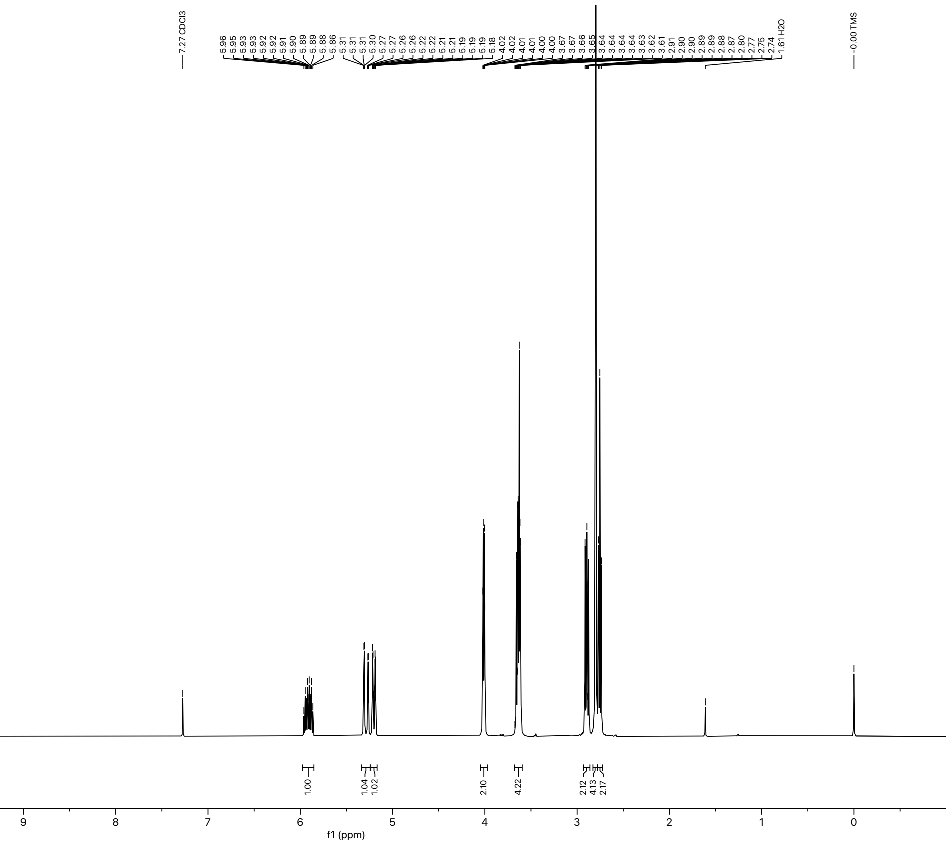

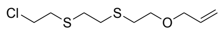

$C_9H_{17}ClOS_2$
MW: 240.80

S25

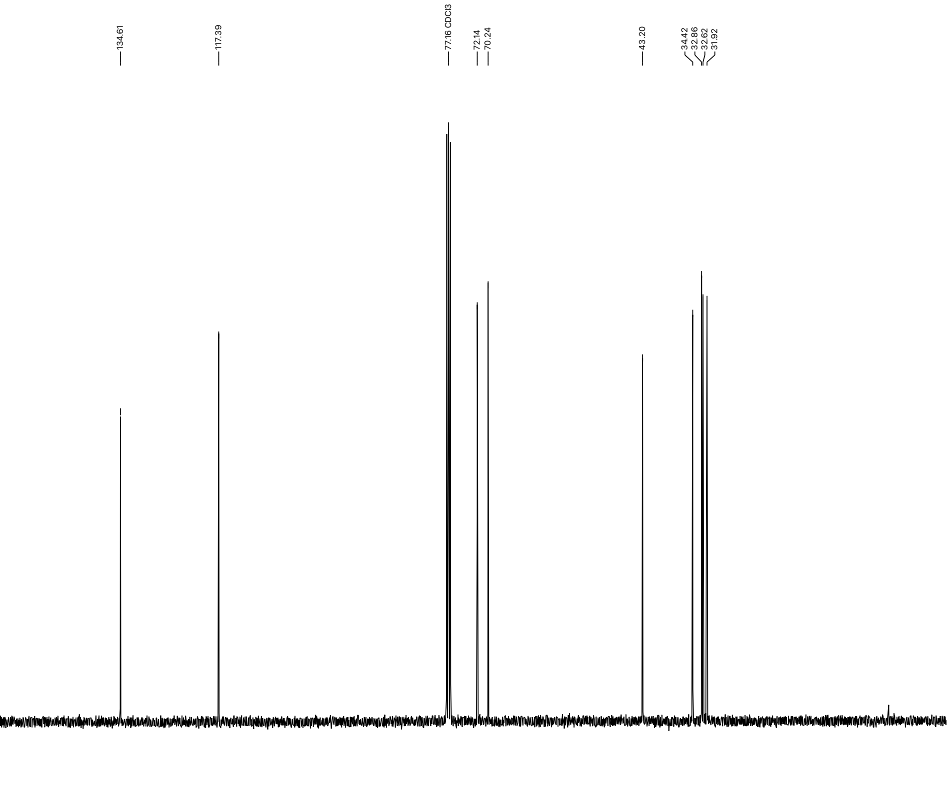

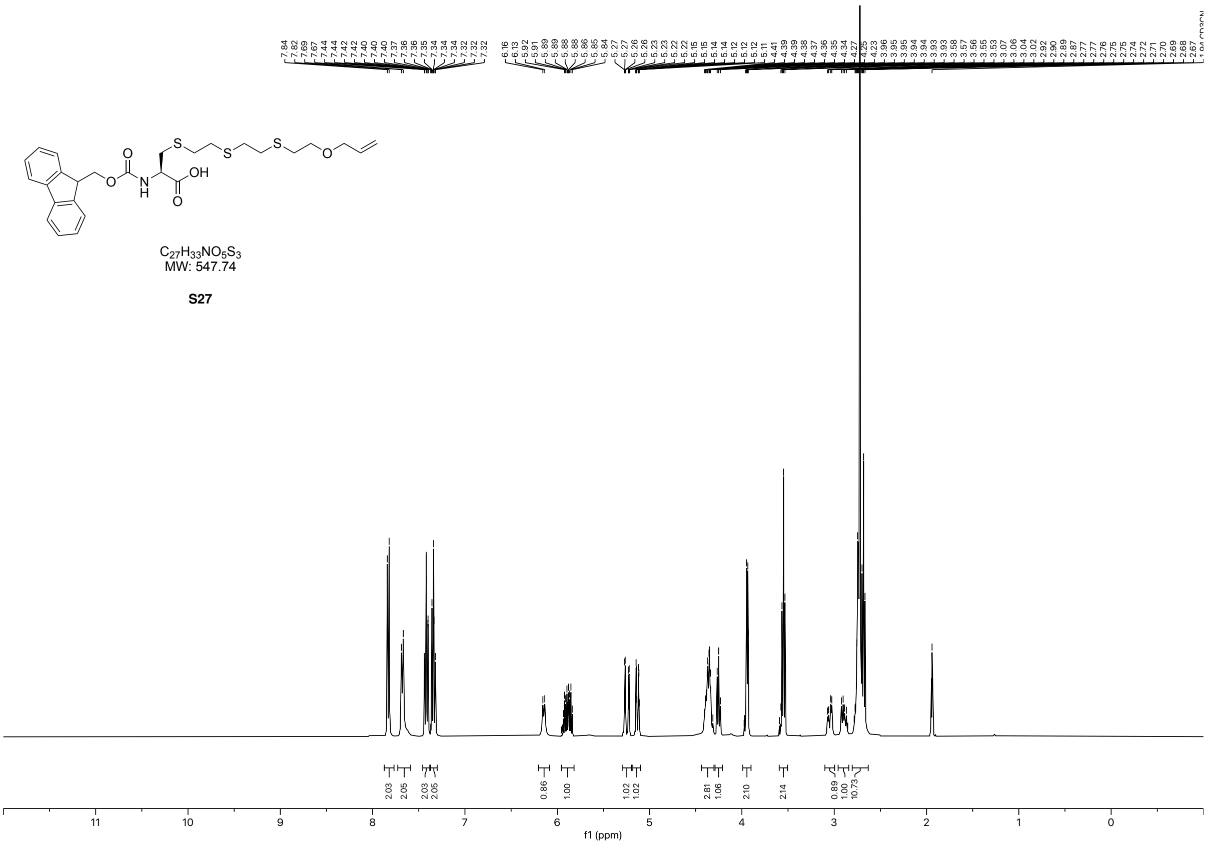
C27H33NO5S3
MW: 547.74
S27
7.84 7.82 7.69 7.67 7.44 7.44 7.42 7.42 7.40 7.40 7.37 7.36 7.36 7.35 7.34 7.34 7.34 7.32 7.32 7.32
6.16 6.13 5.92 5.91 5.89 5.89 5.88 5.88 5.86 5.85 5.84 5.27 5.27 5.26 5.26 5.23 5.23 5.22 5.22 5.15 5.15 5.14 5.14 5.12 5.12 5.12 5.12 5.11 4.41 4.39 4.39 4.38 4.37 4.36 4.35 4.34 4.27 4.25 4.23 3.96 3.95 3.95 3.94 3.94 3.93 3.93 3.58 3.57 3.56 3.55 3.53 3.07 3.06 3.04 3.02 2.92 2.90 2.89 2.87 2.77 2.77 2.76 2.75 2.75 2.74 2.72 2.71 2.70 2.69 2.68 2.67
2.03 2.05 2.03 2.05 0.86 1.00 1.02 1.02 2.81 1.06 2.10 2.14 0.89 1.00 10.73
f1 (ppm)

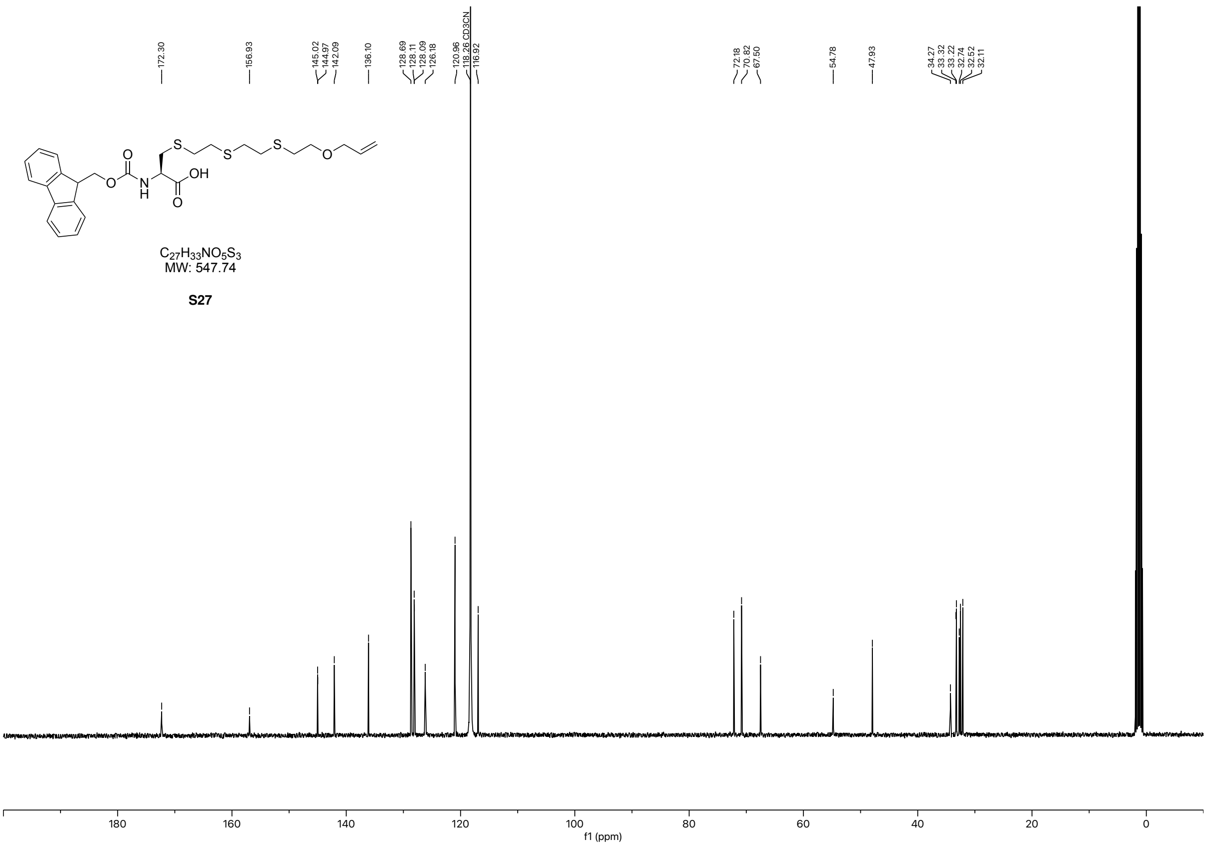
172.30
156.93
145.02 144.97 142.09
136.10
128.69 128.11 128.09 126.18
120.96 118.26 CD3CN 116.92
72.18 70.82 67.50
54.78
47.93
34.27 33.32 33.22 32.74 32.52 32.11
C27H33NO5S3
MW: 547.74
S27
f1 (ppm)

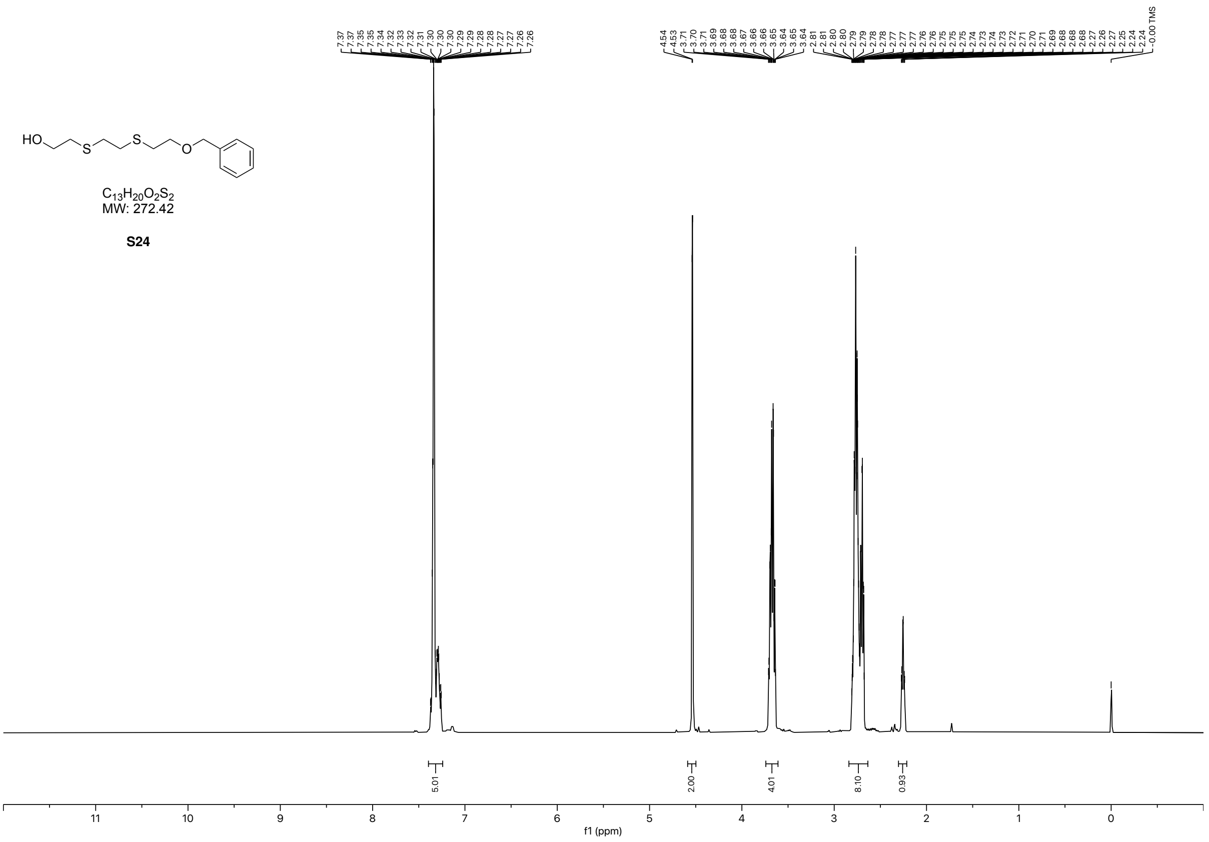
HO S S O
$C_{13}H_{20}O_2S_2$
MW: 272.42
S24
5.01
2.00
4.01
8.10
0.93
f1 (ppm)

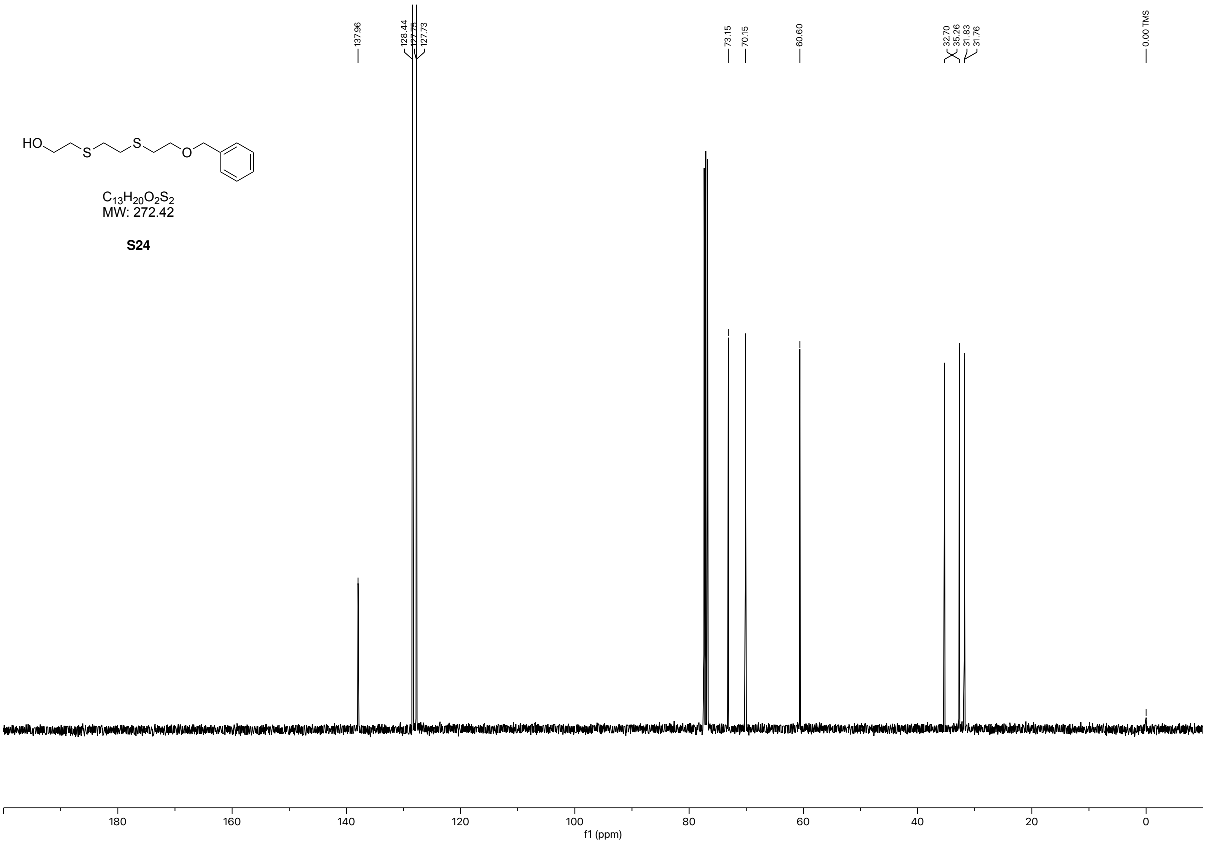
HO S S O
$C_{13}H_{20}O_2S_2$
MW: 272.42
S24
137.96
128.44
127.73
73.15
70.15
60.60
35.26
32.70
31.83
31.76
0.00 TMS
f1 (ppm)

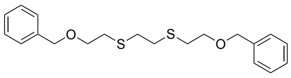

$C_{20}H_{26}O_2S_2$
MW: 362.55

S24'

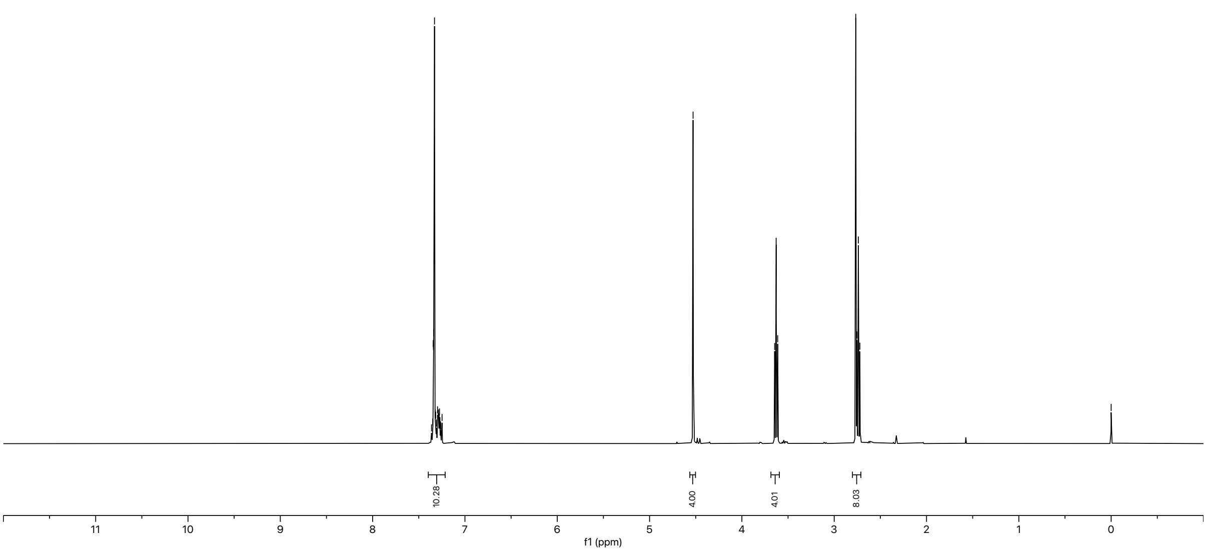

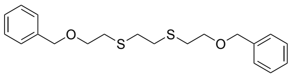

$C_{20}H_{26}O_2S_2$
MW: 362.55

S24'

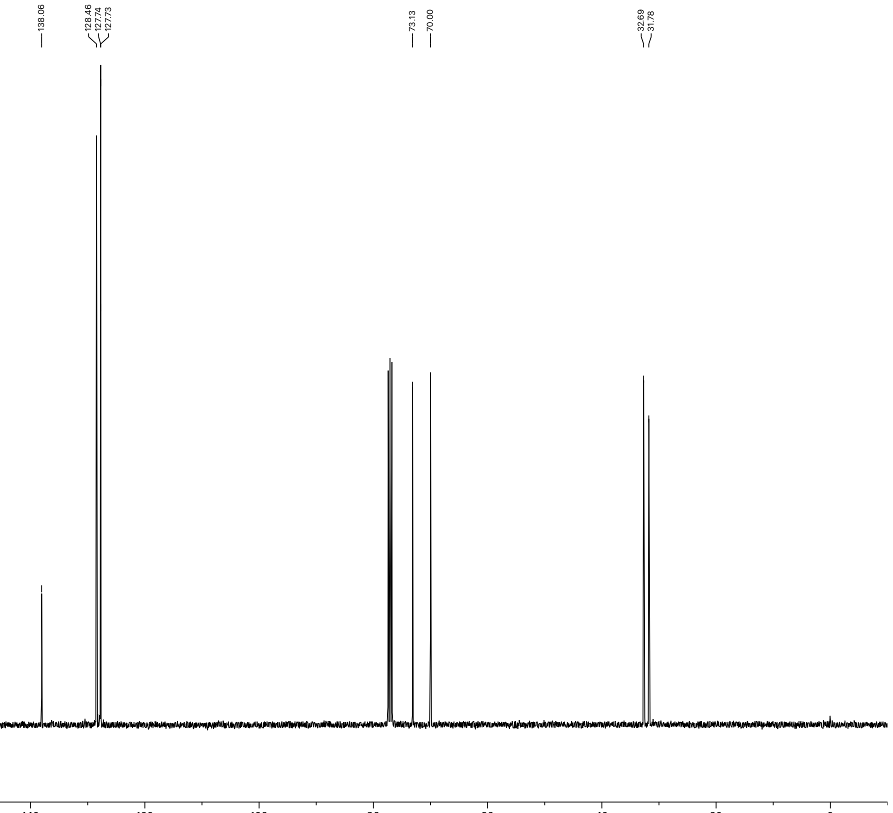

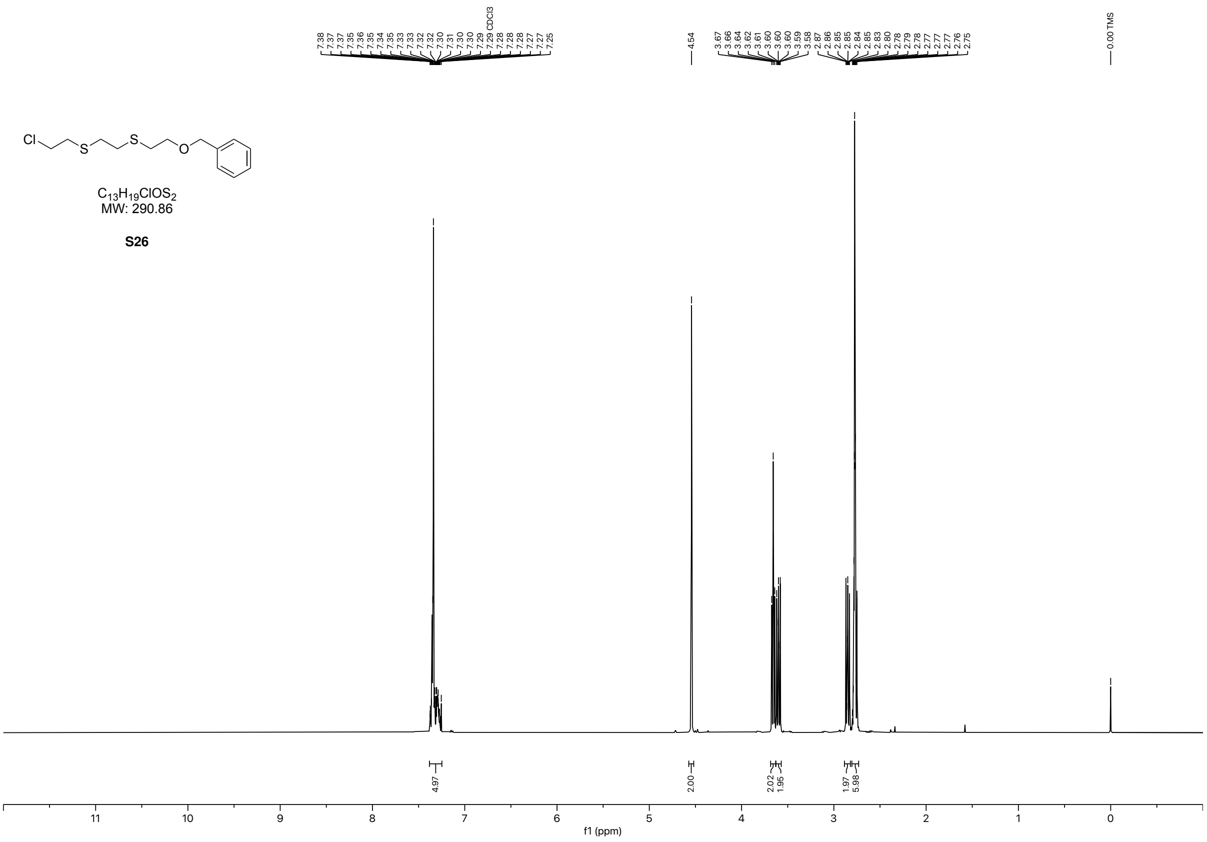
7.38 7.37 7.37 7.35 7.36 7.35 7.34 7.35 7.33 7.33 7.32 7.32 7.30 7.31 7.30 7.30 7.29 7.29 CDCl3 7.28 7.28 7.28 7.27 7.27 7.25
4.54
3.67 3.66 3.64 3.62 3.61 3.60 3.60 3.60 3.60 3.59 3.58
2.87 2.86 2.85 2.85 2.84 2.85 2.83 2.80 2.78 2.79 2.78 2.77 2.77 2.76 2.75
0.00 TMS
Cl S S O
$C_{13}H_{19}ClOS_2$
MW: 290.86
S26
4.97
2.00
2.02
1.95
1.97
5.98
11 10 9 8 7 6 5 4 3 2 1 0
f1 (ppm)

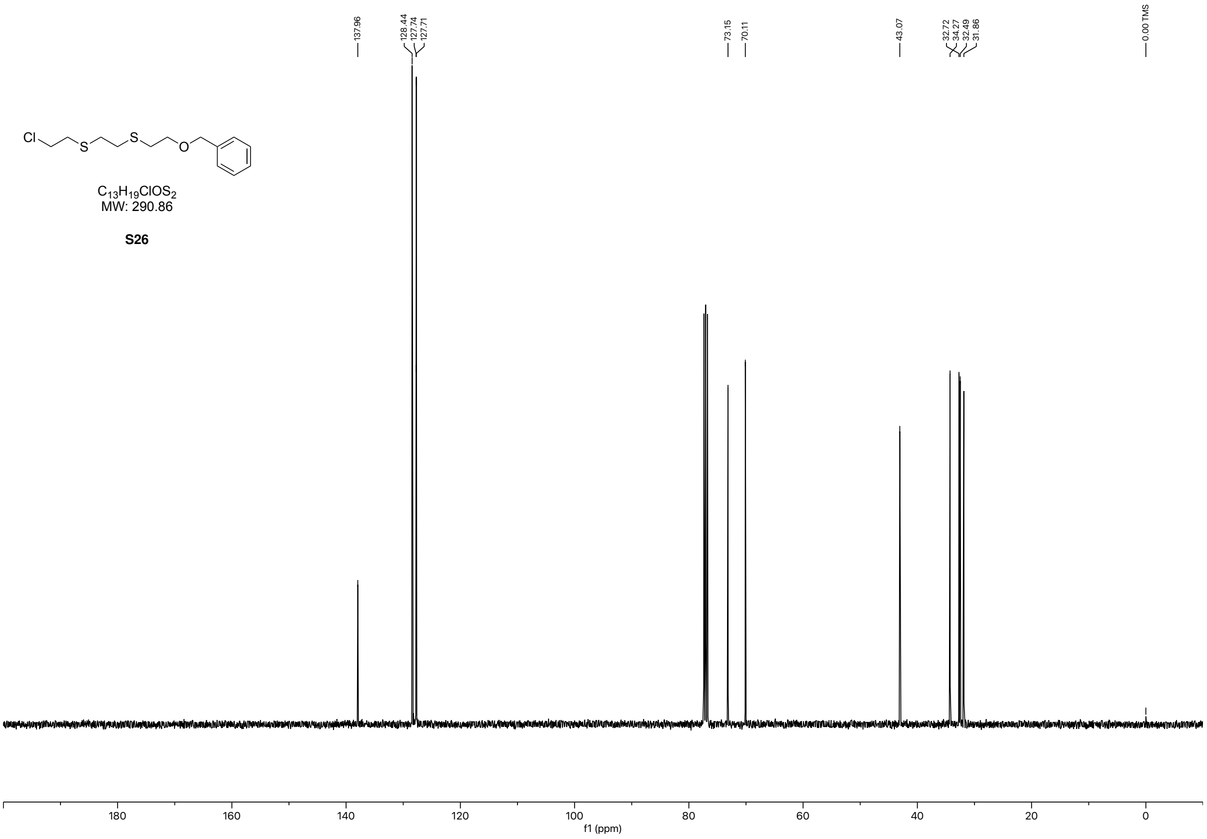
137.96
128.44 127.74 127.71
73.15
70.11
43.07
32.72 34.27 32.49 31.86
0.00 TMS
Cl S S O
$C_{13}H_{19}ClOS_2$
MW: 290.86
S26
180 160 140 120 100 80 60 40 20 0
f1 (ppm)

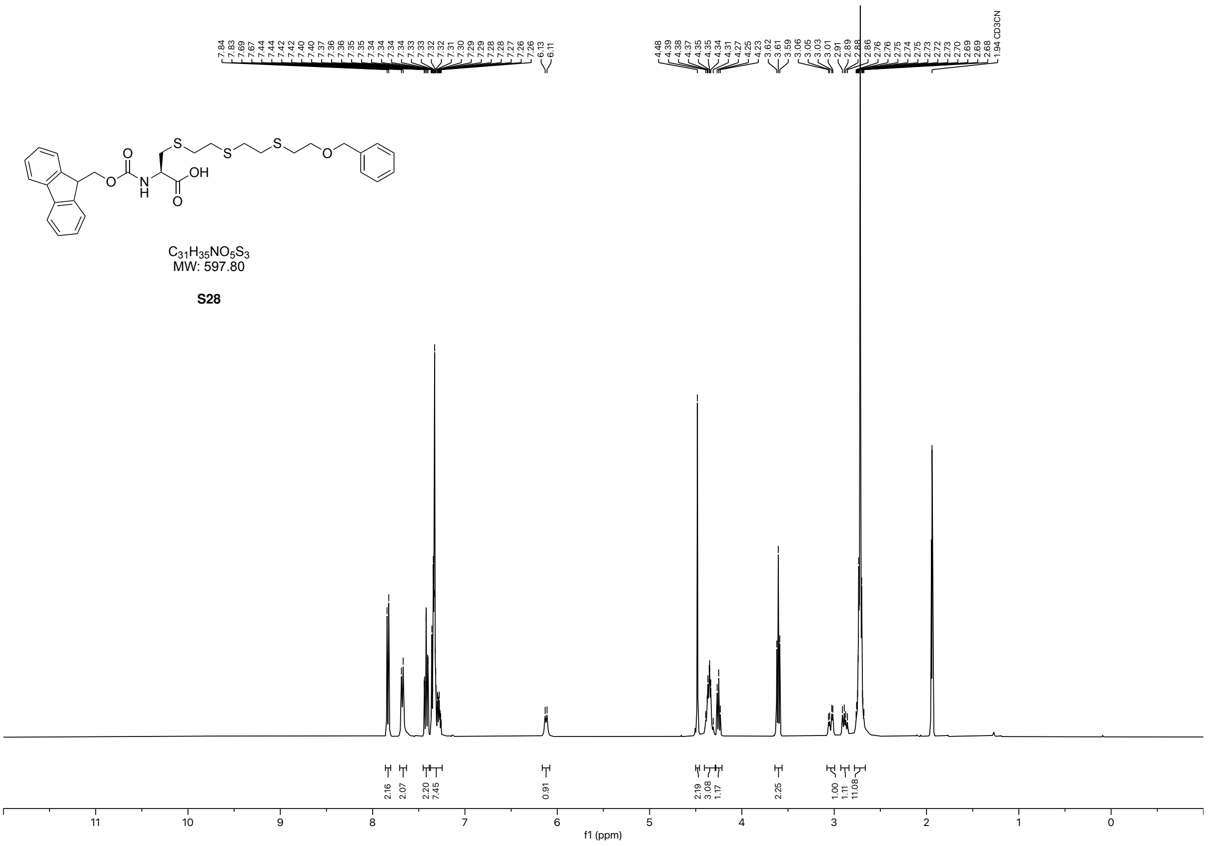
7.84
7.83
7.69
7.67
7.44
7.44
7.42
7.42
7.40
7.40
7.37
7.36
7.36
7.35
7.35
7.34
7.34
7.34
7.34
7.33
7.33
7.32
7.32
7.31
7.30
7.29
7.29
7.28
7.28
7.27
7.26
7.26
6.13
6.11
4.48
4.39
4.38
4.37
4.36
4.35
4.34
4.31
4.27
4.25
4.23
3.62
3.61
3.59
3.06
3.05
3.03
3.01
2.91
2.89
2.88
2.86
2.76
2.76
2.75
2.74
2.75
2.73
2.72
2.73
2.70
2.69
2.69
2.68
1.94 CD3CN
O
O
N
H
S
S
S
O
OH
O
$C_{31}H_{35}NO_5S_3$
MW: 597.80
S28
2.16
2.07
2.20
7.45
0.91
2.19
3.08
1.17
2.25
1.00
1.11
11.08
11
10
9
8
7
6
5
4
3
2
1
0
f1 (ppm)

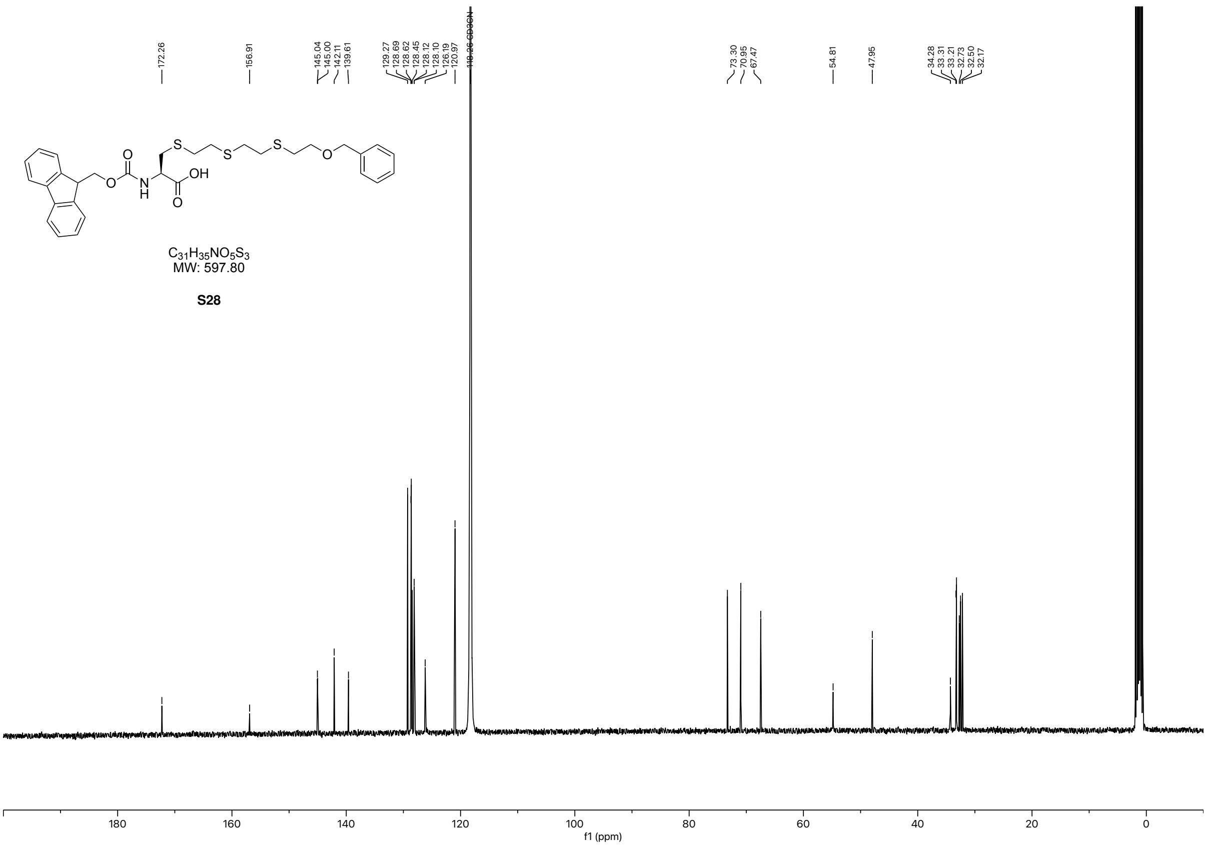
172.26
156.91
145.04
145.00
142.11
139.61
129.27
128.69
128.62
128.45
128.12
128.10
126.19
120.97
118.26 CD3CN
73.30
70.95
67.47
54.81
47.95
34.28
33.31
33.21
32.73
32.50
32.17
O
O
N
H
S
S
S
O
OH
O
$C_{31}H_{35}NO_5S_3$
MW: 597.80
S28
180
160
140
120
100
80
60
40
20
0
f1 (ppm)

2.3 MS/MS-Spectra of Synthetically Prepared Peptides

QNC(CAM)ELFE(HETE)QLGEYK (**2a**)

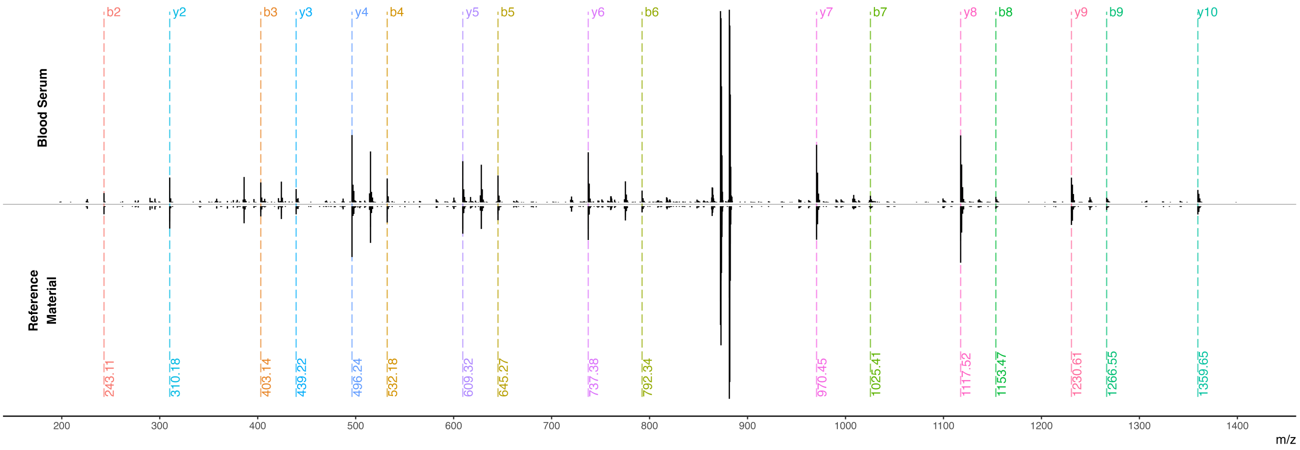

QNC(CAM)ELFE(HETETE)QLGEYK (**2b**)

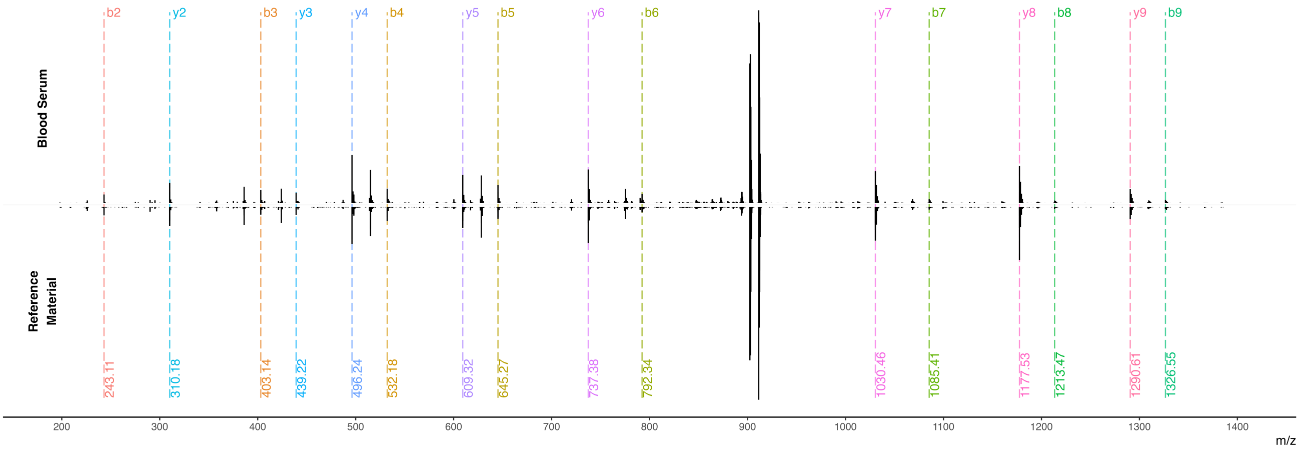

QNC(CAM)ELFE(HETEOETE)QLGEYK (**2c**)

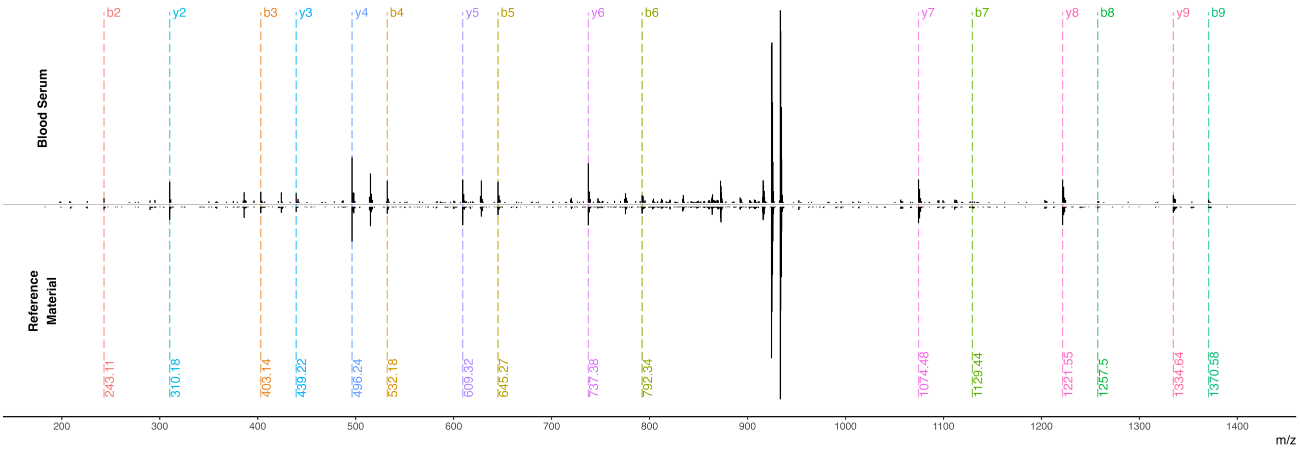

SLHTLFGD(HETETE)K (**3**)

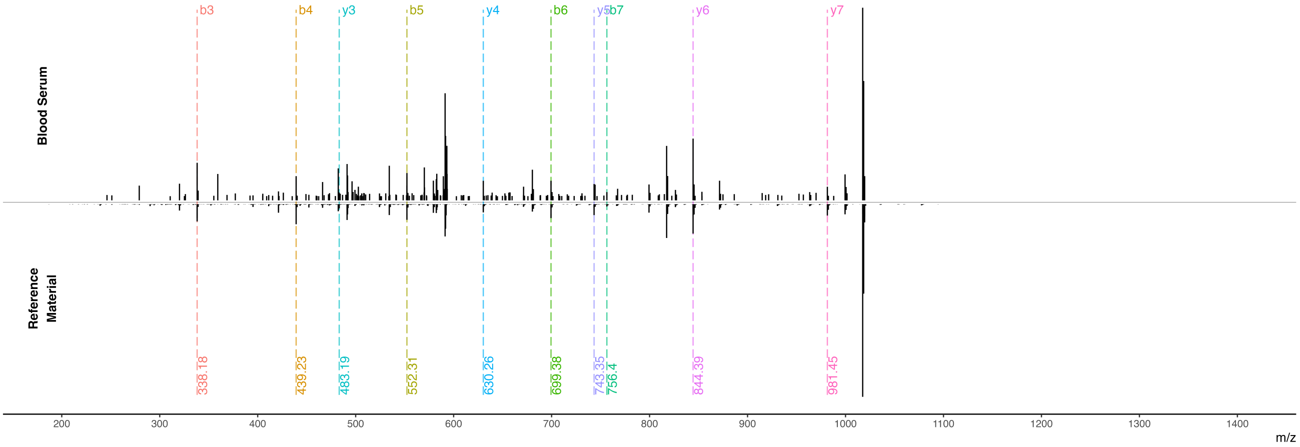

RH(HETETE)PDYSVVLLLR (**4a**)

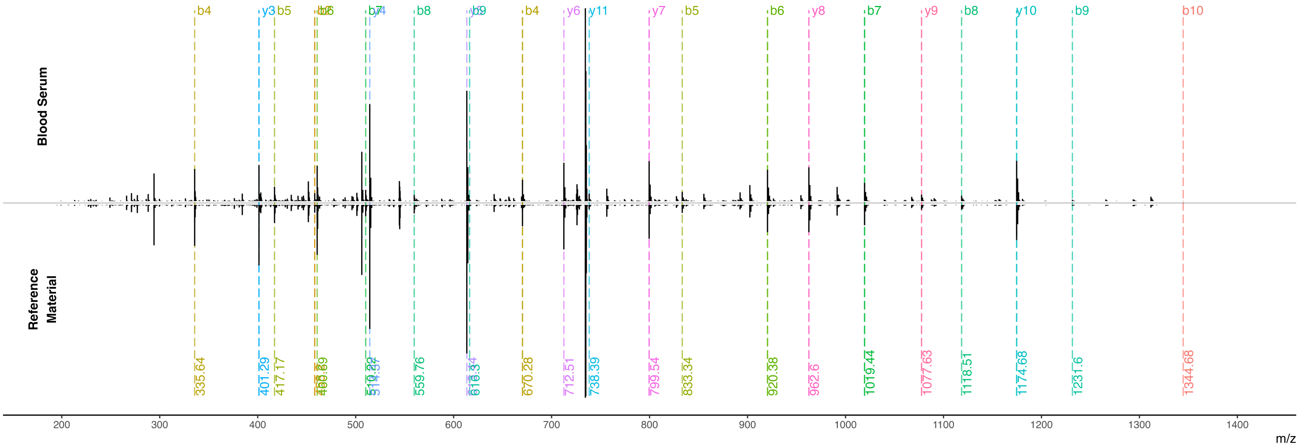

RH(HETETE)PDYSVVLLLR (**4b**)

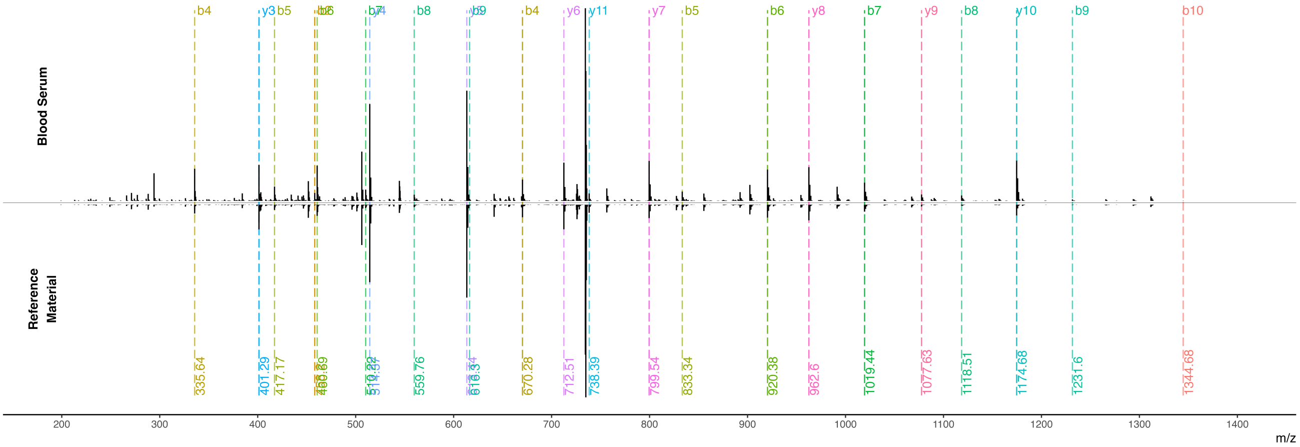

Supplement: Supplementary file 1 [file ac5c07620_si_001.pdf]
